# Reductive α-borylation of α,β-unsaturated esters using NHC–BH_3_ activated by I_2_ as a metal-free route to α-boryl esters†
†Electronic supplementary information (ESI) available: Full synthetic methods and characterization details (.PDF), computational details (.xyz) and crystallographic data (.cif). CCDC 1864774–1864779. For ESI and crystallographic data in CIF or other electronic format see DOI: 10.1039/c8sc04305a


**DOI:** 10.1039/c8sc04305a

**Published:** 2018-11-19

**Authors:** James E. Radcliffe, Valerio Fasano, Ralph W. Adams, Peiran You, Michael J. Ingleson

**Affiliations:** a School of Chemistry , University of Manchester , Manchester , M13 9PL , UK . Email: michael.ingleson@manchester.ac.uk

## Abstract

Useful α-boryl esters can be synthesized in one step from α,β-unsaturated esters using just a simple to access NHC–BH_3_ (NHC = N-heterocyclic carbene) and catalytic I_2_. The scope of this reductive α-borylation methodology is excellent and includes a range of alkyl, aryl substituted and cyclic and acyclic α,β-unsaturated esters. Mechanistic studies involving reductive borylation of a cyclic α,β-unsaturated ester with NHC–BD_3_/I_2_ indicated that concerted hydroboration of the alkene moiety in the α,β-unsaturated ester proceeds instead of a stepwise process involving initial 1,4-hydroboration; this is in contrast to the recently reported reductive α-silylation. The BH_2_(NHC) unit can be transformed into electrophilic BX_2_(NHC) moieties (X = halide) and the ester moiety can be reduced to the alcohol with the borane unit remaining intact to form β-boryl alcohols. The use of a chiral auxiliary, 8-phenylmenthyl ester, also enables effective stereo-control of the newly formed C–B bond. Combined two step ester reduction/borane oxidation forms diols, including excellent e.e. (97%) for the formation of *S*-3-phenylpropane-1,2-diol. This work represents a simple transition metal free route to form bench stable α-boryl esters from inexpensive starting materials.

## Abstract 2

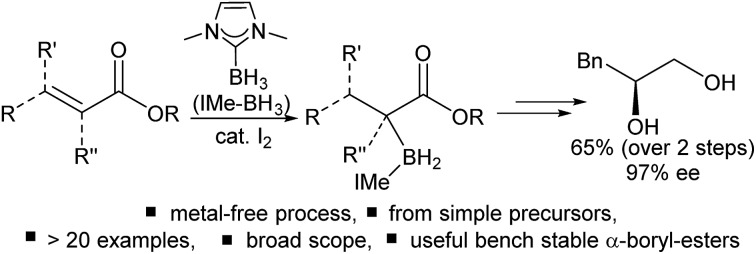
The combination of NHC–BH_3_/I_2_ represents a simple method for the reductive α-borylation of α,β-unsaturated esters to form useful α-boryl esters.

## Introduction

**Scheme 1:**
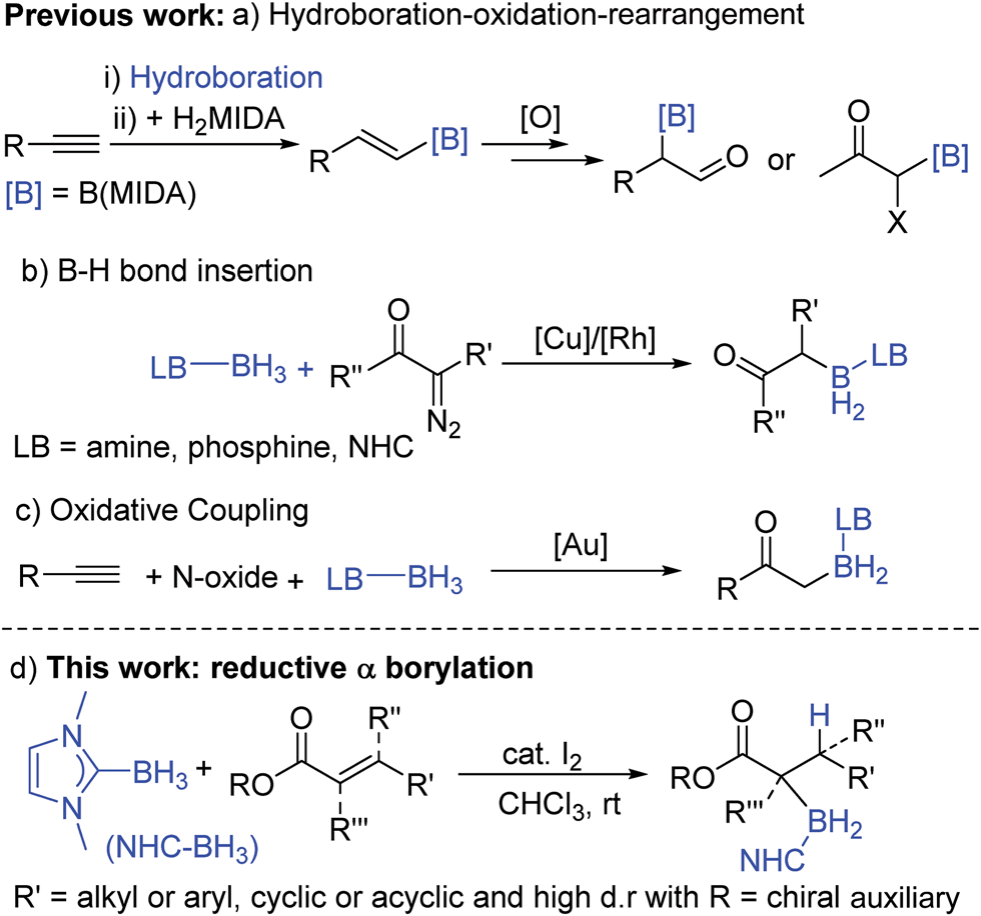
Previous main routes to quaternized α-boryl carbonyls and this work.

Organoboranes are ubiquitous in synthetic chemistry due to the wide range of C–Y (Y = C, N, O, *etc.*) bond forming reactions that can be carried out using these species.^1^,^2^ To this end, the discovery of new C–B bond forming reactions remains of importance and topical interest. The ability to form α-boryl carbonyl compounds is desirable as they are functionality rich amphoteric molecules that contain nucleophilic organoborane and electrophilic carbonyl moieties. Until recently, these compounds were largely overlooked in synthetic endeavours due to their instability with respect to formation of the O-boron enolate isomer. However, this has started to change in the last decade due to the quaternized at B stabilization approach which has enabled access to stable (with regard to C → O boryl migration) α-boryl-carbonyls thereby facilitating a range of subsequent transformations.^3^,^4^ MIDA-protected (MIDA = *N*-methyliminodiacetate) α-boryl carbonyls are the most explored to date and are accessed by a hydroboration–oxidation-rearrangement procedure (Scheme 1a).^3^,^4^ While notable, the multi-step nature and oxidising conditions indicate that there is a need for other routes. This is particularly the case if the new α-boryl carbonyls use boron protecting groups complementary to MIDA (in terms of stability/reagent compatibility).

In notable previous work, the use of base-stabilized boranes and α-diazo carbonyls enabled catalysed formation of alternative (to MIDA) quaternized at boron α-boryl carbonyls (Scheme 1b).^5^,^6^ In this area Curran *et al.* pioneered the use of NHC–BH_3_ (NHC = N-heterocyclic carbene) compounds to afford α-boryl carbonyls under transition metal or I_2_ catalysis.^7^,^8^ Base stabilised α-BH_2_-carbonyl products are bench stable and stable to strong bases/nucleophiles,^5^,^6^ and thus are complementary to MIDA-boronates which while being bench/acid stable are sensitive to strong bases.^9^ In comparison, α-Bpin esters and amides (produced *via* a novel Cu catalyzed 1,4-hydroboration/isomerization process) are not bench stable and are highly sensitive to protodeboronation due to their non-quaternized at B nature.^10^ Base stabilised organoboranes, including α-BH_2_-esters, can be utilised in a range of functional group transformations including the Suzuki–Miyaura reaction on appropriate activation.^5^–^11^ However, the current synthetic approaches to base stabilised α-BH_2_ carbonyls generally require diazo compounds (of which extremely limited examples are commercially available), precluding the formation of quaternary α-boryl carbonyls, while their more hazardous nature provides an additional drawback. Very recently, Zhu and co-workers demonstrated that α-boryl carbonyls also can be accessed by gold-catalysed oxidative coupling of terminal alkynes with Lewis base–borane adducts (Scheme 1c), while notable this is limited to forming primary α-boryl carbonyls.^12^ Therefore the formation of a wide range of bench stable α-boryl-carbonyl derivatives in one-pot from simple starting materials without using transition metal catalysis was an unsolved problem before this work.

**Scheme 2:**
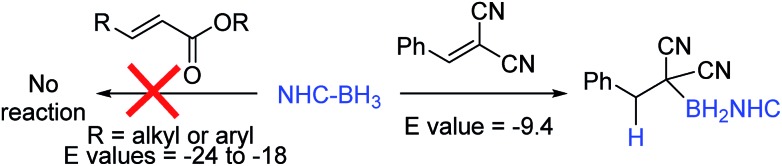
Previous work on reductive borylation of Michael acceptors using NHC–BH_3_.

An attractive route to α-boryl carbonyls that is the focus of this work is 3,4-hydroboration of α,β-unsaturated carbonyls, where the boryl group is selectively added at the 3 position, termed reductive α-borylation (Scheme 1d). While the reductive β-borylation of α,β-unsaturated carbonyl species has been reported,^13^,^14^ we are aware of no previous examples of selective reductive α-borylation of substrates such as cinnamates and crotonates. It should be noted that NHC–BH_3_ can reductively borylate strong Michael acceptors (Mayr *E* values > –18); however, the direct reaction between NHC–BH_3_ and α,β-unsaturated carbonyl derivatives that are weaker Michael acceptors does not proceed (ethyl cinnamate has an *E* value of –24.5 for example, Scheme 2).^15^ Furthermore, the catalysed hydroboration of α,β-unsaturated carbonyl compounds using common boranes (*e.g.* CatBH and PinBH) proceeds *via* 1,4-hydroboration.^16^ Recently, the Chang group reported the reductive α-silylation of α,β-unsaturated carbonyls, using silanes/B(C_6_F_5_)_3_.^17^ Previous work from some of us has indicated that on appropriate activation NHC–boranes can react with π nucleophiles in an analogous manner to silanes/B(C_6_F_5_)_3_.^18^ Therefore we hypothesised that NHC–BH_3_, upon activation, will react with α,β-unsaturated esters to generate NHC-stabilized α-boryl esters (Scheme 1d). Herein, we demonstrate that a wide range of α,β-unsaturated esters undergo highly selective reductive α-borylation using a simple NHC–BH_3_ compound and catalytic I_2_ as an activator. This represents a facile, metal free route to bench stable α-boryl-esters (including quaternary organoborane and diastereoselective examples) that are amenable to further functionalisation, for example to afford enantiopure diols.

## Results and discussion

**Scheme 3:**
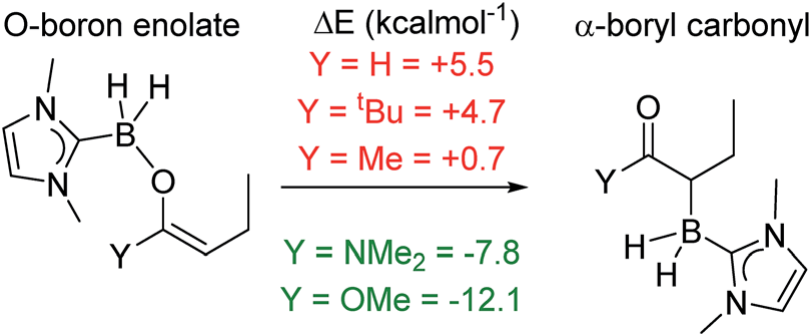
Calculated relative energies of O- and C-BH_2_(IMe) bound isomers.

This work commenced with the assessment of the relative stability of the O- and C-boron bound isomers derived from reductive borylation of α,β-unsaturated carbonyls using NHC–BH_3_. Notably, previous work calculated that α-boryl esters are thermodynamically favored compared to the O-boron enolate isomer when the boron moiety is B(diol), whereas when it is BMe_2_ the two isomers are effectively isoenergetic (Δ*G* < 1 kcal mol^–1^). The BMe_2_ analogue is more Lewis acidic at boron and thus also had a low energy barrier to interconversion between the O- and C-boron bound isomers.^19^ Reductive borylation will require electrophilic NHC–BH_2_Y (Y = I, NTf_2_*etc.*) species, which will possibly form NHC–BHY(*R*) on reaction with α,β-unsaturated esters before hydride transfer from NHC–BH_3_ occurs.^20^,^21^ Thus it was feasible that there would be a low barrier to interconversion between O- and C-BH_2_(NHC) bound isomers mediated by NHC–BHY(*R*). In this case the relative stability of the two isomers would dramatically affect the product distribution. Calculations therefore were carried out at the M06-2X/6-311G(d,p) level with a CH_2_Cl_2_ solvation model (polarizable continuum model, PCM). The α,β-unsaturated carbonyls analyzed were butenal, pentenone, 2,2-dimethylhexenone, *N*,*N*-dimethyl buteneamide and methyl crotonate (Scheme 3). The NHC–borane adduct used was IMe–BH_3_ (IMe = 1,3-dimethyl-imidazol-2-ylidene), as it is the smallest and most readily accessed NHC–borane.^12^ For the ketones and aldehydes it was found that the O-boron enolates were favored over the α-boryl carbonyl isomers. In contrast, it was found that for the ester and amide substrates, the α-boryl carbonyl products were more stable (by 12.1 and 7.8 kcal mol^–1^, respectively). Based upon these findings we investigated the borylation of unsaturated esters and amides using NHC–BH_3_/activator.

**Fig. 1:**
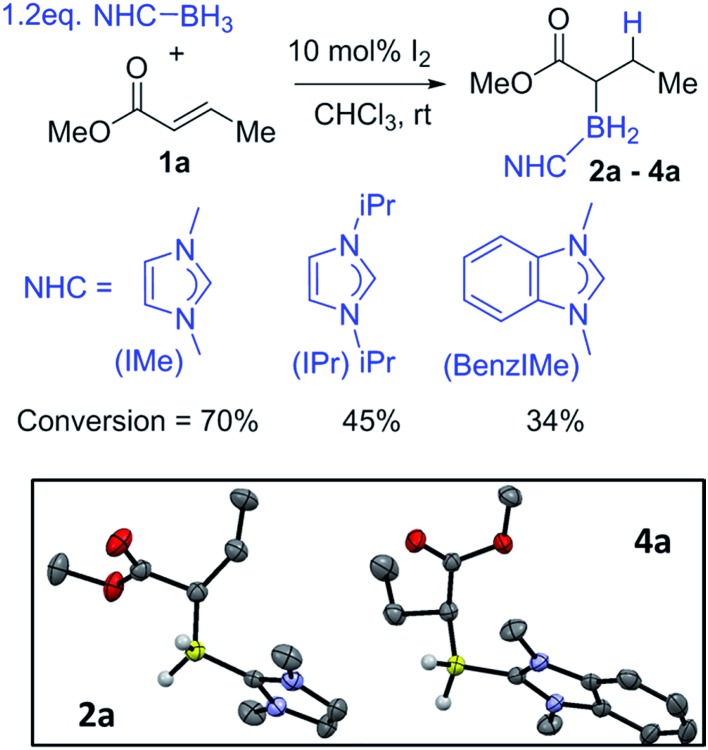
Top: formation of α-boryl carbonyls **2a–4a**. Inset left: solid-state structure of **2a**, inset right: solid-state structure of **4a**, ellipsoids at the 50% probability level. Most hydrogens omitted for clarity.

For reaction optimisation IMe–BH_3_ and I_2_ were selected due to their low cost (or simple synthesis), ease of handling (IMe–BH_3_ is bench stable) and facile reactivity to give electrophilic IMe–BH_2_I.^7^,^20^,^21^ Methyl crotonate, **1a**, was mixed with IMe–BH_3_ in CDCl_3_ followed by addition of 10 mol% I_2_ (Fig. 1, top). NMR spectroscopic analysis after 2 h showed a new triplet ^11^B resonance at –25.4 ppm (^1^*J*_BH_ = 90 Hz) consistent with the desired reductive α-borylation product, **2a** (Fig. 1). However, under these conditions longer reaction times did not lead to acceptable conversion, with the retention of a significant amount of methyl crotonate even after 2 days. Optimization studies (see ESI†) identified increasing the concentration as key, as previously observed by Curran and co-workers,^7^ with a 1 M reagent concentration leading to the highest yield in a reasonable timeframe (*ca.* 70% conversion after 20 hours at room temperature). It should be noted that other electrophilic activators previously used with NHC–BH_3_, *e.g.* HNTf_2_ and B(C_6_F_5_)_3_, led to poorer outcomes.^22^ Recent work has demonstrated that radical initiators can enable alkyne cyclisation reactions and alkyne *trans*-hydroboration using NHC–BH_3_.^23^,^24^ Therefore the use of *tert*-butylhydroperoxide (TBHP) was explored under comparable conditions to those reported for *trans*-hydroboration. However, heating a mixture of IMe–BH_3_, **1a** and 20 mol% TBHP in benzene for 18 hours led to no reductive α-borylation.

**Fig. 2:**
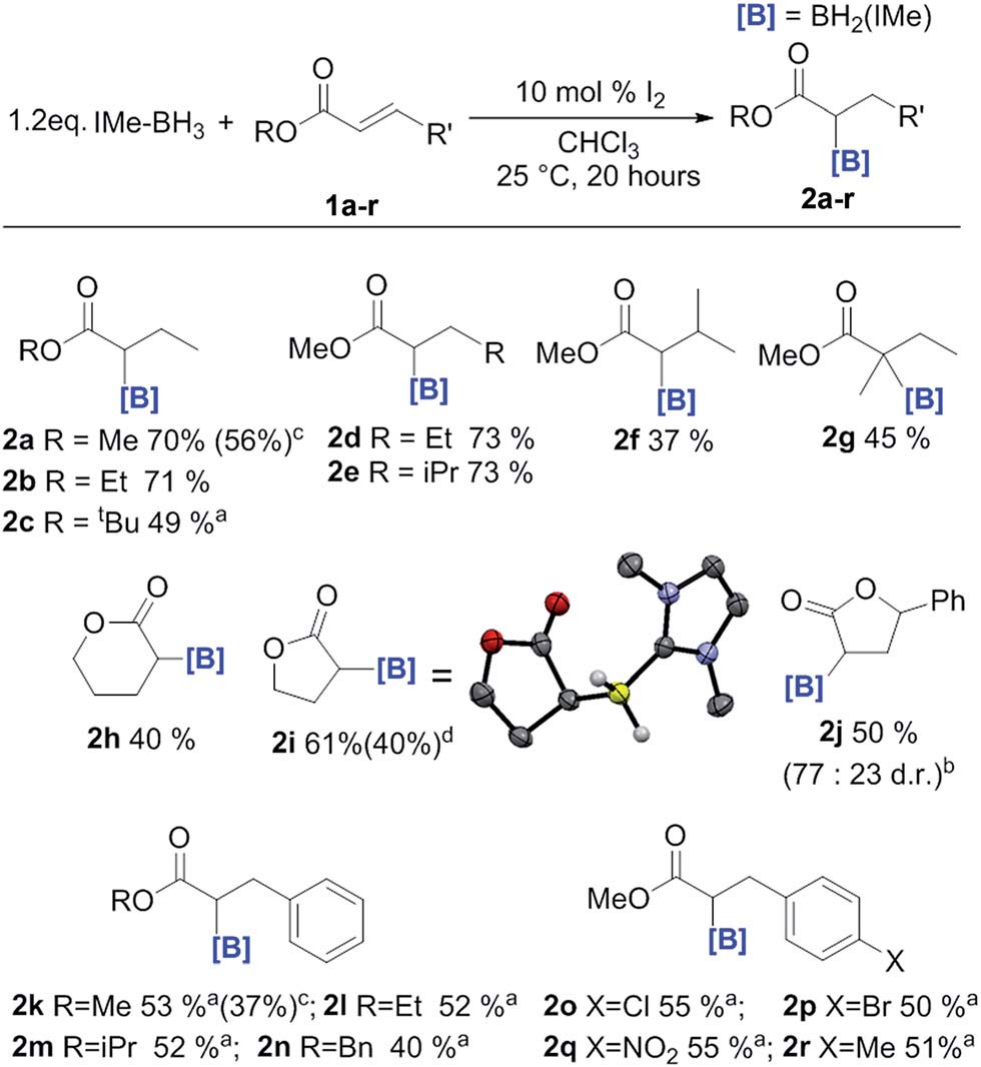
Substrate scope of the reductive α-borylation reaction. (a) = 20 mol% I_2_. (b) = diastereomeric ratios obtained by ^1^H NMR analysis of the reaction mixture. (c) = yields from *in situ*^1^H NMR spectroscopy *versus* an internal standard. Isolated yields are provided in parentheses. (d) = isolated yield of the d_2_ isotopomer.

In order to probe the effect of varying the NHC, ^i^Pr–BH_3_ and BenzIMe–BH_3_ were explored (Fig. 1). While both of these NHC–boranes successfully led to reductive α-borylation of **1a** upon activation with I_2_, the conversions were lower than when using IMe–BH_3_ under identical conditions (70% *versus* 43 and 34%, respectively), thus in further reactions IMe–BH_3_ was used. X-ray diffraction studies (Fig. 1, inset) further confirmed the formulation of **2a** and **4a**. A substrate screening study revealed that a wide range of acyclic and cyclic α,β-unsaturated esters gave good-to-moderate yields of the reduced α-boryl ester products **2a–r** (Fig. 2). Generally, it was found that α,β-unsaturated esters with β-alkyl substituents gave good yields (*e.g.***2a**, **2b**, **2d** and **2e**). Increasing the substitution at the α or β position was viable (*e.g.***2f** and **2g**), with the formation of **2g** being notable as it contains a quaternary center and thus cannot be accessed *via* procedures starting from the α-diazo-ester. Cyclic esters were also amenable (**2h**, **2i**, and **2j**), with the reductive borylation of furanone proceeding with a yield of 61%, and **2i** successfully crystallized, with the solid-state structure from X-ray diffraction studies demonstrating the expected connectivity. The diastereoselectivity in the formation of **2j** was moderate (d.r. = 77 : 23). Cinnamates were also viable substrates and were borylated in moderate yields of 40–55% (**2k–2n**). Functionalisation at the *para*-position of the cinnamate phenyl ring with electron withdrawing groups and electron donating groups (–Cl, –Br, –NO_2_, and –Me) was possible with comparable yields (**2o–2r**). It should be noted that attempts with α,β-unsaturated ketones led to no significant α-borylated carbonyl products, in line with our calculations and previous work where C=O hydroboration occurs for similar compounds.^25^ Using the optimized conditions the reaction was amenable to scaling up to produce 0.9 g of **2a** and 1.0 g of **2k**.

**Scheme 4:**
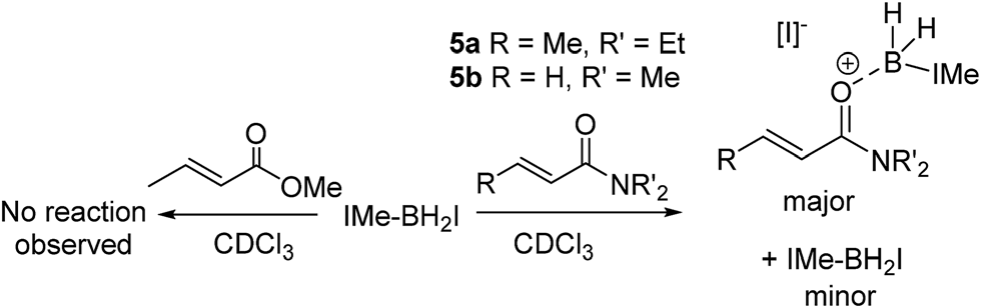
Reactivity disparity on combining α,β-unsaturated amides and esters with NHCBH_2_I.

Attempts to extend the optimized procedure to α,β-unsaturated amides led to complete amide consumption but with the formation of complex mixtures in which the desired α-boryl amide was only a minor intractable component (with *N*,*N*-diethyl-crotonamide, **5a**, and *N*,*N*-dimethylprop-2-enamide, **5b**). This is in contrast to reductive α-silylation which proceeds with both α,β unsaturated esters and amides (including **5a**).^17^ Compound **1b** which undergoes reductive borylation and **5b** have effectively identical *E* parameters on the Mayr electrophilicity scale (*E* = –23.59 and –23.54, respectively),^26^ and thus they would be expected to react at similar rates with unhindered nucleophiles, in contrast to what is observed here. Notably, while the *E* parameters are similar there is a significant difference in the nucleophilicity of esters and amides as Lewis bases towards boron electrophiles, with amides being significantly more Lewis basic than esters towards B(C_6_F_5_)_3_.^27^ Therefore the *in situ* NMR spectra were analyzed for any disparities between these esters and amides on addition to IMe–BH_2_I/IMe–BH_3_ mixtures. On combining a range of α,β-unsaturated esters/IMe–BH_3_/I_2_ (at various I_2_ loadings) in chloroform IMe–BH_2_I is observed (*δ*_11B_ –31 ppm) as the only new boron-containing species at short reaction times (at longer times the α-boryl esters are also observed with no intermediates detected). Thus these esters do not displace the iodide from boron in IMe–BH_2_I to any observable extent. In contrast, on combining IMe–BH_3_/I_2_ and **5a** or **5b** minimal IMe–BH_2_I is observed (even when using 50 mol% I_2_). Instead, a new broad ^11^B resonance (at –10.6 and –11.4 ppm when using **5a** and **5b**, respectively) is observed (Scheme 4). These products are assigned as the products from amide coordination to {IMe–BH_2_}^+^, with the *δ*_11B_ being consistent with [IMe–BH_2_(L)]^+^ boronium cations,^18^ and NHCBH_2_(OR) species.^28^ Furthermore, the NMR spectra obtained immediately after combining stoichiometric **5a** and IMe–BH_2_I revealed that the *δ*_11B_ –10.6 ppm species forms concomitantly with two new vinylic resonances in the ^1^H NMR spectrum indicating the persistence of the α,β-unsaturated moiety and the absence of any observable hydride transfer to form the O-boron enolate (which only has one vinylic proton). After longer reaction times (24 h) this stoichiometric reaction produced the α-boryl amide as only a minor product.

**Scheme 5:**
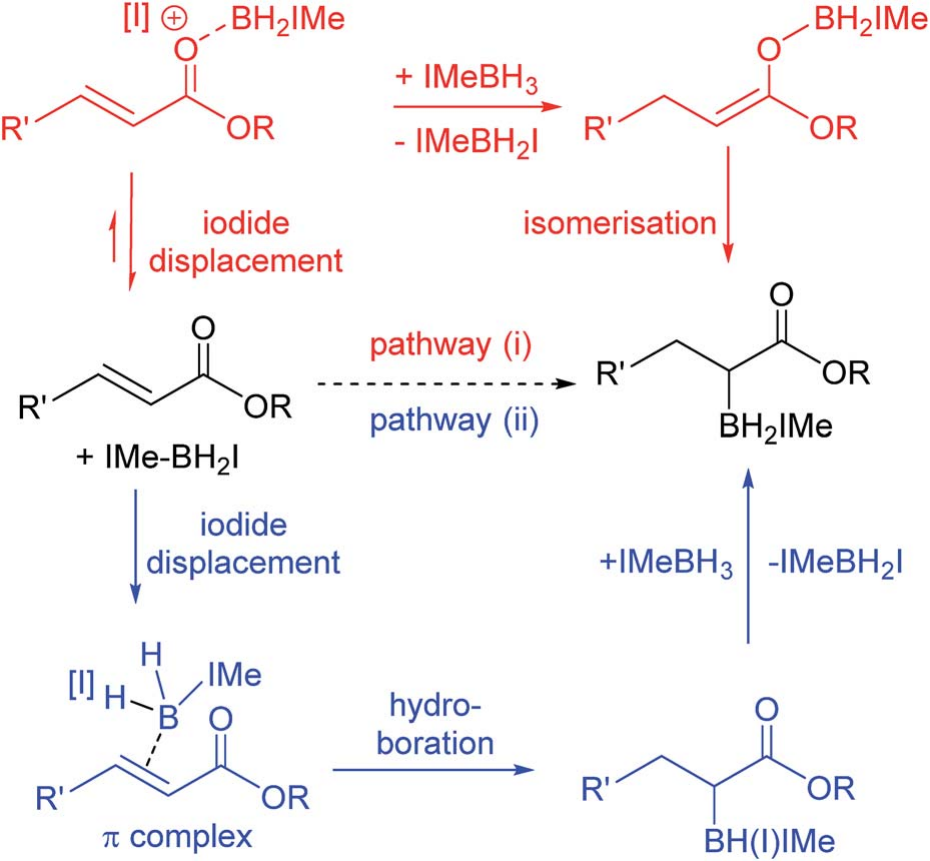
Pathways (i) and (ii) for reductive α-borylation of α,β-unsaturated esters.

The reactivity disparity between amides and esters suggested that coordination of carbonyl moieties to [IMe–BH_2_]^+^ does not lead to effective reductive α-borylation, at least for amide derivatives. This suggested that pathway (i) (Scheme 5, top) may not be operating for the reductive α-borylation of esters and that an alternative mechanism needs to be considered. Specifically, we considered a concerted addition of a B–H unit of IMe–BH_2_I across the double bond, prior to hydride transfer from another IMe–BH_3_ molecule (Scheme 5, bottom) as proposed for the hydroboration of simple alkenes.^21^,^22^ Mechanistic insight initially was sought *via* reductive borylation using a mixture of BenzIMe–BH_3_ and IMe–BD_3_; however, this was inconclusive as combining IMe–BD_3_ and BenzIMe–BH_3_ in CDCl_3_ solution with catalytic I_2_ led to rapid H/D exchange. Next the KIE was determined to be approximately 1.0 when using IMe–BD_3_ and IMe–BH_3_ in the reductive α-borylation of **1a** (initial KIE values ranged from 0.95 to 1.1). However, the absence of a significant KIE does not discriminate between either of the pathways, as a π complex (where the alkene has displaced the iodide) is potentially an intermediate in the hydroboration pathway using NHC–BH_2_I.^21^

**Scheme 6:**
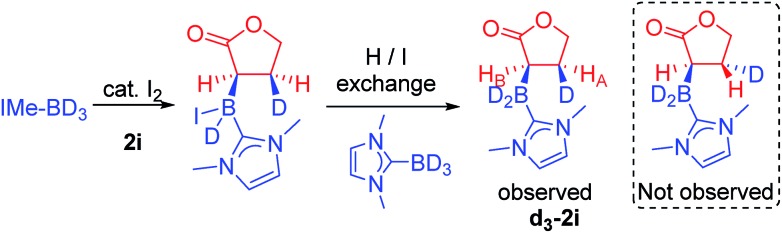
. Reductive borylation of **2i** with IMe–BD_3_.

Thus iodide displacement (by the carbonyl or by the alkene) is potentially the rate limiting step in both pathways. Finally, the reduction of cyclic ester **1i** with IMe–BD_3_/catalytic I_2_ was used to probe the stereoselectivity of reductive borylation, with a concerted hydroboration mechanism (pathway (ii)) leading to D and IMe–BD_2_ addition to the same face while pathway (i) is expected to give a mixture of isomers due to the lack of any facial discrimination during isomerization of the O-boron enolate to the α-boryl ester. The ^3^*J*_HH_ coupling constant between H_A_ and H_B_ (Scheme 6) for the d_3_-isotopomer of **2i** (**d_3_-2i**) allowed for assignment of a *syn*-configuration for the IMe–BD_2_ and D groups.^29^–^33^ Specifically, the ^3^*J*_HH_ coupling was 8.2 Hz, in good agreement with the 7.1 Hz (Karplus) and 9.7 Hz (Altona) values predicted for the *syn*-configuration of IMe–BD_2_ and D for **2i** (calculated based upon the solid-state structure of **2i**). The alternative isomer (see the inset in Scheme 6) had a calculated ^3^*J*_HH_ of <2 Hz and no **d_3_-2i** product containing this arrangement of D and IMe–BD_2_ was observed. Based upon these observations we conclude that α-borylation of this unsaturated ester using IMe–BH_3_/I_2_ occurs *via* a concerted addition mechanism (pathway (ii)).

**Scheme 7:**
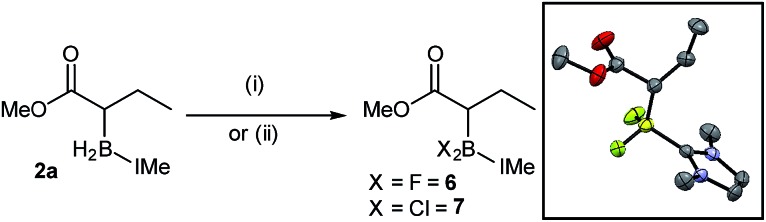
Formation of dihalo derivatives. (i) = selectfluor/MeCN and (ii) = NCS, toluene. Inset, solid state structure of **6**, ellipsoids at 50% probability (hydrogens omitted for clarity).

A series of reactions were carried out to functionalize these α-boryl esters. Curran *et al.* have shown that NHC–BH_2_-(*R*) can be converted to NHC–BX_2_(*R*) intermediates (X = halide) that are electrophilic at boron.^11^ Using the reported conditions IMe–BF_2_-ester, **6**, was synthesized (quantitatively by *in situ* NMR spectroscopy) and isolated in 46% yield after purification by column chromatography, with X-ray diffraction confirming the formulation (Scheme 7). The IMe–BCl_2_-derivative, **7**, was also accessible (again quantitatively by *in situ* NMR spectroscopy) through the reaction of **2a** with *N*-chloro-succinimide (NCS). Attempts to transform **2a** into the α-BPin-ester derivative by addition of NCS/pinacol under various conditions (with and without H_2_O/triethylamine, the latter as a base to sequester the expected HCl by-product) led instead to protodeboronation with no α-BPin congener observed. Furthermore, attempts to use **6** or **7** in a range of Suzuki–Miyaura cross coupling protocols were unsuccessful in our hands, including conditions reported by Chiu *et al.*^10^ for the cross-coupling of α-BPin esters, instead the protodeborylated product dominated. These observations are consistent with the sensitivity of non-quaternized α-boryl ester derivatives to rapid protodeboronation,^10^ in contrast to bench/column stable **2a–r**.

**Scheme 8:**
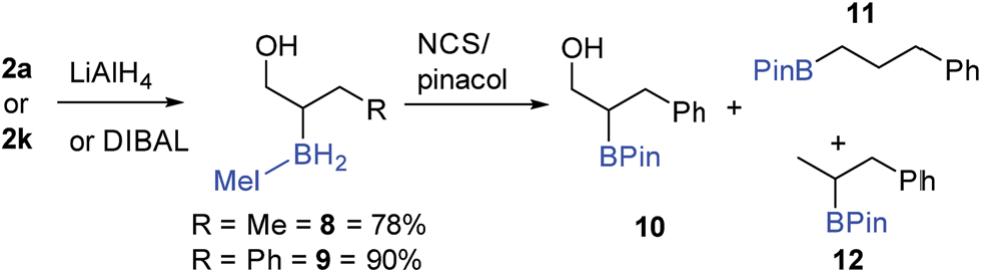
Reduction of α-boryl esters and subsequent reaction with NCS/pinacol.

Seeking transformations that cannot be achieved with α-BPin ester analogues, the reduction of **2a** to the alcohol was explored next with β-boryl alcohols previously reported and shown to be useful intermediates.^34^ Both LiAlH_4_ and DIBAL were used with **2a** to form the IMe–BH_2_-alcohol, **8** (Scheme 8), with no aldehyde observed under a range of conditions/reagent stoichiometry (lower equivalents of DIBAL instead led to a lower conversion to the alcohol and unreacted starting material). A phenyl analogue, **9**, can also be accessed, *e.g.* by addition of DIBAL to **2k**. It should be noted that in contrast to **2a** (or **2k**) organoBPin derivatives undergo transformation at boron with LiAlH_4_, to form [RBH_3_]^–^ species.^35^ Thus the successful conversion of **2a** (or **2k**) to **8** (or **9**) demonstrates the complementarity of the α-BPin and α-BH_2_NHC ester congeners.

The transformation of the BH_2_IMe group in **9** into BPin also was explored briefly. Compound **9** was combined with pinacol in dry toluene, and then NCS was added to form the reactive (towards ROH) –BCl_2_(NHC) congener *in situ*. ^11^B NMR spectroscopy displayed a major signal at 34 ppm consistent with an alkyl-BPin, but upon work-up the desired product **10** (Scheme 8) was only a minor component (31%). Two de-hydroxylated products, **11** and **12**, were produced alongside **10** (Scheme 8), in a combined yield of 51%. The formation of **11** and **12** is tentatively attributed to a Bora–Peterson reaction^36^ between the alcohol moiety and an electrophilic boryl group (*e.g.* RBCl(H)–IMe) derived from **9**/NCS which would on B–O bond formation eliminate the alkene (allylbenzene) and a reactive B–H species that can effect hydroboration of allylbenzene (the subsequent reaction with pinacol would then give **11** and **12**). Any acid initiated alcohol dehydration steps to form an alkene (for hydroboration) are disfavored due to comparable outcomes (formation of **10**, **11** and **12**) being observed in the presence and absence of amine bases (HCl is the expected by-product from addition of pinacol to B–Cl species). Due to the competitive formation of **10**, **11** and **12** and the greater step efficiency of using the NHC–BH_2_R species directly the direct functionalisation of the C–B moiety in **9** was explored. Compound **9** could be oxidized directly to 3-phenylpropane-1,2-diol using standard oxidation conditions for organoboranes (H_2_O_2_/NaOH) confirming the utility of these NHC-organoboranes and obviating the need for prior conversion to BPin (or other boron moieties).

**Fig. 3:**
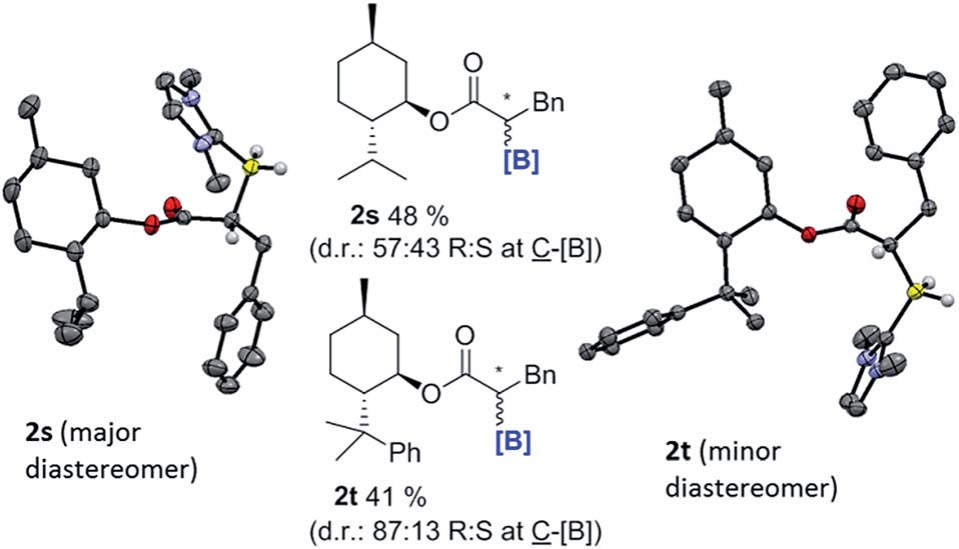
α-boryl esters containing chiral auxiliaries. Left, solid state structure of **2s** (the major diastereomer) and right, **2t** (the minor diastereomer), ellipsoids at 50% probability and most hydrogens omitted for clarity. Values shown are for isolated yields of the diastereomeric mixtures.

**Scheme 9:**
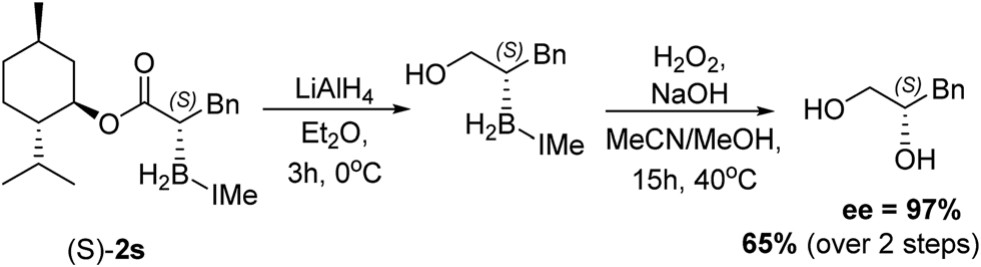
Reduction of a single diastereomer of **2s** and subsequent oxidation to form *S*-3-phenylpropane-1,2-diol.

With the feasibility of direct oxidation demonstrated this process was extended to enantioenriched products by performing reductive α-borylation on α,β-unsaturated esters containing chiral auxiliaries. The reductive α-borylation of methyl cinnamate with menthyl as the chiral auxiliary proceeded with minimal diastereoselectivity (**2s** d.r. = 57 : 43); however, the two products could be readily separated by column chromatography. Crystals of **2s** were grown enabling characterization by X-ray crystallography and unambiguous assignment of stereochemistry for both products of the reaction (Fig. 3). The more bulky 8-phenyl menthyl group was next utilized as a chiral auxiliary as it has been successfully used to induce high diastereoselectivity in Diels–Alder reactions using acrylates.^37^ In this case, two diastereomeric products of **2t** were isolated in a 87 : 13 d.r. after reductive α-borylation at room temperature (again these were readily separable by column chromatography). Confirmation of the major and minor components was achieved by X-ray diffraction. Finally, we investigated the possibility of transforming the enantiopure organoborane products into the chiral diol in two steps without isolation of any intermediates. A single diastereomer of **2s** was reduced to the alcohol using LiAlH_4_ and, after extraction, the crude reaction mixture was oxidized to yield *S*-3-phenylpropane-1,2-diol in an overall 65% yield, with no observable reduction in enantiopurity which was >97% (by chiral HPLC, Scheme 9).

## Conclusions

In conclusion, iodine activation of a bench stable borane, IMe–BH_3_, enables reductive α-borylation of a range of readily accessible (and in many cases commercially available) α,β-unsaturated esters. This procedure represents a simple route to bench stable quaternized (at B) α-boryl-esters that does not require transition metal catalysis. It is applicable to a wide range of ester substrates including cyclic, acyclic, and highly substituted derivatives and chiral precursors, the latter enable high diastereoselectivity in the reductive borylation step. Mechanistic studies reveal that concerted hydroboration proceeds in contrast to the mechanism for reductive α-silylation. The α-boryl ester products could be converted readily into the B–X (X = F and Cl) derivatives and β-boryl alcohols and glycols, including an example proceeding with high enantiopurity demonstrating the utility of this process and the products.

## Experimental

For general considerations see the ESI.†

### Synthesis of **2a**

A Schlenk flask was charged with IMe–BH_3_ (1.00 g, 9.1 mmol) and placed under an N_2_ atmosphere before CHCl_3_ (7.5 mL) was added. Methyl crotonate (0.80 mL, 7.6 mmol) was added to the flask and the resulting reaction solution was cooled to 0 °C. Iodine (192 mg, 0.76 mmol) was added to the mixture causing instant effervescence and the reaction was allowed to warm to room temperature. After being left to stir for 24 hours the solvent was removed under vacuum, yielding the crude product as an oily solid. The product was subsequently purified by column chromatography (SiO_2_, 9 : 1 ethyl acetate : pet. ether) to yield 897 mg of a white crystalline solid (56% yield). The identity and purity of the title compound was confirmed by ^1^H, ^13^C{^1^H} and ^11^B NMR spectroscopy. Crystals suitable for X-ray crystallography were successfully grown by slow evaporation of a chloroform solution confirming the molecular structure.

^1^H NMR (400 MHz, chloroform-d) *δ* 6.82 (s, 2H), 3.74 (d, *J* = 1 Hz, 6H), 3.44 (d, *J* = 1 Hz, 3H), 1.72 (m, 2H), 1.38 (t, *J* = 8 Hz, 1H), 0.87 (t, *J* = 7 Hz, 3H). ^13^C{^1^H} NMR (101 MHz, chloroform-d) *δ* 182.9, 120.4, 50.4, 41.6–38.7 (m), 36.1, 26.4, 15.1. ^11^B NMR (128 MHz, chloroform-d) *δ* –25.3 (t, *J* = 90 Hz).

### Synthesis of **d_3_-2i**

A Young's ampule was charged with IMe–BD_3_ (137 mg, 1.2 mmol) and placed under an N_2_ atmosphere. CHCl_3_ (1 mL) was added, followed by furanone (71 μL, 1 mmol) before the mixture was cooled to 0 °C. Iodine (25 mg, 0.1 mmol) was added causing rapid effervescence, after which the solution was allowed to warm to room temperature and left to stir for 24 hours. The solvent was then removed under vacuum to yield a crude product as an oily residue. The product was purified by column chromatography (SiO_2_, 95 : 5 DCM : MeOH) to yield the product (78 mg, 40% yield). The identity of the product was confirmed as that of the title compound by ^1^H, ^13^C and ^11^B NMR spectroscopy. Crystals of compound **d_3_-2i** suitable for X-ray diffraction studies were grown by slow evaporation from a chloroform solution, and the resulting structure was used as an input to determine predicted H–H coupling constants *via* the Karplus and Altona methods.

^1^H NMR (400 MHz, chloroform-d) *δ* 6.81 (d, *J* = 2.0 Hz, 2H), 4.46–4.35 (m, 1H), 4.28–4.15 (m, 1H), 3.70 (s, 6H), 2.31 (s, 1H), 1.77 (s, 1H). ^13^C{^1^H} NMR (101 MHz, chloroform-d) *δ* 187.9, 171.3–165.6 (m), 120.6, 67.6, 35.9, 33.5–28.2 (m).

^11^B NMR (128 MHz, chloroform-d) *δ* –27.4 (br). ^2^H NMR (61 MHz, chloroform-d) *δ* 1.87 (s, br), 1.76–1.15 (m).

### Synthesis of **2k**

A Schlenk flask was charged with IMe–BH_3_ (1.32 g, 12 mmol) and methyl cinnamate (1.62 g, 10 mmol) before placing under an N_2_ atmosphere. CHCl_3_ (10 mL) was added and the resulting solution was cooled to 0 °C. Iodine (506 mg, 2 mmol) was added causing instant effervescence and the reaction was allowed to warm to room temperature. The mixture was left to stir for 20 hours, after which time the solvent was removed under vacuum to yield a pale yellow oil as the crude product. The product was purified by column chromatography (SiO_2_, 2 : 1 ethyl acetate : pet. ether), yielding 1.01 g of a clear colourless oil (37% yield). The identity of the product was confirmed as the title compound by ^1^H and ^11^B NMR spectroscopic data matching with those previously reported in the literature.^8^

### Synthesis of **2s**

A Young's ampule was charged with menthyl cinnamate (286 mg, 1 mmol) and IMe–BH_3_ (132 mg, 1.2 mmol) before placing under an N_2_ atmosphere. CHCl_3_ (1 mL) was added and the resulting solution was cooled to 0 °C prior to addition of iodine (51 mg, 0.2 mmol). Effervescence was observed and the solution was allowed to warm to room temperature before being left to stir for 20 hours. The solvent was removed under vacuum giving an oily white residue which was purified by column chromatography (SiO_2_, ethyl acetate : pet. ether 1 : 1 → 2 : 1 varying polarity). Two diastereomeric products were isolated (total yield 191 mg, 48%; major: 108 mg, minor: 83 mg, 57 : 43 d.r.). The identity of the products as that of the title compounds was confirmed by ^1^H, ^13^C{^1^H} and ^11^B NMR spectroscopy. Crystals of the major diastereomer suitable for X-ray diffraction were successfully grown by evaporation of a DCM solution confirming the molecular structure of the compound, and allowing for the assignment of the stereochemistry at the C–B bond as the *R* configuration.

#### **2s** major diastereomer

^1^H NMR (400 MHz, chloroform-d) *δ* 7.24–7.14 (m, 4H), 7.12–7.03 (m, 1H), 6.79 (s, 2H), 4.37 (td, *J* = 11, 4 Hz, 1H), 3.76 (s, 6H), 3.06 (dd, *J* = 14, 11 Hz, 1H), 2.72 (dd, *J* = 14, 4 Hz, 1H), 2.22 (s, 1H), 1.61–1.26 (m, 6H), 1.10–0.98 (m, 1H), 0.97–0.84 (m, 1H), 0.78 (d, *J* = 6 Hz, 3H), 0.69 (d, *J* = 7 Hz, 3H), 0.51 (d, *J* = 7 Hz, 3H), 0.36 (q, *J* = 12 Hz, 1H).

^13^C{^1^H} NMR (101 MHz, chloroform-d) *δ* 181.2, 145.1, 128.7, 127.8, 125.0, 120.4, 71.3, 47.3, 41.2, 39.8, 39.7, 36.4, 34.5, 31.3, 25.7, 23.4, 22.3, 20.9, 16.3.

^11^B NMR (128 MHz, chloroform-d) *δ* –25.1 (t, *J* = 70 Hz).

#### **2s** minor diastereomer

^1^H NMR (400 MHz, chloroform-d) *δ* 7.21–7.16 (m, 4H), 7.10–7.03 (m, 1H), 6.78 (s, 2H), 4.45 (td, *J* = 11, 4 Hz, 1H), 3.75 (s, 6H), 3.08 (dd, *J* = 15, 10 Hz, 1H), 2.71 (dd, *J* = 15, 5 Hz, 1H), 2.25 (s, 1H), 1.74–1.65 (m, 1H), 1.59–1.52 (m, 2H), 1.47–1.31 (m, 2H), 1.25–1.15 (m, 1H), 0.99–0.86 (m, 1H), 0.81 (d, *J* = 7 Hz, 3H), 0.80–0.72 (m, 2H), 0.71 (d, *J* = 7 Hz, 3H), 0.54 (d, *J* = 7 Hz, 3H).

^13^C{^1^H} NMR (101 MHz, chloroform-d) *δ* 181.5, 144.6, 128.5, 127.9, 125.1, 120.5, 72.0, 47.1, 41.2, 39.6, 36.4, 34.5, 31.4, 25.2, 23.0, 22.2, 21.3, 15.9.

^11^B NMR (128 MHz, chloroform-d) *δ* –25.2 (t, *J* = 89 Hz).

### Synthesis of **2t**

A Young's ampule was charged with 8-phenyl-menthyl cinnamate (362 mg, 1 mmol) and IMe–BH_3_ (132 mg, 1.2 mmol) before placing under an N_2_ atmosphere. CHCl_3_ (1 mL) was added and the resulting solution was cooled to 0 °C prior to addition of iodine (51 mg, 0.2 mmol). Effervescence was observed and the solution was allowed to warm to room temperature before being left to stir for 20 hours. Subsequently, mesitylene (139 μL, 1 mmol) was added and an aliquot of the reaction solution was removed and subjected to NMR spectroscopic analysis to determine the degree of substrate consumption. This indicated a combined conversion of 72%. The sample was returned to the bulk solution before the solvent was removed under vacuum to yield the crude product mixture as an oily white residue. Two diastereomeric products were isolated by column chromatography (SiO_2_, ethyl acetate : pet. ether 1 : 1 → 2 : 1 varying polarity) as 167 mg and 26 mg (41% total yield, 87 : 13 d.r.). The identities of the products were confirmed by ^1^H, ^13^C{^1^H} and ^11^B NMR spectroscopy. Crystals of the minor diastereomer were successfully crystallised from slow cooling of a hot hexane solution confirming the structure of the product, and allowing for the assignment of the stereochemistry of the C–B bond as the *S* configuration for the minor component, confirming that the major component has the *R* configuration at the C–B.

#### **2t** major diastereomer

^1^H NMR (400 MHz, chloroform-d) *δ* 7.25–7.03 (m, 8H), 6.99–6.93 (m, 2H), 6.74 (s, 2H), 4.41 (td, *J* = 11, 4 Hz, 1H), 3.59 (s, 6H), 3.07 (dd, *J* = 14, 10 Hz, 1H), 2.61 (dd, *J* = 14, 4 Hz, 1H), 1.94–1.85 (m, 1H), 1.66–1.54 (m, 2H), 1.54–1.45 (m, 2H), 1.43–1.35 (m, 2H), 0.96 (d, *J* = 5 Hz, 6H), 0.93–0.77 (m, 2H), 0.73 (d, *J* = 7 Hz, 3H), 0.70–0.56 (m, 1H), 0.09 (q, *J* = 12 Hz, 1H).

^13^C{^1^H} NMR (101 MHz, chloroform-d) *δ* 180.9, 152.3, 145.3, 129.1, 127.9, 127.8, 125.9, 125.2, 124.7, 120.4, 72.7, 51.0, 41.6, 39.7, 39.6, 36.3, 34.9, 31.0, 27.1, 26.6, 26.0, 22.1.

^11^B NMR (128 MHz, chloroform-d) *δ* –25.0 (t, *J* = 90 Hz).

#### **2t** minor diastereomer

^1^H NMR (400 MHz, chloroform-d) *δ* 7.29–7.23 (m, 4H), 7.20–7.17 (m, 4H), 7.15–7.05 (m, 2H), 6.75 (s, 2H), 4.70 (td, *J* = 10.5, 4.3 Hz, 1H), 3.73 (s, 6H), 2.96 (dd, *J* = 13.9, 9.0 Hz, 1H), 2.62 (dd, *J* = 13.9, 6.0 Hz, 1H), 2.22–2.14 (m, 1H), 1.78 (ddd, *J* = 12.1, 10.4, 3.4 Hz, 1H), 1.68–1.60 (m, 1H), 1.43–1.35 (m, 2H), 1.28 (s, 3H), 1.20–1.17 (m, 3H), 1.08 (dq, *J* = 13.3, 3.3 Hz, 1H), 0.97–0.84 (m, 2H), 0.80 (dd, *J* = 12.6, 3.2 Hz, 1H), 0.74 (d, *J* = 6.4 Hz, 3H), 0.71–0.60 (m, 2H).

^13^C{^1^H} NMR (101 MHz, chloroform-d) *δ* 181.4, 151.2, 144.2, 128.9, 128.0, 127.8, 125.9, 125.2, 125.1, 120.6, 73.7, 50.6, 42.0, 40.7, 39.7, 36.3, 34.7, 31.3, 31.0, 27.7, 22.4, 21.9.

^11^B NMR (128 MHz, chloroform-d) *δ* –25.8 (t, *J* = 87 Hz).

### General catalytic substrate screening protocol

An oven dried J. Young's NMR tube was equipped with a *d*_6_-benzene filled capillary and charged with IMe–BH_3_ (0.6 mmol) before placing under an N_2_ atmosphere. Chloroform (0.5 mL) was added, followed by the desired α,β-unsaturated ester (0.5 mmol). The resulting starting material solution was analysed by ^1^H, ^11^B and ^11^B{^1^H} NMR spectroscopy to provide a comparison for reaction monitoring. Solid I_2_ (0.05 or 0.1 mmol) was added to the reaction mixture causing major effervescence in the tube, and ^1^H, ^11^B and ^11^B{^1^H} NMR spectra were recorded at *t* = 0. Subsequently, the reaction was set to mix for 20 hours, after which time further NMR spectroscopic analysis was undertaken. Mesitylene (0.5 mmol) was added to the sample, and ^1^H NMR spectroscopy allowed for the *in situ* reaction yield to be measured by integration of the product signals relative to mesitylene.

## Conflicts of interest

There are no conflicts to declare.

## Supplementary Material

Supplementary information

Supplementary information

Crystal structure data
