# Supplementary material for: Reductive α-borylation of α,β-unsaturated esters using NHC–BH3 activated by I2 as a metal-free route to α-boryl esters
Source: Chem Sci. 2018 Nov 19;10(5):1434–41. doi: 10.1039/c8sc04305a (PMC6354834; doi:10.1039/c8sc04305a)
Supplement: Supplementary file 2 [file SC-010-C8SC04305A-s002.pdf]

Electronic Supplementary Material (ESI) for Chemical Science.
This journal is © The Royal Society of Chemistry 2018

Supporting Information for:

Reductive α-borylation of α,β-unsaturated esters using NHC-BH_3 activated by I_2 as a metal free route to α-boryl esters.

James E. Radcliffe, Valerio Fasano, Ralph W. Adams, Peiran You, Michael J. Ingleson*
School of Chemistry, the University of Manchester, Manchester, M13 9PL, United Kingdom

michael.ingleson@manchester.ac.uk

1 General considerations 1
2 Synthesis of Starting Materials 2
3 Conditions screening 3
4 Initiator Screening 4
5 NHC-BH_3 screening 4
6 Larger scale synthesis of borylated esters: 5
7 Catalysis results 6
8 Mechanistic Studies 10
9 Reactivity of IMeBH_2-esters 26
10 Oxidation of IMe-BH_2-Phalcohol 35
11 Computational Details 38
12 X-ray crystallographic details 39
13 NMR Spectra of Novel Compounds 46
14 NMR Spectra of Catalysis runs 65
15 References 88

1 General considerations

Unless otherwise stated all procedures were carried out under inert conditions using standard Schlenk techniques (N_2 atmosphere) or in an MBraun Unilab glovebox (< 0.1 ppm H_2O / O_2, Ar atmosphere). Dichloromethane, toluene, tetrahydrofuran, pentane and diethyl ether were dried using a SPS prior to storing over 3 Å molecular sieves. *Protio*-chloroform, *deutero*-chloroform, *ortho*-dichlorobenzene and 1,2-dichloroethane were dried over CaH_2, distilled and stored over 3 Å molecular sieves. Unless otherwise stated all compounds were purchased from commercial sources and used as received without further purification. NMR spectra were recorded on Bruker AvanceIII-400 or Bruker Ascend-400 spectrometers. Chemical shifts are reported as dimensionless δ values and are frequency referenced relative to residual *protio*- impurities in the NMR solvents for ^{1}H and $^{13}C\{^{1}H\}$ respectively, while $^{11}B\{^{1}H\}$ and $^{19}F\{^{1}H\}$ shifts are referenced relative to external BF_3- Et_2O and hexafluorobenzene, respectively. Coupling constants *J* are given in Hertz (Hz) as positive values regardless of their real individual signs. The multiplicity of the signals are indicated as “s”, “d”, “t” “q” “pent”, “sept” or “m” for singlet, doublet, triplet, quartet, pentet, septet or multiplet, respectively. The ^{13}C resonance for carbons directly bonded to boron are not always observed due to signal broadening caused by quadrupolar relaxation.

Mass spectrometry was attempted for the novel compounds isolated in this study, but suitable conditions to ionise the borylated species could not be found. ASAP, ESI and CI techniques were all trialled in a range of solvents, none of which were found to yield molecular ions or identifiable fragments derived from these for identification. Moreover, CHN analysis on a range of isolated borylated products resulted in unsatisfactory data therefore purity is based on the observation of clean ^{1}H / ^{11}B NMR spectral data for isolated products that fully dissolved in organic solvents.

2 Synthesis of Starting Materials

2.1 1,3-Dimethylimidazol-2-ylidene borane (IMe-BH_3)

IMe-BH_3 was synthesised using a modified version of the preparation reported by Curran et al.[1] A 2-necked round bottom flask was charged with N-methyl imidazole before DCM was added. Iodomethane was subsequently added (1.2 eq.) to the solution dropwise and the reaction was left to stir at room temperature for 2 hours. After this time the solvent was removed under vacuum, yielding an oily residue to which toluene was then added, causing the imidazolium salt to precipitate out of solution as a white solid. $NaBH_4$ was added (1.5 eq.) to the suspension and the mixture was heated at reflux for 18 hours, after which time the product mixture was filtered while still hot. The reaction flask was washed with further hot toluene and the washings combined before the solvent was removed *in vacuo* to yield the crude product as a white solid. The product was purified by recrystallization from boiling water to yield the title compound as fine white needles. The identity and purity of the product were confirmed by ^1H and ^{11}B NMR spectroscopy, with the data matching those previously reported in the literature.[1]

IMe-BD_3 was synthesised in an identical manor, substituting $NaBH_4$ with $NaBD_4$.

2.2 1,3-Dimethylbenzimidazol-2-ylidene borane (BenzIMe-BH_3)

BenzIMe-BH_3 was synthesised following a modified version of the preparation reported by Walton, Lacôte, and Curran et al.[2] A flask was loaded with N-methylbenzimidazole, before DCM was added, followed by the dropwise addition of iodomethane. The solution was left to stir at room temperature overnight before the solvent was removed under vacuum to yield a beige solid. The solid was washed with small portions of diethyl ether before drying under vacuum and dissolving in THF. The resulting solution was cooled to –78 °C before KHMDS was added, followed an hour later by the dropwise addition of BH_3.THF. The reaction was allowed to warm to room temperature and left to stir for 16 hours, in which time a yellow solution formed along with a white precipitate. The solvent was removed under vacuum and the product was isolated by column chromatography (SiO_2, 4:1 pet. ether/ethyl acetate). Analysis by ^1H and ^{11}B NMR spectroscopy confirmed the identity and purity of the title product, with the data matching those reported in the literature.[2]

2.3 1,3-Diisopropylimidazol-2-ylidene borane (I^{i}Pr-BH_3)

I^{i}Pr-BH_3 was synthesised following a modified version of the preparation reported by Lacôte, and Curran et al.[1] A flask was loaded with N-isopropylimidazole before DCM was added, followed by the addition of isopropyl iodide (1.5 eq). The resulting solution was heated at reflux for 20 hours, after which time the solvent was removed under vacuum, yielding an oily residue. Diethyl ether was added and the mixture was stirred causing copious amounts of white precipitate to form. The diethyl ether was removed under vacuum before being replaced by toluene. $NaBH_4$ was added (0.75 eq.) and the mixture was set to reflux for 24 hours. After this time a further 0.75 eq $NaBH_4$ was added and the reaction was heated at reflux for another 24 hours. The toluene was removed under vacuum to yield an oily residue as the crude product. The product was purified by column chromatography (SiO_2, 2:1 pet. ether:ethyl acetate) and the identity of the title product was confirmed by ^1H and ^{11}B NMR spectroscopy, with the data matching those reported in the literature.[1]

2.4 **5-phenylfuran-2(5H)-one**

5-phenylfuran-2(5H)-one was synthesised following the preparation reported by Chang et al.[3] The identity of the product was confirmed by ^{1}H NMR spectroscopy with the data matching those reported in the literature.

2.5 **Menthyl cinnamate**

A flask was charged with cinnamoyl chloride and DCM prior to the addition of (1R,2S,5R)-(−)-menthol. The solution was cooled to 0 °C and triethylamine (2 eq.) was added. The solution was allowed to warm to room temperature and left to stir for 16 hours, after which time a large amount of white precipitate had formed. The solution was washed with 3 x HCl (1 M, aq.) before the organic fraction was dried over $MgSO_4$, filtered and the solvent removed under vacuum to yield the crude product as a yellow oil. The product was purified by column chromatography (SiO_2, 9:1 pet. ether:ethyl acetate) to yield the title compound as a clear oil. The identity and purity of the product were confirmed by ^{1}H and $^{13}C\{^{1}H\}$ NMR spectroscopic data matching those reported in the literature.[4]

2.6 **8-phenyl menthyl cinnamate**

A preparation reported by Muñiz et al. was followed. The identity of the product was confirmed by the ^{1}H NMR spectroscopic data matching those reported in the literature.[5]

3 Conditions screening

3.1 **Low concentration general procedure**

A Young's NMR tube was charged with IMe-BH_3 (16.5 mg, 0.15 mmol) and a C_6D_6 filled capillary before solvent (0.5 mL) was added. Methyl crotonate (10.6 µL, 0.1 mmol) was added to the solution and NMR spectroscopy confirmed that no reaction between the starting materials had occurred. Iodine (0.005 – 0.05 mmol) was then added to the mixture causing rapid effervescence before NMR spectroscopic analysis was used to confirm the composition of the reaction mixture at "t=0" (throughout t = 0 means approx. 5 minutes after addition of the last reagent). The reaction vessel was then set to mix at room temperature for 48 hours after which time the progress of the reaction was investigated by further NMR spectroscopy. Mesitylene (13.9 µL, 0.1 mmol) was added, before ^{1}H NMR spectroscopy was used to analyse the *in situ* reaction yield (measured by integration of product signals relative to mesitylene).

A range of solvents (DCM, DCE, *o*-DCB, o-DFB (o-DXB = ortho-dihalobenzene), toluene, $CHCl_3$), reaction temperatures (room temperature, 60 °C, 100 °C), and catalyst loadings (5 – 50 mol %) were screened. From these investigations 10 mol % I_2, $CHCl_3$, and 48 hours mixing at room temperature was found to yield the highest conversions of 57 % formation of **2a**, with 97 % consumption of methyl crotonate, indicative of some competitive reaction, presumably oligomerisation of methyl crotonate.

3.2 **High concentration procedure**

A Young's NMR tube was charged with IMe-BH_3 (66 mg, 0.6 mmol) and a C_6D_6 filled capillary before $CHCl_3$ (0.5 mL) was added. Methyl crotonate (53 µL, 0.5 mmol) was added to the solution. Iodine (12.7 mg, 0.05 mmol) was then added to the mixture causing rapid effervescence before NMR

spectroscopic analysis was used to confirm the composition of the reaction mixture at t=0. The reaction vessel was then set to mix at room temperature for 20 hours after which time the progress of the reaction was investigated by NMR spectroscopy. Mesitylene (69 µL, 0.5 mmol) was added, and ^{1}H NMR spectroscopy used to analyse the *in situ* reaction yield (measured by integration of product signals relative to mesitylene). There was measured to be 70 % formation of **2a**, with 95 % consumption of methyl crotonate.

4 Initiator Screening

4.1 Initiator = $B(C_6F_5)_3$

A Young's NMR tube was charged with IMe-BH_3 (66 mg, 0.6 mmol) and a C_6D_6 filled capillary before $CHCl_3$ (0.5 mL) was added. Methyl crotonate (53 µL, 0.5 mmol) was added to the solution. Tris(pentafluorophenyl)borane (25.6 mg, 0.05 mmol) was then added to the mixture causing some minor effervescence* before NMR spectroscopic analysis was used to confirm the composition of the reaction mixture at t=0. The reaction vessel was then set to mix at room temperature for 20 hours after which time the progress of the reaction was investigated by further NMR spectroscopy. Mesitylene (69 µL, 0.5 mmol) was added, and NMR spectroscopy used to analyse the *in situ* reaction yield (measured by integration of product signals relative to mesitylene in the ^{1}H spectrum). There was measured to be 16 % formation of **2a**, with 40 % consumption of methyl crotonate.

4.2 TBHP (tert-butyl hydrogen peroxide).

The conditions used were analogous to the recent report on radical mediated alkyne trans-hydroboration by Curran *et al*.[6]

A Young's NMR tube was charged with IMe-BH_3 (66 mg, 0.6 mmol) and C_6D_6 (0.5 mL) was added. Methyl crotonate (53 µL, 0.5 mmol) was added to the solution. *Tert*-butylhydroperoxide (18 µL, 0.1 mmol, 5.5 M in nonanes) was added prior to the reaction mixture being heated at 60 °C for 18 hours. The reaction was analysed by NMR spectroscopy, showing that no conversion to the reductive borylation product had occurred (see Fig. S75 and S76). Based upon this finding no further investigation using radical initiators was carried out.

5 NHC-BH_3 screening

5.1 BenzIMe-BH_3

A Young's NMR tube was charged with BenzIMe-BH_3 (96 mg, 0.6 mmol) and a C_6D_6 filled capillary before $CHCl_3$ (0.5 mL) was added. Methyl crotonate (53 µL, 0.5 mmol) was added to the solution. Iodine (12.7 mg, 0.05 mmol) was then added to the mixture causing rapid effervescence before NMR spectroscopic analysis was used to confirm the composition of the reaction mixture at t=0. The reaction vessel was then set to mix at room temperature for 20 hours after which time the progress of the reaction was investigated by further NMR spectroscopy. Mesitylene (69 µL, 0.5 mmol) was added, and ^{1}H NMR spectroscopy used to analyse the *in situ* reaction yield (measured by integration of product signals relative to mesitylene). There was measured to be 34 % formation of **Benz-IMe-BH_2-ester 4a**, with 50 % consumption of methyl crotonate.

* It is assumed that the minor effervescence arises from reaction of BCF with trace water present in the starting materials.

5.2 **I^{i}Pr-BH$_3$**

A Young's NMR tube was charged with IPr-BH$_3$ (100 mg, 0.6 mmol) and a C_6D_6 filled capillary before $CHCl_3$ (0.5 mL) was added. Methyl crotonate (53 µL, 0.5 mmol) was added to the solution and NMR spectroscopy confirmed that no reaction between the starting materials had occurred. Iodine (12.7 mg, 0.05 mmol) was then added to the mixture causing rapid effervescence before NMR spectroscopic analysis was used to confirm the composition of the reaction mixture at t=0. The reaction vessel was then set to mix at room temperature for 20 hours after which time the progress of the reaction was investigated by further NMR spectroscopy. Mesitylene (69 µL, 0.5 mmol) was added, and ^{1}H NMR spectroscopy used to analyse the *in situ* reaction yield (measured by integration of product signals relative to mesitylene). There was measured to be 43 % formation of **IPr-BH$_2$-ester 3a**, with 72 % consumption of methyl crotonate.

6 Larger scale synthesis of borylated esters:

6.1 Larger scale synthesis of IMe-BH$_2$-ester 2a

A Schlenk flask was charged with IMe-BH$_3$ (1.00 g, 9.1 mmol) and placed under an N_2 atmosphere before $CHCl_3$ (7.5 mL) was added. Methyl crotonate (0.80 mL, 7.6 mmol) was added to the flask and the resulting reaction solution was cooled to 0 °C. Iodine (192 mg, 0.76 mmol) was added to the mixture causing instant effervescence and the reaction was allowed to warm to room temperature. After being left to stir for 24 hours the solvent was removed under vacuum, yielding the crude product as an oily solid. The product was subsequently purified by column chromatography (SiO, 9:1 ethyl acetate:pet. ether) to yield 897 mg of a white crystalline solid (56 % yield). The identity and purity of the title compound was confirmed by ^{1}H, ^{13}C{^{1}H} and ^{11}B NMR spectroscopy. Crystals suitable for X-ray crystallography were successfully grown by slow evaporation from a chloroform solution confirming the molecular structure.

^{1}H NMR (400 MHz, Chloroform-d) δ 6.82 (s, 2H), 3.74 (d, J = 1 Hz, 6H), 3.44 (d, J = 1 Hz, 3H), 1.72 (m, 2H), 1.38 (t, J = 8 Hz, 1H), 0.87 (t, J = 7 Hz, 3H).

^{13}C{^{1}H} NMR (101 MHz, Chloroform-d) δ 182.9, 120.4, 50.4, 41.6 – 38.7 (m), 36.1, 26.4, 15.1.

^{11}B NMR (128 MHz, Chloroform-d) δ -25.3 (t, J = 90 Hz).

6.2 Large scale synthesis of IMe-BH$_2$-Phester 2k

A Schlenk flask was charged with IMe-BH$_3$ (1.32 g, 12 mmol) and methyl cinnamate (1.62 g, 10 mmol) before placing under an N_2 atmosphere. $CHCl_3$ (10 mL) was added and the resulting solution was cooled to 0 °C. Iodine (506 mg, 2 mmol) was added causing instant effervescence and the reaction was allowed to warm to room temperature. The mixture was left to stir for 20 hours, after which time the solvent was removed under vacuum to yield a pale yellow oil as the crude product. The product was purified by column chromatography (SiO_2, 2:1 ethyl acetate:pet. ether), yielding 1.01 g of a clear colourless oil (37 % yield). The identity of the product was confirmed as the title compound by ^{1}H and ^{11}B NMR spectroscopic data matching with those previously reported in the literature.[7]

7 Catalysis results

7.1 General Catalytic Substrate Screening Protocol

An oven dried J. Young's NMR tube was equipped with a d_6-benzene filled capillary and charged with IMe-BH_3 (0.6 mmol) before placing under an N_2 atmosphere. Chloroform (0.5 mL) was added, followed by the desired α,β-unsaturated ester (0.5 mmol). The resulting starting material solution was analysed by ^{1}H, ^{11}B and $^{11}B\{^{1}H\}$ NMR spectroscopy to provide a comparison for reaction monitoring. Solid I_2 (0.05 or 0.1 mmol) was added to the reaction mixture causing major effervescence in the tube, and ^{1}H, ^{11}B and $^{11}B\{^{1}H\}$ NMR spectra were recorded at t=0. Subsequently, the reaction was set to mix for 20 hours, after which time further NMR spectroscopic analysis was undertaken. Mesitylene (0.5 mmol) was added to the sample, and ^{1}H NMR spectroscopy allowed for the *in situ* reaction yield to be measured by integration of the product signals relative to mesitylene.

7.2 **Table S1: full catalysis results**

Substrate	Catalyst	Catalyst loading	Ester consumption	Product	Yield (isolated)
Methyl crotonate (1a)	I_2	10%	95%	**2a**	70% (56%)
Ethyl crotonate (1b)	I_2	10%	89%	**2b**	71%
tert-butyl crotonate (1c)	I_2	10%	67%	**2c**	42%
Methyl pentenoate (1d)	I_2	10%	94%	**2d**	73%
Methyl cinnamate (1k)	I_2	20%	89%	**2k**	53% (37%)
Ethyl cinnamate (1l)	I_2	20%	85%	**2l**	52%
Furanone (1i)	I_2	10%	100%	**2i**	61% (40%)
iso-Propyl cinnamate (1m)	I_2	10%	80%	**2m**	52%
Benzyl cinnamate (1n)	I_2	10%	79%	**2n**	40%
Dihydropyranone (1h)	I_2	10%	100%	**2h**	40%
4-Nitrocinnamate (1q)	I_2	20%	80%	**2q**	55%
4-Chlorocinnamate (1o)	I_2	20%	82%	**2o**	55%
Methyl tiglate (1g)	I_2	20%	60%	**2g**	45%
Methyl dimethylacrylate (1f)	I_2	20%	80%	**2f**	37%
4-Bromocinnamate (1p)	I_2	20%	88%	**2p**	50%
Methyl-4-methylpentenoate (1e)	I_2	20%	92%	**2e**	73%
Ph-lactone (1j)	I_2	10%	96%	**2j**	40%
Methyl crotonate (1a)	I_2	10%	50%	**2a**	34%
Methyl crotonate (1a)	$B(C_6F_5)_3$	10%	40%	**2a**	16%
Methyl crotonate (1a)	I_2	10%	72%	**2a**	43%
Para-methyl methyl cinnamate (1r)	I_2	20%	88%	**2r**	51%

7.3 Synthesis of IMe-BH_2-menthyl-ester 2s

A Young's ampule was charged with menthyl cinnamate (286 mg, 1 mmol) and IMe-BH_3 (132 mg, 1.2 mmol) before placing under an N_2 atmosphere. $CHCl_3$ (1 mL) was added and the resulting solution was cooled to 0 °C prior to addition of iodine (51 mg, 0.2 mmol). Effervescence was observed and the solution was allowed to warm to room temperature before being left to stir for 20 hours. The solvent was removed under vacuum giving an oily white residue which was purified by column chromatography (SiO_2, ethyl acetate:pet. ether 1:1 → 2:1 varying polarity). Two diastereomeric products were isolated (total yield 191 mg, 48 %. Major: 108 mg, Minor: 83 mg, 57:43 d.r.). The identity of the products as that of the title compounds was confirmed by ^{1}H, $^{13}C\{^{1}H\}$ and ^{11}B NMR spectroscopy. Crystals of the major diastereomer suitable for X-ray diffraction were successfully grown by evaporation of a DCM solution confirming the molecular structure of the compound, and allowing for the assignment of the stereochemistry at the C-B bond as the R configuration.

7.3.1 Compound 2s major diastereomer

^{1}H NMR (400 MHz, Chloroform-*d*) δ 7.24 – 7.14 (m, 4H), 7.12 – 7.03 (m, 1H), 6.79 (s, 2H), 4.37 (td, *J* = 11, 4 Hz, 1H), 3.76 (s, 6H), 3.06 (dd, *J* = 14, 11 Hz, 1H), 2.72 (dd, *J* = 14, 4 Hz, 1H), 2.22 (s, 1H), 1.61 – 1.26 (m, 6H), 1.10 – 0.98 (m, 1H), 0.97 – 0.84 (m, 1H), 0.78 (d, *J* = 6 Hz, 3H), 0.69 (d, *J* = 7 Hz, 3H), 0.51 (d, *J* = 7 Hz, 3H), 0.36 (q, *J* = 12 Hz, 1H).

^{13}C{^{1}H} NMR (101 MHz, Chloroform-*d*) δ 181.2, 145.1, 128.7, 127.8, 125.0, 120.4, 71.3, 47.3, 41.2, 39.8, 39.7, 36.4, 34.5, 31.3, 25.7, 23.4, 22.3, 20.9, 16.3.

^{11}B NMR (128 MHz, Chloroform-*d*) δ -25.1 (t, *J* = 70 Hz).

7.3.2 Compound 2s minor diastereomer

^{1}H NMR (400 MHz, Chloroform-d) δ 7.21 – 7.16 (m, 4H), 7.10 – 7.03 (m, 1H), 6.78 (s, 2H), 4.45 (td, J = 11, 4 Hz, 1H), 3.75 (s, 6H), 3.08 (dd, J = 15, 10 Hz, 1H), 2.71 (dd, J = 15, 5 Hz, 1H), 2.25 (s, 1H), 1.74 – 1.65 (m, 1H), 1.59 – 1.52 (m, 2H), 1.47 – 1.31 (m, 2H), 1.25 – 1.15 (m, 1H), 0.99 – 0.86 (m, 1H), 0.81 (d, J = 7 Hz, 3H), 0.80 – 0.72 (m, 2H), 0.71 (d, J = 7 Hz, 3H), 0.54 (d, J = 7 Hz, 3H).

^{13}C{^{1}H} NMR (101 MHz, Chloroform-d) δ 181.5, 144.6, 128.5, 127.9, 125.1, 120.5, 72.0, 47.1, 41.2, 39.6, 36.4, 34.5, 31.4, 25.2, 23.0, 22.2, 21.3, 15.9.

^{11}B NMR (128 MHz, Chloroform-d) δ -25.2 (t, J = 89 Hz).

7.4 Synthesis of IMe-BH_2-8-phenyl-menthyl-ester 2t

A Young's ampule was charged with 8-phenyl-menthyl cinnamate (362 mg, 1 mmol) and IMe-BH_3 (132 mg, 1.2 mmol) before placing under an N_2 atmosphere. $CHCl_3$ (1 mL) was added and the resulting solution was cooled to 0 °C prior to addition of iodine (51 mg, 0.2 mmol). Effervescence was observed and the solution was allowed to warm to room temperature before being left to stir for 20 hours. Subsequently, mesitylene (139 µL, 1 mmol) was added and an aliquot of the reaction was removed and subjected to NMR spectroscopic analysis to determine the degree of substrate consumption. This indicated a combined conversion of 72 %. The sample was returned to the bulk solution before the solvent was removed under vacuum to yield the crude product mixture as an oily white residue. Two diastereomeric products were isolated by column chromatography (SiO_2, ethyl acetate:pet. ether 1:1 → 2:1 varying polarity) as 167 mg and 26 mg (41 % total yield, 87:13 d.r.). The

identities of the products were confirmed by ^{1}H, ^{13}C{^{1}H} and ^{11}B NMR spectroscopy. Crystals of the minor diastereomer were successfully crystallised from slow cooling of a hot hexane solution confirming the structure of the product, and allowing for the assignment of the stereochemistry of the C-B bond as the S configuration for the minor component, confirming the major is the R configuration at the C-B

7.4.1 Compound 2t major diastereomer

^{1}H NMR (400 MHz, Chloroform-d) δ 7.25 – 7.03 (m, 8H), 6.99 – 6.93 (m, 2H), 6.74 (s, 2H), 4.41 (td, J = 11, 4 Hz, 1H), 3.59 (s, 6H), 3.07 (dd, J = 14, 10 Hz, 1H), 2.61 (dd, J = 14, 4 Hz, 1H), 1.94 – 1.85 (m, 1H), 1.66 – 1.54 (m, 2H), 1.54 – 1.45 (m, 2H), 1.43 – 1.35 (m, 2H), 0.96 (d, J = 5 Hz, 6H), 0.93 – 0.77 (m, 2H), 0.73 (d, J = 7 Hz, 3H), 0.70 – 0.56 (m, 1H), 0.09 (q, J = 12 Hz, 1H).

^{13}C{^{1}H} NMR (101 MHz, Chloroform-d) δ 180.9, 152.3, 145.3, 129.1, 127.9, 127.8, 125.9, 125.2, 124.7, 120.4, 72.7, 51.0, 41.6, 39.7, 39.6, 36.3, 34.9, 31.0, 27.1, 26.6, 26.0, 22.1.

^{11}B NMR (128 MHz, Chloroform-d) δ -25.0 (t, J = 90 Hz).

7.4.2 Compound 2t minor diastereomer

^{1}H NMR (400 MHz, Chloroform-d) δ 7.29 – 7.23 (m, 4H), 7.20 – 7.17 (m, 4H), 7.15 – 7.05 (m, 2H), 6.75 (s, 2H), 4.70 (td, J = 10.5, 4.3 Hz, 1H), 3.73 (s, 6H), 2.96 (dd, J = 13.9, 9.0 Hz, 1H), 2.62 (dd, J = 13.9, 6.0 Hz, 1H), 2.22 – 2.14 (m, 1H), 1.78 (ddd, J = 12.1, 10.4, 3.4 Hz, 1H), 1.68 – 1.60 (m, 1H), 1.43 – 1.35 (m, 2H), 1.28 (s, 3H), 1.20 – 1.17 (m, 3H), 1.08 (dq, J = 13.3, 3.3 Hz, 1H), 0.97 – 0.84 (m, 2H), 0.80 (dd, J = 12.6, 3.2 Hz, 1H), 0.74 (d, J = 6.4 Hz, 3H), 0.71 – 0.60 (m, 2H).

^{13}C{^{1}H} NMR (101 MHz, Chloroform-d) δ 181.4, 151.2, 144.2, 128.9, 128.0, 127.8, 125.9, 125.2, 125.1, 120.6, 73.7, 50.6, 42.0, 40.7, 39.7, 36.3, 34.7, 31.3, 31.0, 27.7, 22.4, 21.9.

^{11}B NMR (128 MHz, Chloroform-*d*) δ -25.8 (t, *J* = 87 Hz).

8 Mechanistic Studies

8.1 **N,N-dimethyl acrylamide + IMe-BH$_3$ + I$_2$** (using ester optimised conditions) – note the broad resonance in the ^{11}B NMR spectra at -10 ppm (see later stoichiometric reactions).

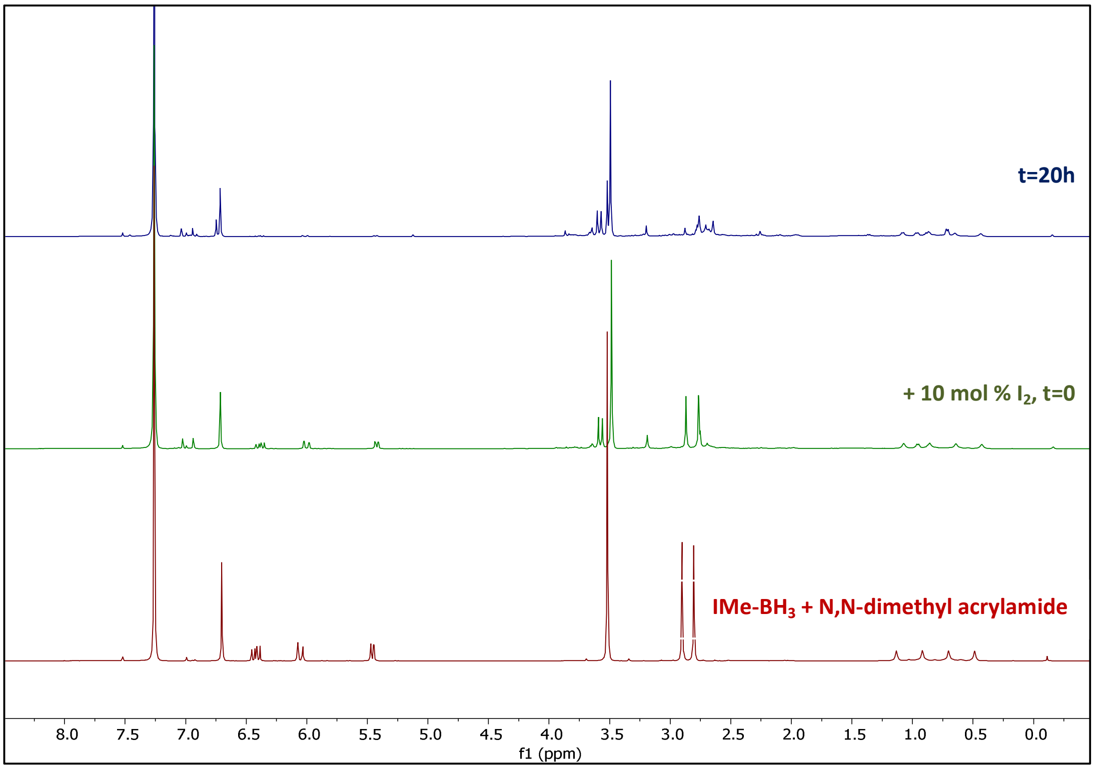

Figure S1: Collected ^{1}H NMR spectra (CHCl$_3$, 400 MHz, 298K with capillary insert containing C$_6$D$_6$) of: IMe-BH$_3$ and N,N-dimethyl acrylamide (bottom); the reaction mixture ~5 minutes after the addition of I$_2$ (middle); the reaction mixture 20 hours after the addition of I$_2$, with 1 eq. mesitylene as a standard (top).

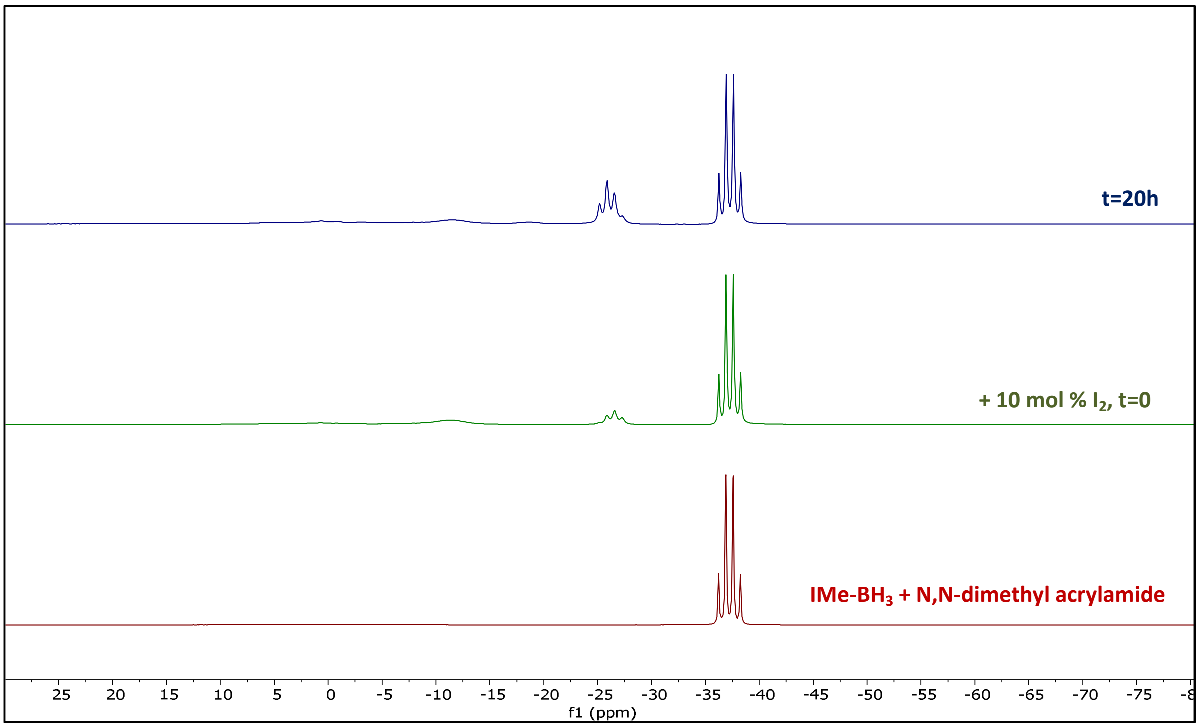

Figure S2: Collected ^{11}B NMR spectra (CHCl$_3$, 128.4 MHz, 298K) of: IMe-BH$_3$ and N,N-dimethyl acrylamide (bottom); the reaction mixture ~5 minutes after the addition of I$_2$ (middle); the reaction mixture after 20 hours after the addition of I$_2$, with 1 eq. mesitylene as a standard (top).

8.2 Intermediacy of NHC-BH$_2$I and the absence of any observable ester-BH$_2$(NHC) adduct

The *in-situ* NMR spectra of IMe-BH$_3$ / I$_2$ / methyl crotonate provided the following ^{11}B NMR spectra at short reaction times (as stated above t=0 is approx. 5 minutes). It is apparent that a mixture of IMe-BH$_3$ and IMe-BH$_2$I has formed and persists on addition of the ester (effectively identical spectra are observed at t=0 for all the esters studied). This indicates that iodide is more coordinating towards boron in these compounds than these esters.

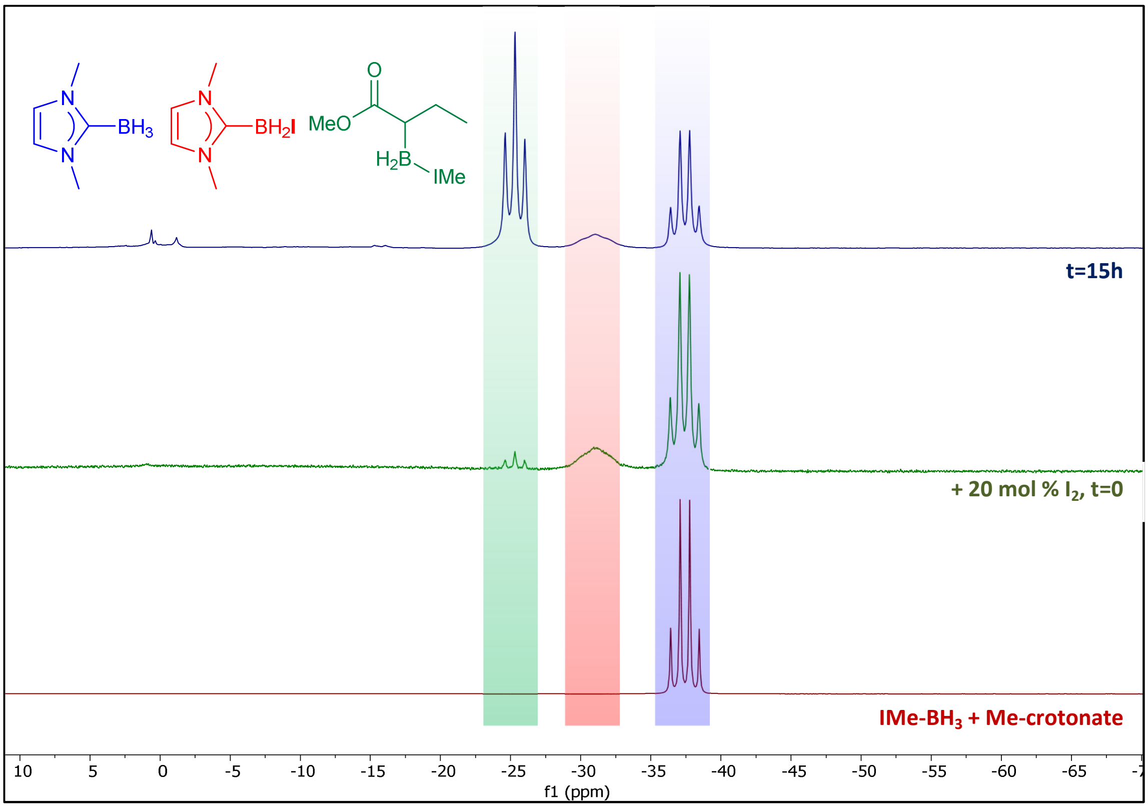

Figure S3: Collected ^{11}B NMR spectra (CHCl$_3$, 128.4 MHz, 298K) of: IMe-BH$_3$ and Me-crotonate (bottom); the reaction mixture ~5 minutes after the addition of I$_2$ (middle); the reaction mixture after mixing at room temperature for 15 hours (top).

8.3 N,N-diethyl crotonamide + IMe-BH_3 reaction

Notably, on addition of N,N-Et_2-crotonamide to mixtures of NHCBH_3/NHCBH_2I the iodide is displaced from boron and a new ^{11}B resonance is observed. On mixing for 20 hours only a trace borylated amide product is observed by ^{11}B NMR spectroscopy.[7,8]

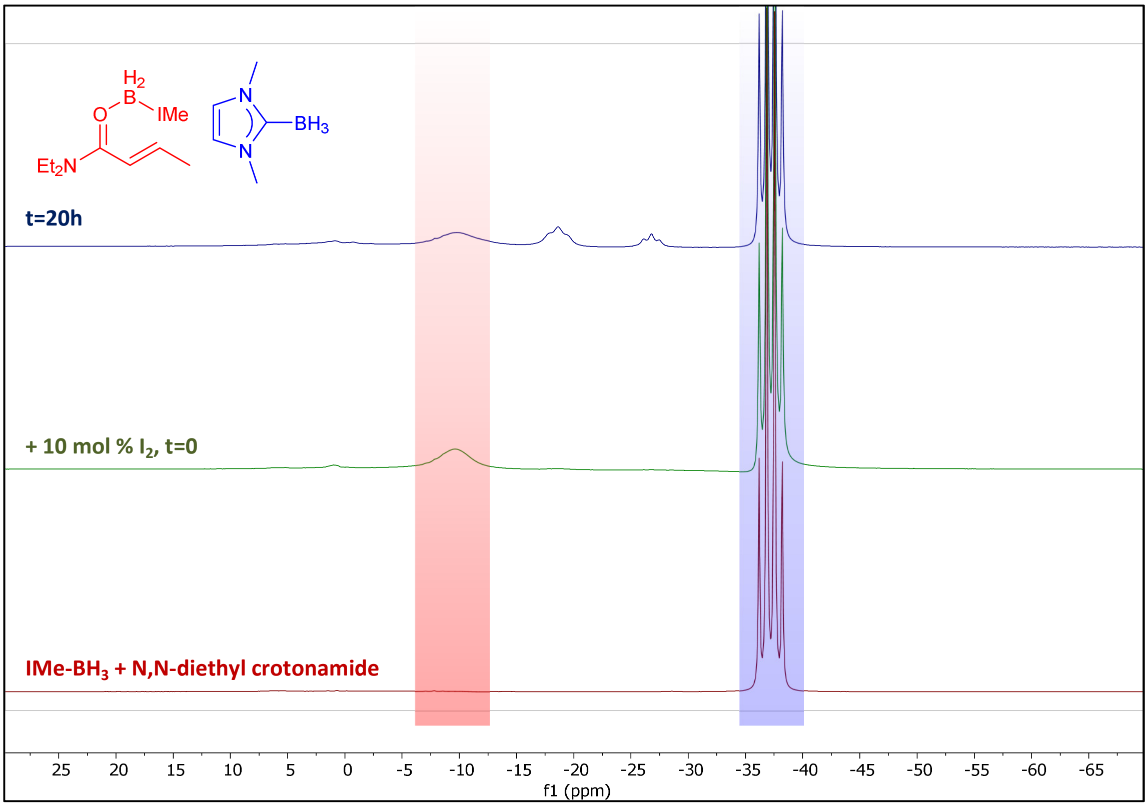

Figure S4: Collected ^{11}B NMR spectra ($CHCl_3$, 128.4 MHz, 298K) of: IMe-BH_3 and N,N-diethyl crotonamide (bottom); the reaction mixture ~5 minutes after the addition of I_2 (middle); the reaction mixture after mixing at room temperature for 20 hours (top).

Due to the formation of a new compound at ca. -10 ppm in the ^{11}B NMR spectra with both amides the stoichiometric reaction between NHCBH_2I and N,N-Et_2-crotonamide was performed to probe the identity further. The spectra are shown below. This clearly shows conversion to a new product as indicated by the major signal at -9.8 ppm in the ^{11}B NMR spectrum. The persistence of the two vinylic protons (that are correlated in the COSY) indicates that the boron enolate is not formed, but instead it is the adduct between the amide and IMe-BH_2. Attempts to isolate this compound or obtain mass spec were not successful in our hands. Furthermore, attempts to improve the conversion to the reductive a borylated product using different quantities of I_2 or IMeBH_3 failed to give any significantly improved results.

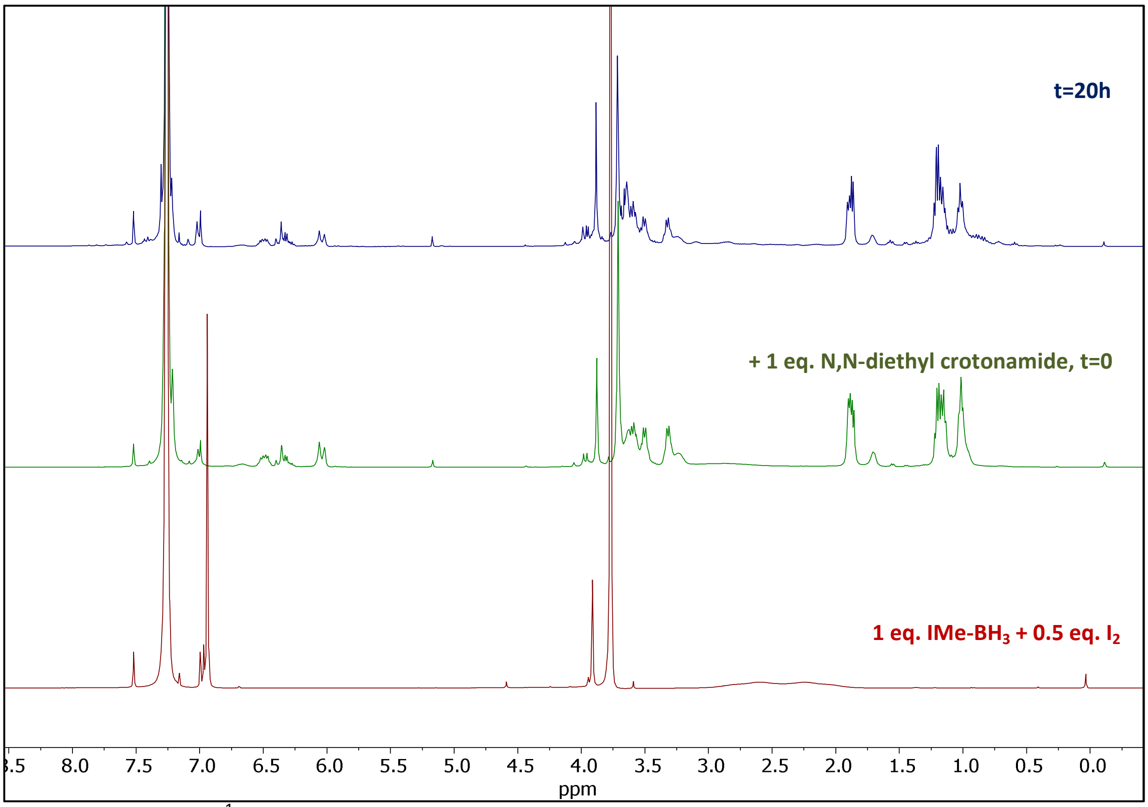

Figure S5: Collected ^{1}H NMR spectra ($CHCl_3$, 400 MHz, 298K with capillary insert containing C_6D_6) of: IMe-BH$_2$I and IMe-BH$_3$ (bottom); the reaction mixture ~5 minutes after the addition of N,N-diethyl crotonamide (middle); the reaction mixture after mixing at room temperature for 20 hours (top).

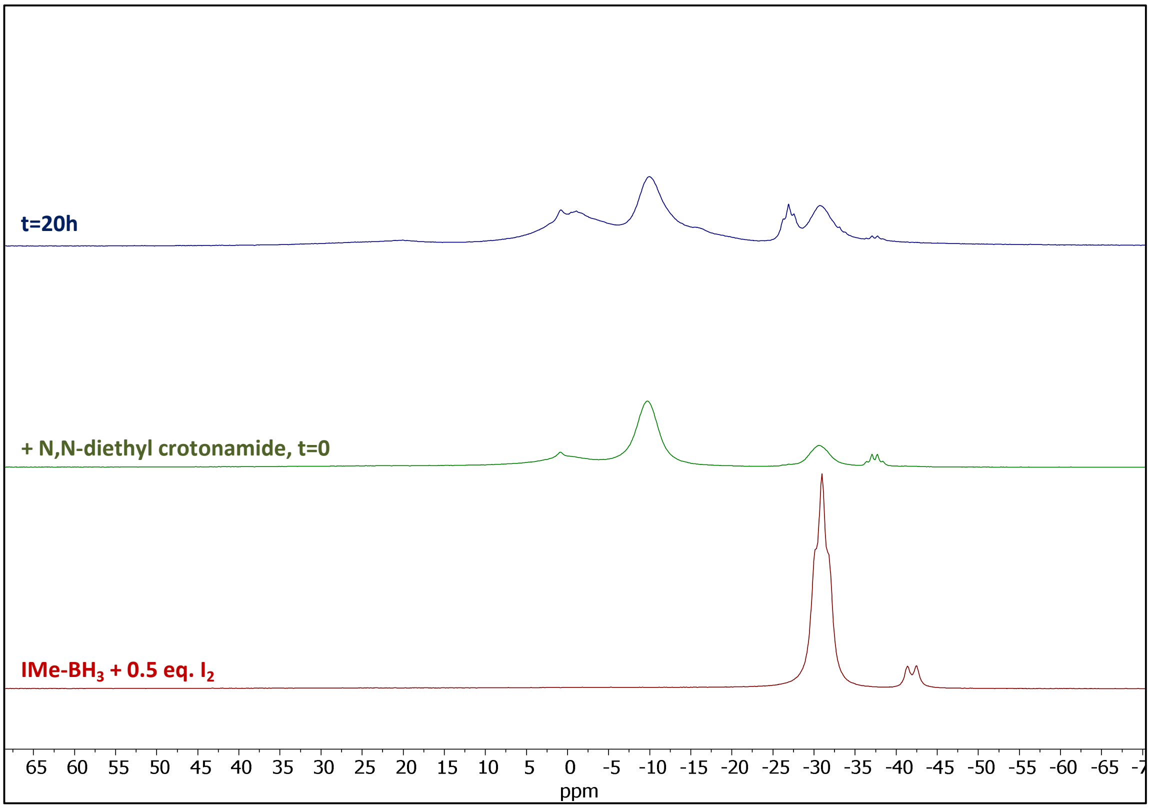

Figure S6: Collected ^{11}B NMR spectra ($CHCl_3$, 128.4 MHz, 298K) of: IMe-BH$_2$I and IMe-BH$_3$ (bottom); the reaction mixture ~5 minutes after the addition of N,N-diethyl crotonamide (middle); the reaction mixture after mixing at room temperature for 20 hours (top).

8.4 IMe-BH$_3$ / IMe-BD$_3$ comparison with Me-crotonate

Two identical reactions were set up following the general catalysis protocol, one containing IMe-BH$_3$ and the other IMe-BD$_3$ for comparison. Both reactions were analysed by NMR spectroscopy at a number of time points in order to observe the progress of the reaction. Both reactions were found to be effectively identical (for ratio of product : starting NHC-BH$_3$ complex), hence the kinetic isotope effect (KIE) is close to 1, indicting B-H cleavage is not occurring in the rate limiting step.

8.4.1 IMe-BH$_3$ + Me-crotonate + I$_2$

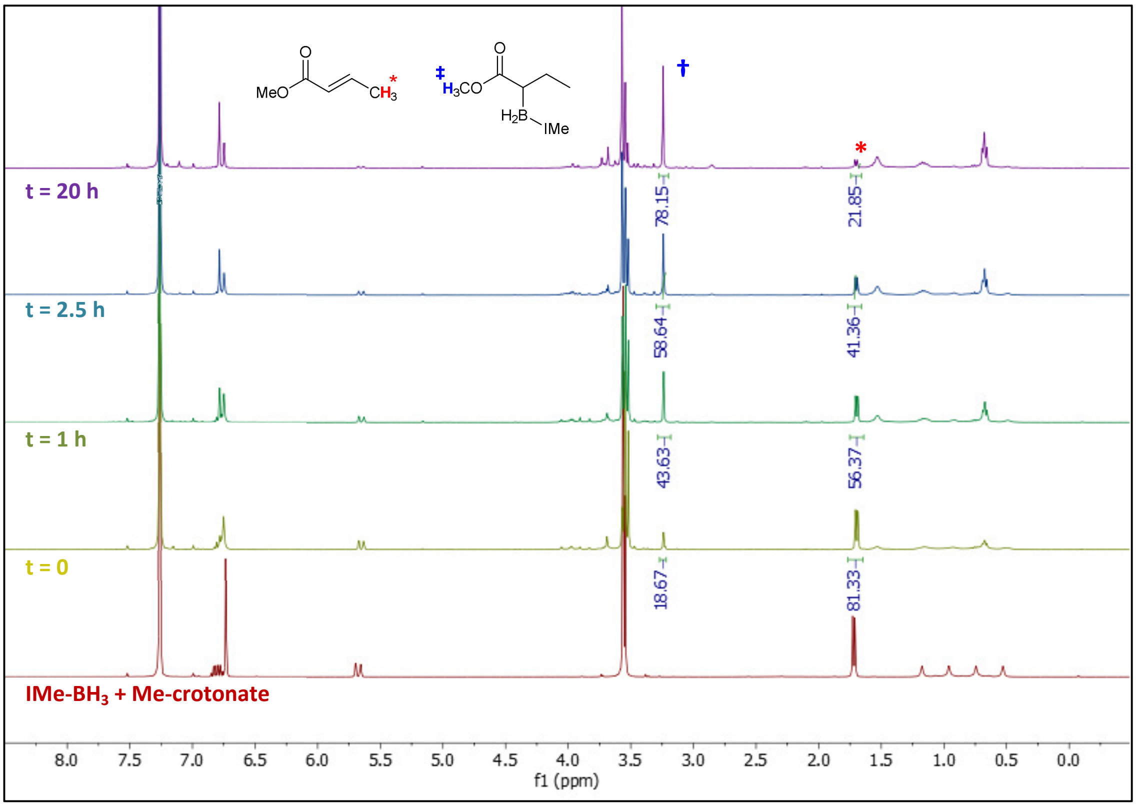

Figure S7: Collected ^{1}H NMR spectra ($CHCl_3$, 400 MHz, 298K with capillary insert containing C_6D_6) of: IMe-BH$_3$ and methyl crotonate (bottom); the reaction mixture after the addition of I$_2$ at increasing time intervals, labelled on the spectra.

8.4.2 *IMe-BD$_3$ + Me-crotonate + I$_2$*

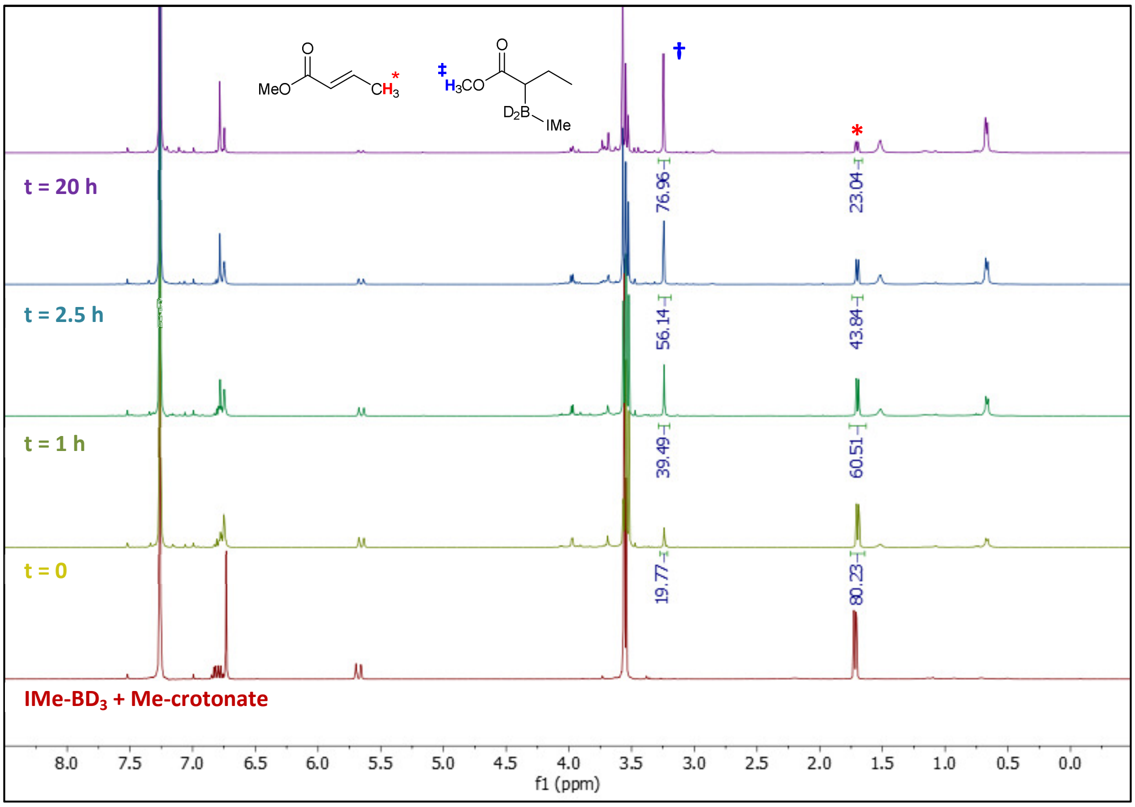

Figure S8: Collected ^{11}B NMR spectra (CHCl$_3$, 128.4 MHz, 298K) of: IMe-BD$_3$ and methyl crotonate (bottom); the reaction mixture after the addition of I$_2$ at increasing time intervals, labelled on the spectra.

8.5 **IMe-BD$_3$ / BenzIMe-BH$_3$ competition reaction**

To determine if any intermolecular steps were involved in the reductive alpha-borylation mechanism, the feasibility of a crossover reaction was tested. However, due to rapid H/D scrambling no definitive evidence could be extracted.

A Young's ampule was charged with IMe-BD$_3$ (136 mg, 1.2 mmol) and BenzIMe-BH$_3$ (192 mg, 1.2 mmol) and placed under an N$_2$ atmosphere. CHCl$_3$ (2 mL) was added, followed by methyl crotonate (211 µL, 2 mmol). The reaction mixture was cooled to 0 °C prior to addition of iodine (50 mg, 0.2 mmol). The reaction was allowed to warm to room temperature and left to stir for 48 hours. The solvent was subsequently removed under vacuum to yield the crude product mixture as an oily residue. The products were purified by column chromatography (SiO$_2$, pet ether:ethyl acetate, vary from 3:2 to 1:4 respectively), yielding IMe-BH/D-ester (114 mg, 27 % yield relative to Me-crotonate) and BenzIMe-BH/D-ester (97 mg, 19 % yield relative to Me-crotonate). Extensive H/D scrambling was observed with both compounds displaying almost identical ^{11}B and ^{2}D NMR spectra containing multiple isotopomers. The identity of **IMe-BH/D-ester 2a** was confirmed by ^{1}H NMR spectroscopic data matching those previously measured (see section 6.1 for the characterisation). The identity of BenzIMe-BH/D-ester **4a** was confirmed by ^{1}H, $^{13}C\{^{1}H\}$ and ^{11}B NMR spectroscopy. Attempts at mass spectrometry yielded no detection of the product. Crystals of compound **4a** were successfully grown by slow evaporation of a chloroform solution confirming the molecular structure.

^{1}H NMR (400 MHz, Chloroform-*d*) δ 7.39 (ddt, *J* = 17.5, 6.3, 3.4 Hz, 4H), 3.91 (s, 6H), 3.35 (s, 3H), 1.89 – 1.68 (m, 2H), 1.50 – 1.35 (m, 1H), 0.87 (t, *J* = 7.0 Hz, 3H).

^{13}C NMR (101 MHz, Chloroform-*d*) δ 182.4, 179.5 – 176.6 (m), 133.0, 124.2, 110.9, 50.3, 40.8 – 38.9 (m), 32.3, 26.4 (d, *J* = 2 Hz), 14.9 (d, *J* = 11 Hz).

^{11}B NMR (128 MHz, Chloroform-*d*) δ -23.8 – -26.4 (m).

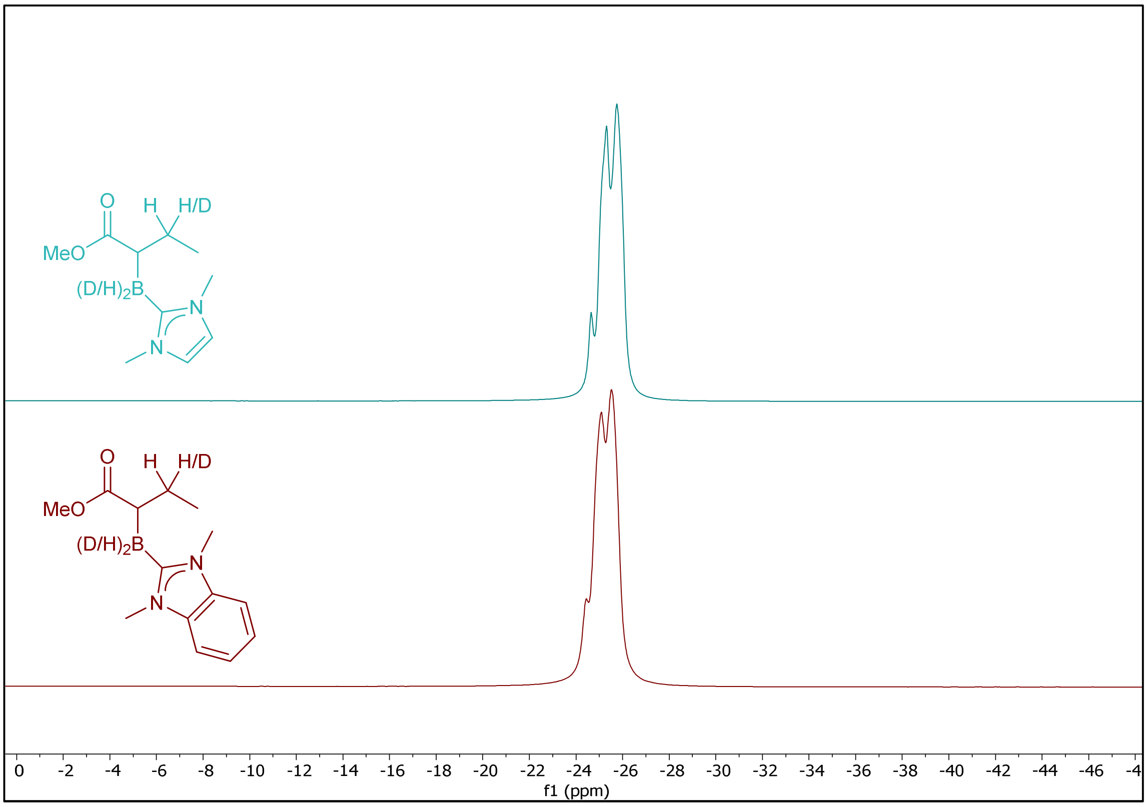

Figure S9: Collected ^{11}B NMR spectra ($CHCl_3$, 128.4 MHz, 298K) of: d_n-BenzIMe-B(H/D)$_2$-ester **4a**(bottom); d_n-IMe-B(H/D)$_2$-ester **2a** (top).

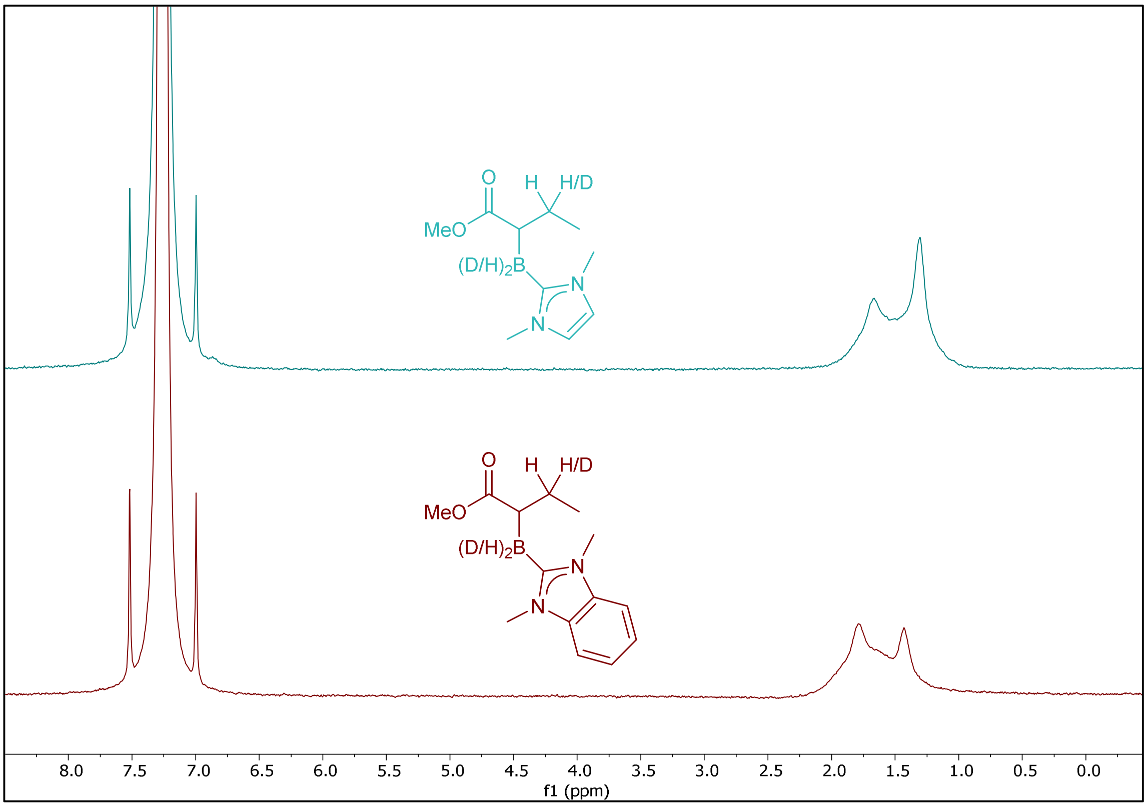

Figure S10: Collected ^{2}D NMR spectra ($CHCl_3$, 62 MHz, 298K) of: d_n-BenzIMe-B(H/D)$_2$-ester **4a** (bottom); d_n-IMe-B(H/D)$_2$-ester **2a** (top).

8.6 IMe-BD$_2$-lactone synthesis (d$_3$-2i)

A Young's ampule was charged with IMe-BD$_3$ (137 mg, 1.2 mmol) and placed under an N_2 atmosphere. $CHCl_3$ (1 mL) was added, followed by furanone (71 µL, 1 mmol) before the mixture was cooled to 0 °C. Iodine (25 mg, 0.1 mmol) was added causing rapid effervescence, after which the solution was allowed to warm to room temperature and left to stir for 24 hours. The solvent was then removed under vacuum to yield a crude product as an oily residue. The product was purified by column chromatography (SiO_2, 95:5 DCM:MeOH) to yield the product (78 mg, 40 % yield). The identity of the product was confirmed as that of the title compound by ^{1}H, ^{13}C and ^{11}B NMR spectroscopy. Crystals of compound **d$_3$-2i** suitable for X-ray diffraction studies were grown by slow evaporation from a chloroform solution, and the resulting structure was used as an input to determine predicted H-H coupling constants *via* the Karplus and Altona methods (see below).

^{1}H NMR (400 MHz, Chloroform-*d*) δ 6.81 (d, *J* = 2.0 Hz, 2H), 4.46 – 4.35 (m, 1H), 4.28 – 4.15 (m, 1H), 3.70 (s, 6H), 2.31 (s, 1H), 1.77 (s, 1H).

^{13}C{^{1}H} NMR (101 MHz, Chloroform-*d*) δ 187.9, 171.3 – 165.6 (m), 120.6, 67.6, 35.9, 33.5 – 28.2 (m).

^{11}B NMR (128 MHz, Chloroform-*d*) δ -27.4 (br)

^{2}H NMR (61 MHz, Chloroform-*d*) δ 1.87 (s, br), 1.76 – 1.15 (m).

8.7 Assignment of stereochemistry of d_3-2i by measuring $^3J_{HH}$ coupling constant

The stereochemistry of **d_3-2i** was determined by a comparison of measured and calculated $^3J_{HH}$.

The energy of the 3D structure of the protio-analogue of **d_3-2i**, shown in Figure S11, was minimized from several starting positions using the MM2 minimization function of Chem3D (Version 16.0.1.4, PerkinElmer Informatics, Inc.), using default parameters, until a minimum steric energy was achieved. The resulting 3D structure was exported in MOL format then opened using MSpin (Version 2.3.2-694, MestReLab Research S. L.). The JCoupling module in MSpin was used to calculate 3JHH using the Karplus equation.[9] Equivalent J_{HD} values were calculated using the relationship $J_{HD} = \gamma D\ J_{HH} / \gamma H$ where γD and γH are the gyromagnetic ratios of deuterium and protium respectively and are given in Table S2. MSpin was also used to calculate $^3J_{HH}$ using the 'Altona' method of Haasnoot, de Leeuw and Altona[10] which includes a correction for electronegativity of the substituents. The atom coordinates from the structure as determined by X ray crystallography were also passed in mol format into the JCoupling module in MSpin and values for J_{HH} calculated using Karplus and Altona methods. These values for J_{HH}, along with the equivalent J_{HD}, are given in Table S3.

The majority of signals in the ^{1}H NMR spectrum of **d_3-2i** that contain coupling constants informative for stereochemistry determination are broadened significantly due to interactions with both ^{2}H and $^{11/10}$ B. To overcome the broad signals and extract coupling constants we implemented an ^{11}B-decoupled variant of the PSYCHE-2DJ experiment.[11] It was hoped that this would allow measurement of both the $^3J_{12}$ and $^3J_{13}$ coupling constants. However, J_{HD} – which was calculated to have a magnitude of either 0.3 Hz or 1.1 Hz – was not observed and a comparison of the calculated values with only the measured value for J_{HH} was used to determine the stereochemistry.

For the energy minimized structure, the couplings calculated using the Karplus equation, when H2 is deuterium and H3 is protium, were $^3J_{12}$ = 0.3 Hz and $^3J_{13}$ = 7.1 Hz. Using the Altona method $^3J_{12}$ = 0.2 Hz and $^3J_{13}$ = 9.7 Hz. When H2 is protium and H3 is deuterium, the Karplus equations yielded $^3J_{12}$ = 1.8 Hz and $^3J_{13}$ = 1.1 Hz and the Altona method gave $^3J_{12}$ = 1.1 Hz and $^3J_{13}$ = 1.5 Hz. A slight discrepancy in the values of the calculated and measured values (measured $^3J_{13}$ = 8.2 Hz) for $^3J_{HH}$ arises due to poor parameterisation of the electronegativity of the BH_2- substituent in the Altona model implemented in MSpin. The Altona method applied to the structure determined by X ray crystallography gives values slightly closer to the measured value of JHH than when applied to the structure determined by computational energy minimisation. However, the Karplus method gave slightly worse agreement with experiment for the structure determined by X ray crystallography.

The relative magnitude of the calculated $^3J_{HH}$ using both the Karplus equation and the Altona method, for both the calculated and X ray crystal structures, is consistent with the structure where H2 is deuterium and H3 is protium. The agreement between the calculated and measured $^3J_{13}$ values for **d_3-2i** established the stereochemistry to be R at the B-substituted position (3) and S at the D-substituted position (4) of the furanone.

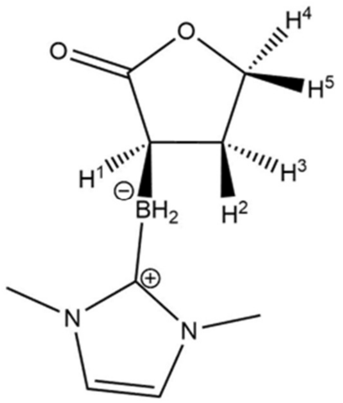

Figure S11. Chemical structure of the protio-analogue of X used for calculation of J_{HH} with numbering.

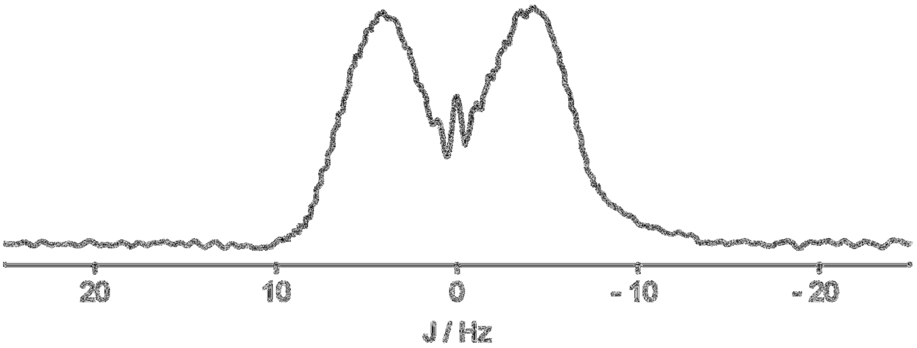

Figure S12. F1 projection of the H1 signal of 1 (at 1.71 ppm) in the 45° tilted 11B-decoupled variant of the PSYCHE-2DJ spectrum used to measure the coupling. The signal is a doublet with 8.2 Hz coupling.

Coupling	Calculated J_{HH} / Hz		Equivalent J_{HD} / Hz		Measured / Hz
	Karplus	Altona	Karplus	Altona	
$^3J_{12}$	1.8	1.1	0.3	0.2	Not observed
$^3J_{13}$	7.1	9.7	1.1	1.5	8.2

Table S2. Calculated and measured values of the coupling constants for the compound in figure S11 relevant for assignment of the stereochemistry, based on the calculated structure.

Coupling	Calculated J_{HH} / Hz		Equivalent J_{HD} / Hz		Measured / Hz
	Karplus	Altona	Karplus	Altona	
$^3J_{12}$	1.6	0.8	0.2	0.1	Not observed
$^3J_{13}$	6.6	8.9	1.0	1.4	8.2

Table S3. Calculated and measured values of the coupling constants for the compound in figure S11 relevant for assignment of the stereochemistry, based on the structure determined from X ray crystallography.

Example Chem3D output file:

```
MM2 Calculation completed successfully
------------MM2 Minimization------------
Pi System:   1   2   3   4
Warning: Some parameters are guessed (Quality = 1).
Iteration    1  Steric Energy   11.205  RMS Gradient    0.009  RMS Move   0.0000
Iteration    2  Steric Energy   11.205  RMS Gradient    0.008  RMS Move   0.0000
Iteration    2: Minimization terminated normally because the gradient norm is less than the minimum gradient norm
Stretch:                 0.4638
Bend:              17.6916
Stretch-Bend:    -0.1244
Torsion:                 -2.8854
Non-1,4 VDW:   -3.5749
1,4 VDW:          6.3120
Charge/Charge:           0.0000
Charge/Dipole:  -11.9506
Dipole/Dipole:   5.2727
Total Energy:     11.2048 kcal/mol
Calculation ended
------------------------------------------
```

Mol file from MM2 Calculation, used for JHH calculation in MSpin:

```
Chem3D Core 16.006071811073D

 29 30  0  0  0  0  0  0  0  0999 V2000
   -2.1180   -1.0147    1.0972 N   0  0  0  0  0  0  0  0  0  0  0  0
   -2.7922   -0.3462    1.9404 C   0  0  0  0  0  0  0  0  0  0  0  0
   -2.8555    0.9322    1.5427 C   0  0  0  0  0  0  0  0  0  0  0  0
   -2.2235    1.0616    0.4494 N   0  0  0  0  0  0  0  0  0  0  0  0
   -1.6575   -0.1948   -0.0102 C   0  3  0  0  0  0  0  0  0  0  0  0
    0.7357   -1.2647   -0.5989 C   0  0  0  0  0  0  0  0  0  0  0  0
    2.2424   -1.0181   -0.6705 C   0  0  0  0  0  0  0  0  0  0  0  0
    0.3088   -1.2998   -2.0421 C   0  0  0  0  0  0  0  0  0  0  0  0
   -0.8064   -1.6122   -2.3853 O   0  0  0  0  0  0  0  0  0  0  0  0
    1.3326   -0.9003   -2.8324 O   0  0  0  0  0  0  0  0  0  0  0  0
    2.4240   -0.4540   -2.0728 C   0  0  0  0  0  0  0  0  0  0  0  0
   -2.1913    2.3750   -0.2179 C   0  0  0  0  0  0  0  0  0  0  0  0
   -1.9597   -2.4677    1.2838 C   0  0  0  0  0  0  0  0  0  0  0  0
   -0.0648   -0.0700    0.0815 B   0  5  0  0  0  0  0  0  0  0  0  0
   -3.2485   -0.7907    2.8324 H   0  0  0  0  0  0  0  0  0  0  0  0
   -3.3722    1.7624    2.0380 H   0  0  0  0  0  0  0  0  0  0  0  0
    0.4994   -2.2308   -0.0998 H   0  0  0  0  0  0  0  0  0  0  0  0
    2.7877   -1.9881   -0.5799 H   0  0  0  0  0  0  0  0  0  0  0  0
    2.6164   -0.3329    0.1227 H   0  0  0  0  0  0  0  0  0  0  0  0
    2.4016    0.6606   -2.0656 H   0  0  0  0  0  0  0  0  0  0  0  0
    3.3722   -0.7900   -2.5501 H   0  0  0  0  0  0  0  0  0  0  0  0
   -3.1292    2.9529   -0.0555 H   0  0  0  0  0  0  0  0  0  0  0  0
```

```
  -1.3300    2.9727    0.1576 H   0  0  0  0  0  0  0  0  0  0  0  0
  -2.0714    2.2358   -1.3163 H   0  0  0  0  0  0  0  0  0  0  0  0
  -2.8014   -2.9183    1.8565 H   0  0  0  0  0  0  0  0  0  0  0  0
  -1.9276   -2.9727    0.2919 H   0  0  0  0  0  0  0  0  0  0  0  0
  -1.0089   -2.6795    1.8233 H   0  0  0  0  0  0  0  0  0  0  0  0
   0.3132    1.0844   -0.5171 H   0  0  0  0  0  0  0  0  0  0  0  0
   0.3067    0.0109    1.3816 H   0  0  0  0  0  0  0  0  0  0  0  0
  1  2  1  0
  5  1  1  0
  1 13  1  0
  2  3  2  0
  2 15  1  0
  3  4  1  0
  3 16  1  0
  5  4  1  0
  4 12  1  0
  5 14  1  0
  6  7  1  0
  8  6  1  0
 14  6  1  0
  6 17  1  0
  7 11  1  0
  7 18  1  0
  7 19  1  0
  8  9  2  0
  8 10  1  0
 11 10  1  0
 11 20  1  0
 11 21  1  0
 12 22  1  0
 12 23  1  0
 12 24  1  0
 13 25  1  0
 13 26  1  0
 13 27  1  0
 14 28  1  0
 14 29  1  0
M  CHG  2   5   1  14  -1
M  END
```

psychejresph1g pulse sequence code used for acquiring decoupled phase sensitive 2D J-resolved spectra with absorption mode line shapes:

```
;psychejresphig

;PSYCHE 2D J-resolved
;with decoupling during acquisition
;phase sensitive 2D J-resolved with absorption mode lineshapes
;using PSYCHE as J-refocusing element
;to acqure N-type and J-reversed N-type ser files
;Using echo / anti-echo processing
;should be prosol friendly - uses non-standard pulse p41, p42 and p43 which are not modified by default
;
;R. Adams and M. Foroozandeh
;University of Manchester
;Avance II+/III Version
;Topspin 3.x
;
;(1)  Foroozandeh, M.; Adams, R. W.; Meharry, N. J.; Jeannerat, D.; Nilsson, M.; Morris, G. A. Angew.
Chem. Int. Ed. 2014, 53, 6990.
;(2)  Foroozandeh, M.; Adams, R. W.; Nilsson, M.; Morris, G. A. J. Am. Chem. Soc. 2014, 136, 11867.
;
;$CLASS=HighRes
;$DIM=2D
;$TYPE=
;$SUBTYPE=
;$COMMENT=

#include <Avance.incl>
#include <Delay.incl>
#include <Grad.incl>

"d0=0u"
"in0=inf1/4"
"d12=20u"
"l0=1"

"cnst50=(cnst20/360)*sqrt((2*cnst21)/(p43/2000000))"
"p30=1000000.0/(cnst50*4)"
"cnst31= (p30/p1) * (p30/p1)"
"spw43=plw1/cnst31"

"p31=1000000.0/(cnst51*4)"
"cnst32= (p31/p1) * (p31/p1)"
"spw41=plw1/cnst32"
"spw42=spw41"
```

```
"p20=p43"
"p21=p41"
"p22=p42"

"d11=30m"

1 ze
2 d1 do:f2
  d11 pl2:f2
3 d12 pl1:f1

  p1 ph1

  if "l0 %2 == 1"
    {
    50u UNBLKGRAD
    p16:gp1
    d16
    d16 pl0:f1
    ( center (p41:sp41 ph2):f1 (p21:gp11) )
    d16
    p16:gp1
    d16
    50u
    50u
    p16:gp2
    d16
    d16
    ( center (p43:sp43 ph3):f1 (p20:gp10) )
    d16
    p16:gp2
    d16
    50u
    d0
(p4 ph5):f2;
    d0
    50u
    p16:gp3
    d16
    d16
    ( center (p42:sp42 ph4):f1 (p22:gp12) )
    d16
    p16:gp3
    d16
    50u BLKGRAD
    d0
(p4 ph5):f2;
```

```
    d0
    }
  else
    {
    d0
(p4 ph5):f2;
    d0
    50u UNBLKGRAD
    p16:gp3
    d16
    d16
    ( center (p42:sp42 ph4):f1 (p22:gp12) )
    d16
    p16:gp3
    d16
    50u
    d0
(p4 ph5):f2;
    d0
    50u
    p16:gp2
    d16
    d16
    ( center (p43:sp43 ph3):f1 (p20:gp10) )
    d16
    p16:gp2
    d16
    50u
    50u
    p16:gp1
    d16
    d16
    ( center (p41:sp41 ph2):f1 (p21:gp11) )
    d16
    p16:gp1
    d16
    50u BLKGRAD
    }
  3u pl12:f2
  go=2 ph31 cpd2:f2
  d1 do:f2 mc #0 to 2 F1EA(iu0, id0)
exit

ph1= 0 2 0 2 0 2 0 2 0 2 0 2 0 2 0 2 0 2 0 2 0 2 0 2 0 2 0 2 0 2 0 2 0 2 0 2 0 2 0 2 0 2 0 2 0 2 0 2 0 2 0 2 0 2 0
2 0 2 0 2 0 2 0 2
ph2= 0 0 0 0 0 0 0 0 1 1 1 1 1 1 1 1 0 0 0 0 0 0 0 0 1 1 1 1 1 1 1 1 0 0 0 0 0 0 0 0 1 1 1 1 1 1 1 1 0 0 0 0 0 0 0
0 1 1 1 1 1 1 1 1
```

ph3= 0 0 0 0 1 1 1 1 0 0 0 0 1 1 1 1 2 2 2 2 3 3 3 3 2 2 2 2 3 3 3 3 0 0 0 0 1 1 1 1 0 0 0 0 1 1 1 1 2 2 2 2 3 3 3
3 2 2 2 2 3 3 3 3
ph4= 0 0 1 1 0 0 1 1 0 0 1 1 0 0 1 1 0 0 1 1 0 0 1 1 0 0 1 1 0 0 1 1 2 2 3 3 2 2 3 3 2 2 3 3 2 2 3 3 2 2 3 3 2 2 3
3 2 2 3 3 2 2 3 3
ph5= 0
ph31=0 2 2 0 2 0 0 2 2 0 0 2 0 2 2 0 0 2 2 0 2 0 0 2 2 0 0 2 0 2 2 0 0 2 2 0 2 0 0 2 2 0 0 2 0 2 2 0 0 2 2 0 2 0
0 2 2 0 0 2 0 2 2 0

;pl0 : zero power (120 dB)
;pl1 : high power (dB)
;p1 : 90 degree high power pulse
;p16 : duration of CTP gradients (1m)
;p20 : duration of weak gradient during PSYCHE pulse element
;p21 : duration of weak gradient during 1st 180-degree swept-frequency pulse
;p22 : duration of weak gradient during 2nd 180-degree swept-frequency pulse
;p43 : duration of double-chirp PSYCHE pulse element
;p41 : duration of 1st 180-degree swept-frequency pulse
;p42 : duration of 2nd 180-degree swept-frequency pulse
;d0 : incremented delay
;d1 : relaxation delay
;d16 : recovery delay for gradients
;spw43 : RF power of double-chirp PSYCHE pulse element
;spw41 : RF power of 1st 180-degree swept-frequency pulse
;spw42 : RF power of 2nd 180-degree swept-frequency pulse
;spnam43: file name for PSYCHE pulse element
;spnam41: file name for 1st 180-degree swept-frequency pulse
;spnam42: file name for 2nd 180-degree swept-frequency pulse
;gpz1: CTP gradient (35%)
;gpz2: CTP gradient (49%)
;gpz3: CTP gradient (77%)
;gpz10: weak gradient during PSYCHE element (1-3%)
;gpz11: weak gradient during 1st 180-degree chirp (1-3%)
;gpz12: weak gradient during 2nd 180-degree chirp (1-3%)
;gpnam1: SINE.100
;gpnam2: SINE.100
;gpnam3: SINE.100
;gpnam10: RECT.1
;gpnam11: RECT.1
;gpnam12: RECT.1
;cnst20: desired flip angle for PSYCHE pulse element (degree) (normally 10-25)
;cnst21: bandwidth of each chirp in PSYCHE pulse element (Hz) (normally 10000)
;cnst51: RF amplitude for 180-degree chirp pulses (Hz)
;l0 : loop for N/R cycle
;in0 : 1/(2 * SW) = DW
;nd0 : 2
;td1 : number of t1 increments
;MC2 : EA

9 Reactivity of $IMeBH_2$-esters

9.1 Synthesis of IMe-BF_2-ester (compound 6) from 2a

A Schlenk flask was loaded with **IMe-BH_2-ester 2a** (50 mg, 0.24 mmol) before being placed under an atmosphere of N_2. Acetonitrile (5 mL) was added, followed by Selectfluor (169 mg, 0.48 mmol) before the reaction mixture was left to mix for 2 hours. The solvent was subsequently removed under vacuum to yield the crude product as a white solid. Column chromatography (SiO_2, 10:1 ethyl acetate:pet. ether) was used to purify the product. The identity of the title compound was confirmed by ^{1}H, $^{13}C\{^{1}H\}$, ^{11}B and ^{19}F NMR spectroscopy and X-ray crystallography carried out upon crystals grown by slow evaporation from a chloroform solution of the product.

^{1}H NMR (500 MHz, Chloroform-d) δ 6.85 (s, 2H), 3.84 (s, 6H), 3.48 (s, 3H), 1.83 – 1.76 (m, 1H), 1.75 – 1.69 (m, 1H), 1.61 – 1.52 (m, 1H), 0.88 (t, J = 7.1 Hz, 3H).**^{13}C{^{1}H} NMR** (126 MHz, Chloroform-d) δ 179.1 (dd, J = 7, 3 Hz), 121.9, 50.7, 47.0 – 44.2 (m), 36.7 (t, J = 5 Hz), 20.0 (t, J = 2 Hz), 15.0.**^{11}B NMR** (128 MHz, Chloroform-d) δ 4.6 (t, J = 68 Hz).**^{19}F NMR** (376 MHz, Chloroform-d) δ -154.2 – -156.1 (m), -158.0 – -159.5 (m).

9.2 In situ synthesis of IMe-BCl_2-ester (compound 7) from 2a

A Young's ampule was charged with a sample of IMe-BH_2-ester **2a** (50 mg, 0.24 mmol) and placed under an N_2 atmosphere. DCM (4 mL) was added, followed by N-chlorosuccinimide (64 mg, 0.48 mmol). The solution was stirred for 10 minutes after which time an aliquot of the reaction was taken for analysis by NMR spectroscopy, demonstrating clean conversion in both ^{1}H and ^{11}B NMR spectra based on comparison with the similar compound reported by Curran et al.[12] Subsequently a solution of pinacol in triethylamine (1 M, 240 µL) was added and the solution was mixed. NMR spectroscopic analysis after 72 hours showed no reaction. H_20 (0.1 mL) was then added and the reaction mixture was left to stir for 1 week, after which time the ^{1}H and ^{11}B NMR spectra displayed complete decomposition of the product.

Screening of Suzuki-Miyaura reactions conditions:

These R(NHC)BF_2 and R(NHC)BCl_2 compounds were explored for use in Suzuki-Miyaura cross coupling reactions with 4-iodoanisole and 4-CF_3-bromobenzene. The conditions explored included:

THF/H_2O / Pd(dppf)Cl_2

THF/H_2O / $Pd_2(dba)_3$/SPhos / K_3PO_4

THF/H2O / SPhos / Ag_2O

Furthermore, attempts were made to convert a range of boranes to an active (for transmetallation) species by (i) attempted conversion of the BCl_2 to $B(OMe_2)$ derivative (by action of TMSOMe) and (ii) by reaction of 2a with tosic acid, which evolved H_2 to form the $B(OTs)_2$ derivative. Anhydrous couplings conditions then were used (based on the report using α-BPin ester in Suzuki-Miyaura couplings– specifically $Pd_2(dba)_3$ / Ag_2O (or other bases e.g. NaOMe), catacxium / toluene / heat) with the haloarenes mentioned above. However, in all cases only minimal cross coupled product was observed at best, with protodeboronation dominating.

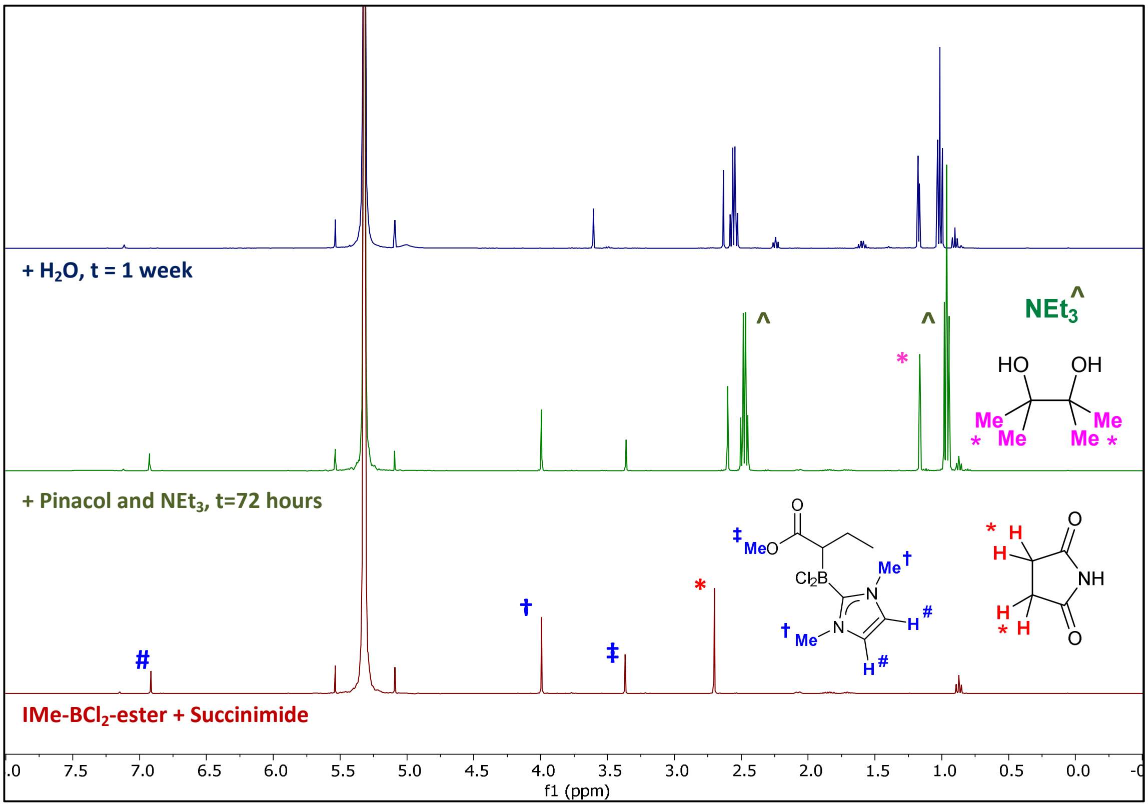

Figure S13: Collected ^{1}H NMR spectra (CH_2Cl_2, 128.4 MHz, 298K) of: **2a** and NCS (bottom); the reaction mixture after 72 hours from pinacol and Et_3N addition (middle); the reaction mixture after 1 week from water addition (top).

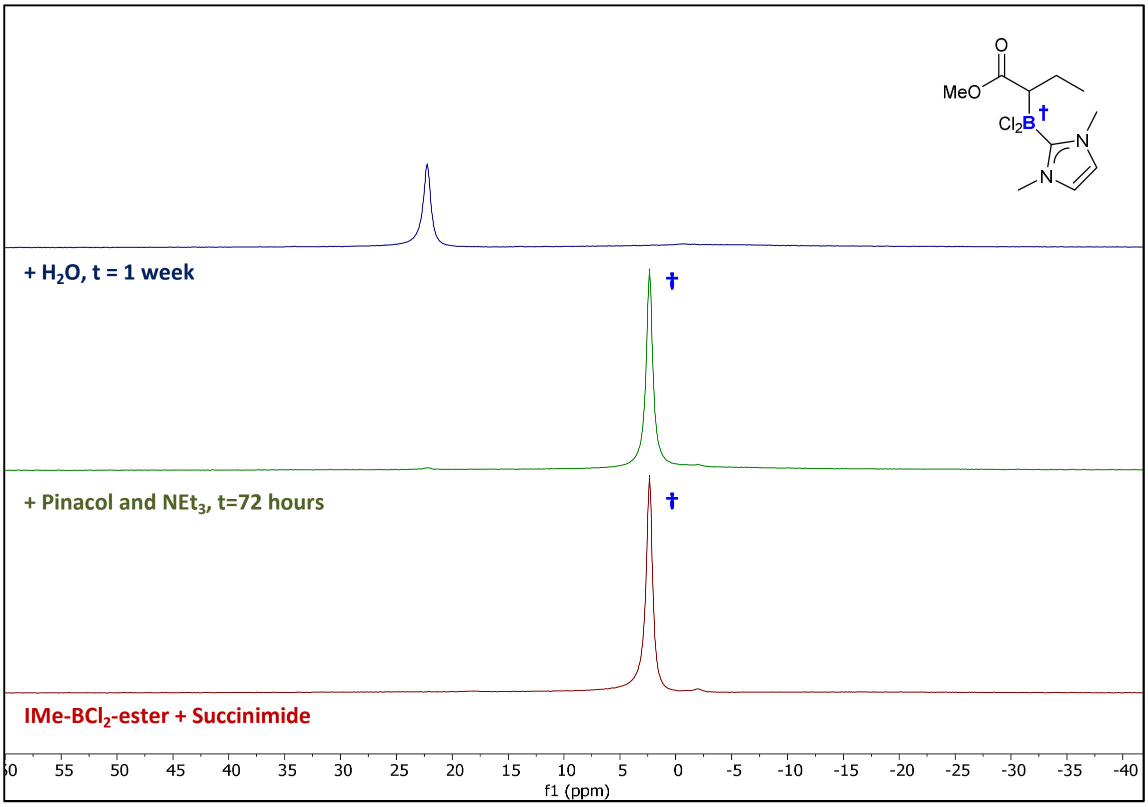

Figure S14: Collected ^{11}B NMR spectra (CH_2Cl_2, 128.4 MHz, 298K) of: **2a** and NCS (bottom); the reaction mixture after 72 hours from pinacol and Et_3N addition (middle); the reaction mixture after 1 week from water addition (top)

9.3 In situ Synthesis of IMe-BCl_2-ester from 2k

A Young's ampule was charged with a sample of IMe-BH_2-ester **2k** (58 mg, 0.21 mmol) and placed under an N_2 atmosphere. DCM (4 mL) was added, followed by N-chlorosuccinimide (59 mg, 0.43 mmol). The solution was stirred for 10 minutes after which time an aliquot of the reaction was taken for analysis by NMR spectroscopy, demonstrating clean conversion in both ^{1}H and ^{11}B NMR spectra based on comparison with similar $RBCl_2$(NHC) compounds reported by Curran et al.[12] Subsequently, unpurified pinacol (26 mg, 0.21 mmol) and Et_3N (209 µL, 1.50 mmol) were added and the solution was mixed. NMR spectroscopic analysis after 18 hours at room temperature showed no reaction. However, heating at 60°C for 18 hours displayed complete decomposition of the product.

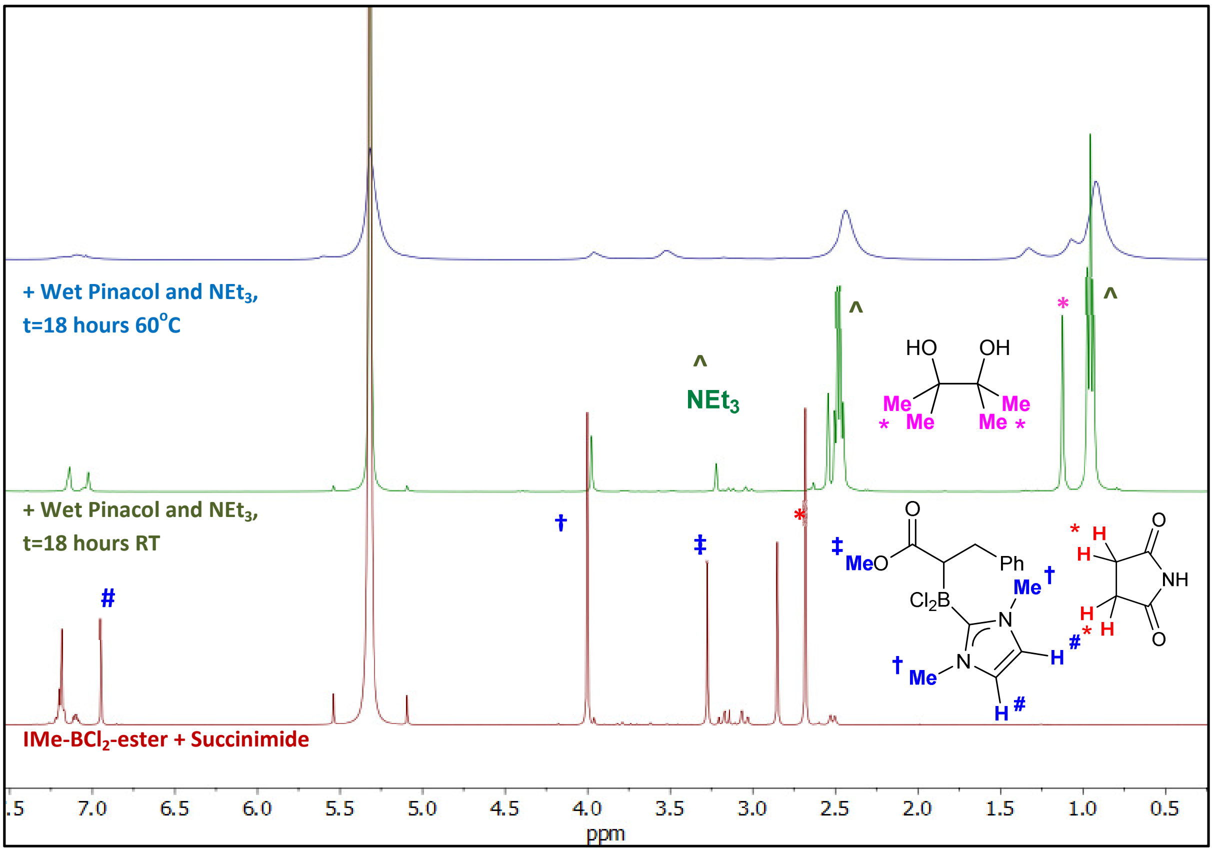

Figure S15: Collected ^{1}H NMR spectra (CH_2Cl_2, 128.4 MHz, 298K) of: **2k** and NCS (bottom); the reaction mixture after 18 hours at RT from wet pinacol and Et_3N addition (middle); the reaction mixture after 18 hours at 60°C from wet pinacol and Et_3N addition (top).

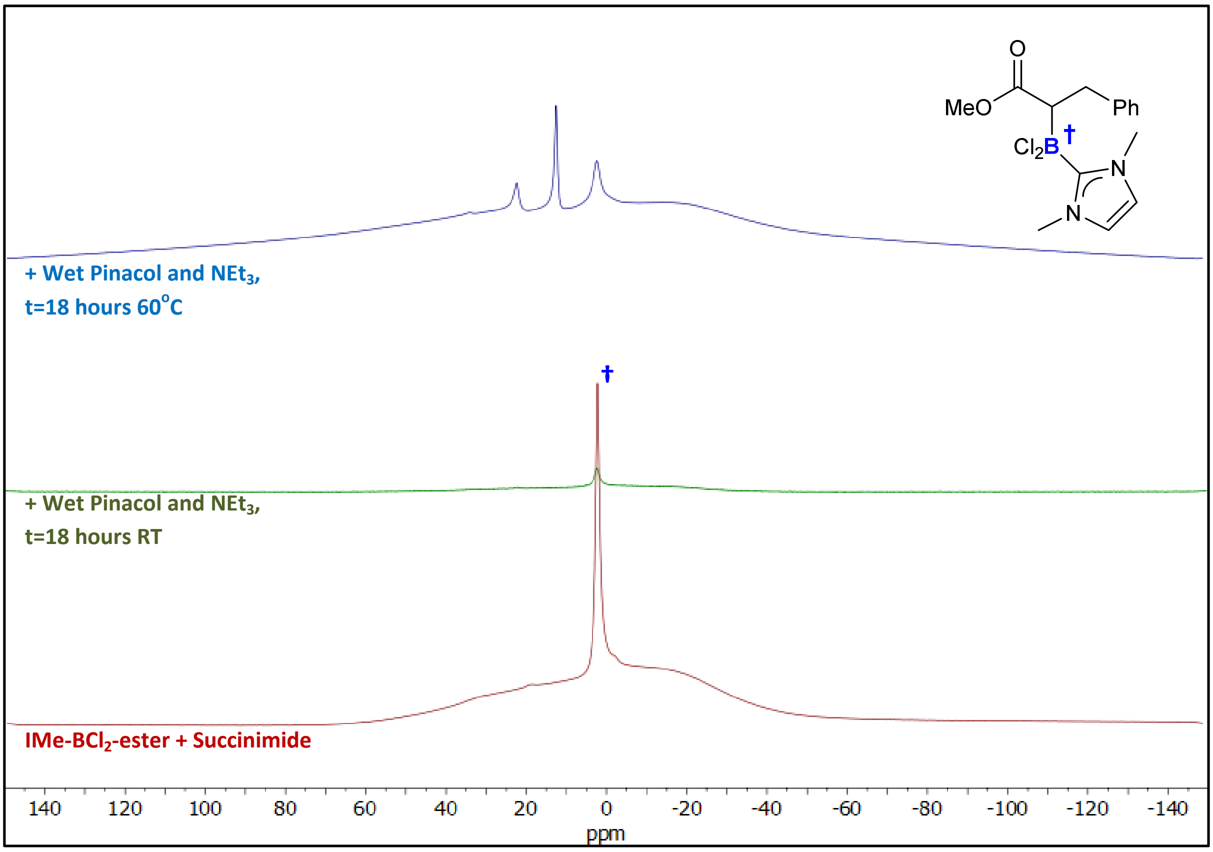

Figure S16: Collected ^{1}H NMR spectra (CH_2Cl_2, 128.4 MHz, 298K) of: **2k** and NCS (bottom); the reaction mixture after 18 hours at RT from wet pinacol and Et_3N addition (middle); the reaction mixture after 18 hours at 60°C from wet pinacol and Et_3N addition (top).

9.4 Reduction of IMe-BH$_2$-ester 2a with LiAlH$_4$

A sample of IMe-BH$_2$-ester **2a** (42 mg, 0.2 mmol) was placed in an ampule and placed under an N_2 atmosphere. Diethyl ether was added before the resulting solution was cooled to 0 °C. A suspension of $LiAlH_4$ (15 mg, 0.4 mmol) in diethyl ether (1 mL) was added dropwise and the mixture was left to stir at 0 °C for 3 hours. H_2O (0.5 mL) was added dropwise before being allowed to warm to room temperature and left to stir for 16 hours. Further H_2O was added (5 mL) before being washed with DCM (3 x 5 mL). The organic fractions were combined, dried over $MgSO_4$, filtered and the solvent removed under vacuum, to yield a clear, colourless oil. The oil was passed through a silica plug, eluted with 95:5 DCM:MeOH. The product **8** was obtained as a colourless oil (28 mg, 78 % yield). The identity of the resulting product was confirmed as that of the title compound by ^{1}H, ^{13}C{^{1}H} and ^{11}B NMR spectroscopy. Characterisation was further confirmed by conversion to the known pinacol boronate ester derivative (see below for conditions)

^{1}H NMR (400 MHz, Chloroform-*d*) δ 6.81 (s, 2H), 3.75 (s, 6H), 3.60 – 3.52 (m, 1H), 3.47 – 3.37 (m, 1H), 2.08 (t, *J* = 5.6 Hz, 1H), 1.37 – 1.20 (m, 1H this resonance is overlapped with the ^{11}B-H 1:1:1:1 resonances), 1.18 – 1.05 (m, 1H), 0.80 (t, *J* = 7.4 Hz, 3H).

^{13}C{^{1}H} NMR (101 MHz, Chloroform-*d*) δ 172.9 (m), 120.40, 68.93, 36.18, 34.5 (br), 26.32, 13.68.

^{11}B NMR (128 MHz, Chloroform-*d*) δ -27.60 (t, *J* = 82.9 Hz).

9.5 **Reduction of IMe-BH$_2$-ester 2a with DIBAL-H**

An identical method to that used for the reduction of **2a** with $LiAlH_4$ was used with **2a** (42 mg, 0.2 mmol) and DIBAL-H (1M in hexanes, 0.4 mL, 0.4 mmol) in place of $LiAlH_4$. The identity of the product was confirmed by the ^{1}H and ^{11}B NMR spectra matching those reported above for IMe-BH$_2$-alcohol **8**.

9.6 **Reduction of IMe-BH$_2$-ester 2k with LiAlH$_4$**

A Schlenk flask was charged with **2k** (544 mg, 2 mmol) and placed under an atmosphere of N_2. Diethyl ether (5 mL) was added, while in a separate flask a suspension of $LiAlH_4$ (152 mg, 4 mmol) in diethyl ether (5 mL) was prepared. Both mixtures were cooled to – 78 °C before the $LiAlH_4$ suspension was added slowly to the ester solution. The solution was subsequently allowed to warm to room temperature and left stirring for 16 hours. The remaining $LiAlH_4$ was quenched by careful addition of saturated aqueous sodium potassium tartrate solution (20 mL total). The aqueous fraction was washed with diethyl ether (3 x 20 mL) before the organic fractions were combined, dried over $MgSO_4$ and filtered. The solvent was removed under vacuum to yield a crude product as a colourless oil. ^{11}B NMR spectroscopy revealed that the crude mixture contained the desired alcohol **9** product, and an unknown by-product (~15 % by ^{11}B NMR).

Thus in this case DIBAL-H is the preferred reagent (see below) which reacts without the side product formation.

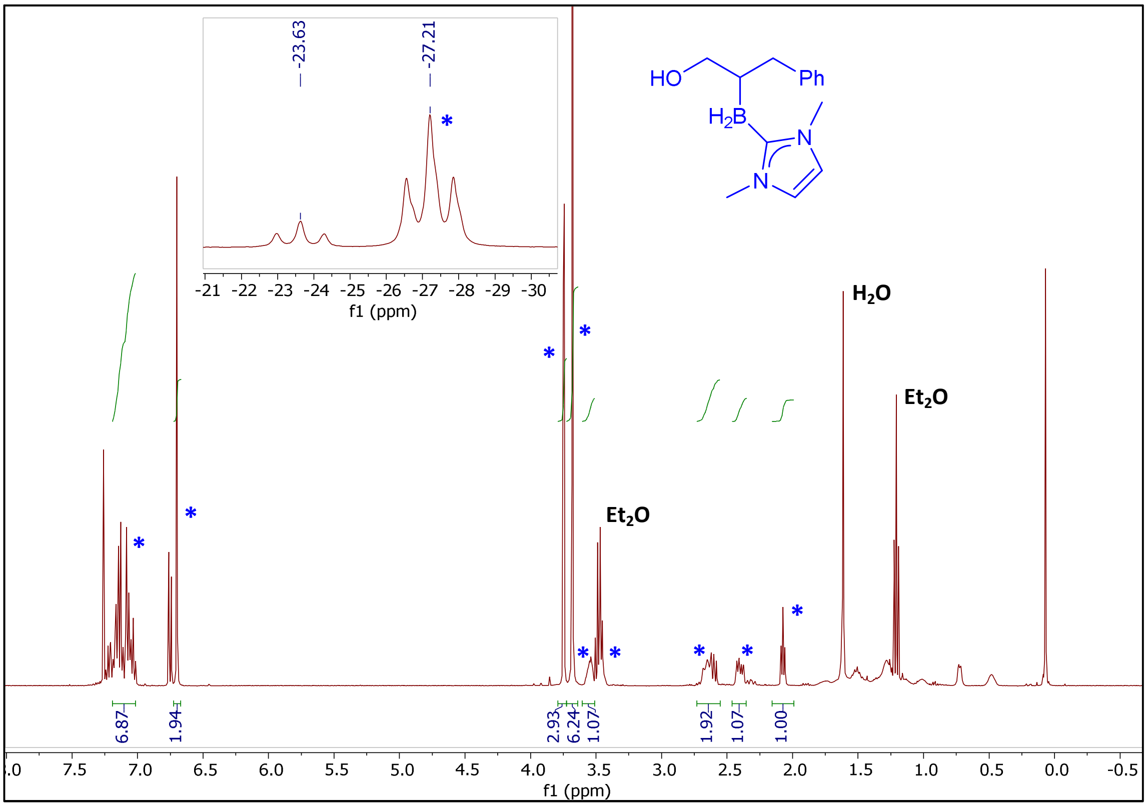

Figure S17: Crude mixture for reduction of 2k with $LiAlH_4$, note the additional C-BH$_2$(IMe) species and the additional resonances between 0.4 and 0.75 ppm. * = resonances assigned to the desired compound.

9.7 Reduction of IMe-BH_2-ester 2k with DIBAL-H

A Schlenk flask was charged with IMe-BH_2-Ph ester **2k** (272mg, 1 mmol) and placed under an N_2 atmosphere, before adding diethyl ether (3 mL). The solution was cooled to -78 °C before DIBAL-H solution (1 M in hexanes, 2.2 mL, 2.2 mmol) was added dropwise. The reaction was left to stir at -78 °C for 3 hours before being removed from the cold bath and aqueous sodium potassium tartrate (2 mL sat. solution) added while cold. The mixture was left to warm to room temperature before more sodium potassium tartrate solution (15 mL) was added. The aqueous phase was washed with diethyl ether (3 x 10 mL) and the organic fractions were combined, dried over $MgSO_4$, filtered and the solvent removed under vacuum. The product **9** was isolated as a clear oil (220 mg, 90 % yield) which was characterised as the title compound by ^{1}H, ^{13}C{^{1}H} and ^{11}B NMR spectroscopy.

^{1}H NMR (400 MHz, Benzene-d_6) δ 7.26 – 7.20 (m, 2H), 7.12 (t, *J* = 7.5 Hz, 2H), 7.01 (t, *J* = 7.3 Hz, 1H), 5.60 (s, 2H), 3.95 (s, 2H), 3.05 (dd, *J* = 12.7, 8.4 Hz, 1H), 2.99 (s, 6H), 2.68 (dd, *J* = 13.2, 6.7 Hz, 1H), 2.32 (s, 1H), 2.23 – 1.58 (m, 2H), 1.46 (s, 1H).

^{13}C NMR (101 MHz, Benzene-d_6) δ 173.97 – 172.03 (m), 145.72, 129.18, 124.99, 119.55, 69.35, 41.52, 36.53 – 34.59 (m), 35.35.

^{11}B NMR (128 MHz, Benzene-d_6) δ -26.76 (t, *J* = 84.5 Hz).

9.8 Pinacol protection of IMe-BH_2-alcohol 8

A Young's ampule was charged with IMe-BH_2-alcohol **8** (80 mg, 0.44 mmol) and placed under an atmosphere of N_2. Toluene (1 mL) was added, followed by pinacol (104 mg, 0.88 mmol). The resulting solution was cooled to 0 °C before adding N-chlorosuccinimide (118 mg, 0.88 mmol). The mixture was allowed to warm to room temperature and stirred for 16 hours. The toluene solution was removed from the flask, and the flask was washed with pentane (3 x 5 mL). The organic fractions were combined and the solvent removed under vacuum to yield a clear colourless oil. Analysis of the oil by ^{11}B NMR spectroscopy revealed two signals (34.1 ppm, 22.5 ppm). All attempts to isolate a single product from the mixture yielded increasing proportions of protodeborylation products. The ^{1}H NMR was consistent with the desired product by comparison to the previously reported compound.

9.9 Pinacol protection of IMe-BH_2-Phalcohol 9

A sample of **9** (244 mg, 1 mmol) was placed in a Schlenk flask and placed under an N_2 atmosphere and pinacol (236 mg, 2 mmol) added. The solids were dissolved in toluene (5 mL) before adding N-chloro-succinimide (267 mg, 2 mmol) and stirring for 2 hours. The toluene solution was removed from the flask which was subsequently washed with pentane (3 x 5 mL). The organic fractions were combined and the solvent removed under vacuum. The oily residue was passed through a silica plug, eluting two fractions, first with pet. ether:ethyl acetate (10:1) followed by a second eluted with neat ethyl acetate. The solvent was removed from both fractions yielding two clear oils. The first pet. ether fraction was analysed by NMR spectroscopy and found to contain primarily a mixture of **11** and **12** (139 mg, 51 % yield) which were assigned based on previously reported data for both compounds.[13] The second neat ethyl fraction was found to be primarily Phalcohol-BPin **10** (81 mg, 31 % yield) which was characterised by its ^{1}H NMR spectrum matching that previously reported by Hoveyda et al.[15]

9.9.1 By-products 11 and 12

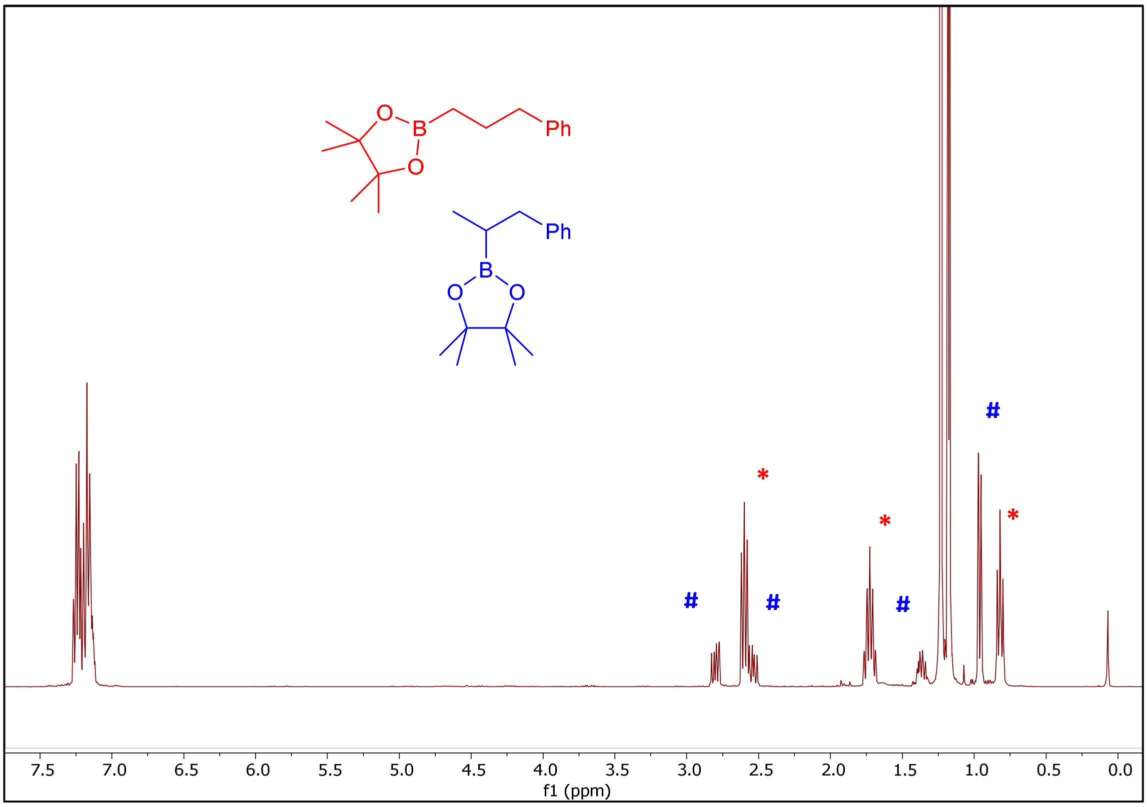

Figure S18: ^{1}H NMR spectrum of the by-product mixture derived from the protection at boron with pinacol using NCS/pinacol.

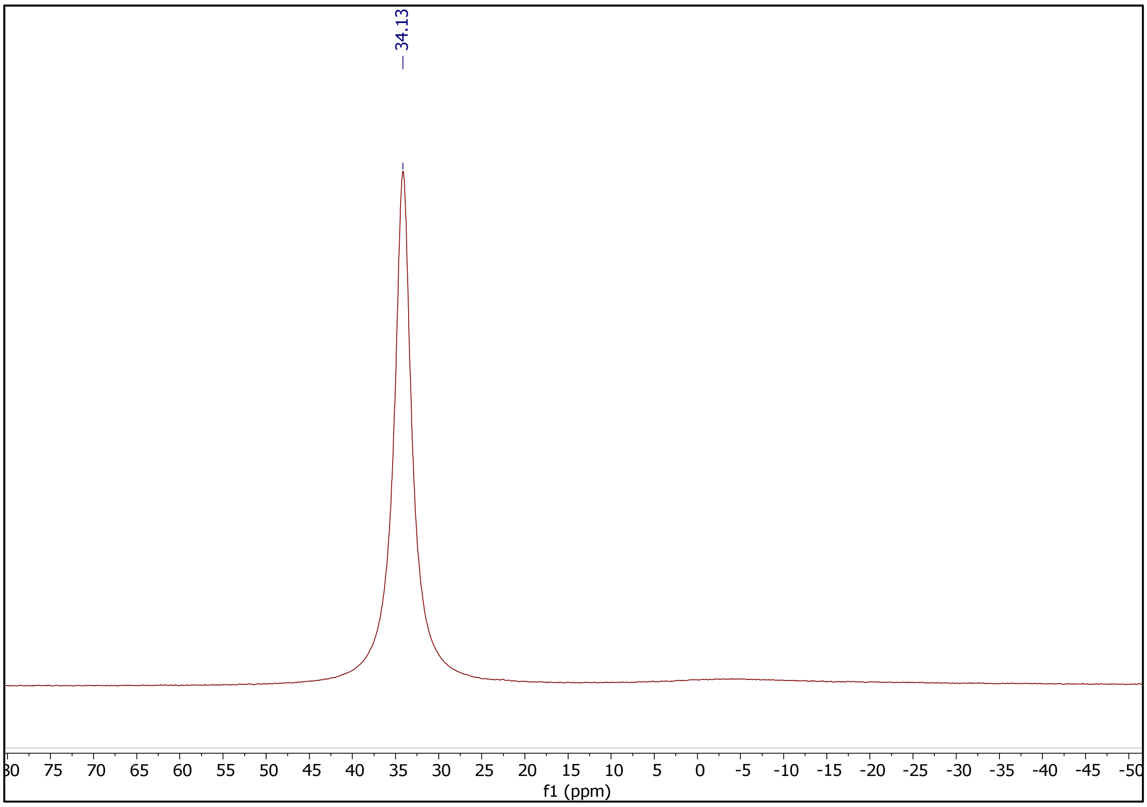

Figure S19: ^{11}B NMR spectrum of the product mixture derived from the protection at boron with pinacol using NCS/pinacol.

9.9.2 IMe-BH$_2$-Phalcohol 10

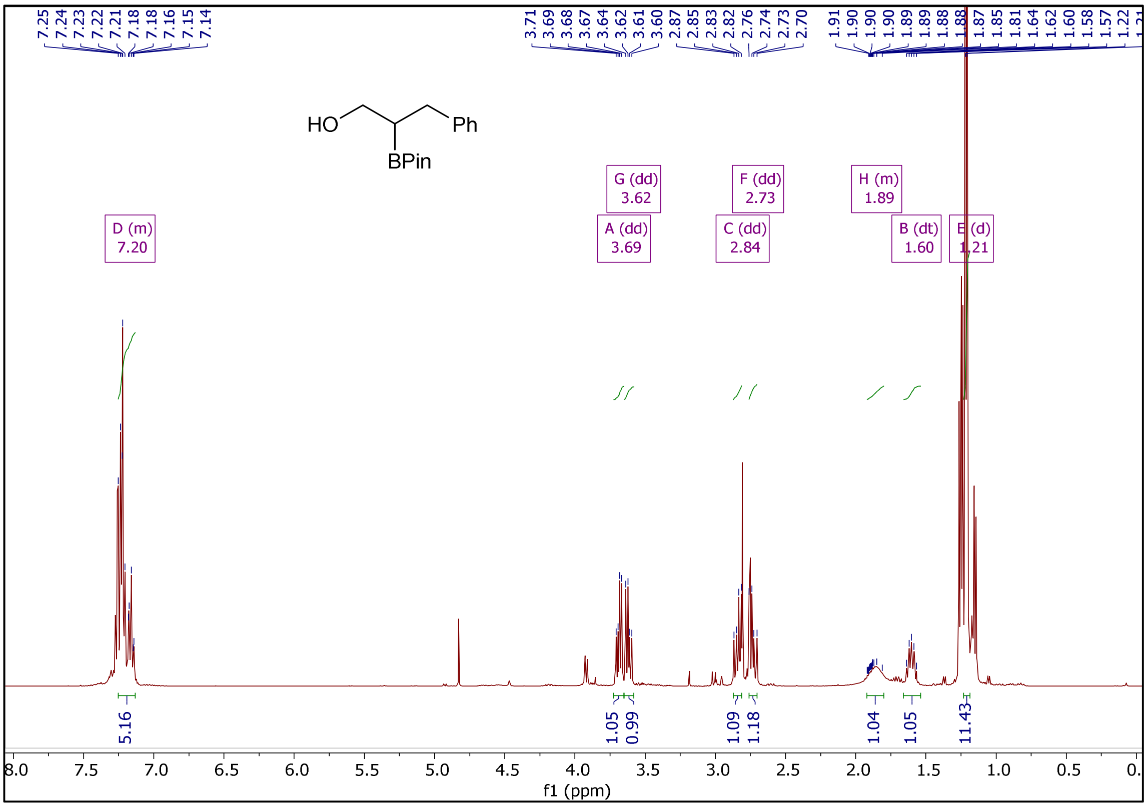

Figure S20: ^{1}H NMR of the desired beta-boryl alcohol

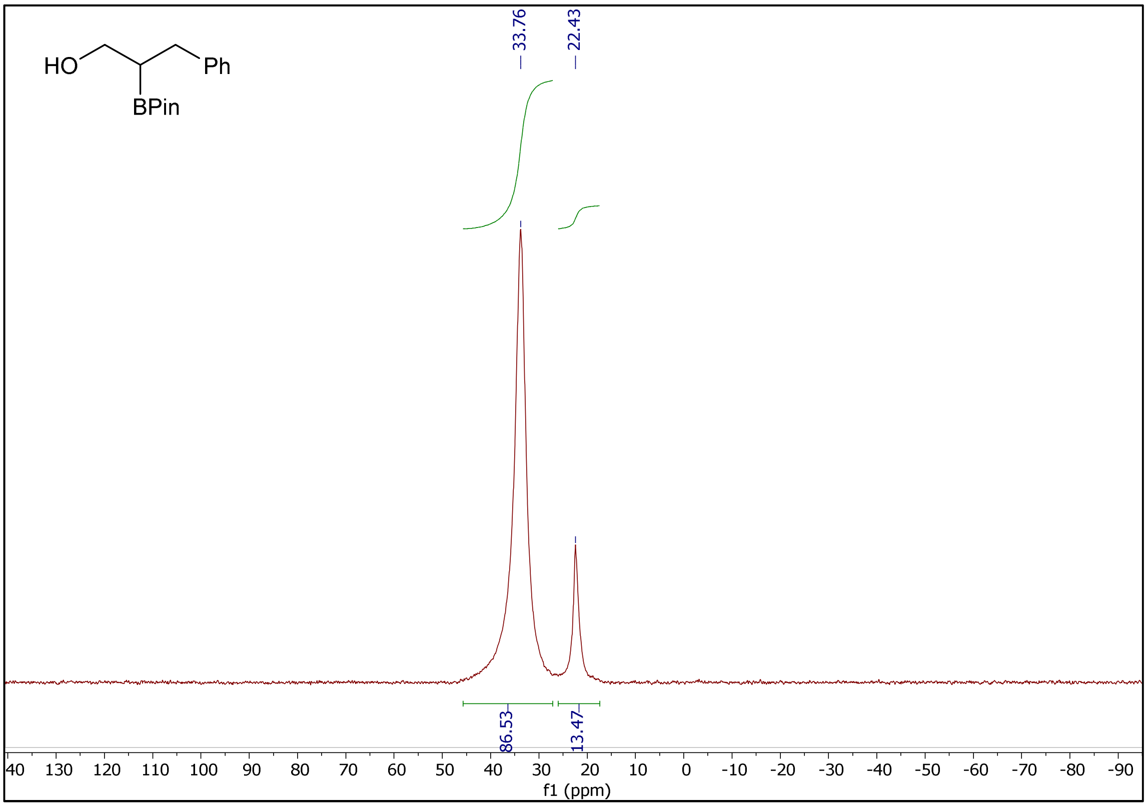

Figure S21: ^{11}B NMR spectrum of the desired beta-boryl alcohol. Minor impurity at 22 ppm is assigned to a protodeboronation by-product

10 Oxidation of IMe-BH_2-Products

10.1.1 Reduction of (S)-2s, followed by the in-situ oxidation of the alcohol

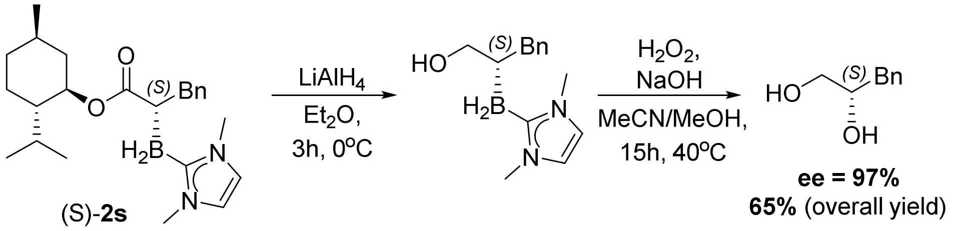

A sample of the ester (S)-**2s** (28 mg, 0.071 mmol) was placed in an ampoule and placed under an N_2 atmosphere. Diethyl ether was added before the resulting solution was cooled to 0 °C. A suspension of $LiAlH_4$ (6 mg, 0.141 mmol) in diethyl ether (1 mL) was added dropwise and the mixture was left to stir at 0 °C for 3 hours. H_2O (0.5 mL) was added dropwise before being allowed to warm to room temperature and left to stir for 16 hours. Further H_2O was added (5 mL) before being washed with DCM (3 x 5 mL). The organic fractions were combined, dried over $MgSO_4$, filtered and the solvent removed under vacuum, to yield a clear, colourless oil (mixture of the desired alcohol and the byproduct menthol). The oil was then transfer in an ampoule and dissolved in 2 mL of a 1:1 mixture of MeCN/MeOH, followed by the addition a 30% v/v solution of H_2O_2 (58 µL, 0.566 mmol) and NaOH (13 mg, 0.325 mmol). A drop of water was then added to enable NaOH solubilisation. The ampoule was sealed and place in an oil bath at 40°C overnight. Then, the system was cooled down to RT, followed by extraction with DCM (3 x 3 mL). The organic fractions were combined, dried over $MgSO_4$, filtered and the solvent removed under vacuum, to yield a clear oil. Purification of the oil by flash-chromatography (Pet. Ether : Et_2O = 1:1) gave (S)-3-phenylpropane-1,2-diol (R_f = 0.1) as a white solid (7 mg, 0.046 mmol, 65%). The data are in agreement with those reported in the literature.[15]

^{1}H NMR (400 MHz, Chloroform-*d*) δ 7.33 (t, *J* = 7.5 Hz, 2H), 7.21-7.25 (m, 3H), 3.93-3.99 (m, 1H), 3.71 (dd, *J* = 11.3, *J* = 3.3 Hz, 1H), 3.53 (dd, *J* = 11.3, *J* = 7.0 Hz, 1H), 2.70-2.76 (m, 2H), 1.90-2.20 (bs, 2H).

^{13}C{^{1}H} NMR (101 MHz, Chloroform-*d*) δ 137.7, 129.3, 128.7, 126.6, 73.0, 66.0, 39.8.

The enantiomeric excess (97%) was determined using HPLC analysis (Chiracel OD-H, Hexane : IPA 98:2, flow rate = 1.5 mL/min, 20°C, 220 nm). The references for the racemic mixture and the authentic (S)-enantiomer of 3-phenylpropane-1,2-diol were prepared by reduction with $LiAlH_4$ of (rac)-3-Phenyllactic acid and (S)-3-Phenyllactic lactic, respectively.

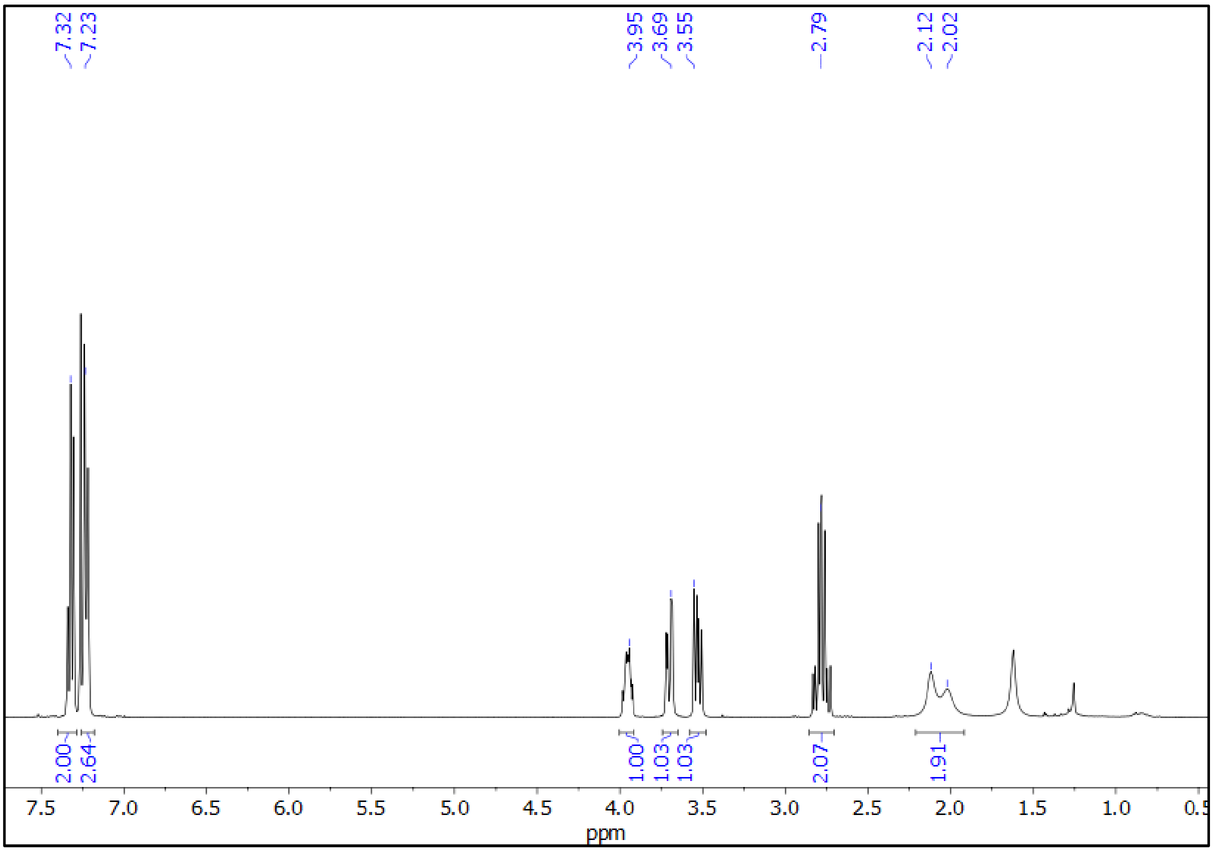

Figure S22: ^{1}H NMR spectrum ($CDCl_3$, 400 MHz, 298K) of (S)-3-phenylpropane-1,2-diol.

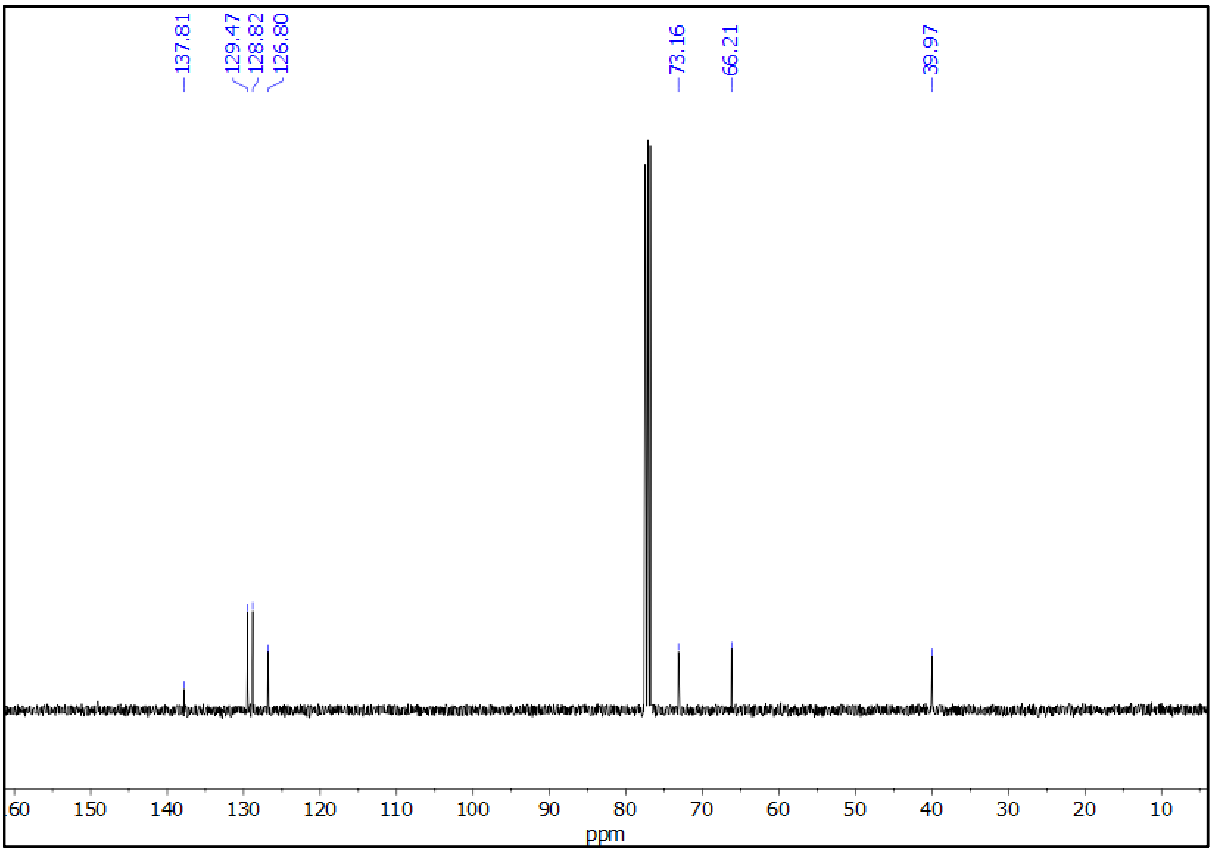

Figure S23: $^{13}C\{^{1}H\}$ NMR spectrum ($CDCl_3$, 400 MHz, 298K) of (S)-3-phenylpropane-1,2-diol.

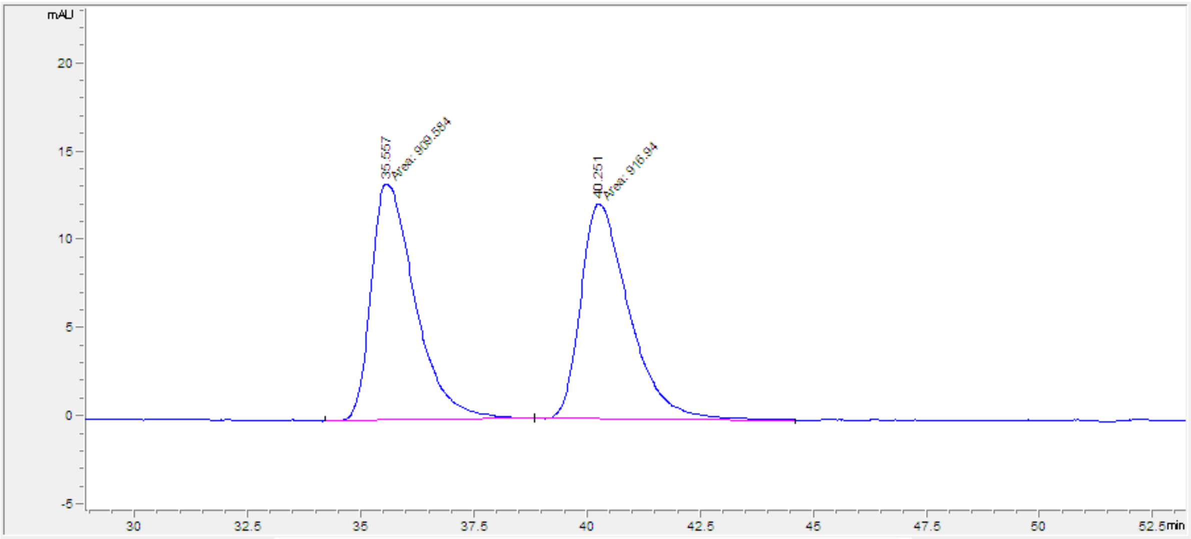

#	Time	Area	Height	Width	Area%	Symmetry
1	35.557	909.6	13.4	1.1333	49.799	0.53
2	40.251	916.9	12.2	1.2499	50.201	0.589

Figure S24: HPLC analysis of (rac)-3-phenylpropane-1,2-diol.

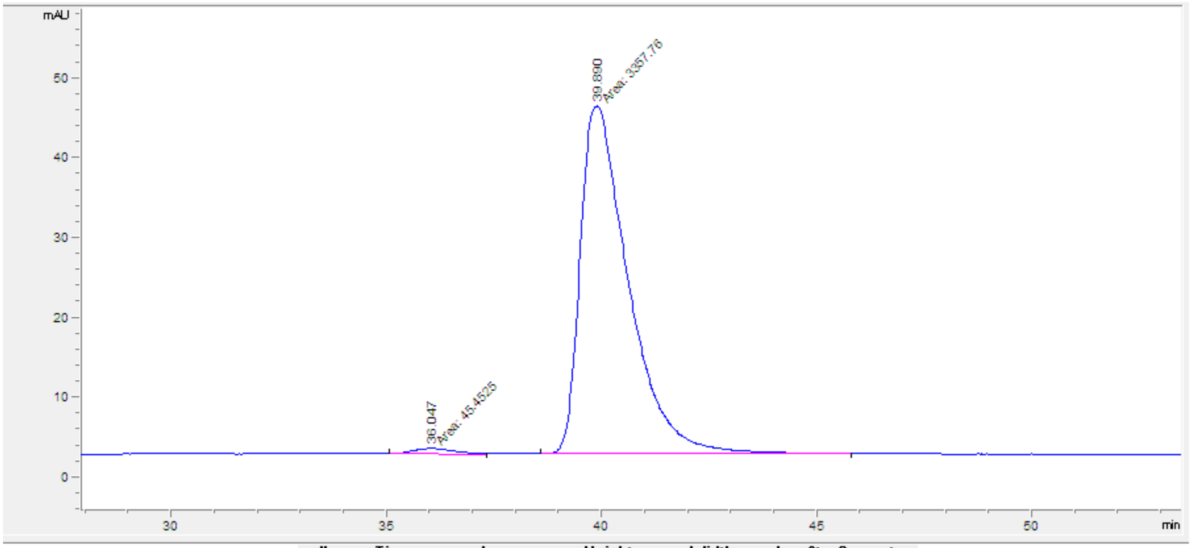

#	Time	Area	Height	Width	Area%	Symmetry
1	36.047	45.5	6.7E-1	1.1248	1.336	0.686
2	39.89	3357.8	43.5	1.2864	98.664	0.53

Figure S25: HPLC analysis of (S)-3-phenylpropane-1,2-diol isolated from the reaction.

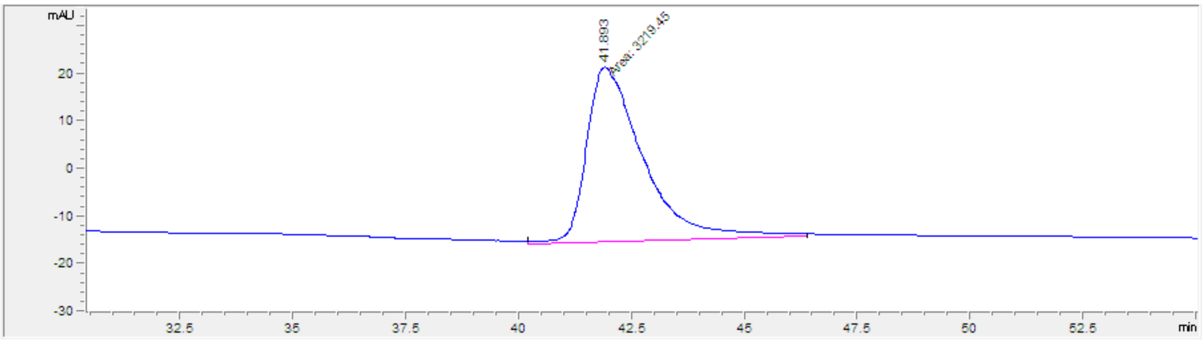

Figure S26: HPLC analysis of (S)-3-phenylpropane-1,2-diol independently synthesized.

11 Computational Details

11.1 General Comments

All calculations were conducted at the M06-2X/6-311G(d,p) level with a solvation model (PCM, CH_2Cl_2) using the Gaussian software package.[16] All geometry optimizations were full, with no restrictions. In all cases, structures were confirmed as minima by frequency analysis and the absence of imaginary frequencies.

11.2 Full table of relative borylation energies of α,β-unsaturated carbonyl compounds

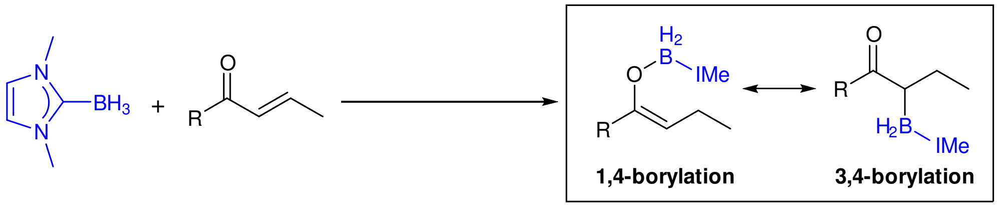

α,β-unsaturated carbonyl compound	Energy of borylation, ΔE		1,4 → 3,4 isomerisation ΔE (kcal mol^{-1})
	1,4 borylation (kcal mol^{-1})	3,4 borylation (kcal mol^{-1})	
Butenal	-34.845	-29.394	+5.5
2,2-Dimethylhexenone	-29.589	-24.868	+4.7
Pentenone	-29.109	-28.405	+0.7
N,N-dimethyl buteneamide	-18.740	-26.577	-7.8
Methyl crotonate	-16.931	-28.991	-12.1

Table S4: Energy of the 1,4 and 3,4 hydroboration products relative to the starting materials in each case.

12 X-ray crystallographic details

12.1 General Comments

Crystallographic data for compound **IMe-BH$_2$-ester 2a** were recorded on an SAXI (Bruker) diffractometer, at 150 K with Cu Kα radiation (mirror monochromator, λ =1.54184). Crystallographic data for compound **BenzIMe-BH$_2$-ester 4a** were recorded on an Agilent Supernova diffractometer, at 150 K with Mo Kα radiation (mirror monochromator, λ =0.7107). Crystallographic data for compounds **IMe-BF$_2$-ester 6, IMe-BH$_2$-lactone 2i**, **IMe-BH$_2$-menthyl ester 2s** and **IMe-BH$_2$-8-phenyl menthyl ester 2t** were collected on a Rigaku FRX diffractometer with Cu Kα radiation (graphite monochromator, λ =1.54184) at 150 K. The CrysAlisPro[17] software package was used for data collection, cell refinement and data reduction. All further data processing was undertaking within the Olex2 software.[18] The molecular structures were solved with ShelXT[19] structure solution program using Intrinsic Phasing. The model was refined with the SHELXL[20-22] refinement package using Least Squares minimisation against F^2. Non-hydrogen atoms were refined anisotropically, while hydrogen atoms were all located in a difference map and repositioned geometrically. For compound **IMe-BH$_2$-lactone 2i** the lactone-ring hydrogens were freely refined, while the remaining hydrogens were located in a difference map and repositioned geometrically.

12.2 **IMe-BH_2-ester 2a**

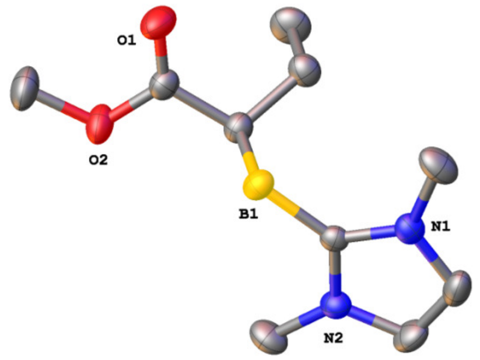

Figure S27: ORTEP plot for the molecular structure of compound **2a** with thermal ellipsoids set at 50 % level and hydrogens omitted for clarity

CCDC Number	1864777
Empirical formula	$C_{10}H_{19}BN_2O_2$
Formula weight	210.08
Temperature/K	150
Crystal system	monoclinic
Space group	$P2_1$
a/Å	6.6119(2)
b/Å	12.8257(2)
c/Å	7.7086(2)
α/°	90
β/°	111.978(3)
γ/°	90
Volume/Å^3	606.20(3)
Z	2
ρ_{calc}g/cm^3	1.151
μ/mm^{-1}	0.629
F(000)	228.0
Crystal size/mm^3	0.1 × 0.1 × 0.1
Radiation	CuKα (λ = 1.54184)
2Θ range for data collection/°	12.382 to 149.246
Index ranges	-8 ≤ h ≤ 7, -16 ≤ k ≤ 15, -9 ≤ l ≤ 9
Reflections collected	8208
Independent reflections	2404 [R_{int} = 0.0219, R_{sigma} = 0.0188]
Data/restraints/parameters	2404/1/148
Goodness-of-fit on F^2	1.072
Final R indexes [I>=2σ (I)]	R_1 = 0.0359, wR_2 = 0.0941
Final R indexes [all data]	R_1 = 0.0383, wR_2 = 0.0958
Largest diff. peak/hole / e Å^{-3}	0.20/-0.16
Flack parameter	0.44(11)

Table S5: Table of crystallographic information for compound **2a**

12.3 **BenzIMe-BH_2-ester 4a**

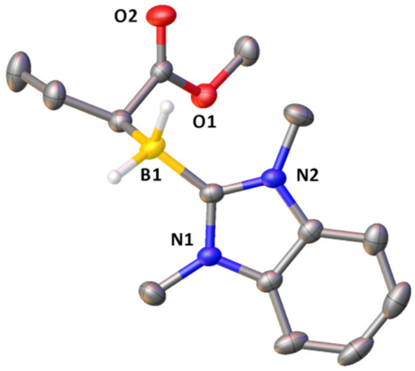

Figure S28: ORTEP plot for the molecular structure of compound **4a** with thermal ellipsoids set at 50 % level and most hydrogens omitted for clarity

CCDC Number	1864774
Empirical formula	$C_{14}H_{21}BN_2O_2$
Formula weight	260.14
Temperature/K	149.9(3)
Crystal system	monoclinic
Space group	I2/a
a/Å	22.1499(9)
b/Å	7.8915(2)
c/Å	17.5288(6)
α/°	90
β/°	111.175(4)
γ/°	90
Volume/Å^3	2857.09(18)
Z	8
ρ_{calc}g/cm^3	1.210
μ/mm^{-1}	0.080
F(000)	1120.0
Crystal size/mm^3	0.9 × 0.6 × 0.3
Radiation	MoKα (λ = 0.71073)
2Θ range for data collection/°	5.526 to 57.948
Index ranges	-27 ≤ h ≤ 29, -9 ≤ k ≤ 10, -23 ≤ l ≤ 23
Reflections collected	13081
Independent reflections	3383 [R_{int} = 0.0374, R_{sigma} = 0.0345]
Data/restraints/parameters	3383/0/184
Goodness-of-fit on F^2	1.108
Final R indexes [I>=2σ (I)]	R_1 = 0.0481, wR_2 = 0.1199
Final R indexes [all data]	R_1 = 0.0591, wR_2 = 0.1258
Largest diff. peak/hole / e Å^{-3}	0.23/-0.20

Table S6: Table of crystallographic information for compound **4a**

12.4 **IMe-BF_2-ester 6**

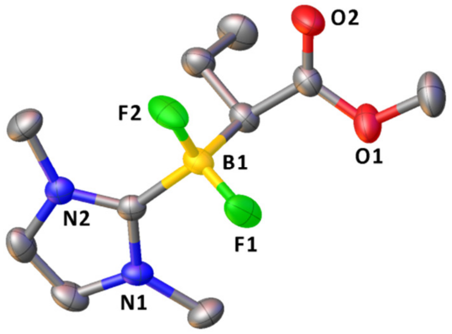

Figure S29: ORTEP plot for the molecular structure of compound **6** with thermal ellipsoids set at 50 % level and hydrogens omitted for clarity

CCDC Number	1864775
Empirical formula	$C_{10}H_{17}BF_2N_2O_2$
Formula weight	246.06
Temperature/K	150
Crystal system	monoclinic
Space group	$P2_1$
a/Å	6.6658(8)
b/Å	13.0852(10)
c/Å	7.7113(8)
α/°	90
β/°	112.529(13)
γ/°	90
Volume/Å^3	621.28(12)
Z	2
ρ_{calc}g/cm^3	1.315
μ/mm^{-1}	0.934
F(000)	260.0
Crystal size/mm^3	0.2 × 0.2 × 0.1
Radiation	CuKα (λ = 1.54184)
2Θ range for data collection/°	20.246 to 141.838
Index ranges	-8 ≤ h ≤ 7, -15 ≤ k ≤ 15, -9 ≤ l ≤ 9
Reflections collected	4616
Independent reflections	2294 [R_{int} = 0.0312, R_{sigma} = 0.0295]
Data/restraints/parameters	2294/1/158
Goodness-of-fit on F^2	1.079
Final R indexes [I>=2σ (I)]	R_1 = 0.0645, wR_2 = 0.1710
Final R indexes [all data]	R_1 = 0.0649, wR_2 = 0.1715
Largest diff. peak/hole / e Å^{-3}	0.41/-0.24
Flack parameter	-0.4(3)

Table S7: Table of crystallographic information for compound **4**

12.5 **IMe-BH$_2$-lactone 2i**

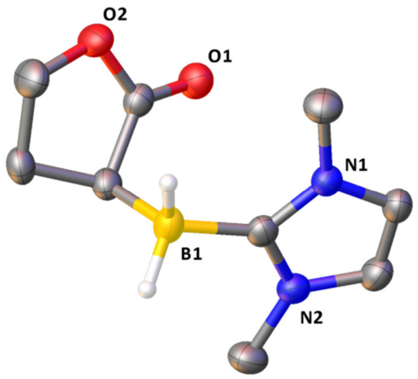

Figure S30: ORTEP plot for the molecular structure of compound **2i** with thermal ellipsoids set at 50 % level and most hydrogens omitted for clarity

CCDC Number	1864778
Empirical formula	$C_9H_{15}BN_2O_2$
Formula weight	194.04
Temperature/K	150.00(10)
Crystal system	monoclinic
Space group	$P2_1/n$
a/Å	8.6843(3)
b/Å	10.1231(2)
c/Å	12.0573(3)
α/°	90
β/°	100.885(3)
γ/°	90
Volume/Å^3	1040.91(5)
Z	4
ρ_{calc}g/cm^3	1.238
μ/mm^{-1}	0.697
F(000)	416.0
Crystal size/mm^3	0.08 × 0.06 × 0.001
Radiation	CuKα (λ = 1.54184)
2Θ range for data collection/°	11.5 to 141.936
Index ranges	-7 ≤ h ≤ 9, -12 ≤ k ≤ 12, -14 ≤ l ≤ 14
Reflections collected	8522
Independent reflections	1892 [R_{int} = 0.0243, R_{sigma} = 0.0216]
Data/restraints/parameters	1892/0/153
Goodness-of-fit on F^2	1.116
Final R indexes [I>=2σ (I)]	R_1 = 0.0583, wR_2 = 0.1531
Final R indexes [all data]	R_1 = 0.0614, wR_2 = 0.1562
Largest diff. peak/hole / e Å^{-3}	0.47/-0.19

Table S8: Table of crystallographic information for compound **2i**

12.6 IMe-BH$_2$-menthyl ester 2s

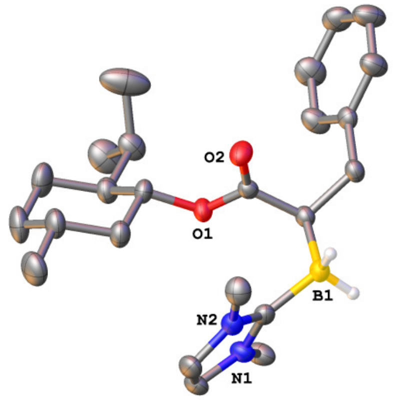

Figure S31: ORTEP plot for the molecular structure of compound **2s** with thermal ellipsoids set at 50 % level and hydrogens omitted for clarity

CCDC Number	1864779
Empirical formula	$C_{24}H_{37}BN_2O_2$
Formula weight	396.36
Temperature/K	149.99(10)
Crystal system	orthorhombic
Space group	$P2_12_12_1$
a/Å	11.9618(3)
b/Å	13.0959(3)
c/Å	15.6088(3)
α/°	90
β/°	90
γ/°	90
Volume/Å^3	2445.13(10)
Z	4
ρ_{calc}g/cm^3	1.077
μ/mm^{-1}	0.520
F(000)	864.0
Crystal size/mm^3	0.2 × 0.2 × 0.05
Radiation	CuKα (λ = 1.54184)
2Θ range for data collection/°	8.814 to 155.504
Index ranges	-15 ≤ h ≤ 14, -16 ≤ k ≤ 16, -19 ≤ l ≤ 19
Reflections collected	14093
Independent reflections	14093 [R_{int} = 0.0554 R_{sigma} = 0.0228]
Data/restraints/parameters	14093/0/268
Goodness-of-fit on F^2	1.072
Final R indexes [I>=2σ (I)]	R_1 = 0.0436, wR_2 = 0.1232
Final R indexes [all data]	R_1 = 0.0480, wR_2 = 0.1275
Largest diff. peak/hole / e Å^{-3}	0.17/-0.18
Flack parameter	0.38(14)

Table S9: Table of crystallographic information for compound **2s**

12.7 IMe-BH_2-8-phenyl menthyl ester **2t**

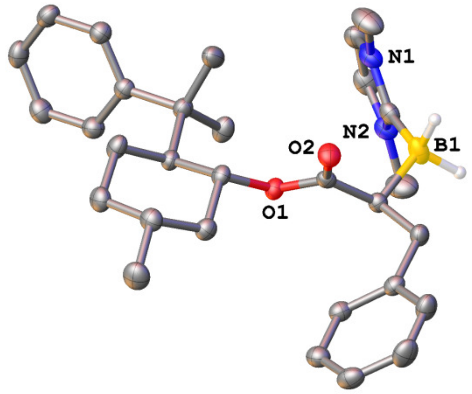

Figure S32: ORTEP plot for the molecular structure of compound **2t** with thermal ellipsoids set at 50 % level and most hydrogens omitted for clarity.

CCDC Number	1864776
Empirical formula	$C_{30}H_{41}BN_2O_2$
Formula weight	472.46
Temperature/K	149.9(2)
Crystal system	monoclinic
Space group	$P2_1$
a/Å	9.10720(10)
b/Å	15.5083(2)
c/Å	9.60940(10)
α/°	90
β/°	95.8720(10)
γ/°	90
Volume/Å^3	1350.08(3)
Z	2
ρ_{calc}g/cm^3	1.162
μ/mm^{-1}	0.551
F(000)	512.0
Crystal size/mm^3	0.05 × 0.05 × 0.05
Radiation	CuKα (λ = 1.54184)
2Θ range for data collection/°	9.252 to 141.89
Index ranges	-10 ≤ h ≤ 11, -18 ≤ k ≤ 18, -11 ≤ l ≤ 11
Reflections collected	13740
Independent reflections	5068 [R_{int} = 0.0245, R_{sigma} = 0.0278]
Data/restraints/parameters	5068/1/329
Goodness-of-fit on F^2	1.018
Final R indexes [I>=2σ (I)]	R_1 = 0.0284, wR_2 = 0.0704
Final R indexes [all data]	R_1 = 0.0299, wR_2 = 0.0712
Largest diff. peak/hole / e Å^{-3}	0.13/-0.14
Flack parameter	0.03(8)

Table S10: Table of crystallographic information for compound **2t**

.

13 NMR Spectra of Novel Compounds

13.1 IMe-BH$_2$-ester 2a

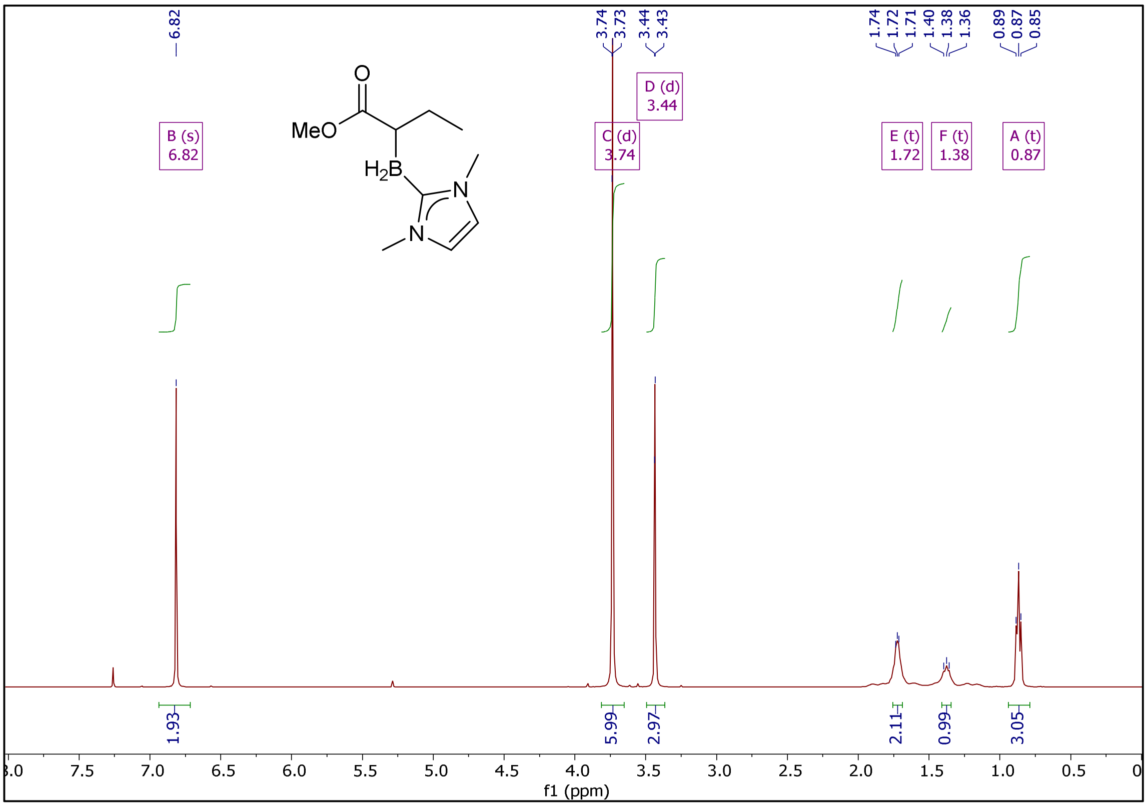

Figure S33: ^{1}H NMR spectrum of **2a** (CDCl$_3$)

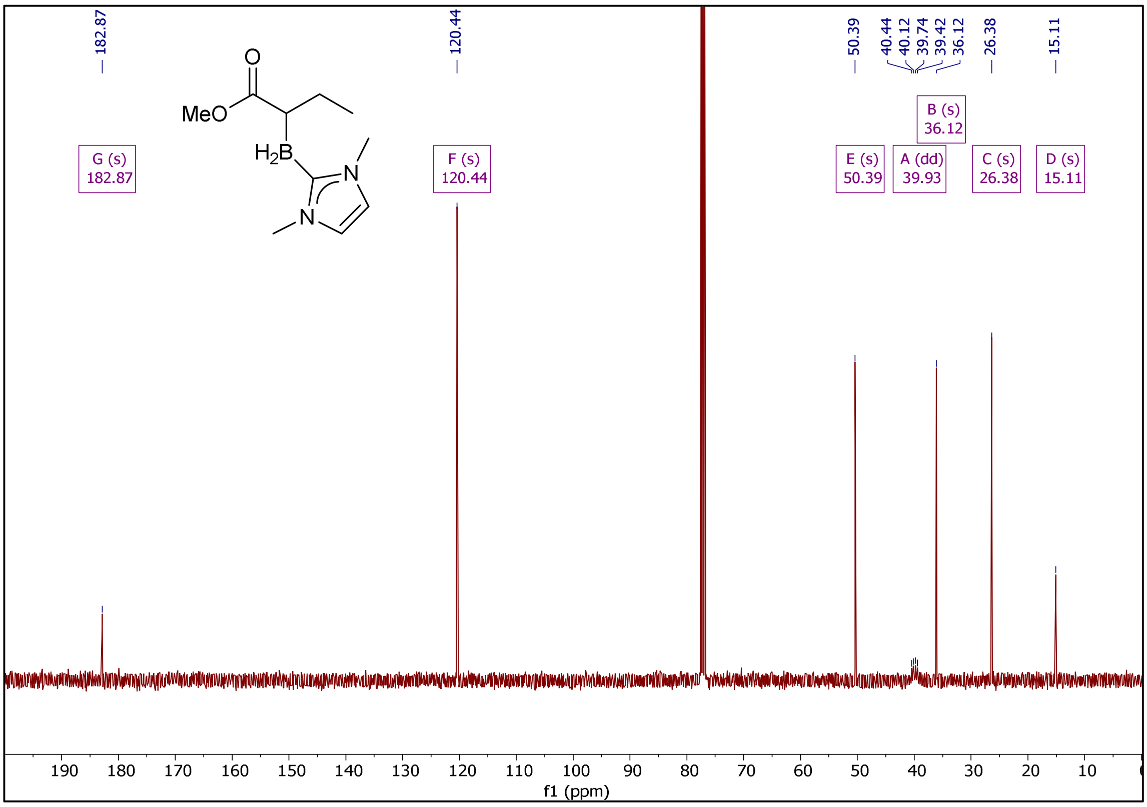

Figure S34: ^{13}C{^{1}H} NMR spectrum of **2a** (CDCl$_3$)

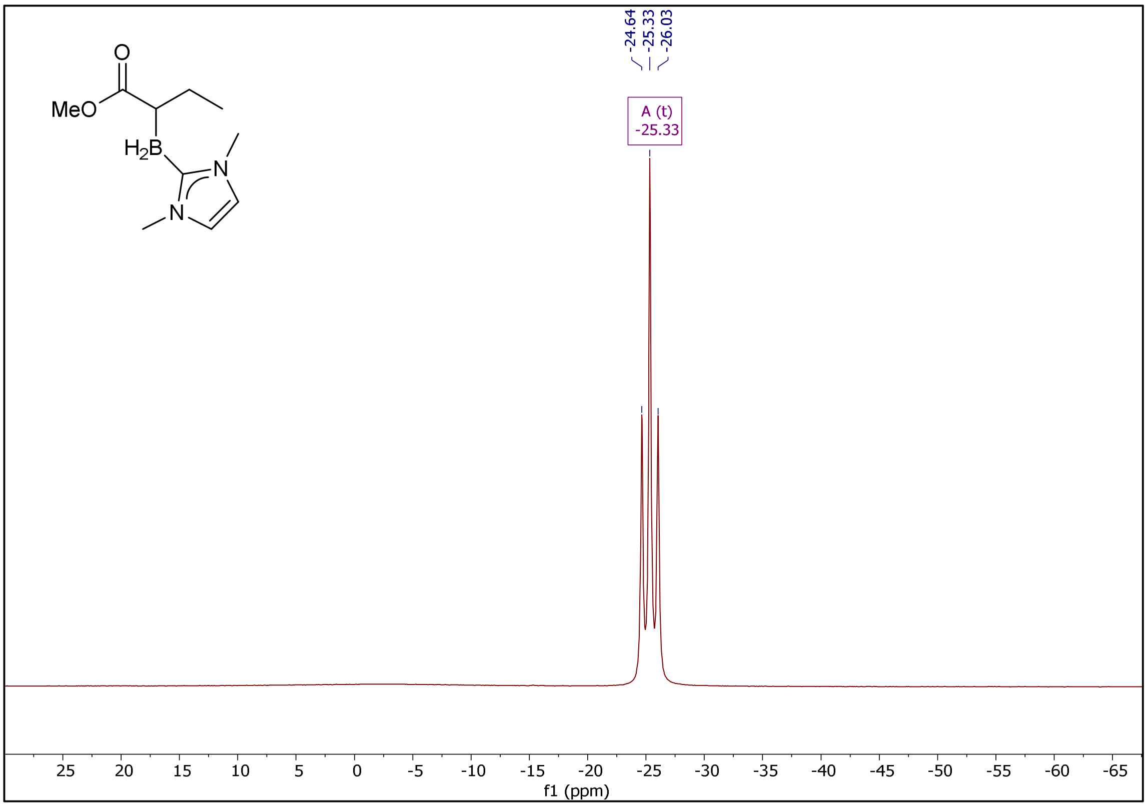

Figure S35: ^{11}B NMR spectrum of **2a** ($CDCl_3$)

13.2 IMe-BH$_2$-Phester 2k

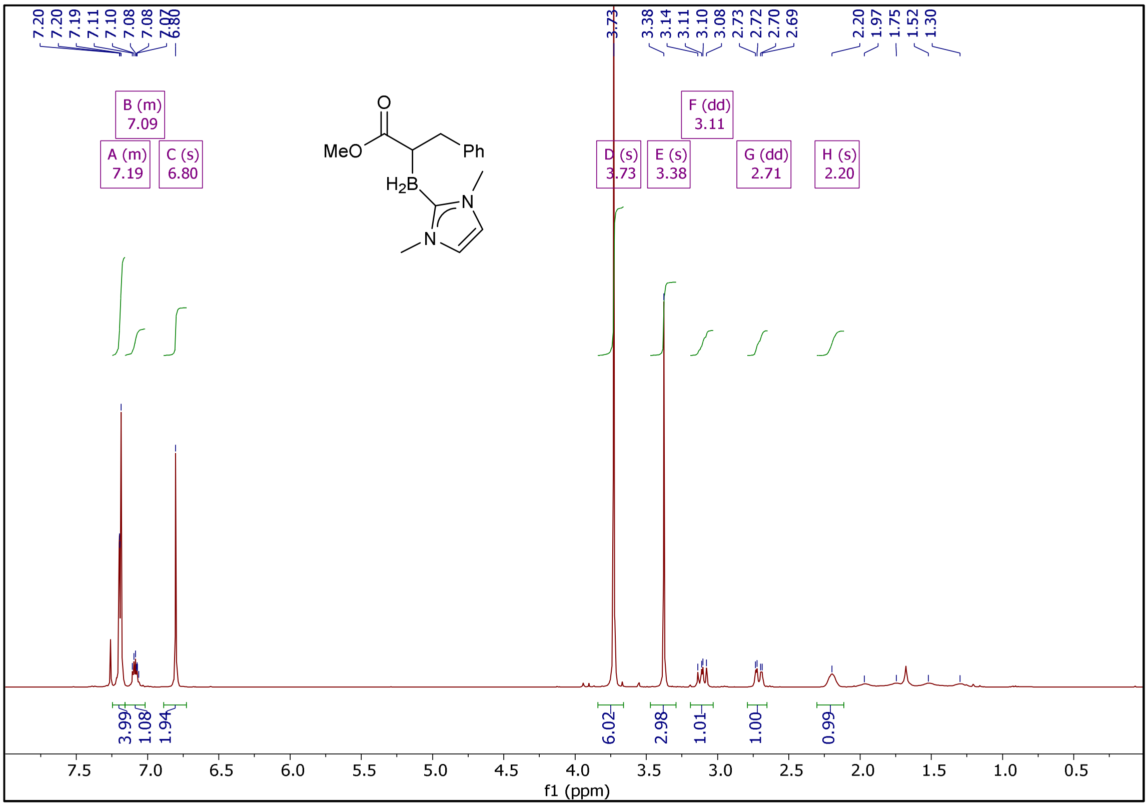

Figure S36: ^{1}H NMR spectrum of **2k** ($CDCl_3$)

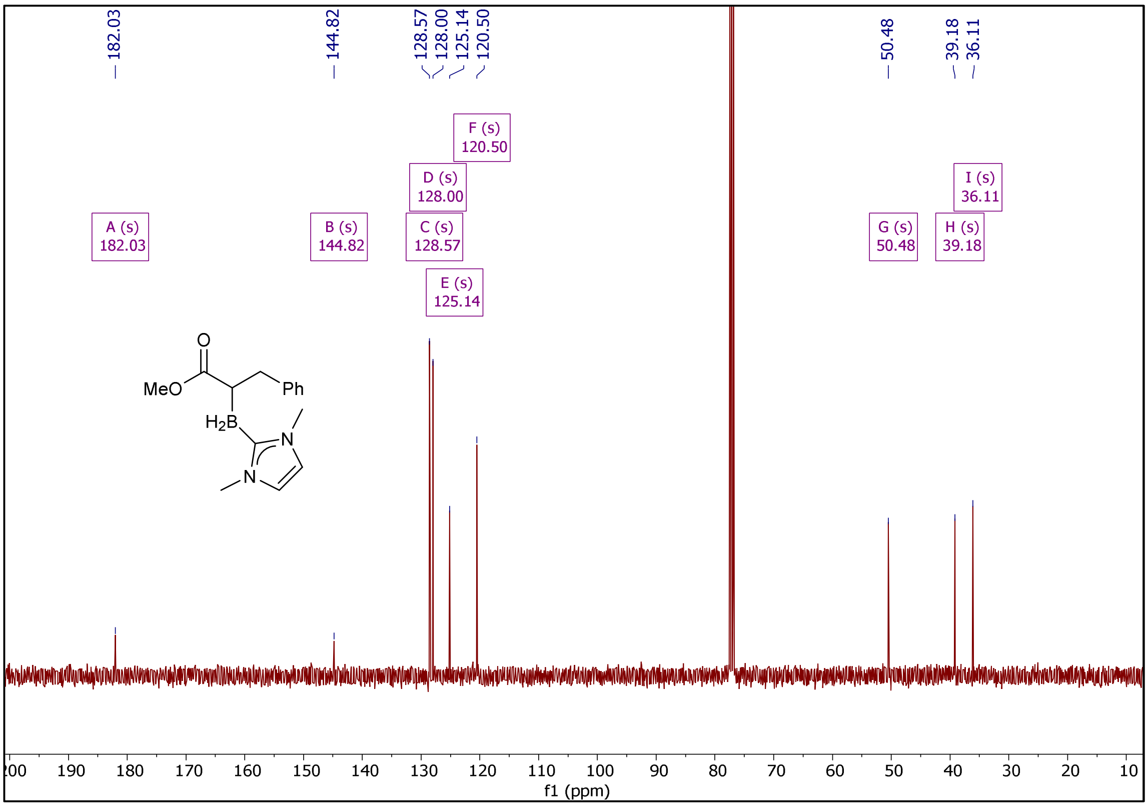

Figure S37: $^{13}C\{^{1}H\}$ NMR spectrum of **2k** ($CDCl_3$)

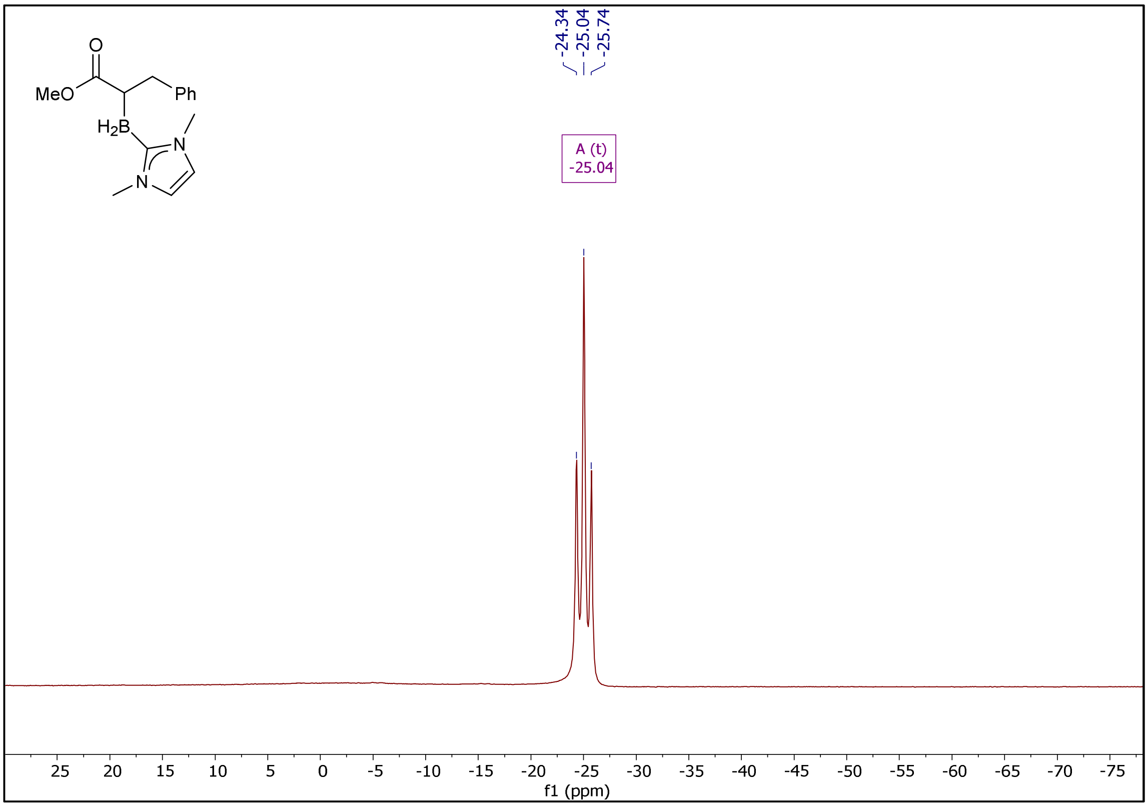

Figure S38: ^{11}B NMR spectrum of **2k** ($CDCl_3$)

13.3 BenzIMe-BH_2-ester 4a

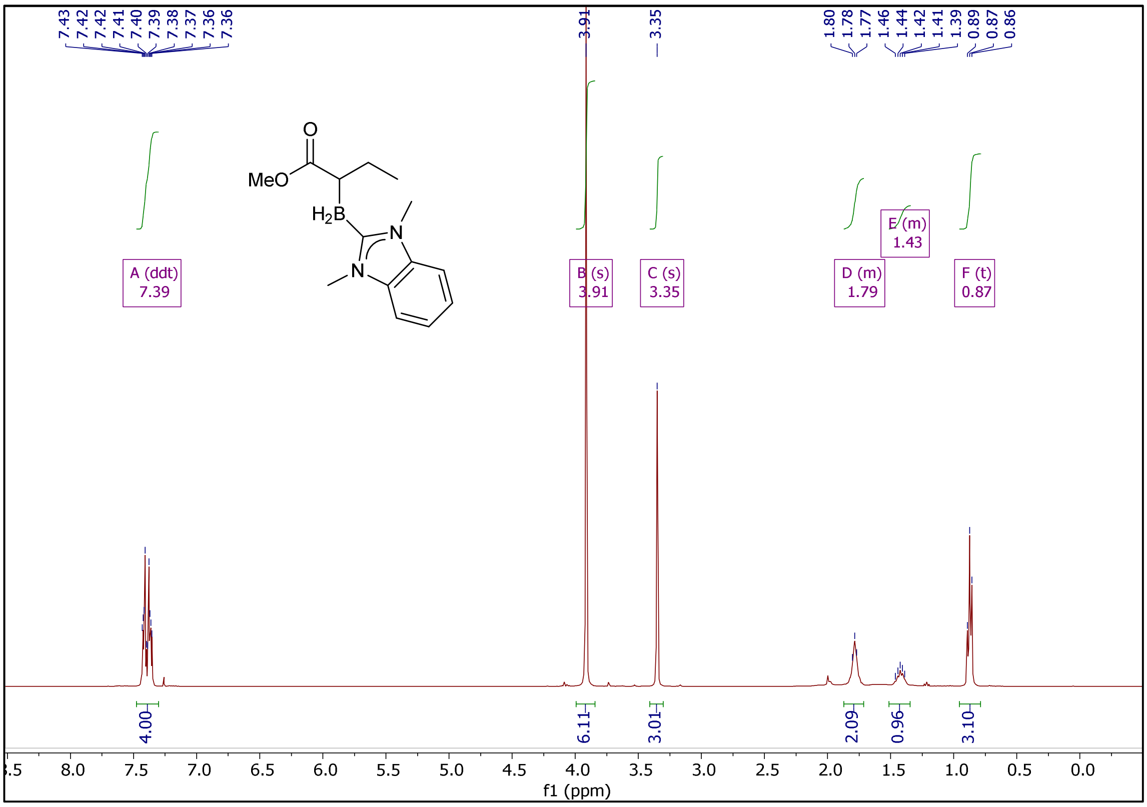

Figure S39: ^{1}H NMR spectrum of **4a** ($CDCl_3$)

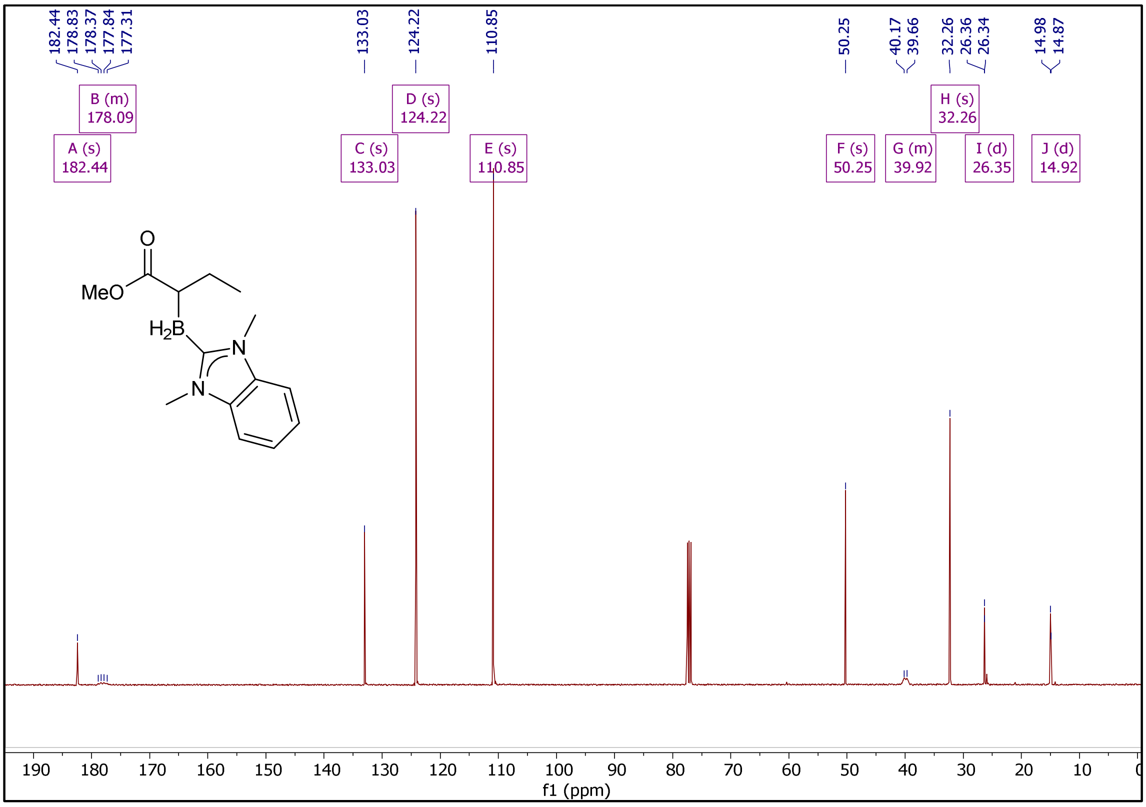

Figure S40: $^{13}C\{^1H\}$ NMR spectrum of **4a** ($CDCl_3$)

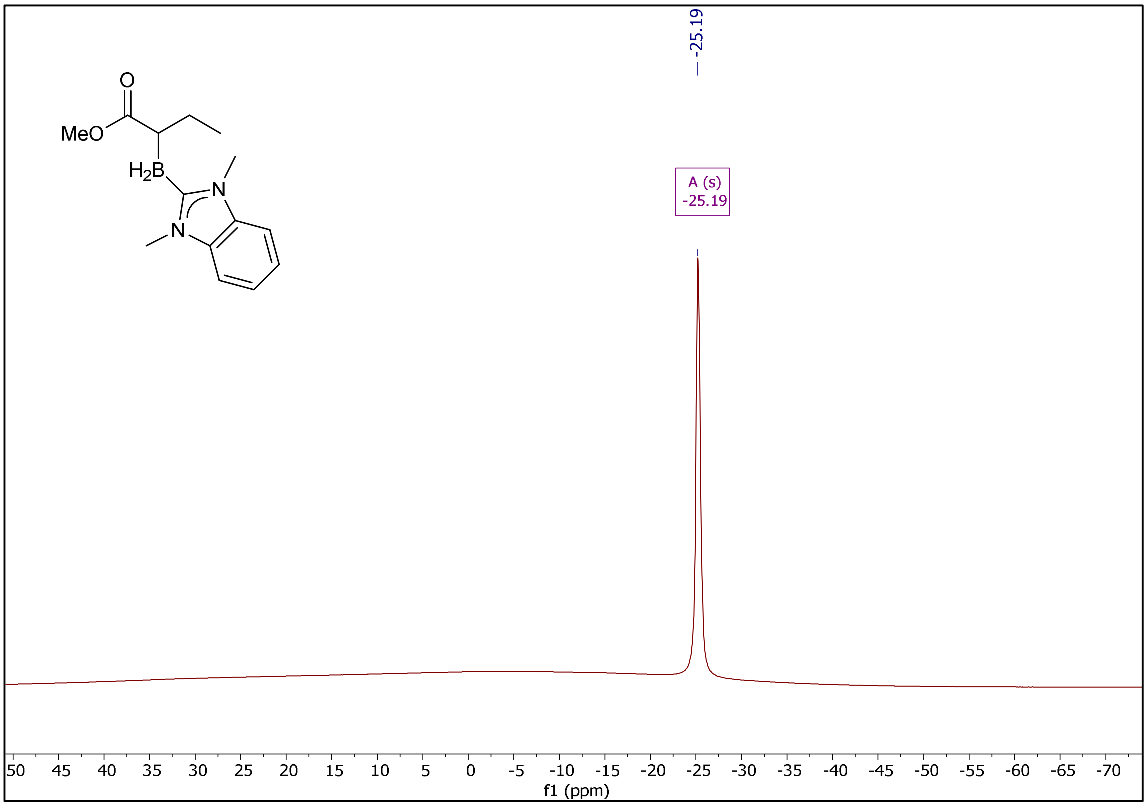

Figure S41: $^{11}B\{^1H\}$ NMR spectrum of **4a** ($CDCl_3$)

13.4 **IMe-BD$_2$-lactone d$_3$-2i**

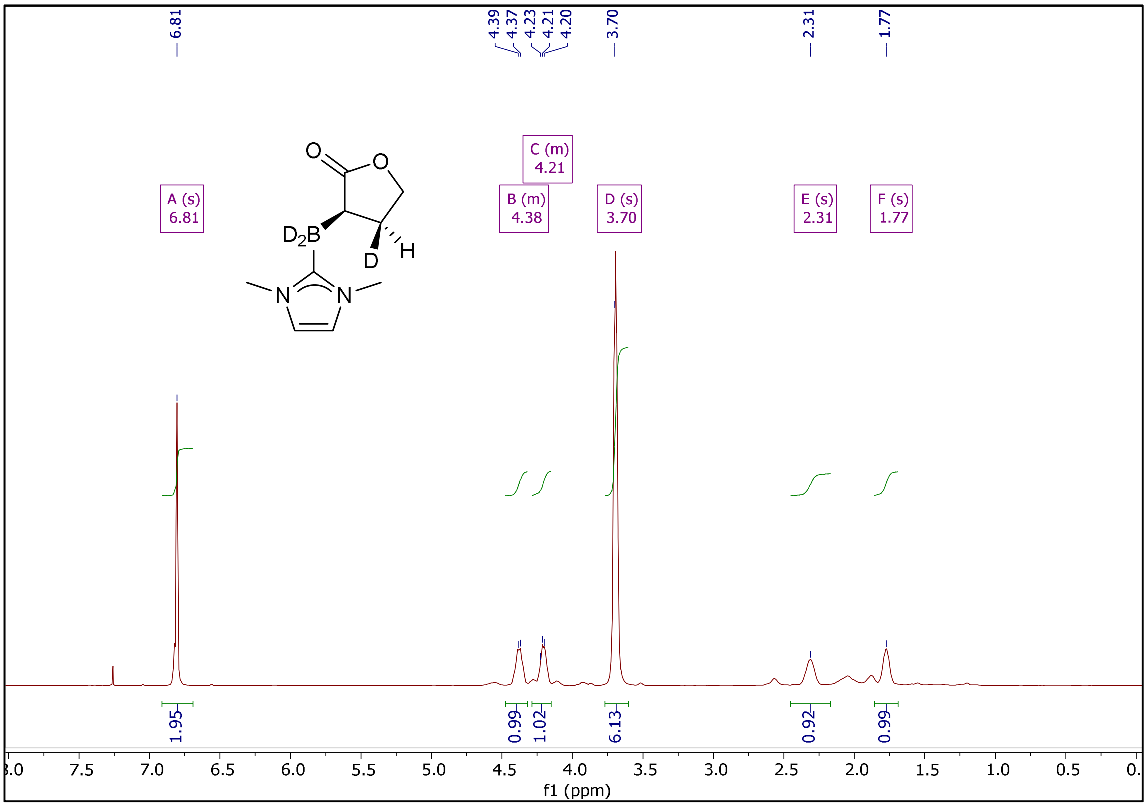

Figure S42: $^{1}H\{^{11}B\}$ NMR spectrum of **d$_3$-2i** ($CDCl_3$)

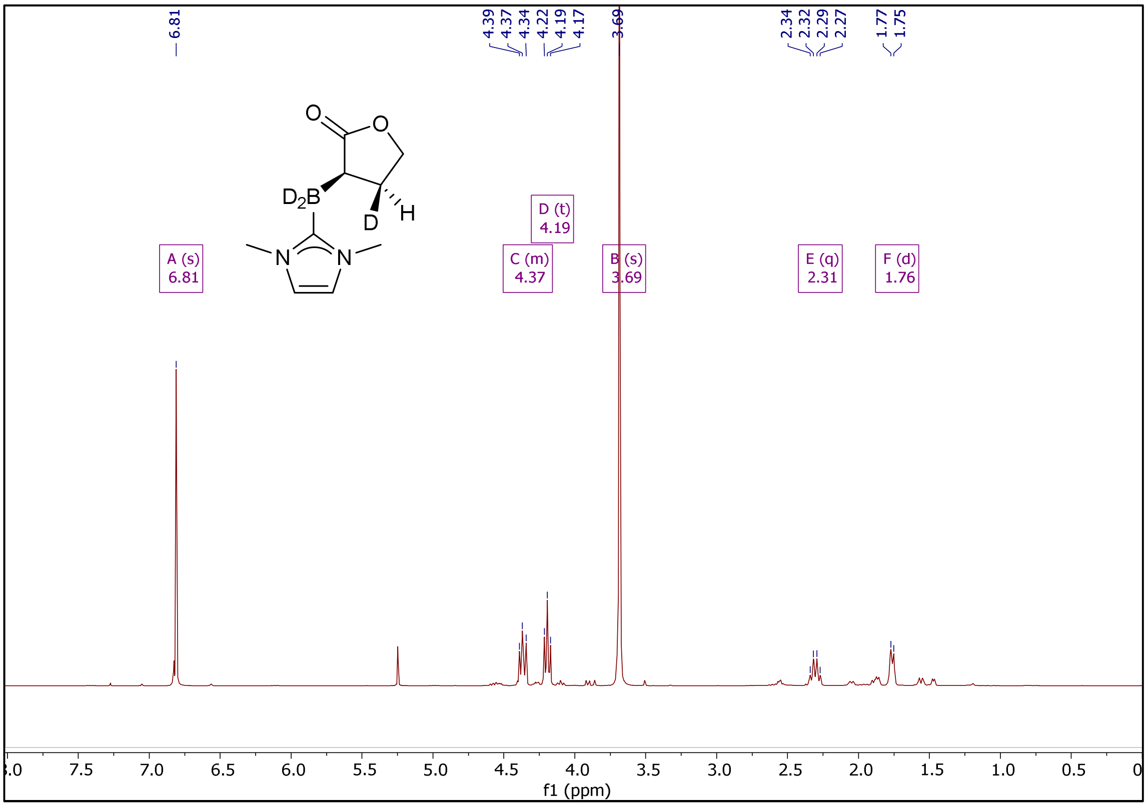

Figure S43: ^{1}H NMR spectrum of **d$_3$-2i** ($CDCl_3$)

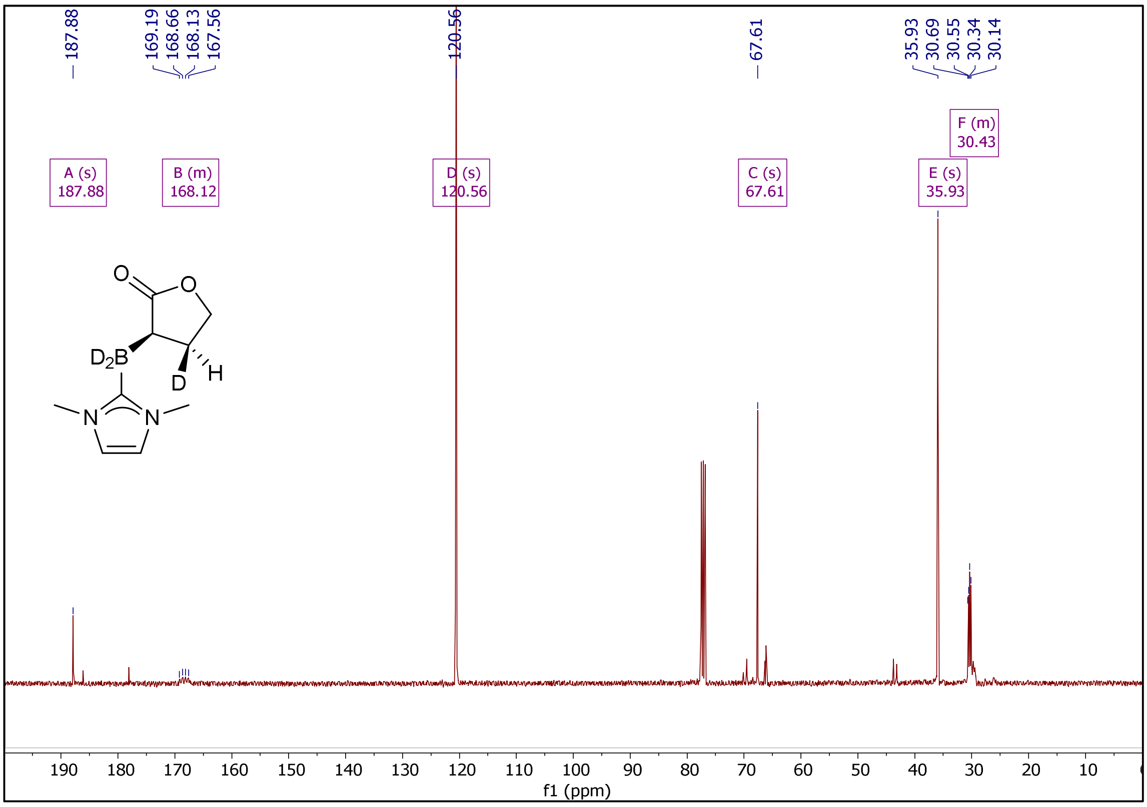

Figure S44: $^{13}C\{^{1}H\}$ NMR spectrum of **d$_3$-2i** ($CDCl_3$)

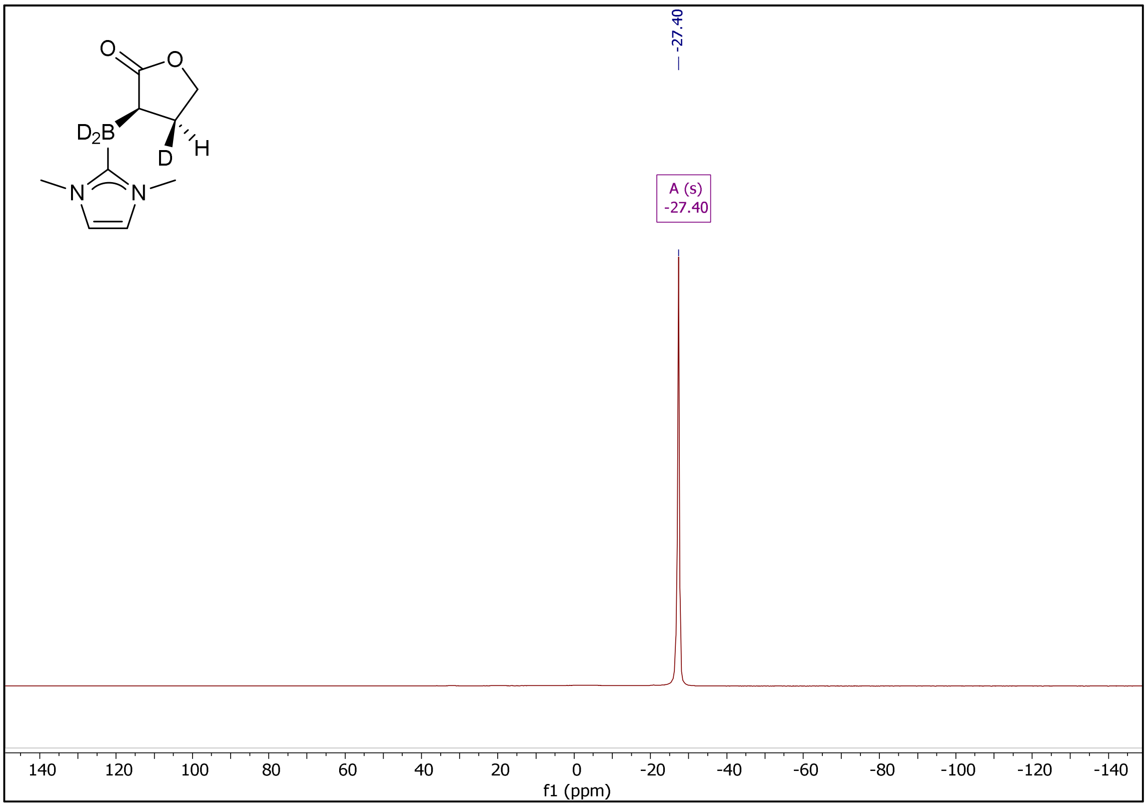

Figure S45: ^{11}B NMR spectrum of **d$_3$-2i** ($CDCl_3$)

13.5 **IMe-BF$_2$-ester 6**

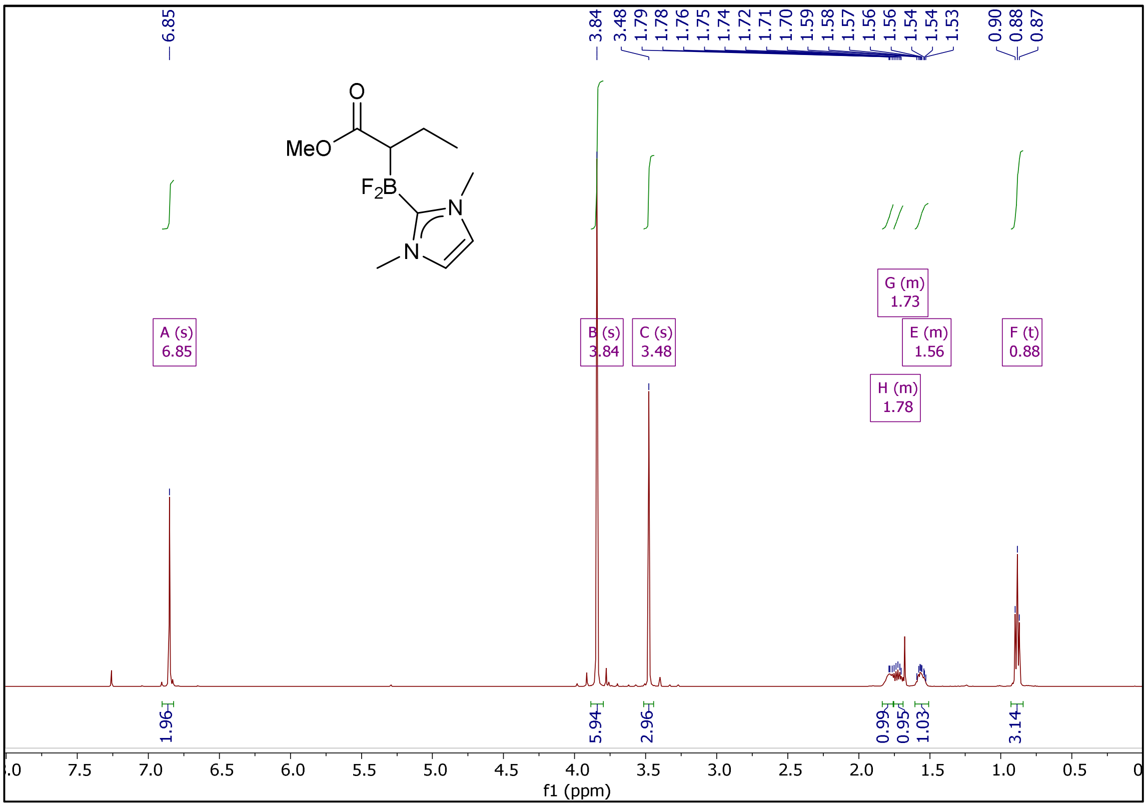

Figure S46: ^{1}H NMR spectrum of **6** (CDCl$_3$)

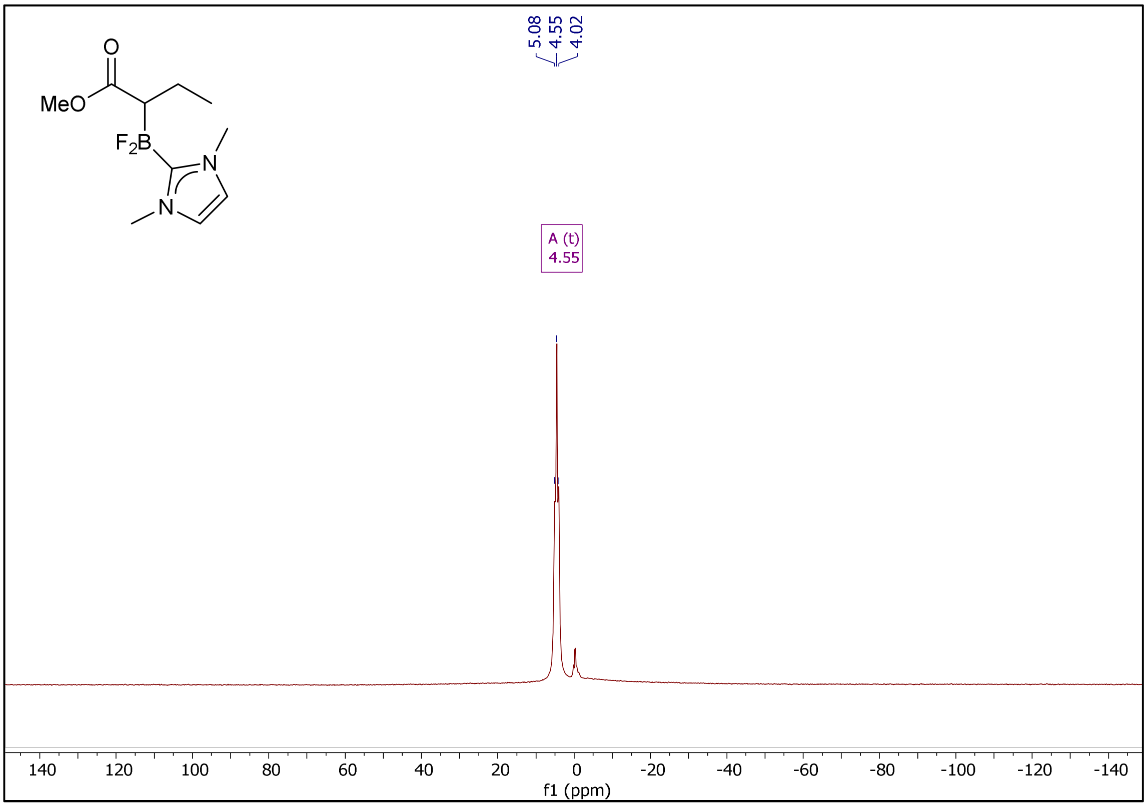

Figure S47: ^{11}B NMR spectrum of **6** (CDCl$_3$)

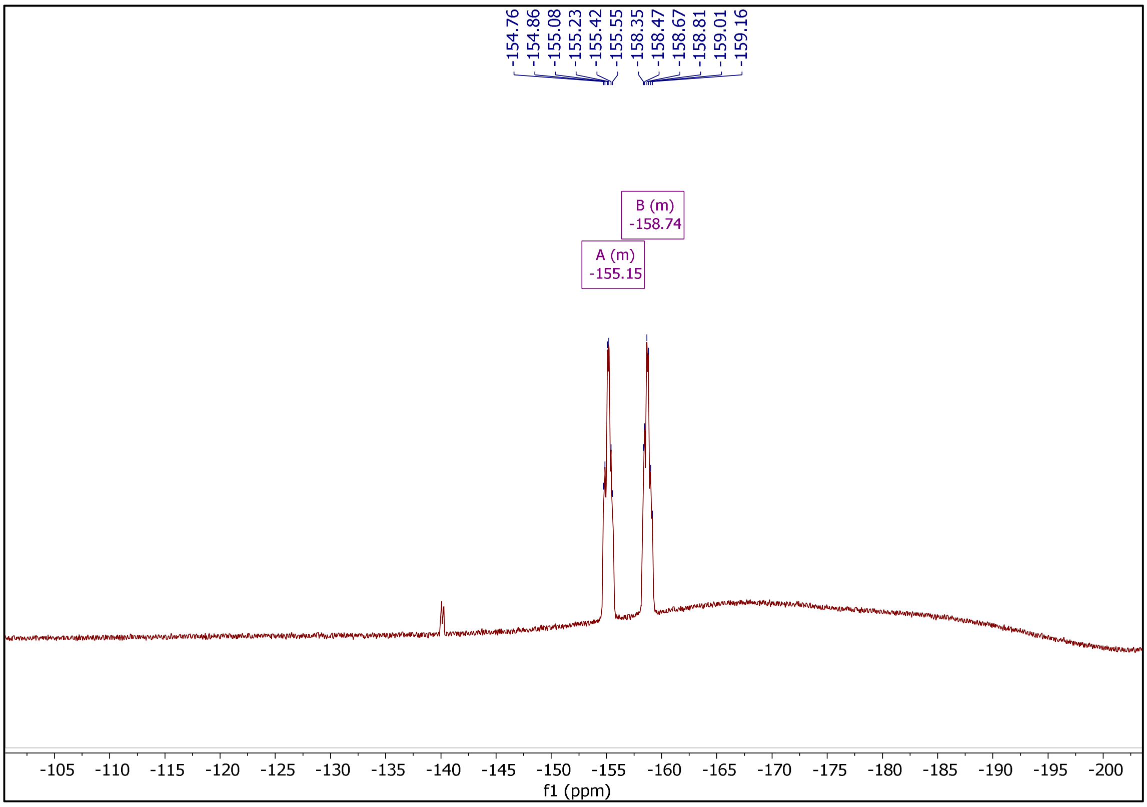

Figure S48: $^{19}F\{^{1}H\}$ NMR spectrum of **6** ($CDCl_3$)

13.6 IMe-BCl_2-ester 7

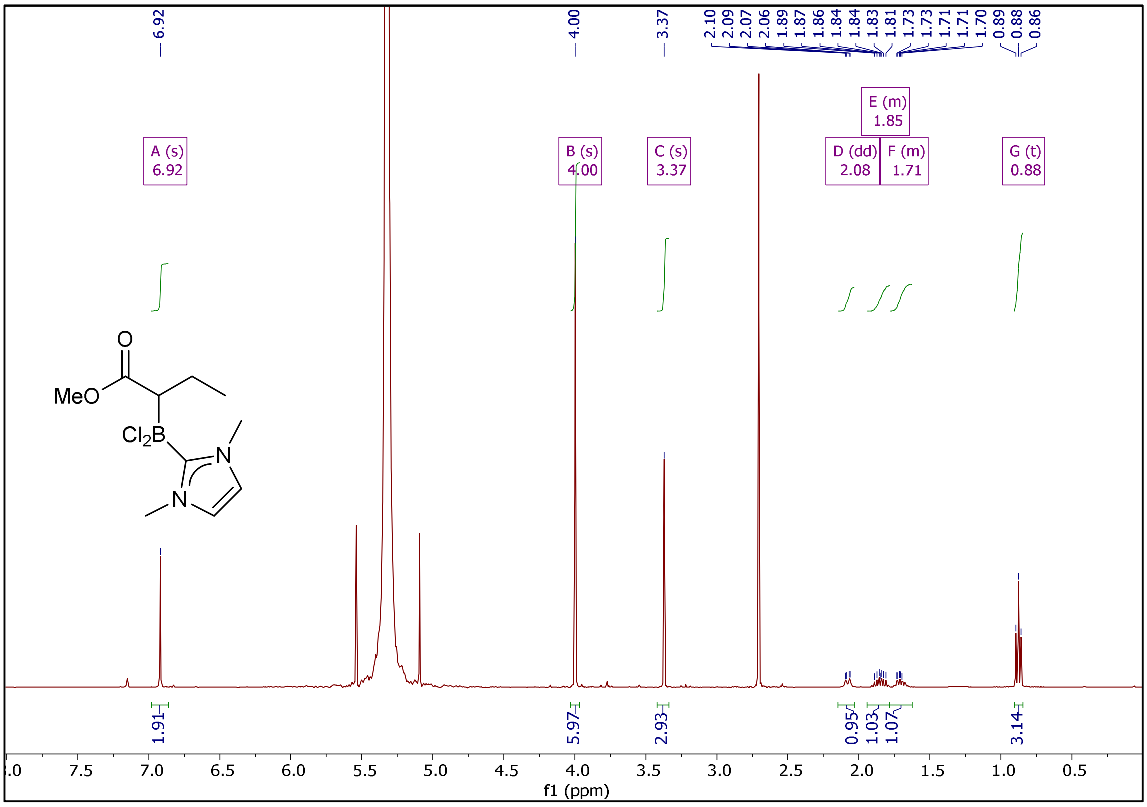

Figure S49: In-situ ^{1}H NMR spectrum of **7** ($CDCl_3$) from the reaction with NCS (succinimide by-product also present)

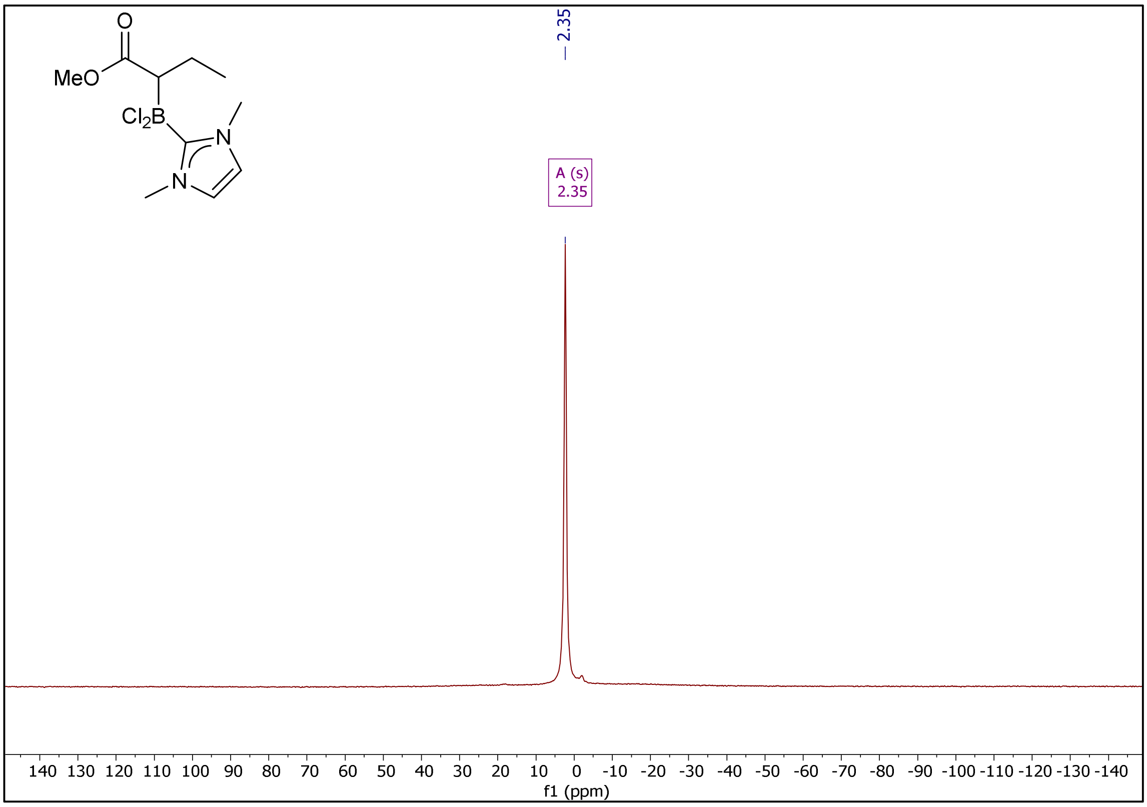

Figure S50: In-situ ^{11}B NMR spectrum of **7** ($CDCl_3$)

13.7 IMe-BH$_2$-menthyl ester 2s

13.7.1 Compound 2s major diastereomer

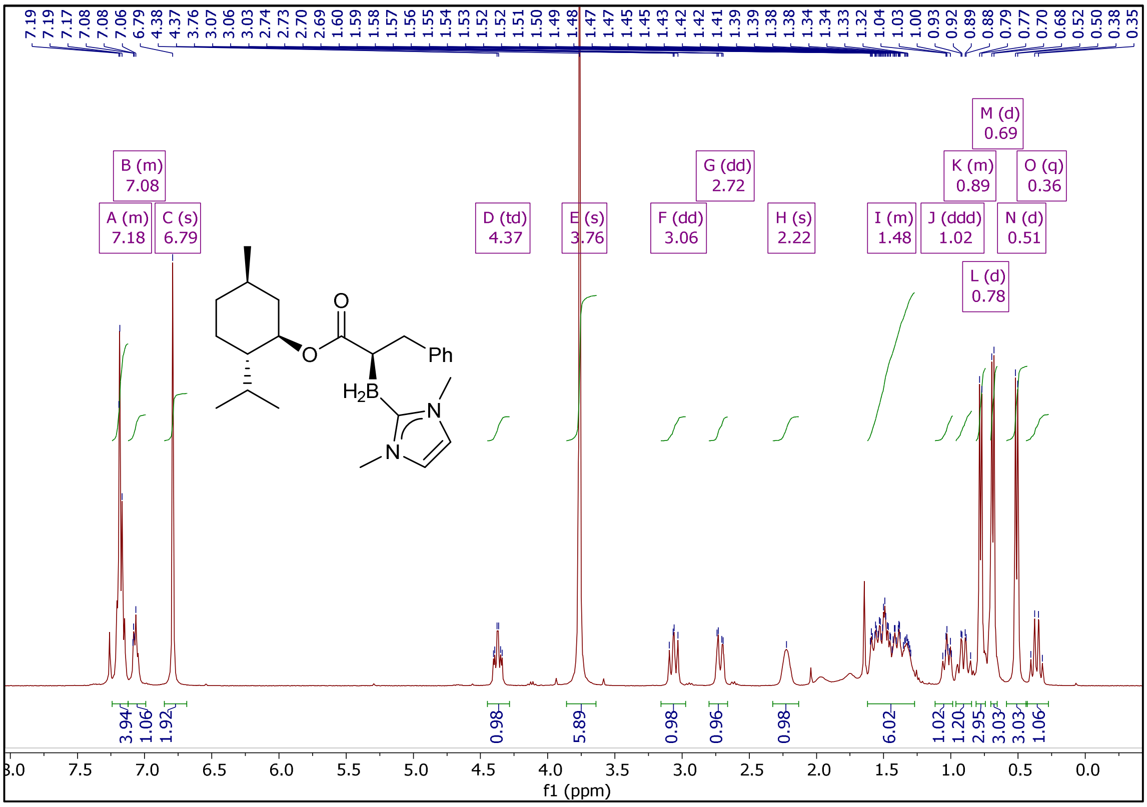

Figure S51: ^{1}H NMR spectrum of **2s** (CDCl$_3$)

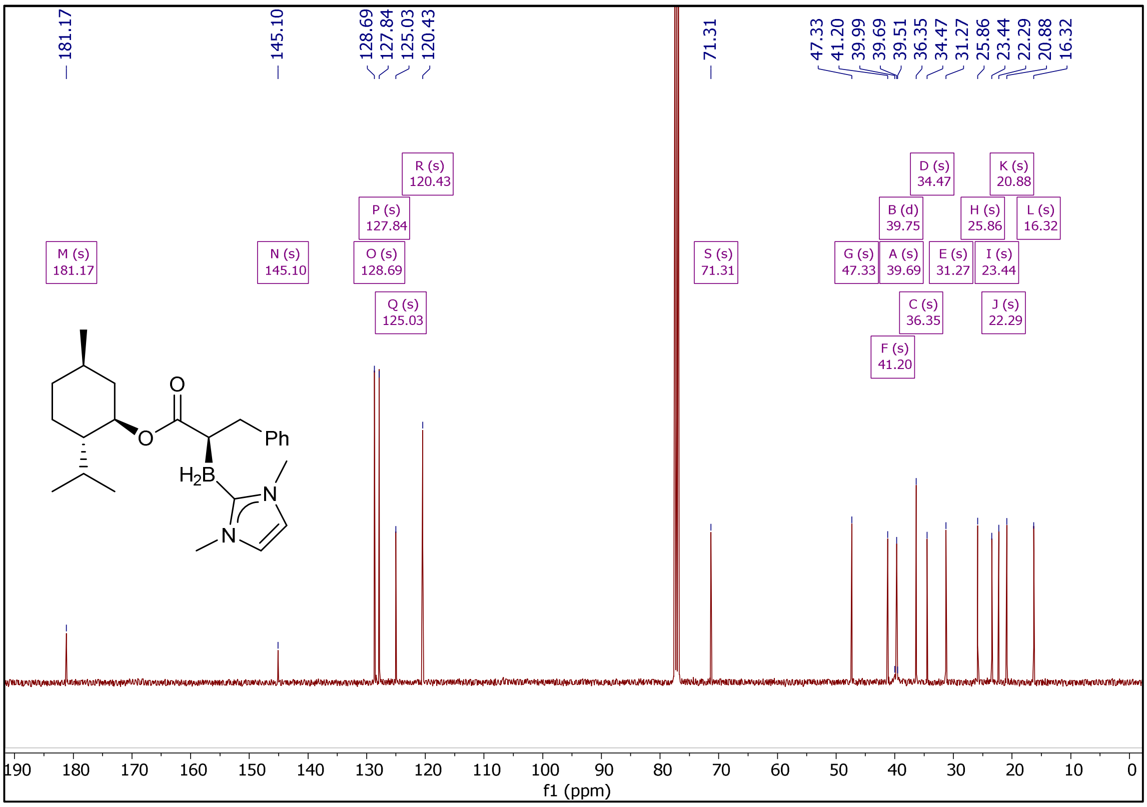

Figure S52: ^{13}C{^{1}H} NMR spectrum of **2s** (CDCl$_3$)

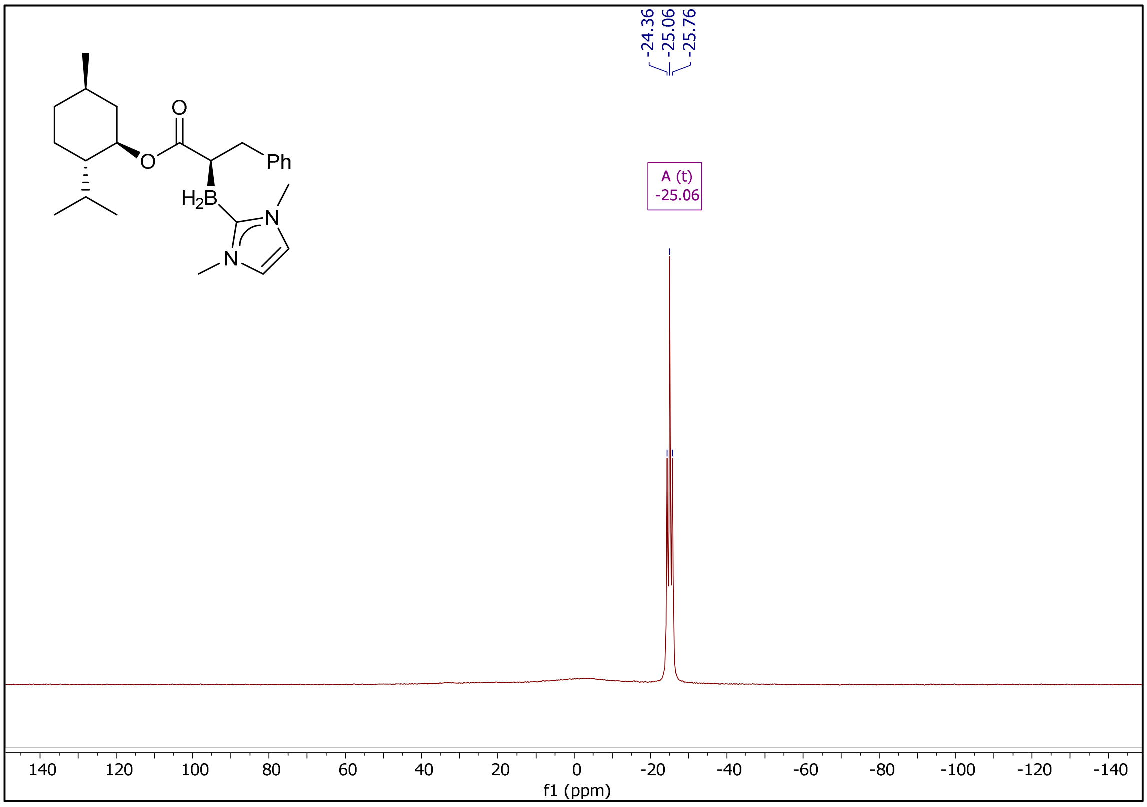

Figure S53: ^{11}B NMR spectrum of **2s** ($CDCl_3$)

13.7.2 Compound 2s minor diastereomer

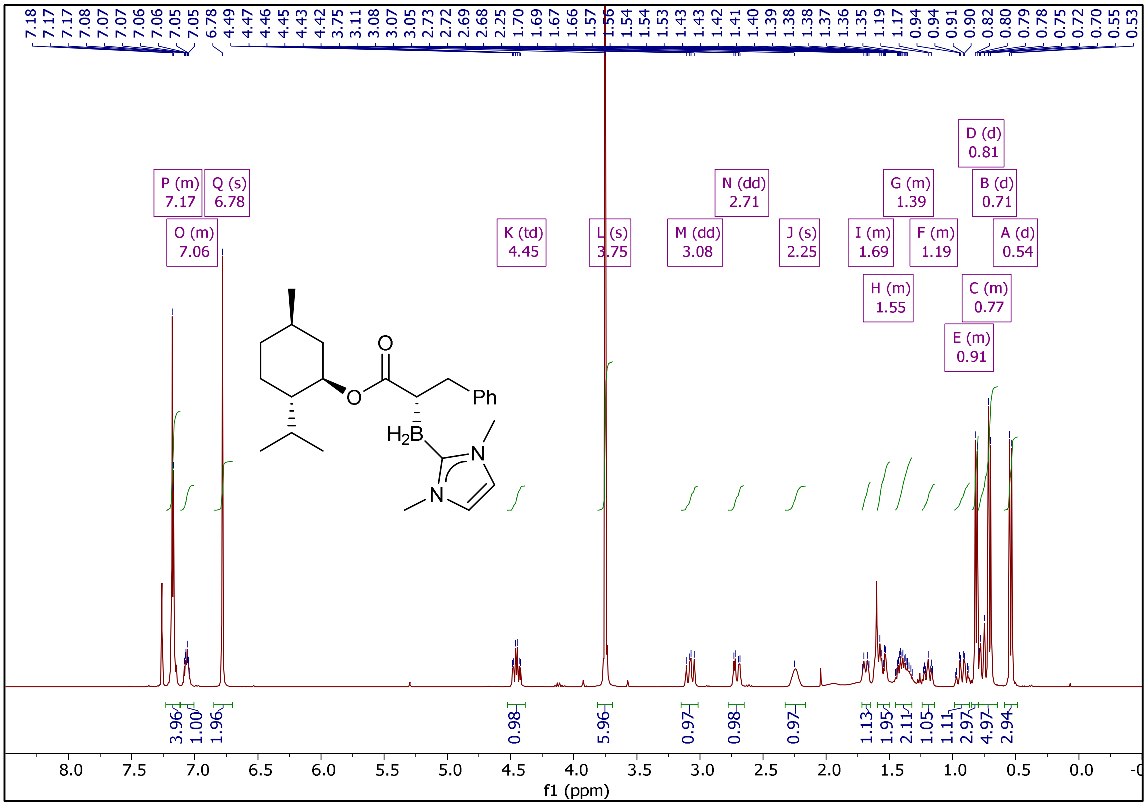

Figure S54: ^{1}H NMR spectrum of **2s** ($CDCl_3$)

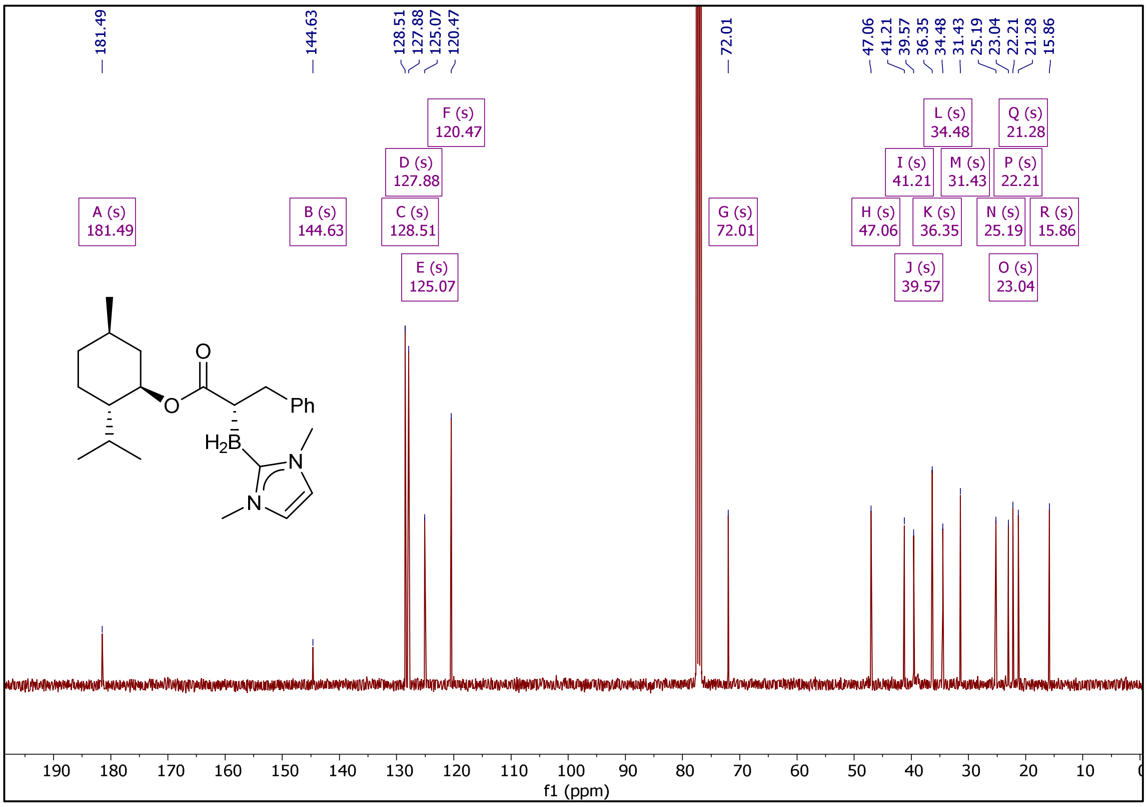

Figure S55: $^{13}C\{^{1}H\}$ NMR spectrum of **2s** ($CDCl_3$)

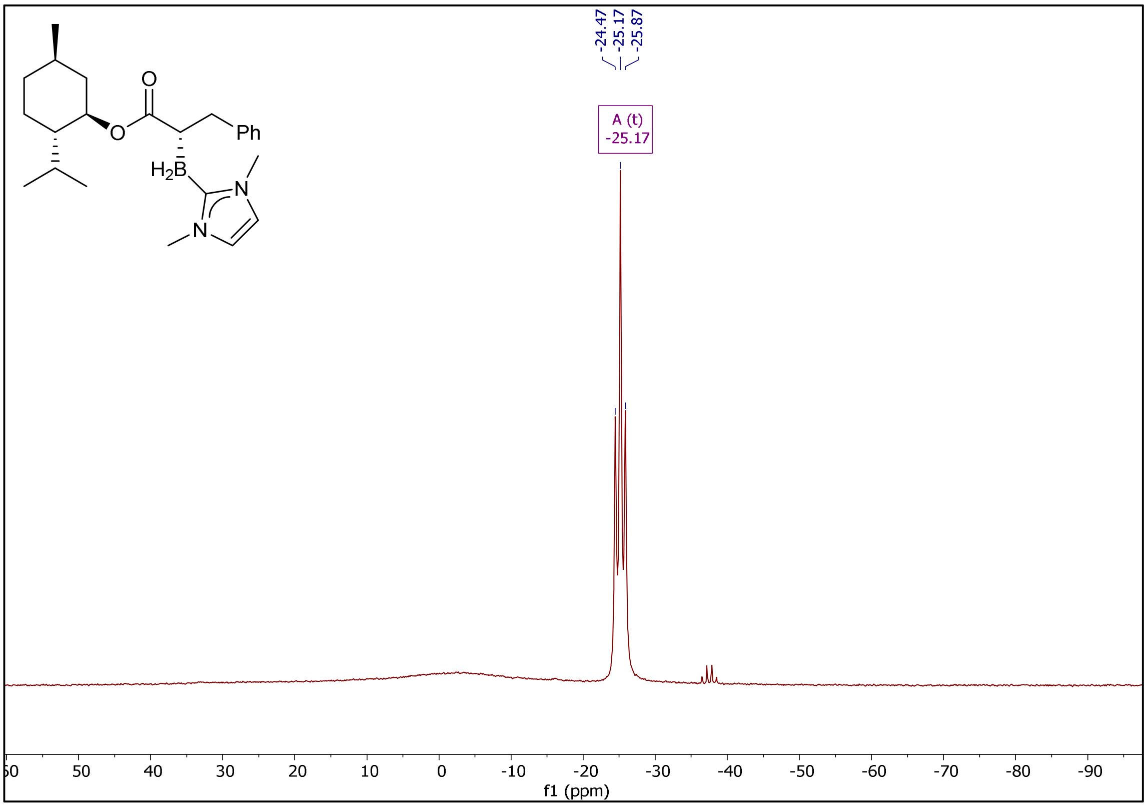

Figure S56: $^{11}B\{$ NMR spectrum of **2s** ($CDCl_3$)

13.8 IMe-BH$_2$-8-phenyl menthyl ester

13.8.1 Compound 2t major diastereomer

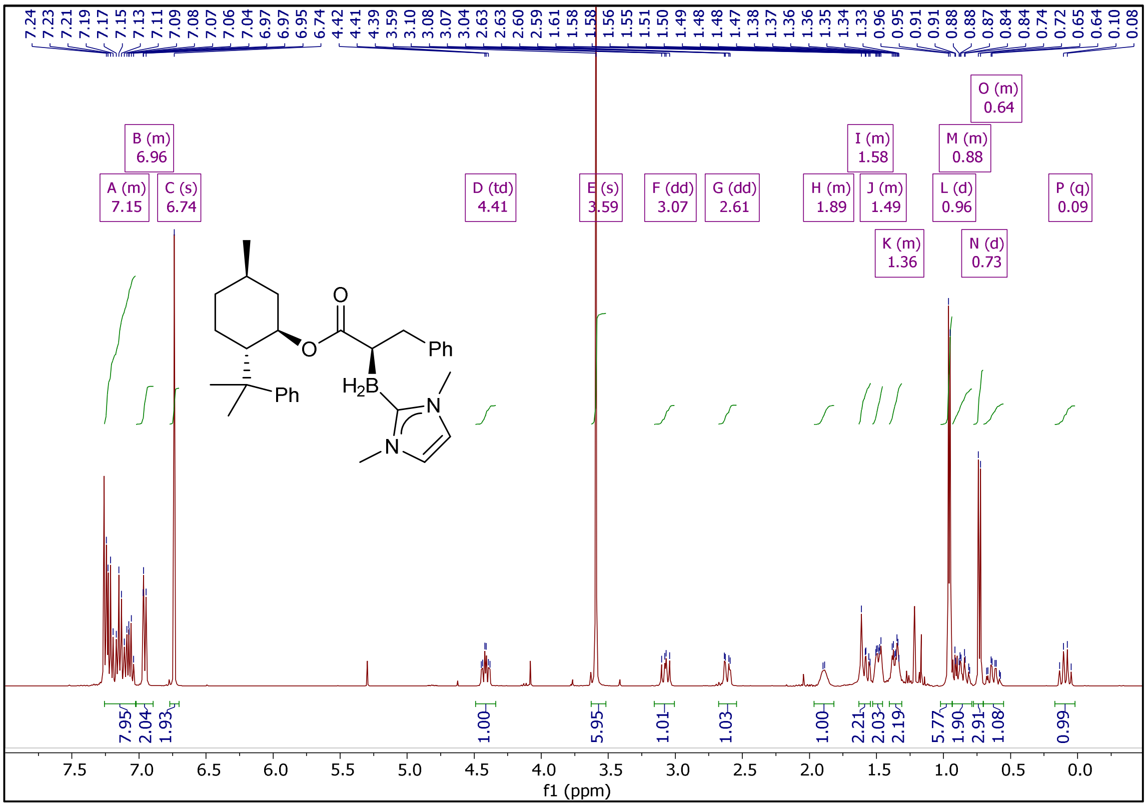

Figure S57: ^{1}H NMR spectrum of **2t** ($CDCl_3$)

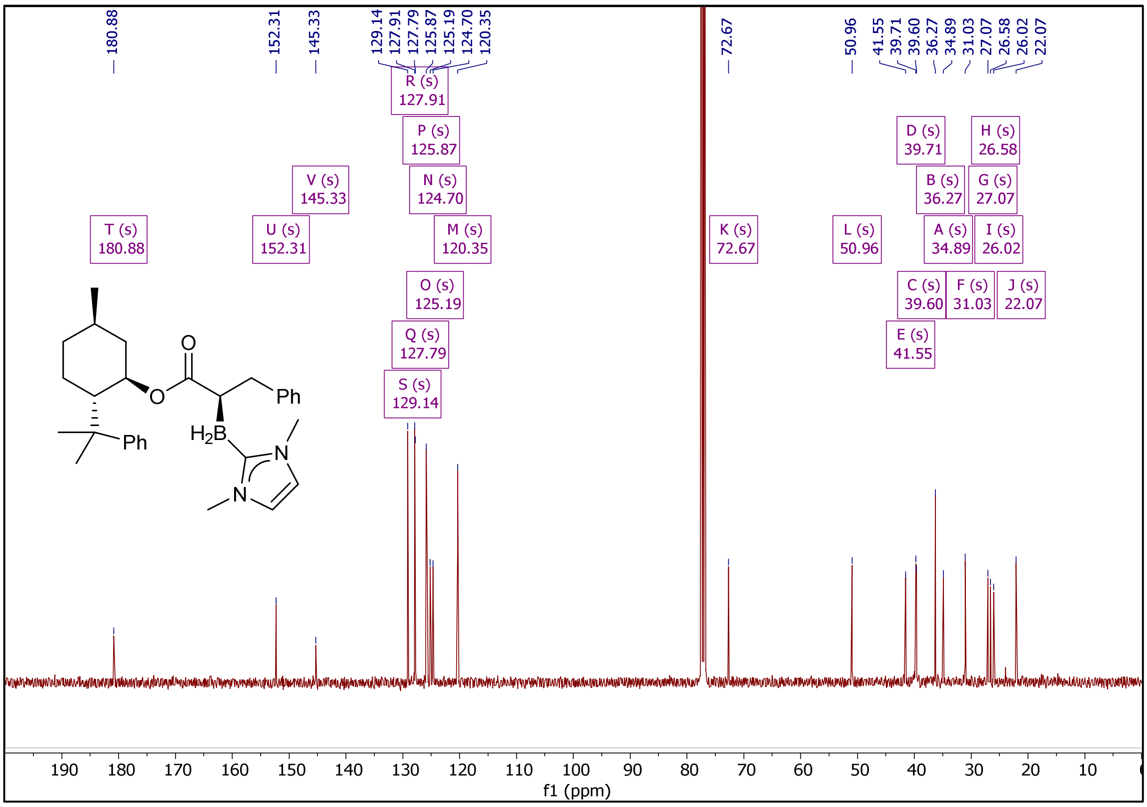

Figure S58: $^{13}C\{^{1}H\}$ NMR spectrum of **2t** ($CDCl_3$)

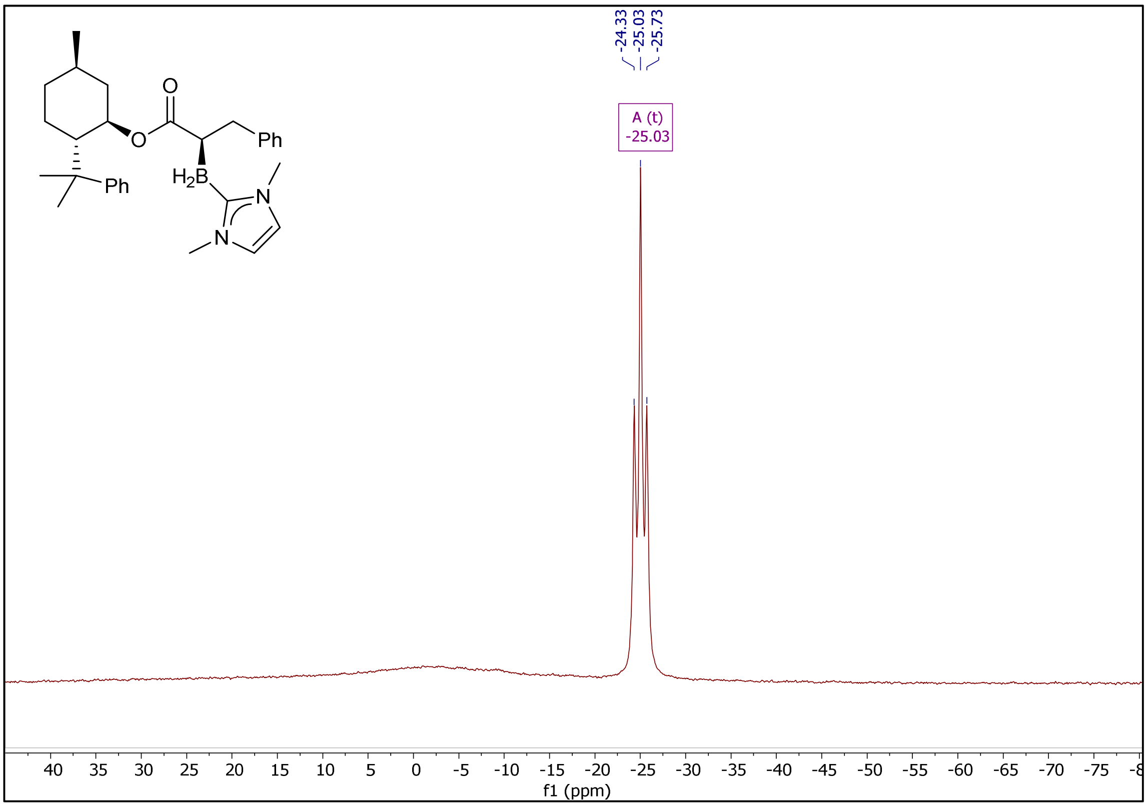

Figure S59: ^{113}B NMR spectrum of **2t** ($CDCl_3$)

13.8.2 Compound 2t minor diastereomer

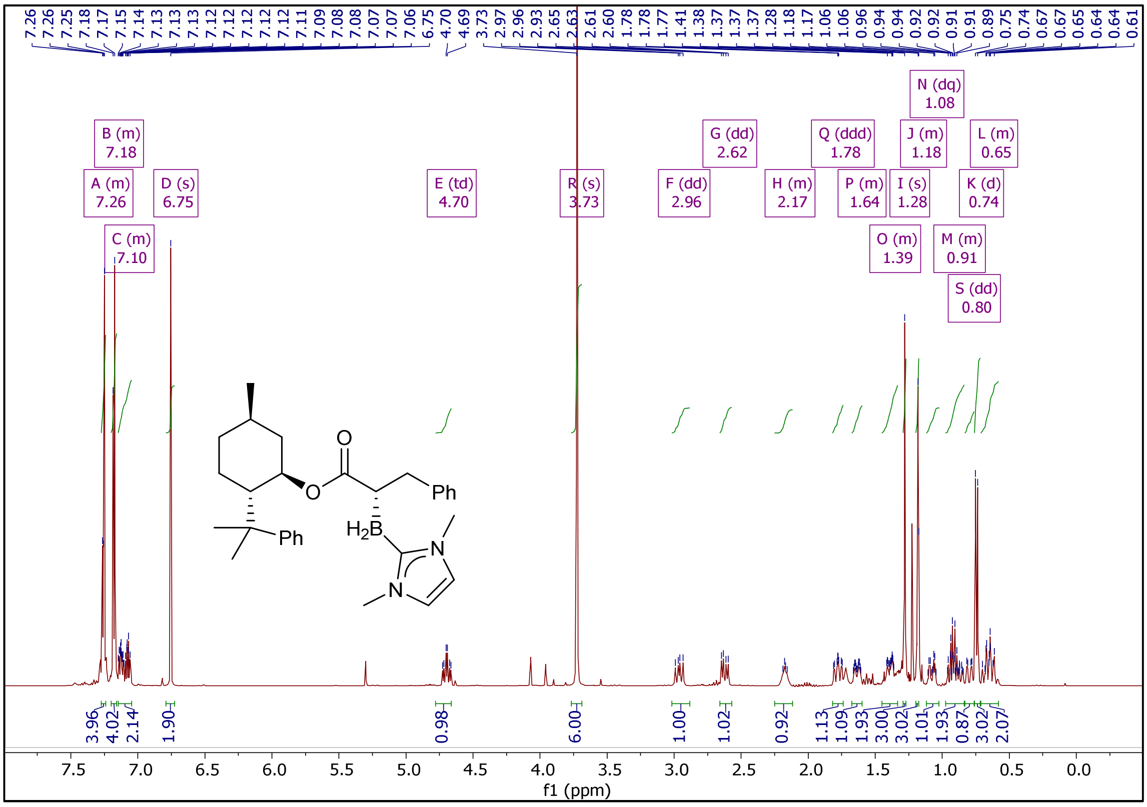

Figure S60: ^{1}H NMR spectrum of **2t** ($CDCl_3$)

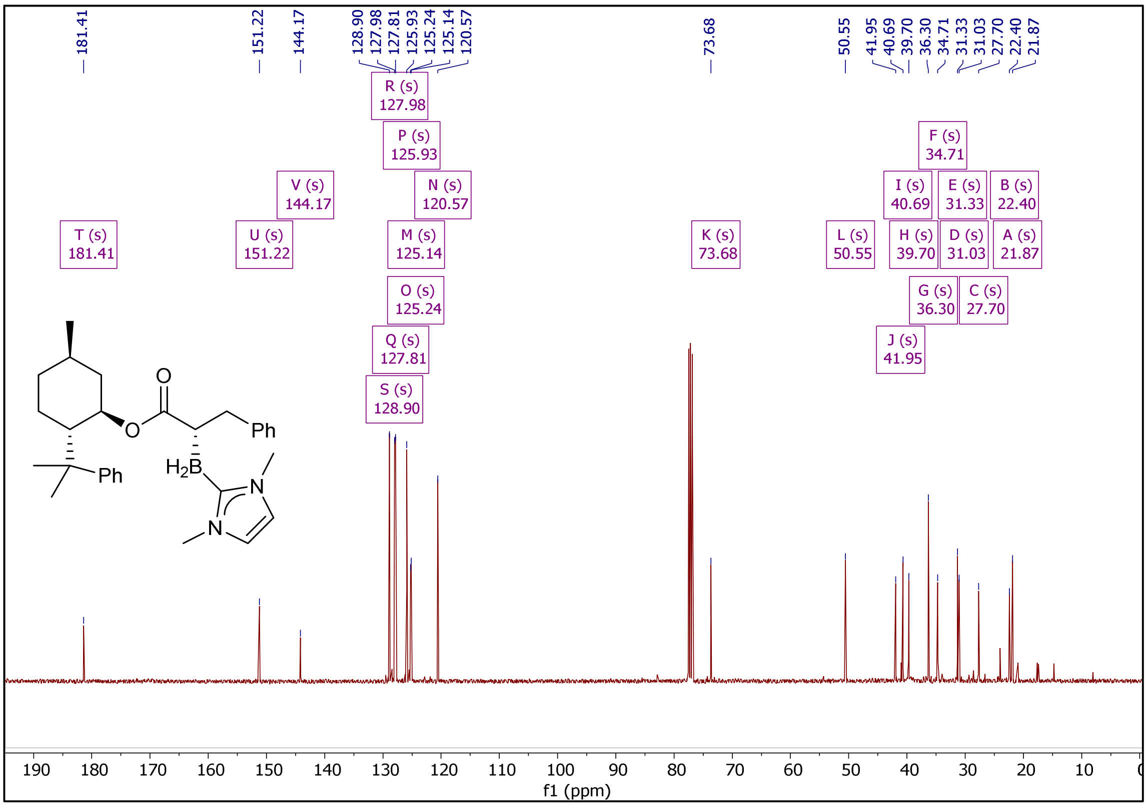

Figure S61: $^{13}C\{^{1}H\}$ NMR spectrum of **2t** ($CDCl_3$)

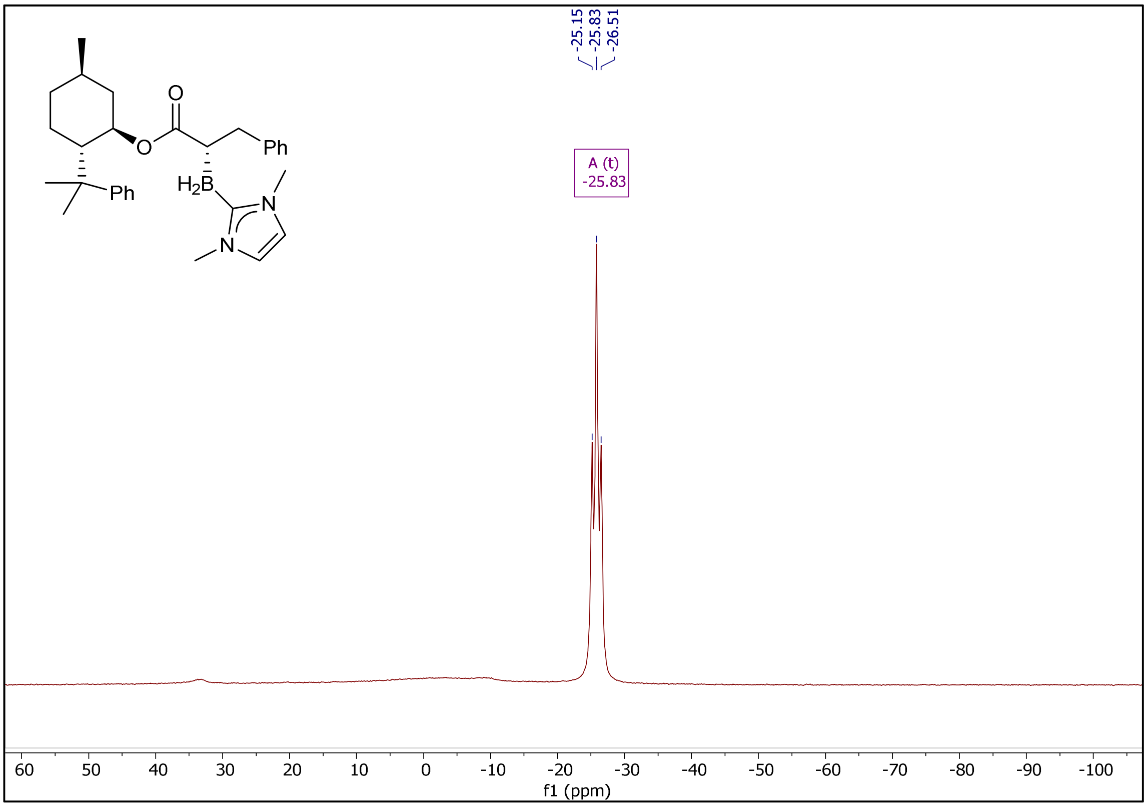

Figure S62: ^{11}B NMR spectrum of **2t** ($CDCl_3$)

13.9 **IMe-BH$_2$-alcohol 8**

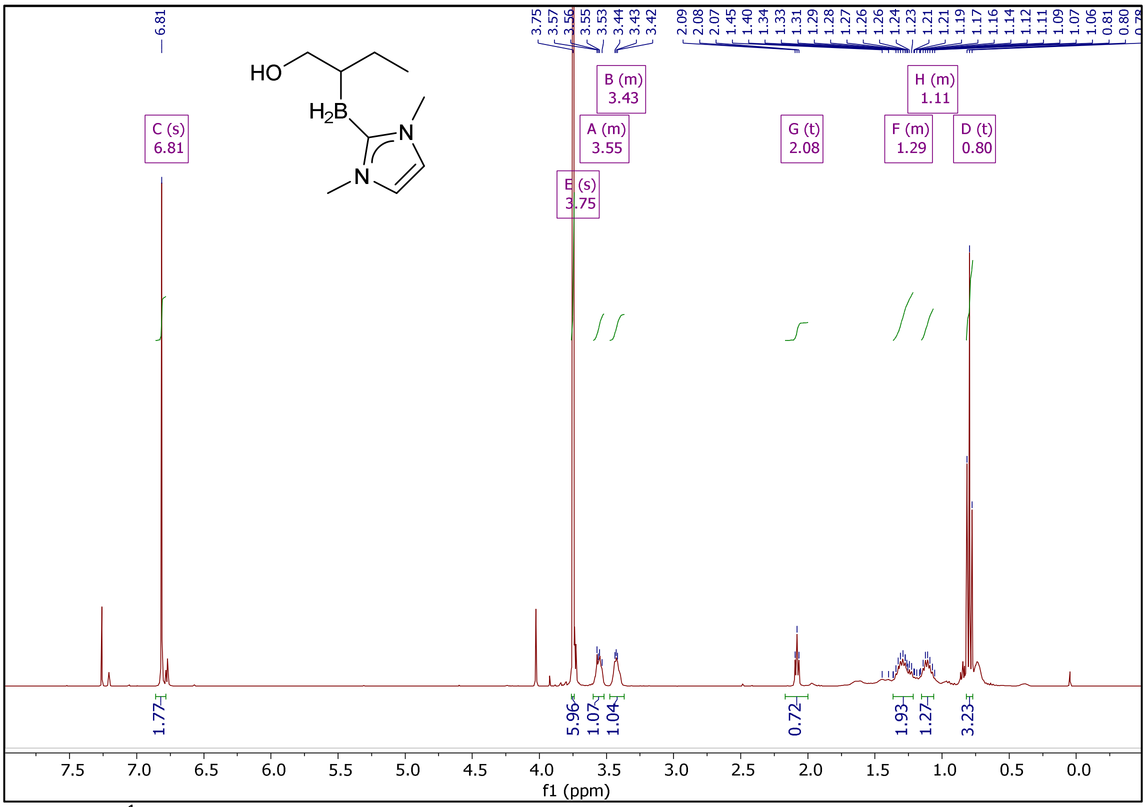

Figure S63: ^{1}H NMR spectrum of **6** ($CDCl_3$)

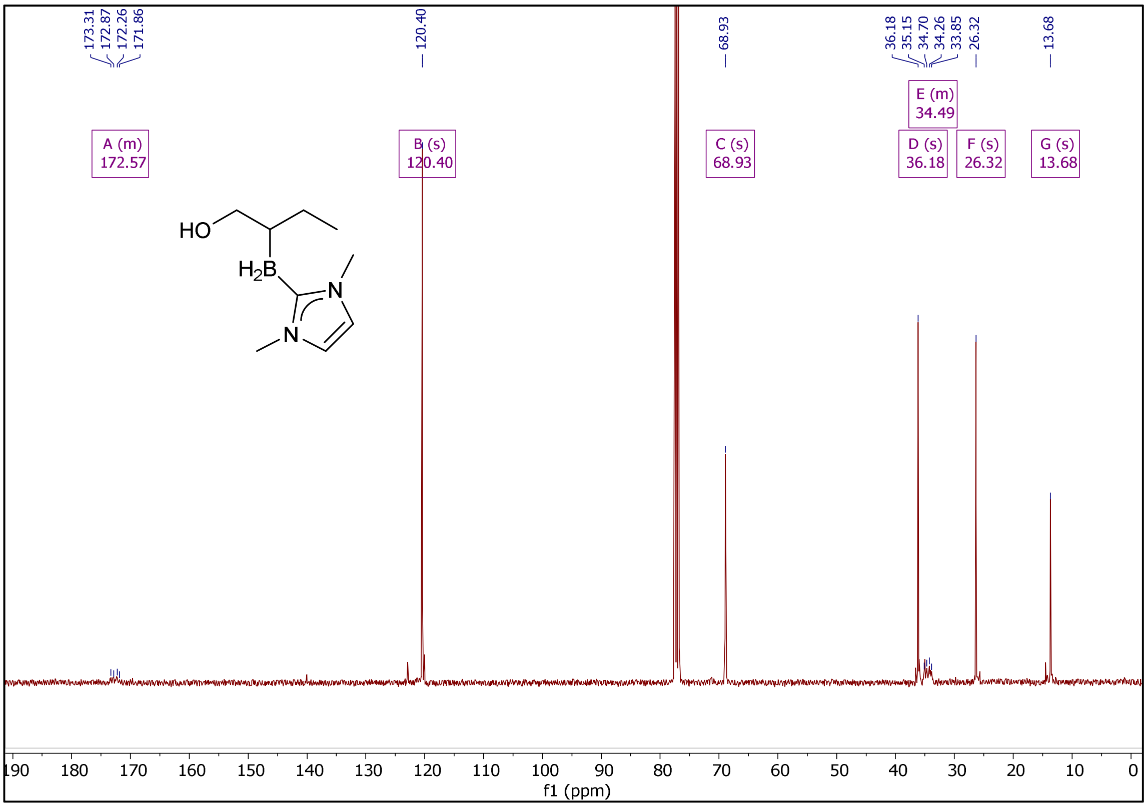

Figure S64: $^{13}C\{^{1}H\}$ NMR spectrum of **8** ($CDCl_3$)

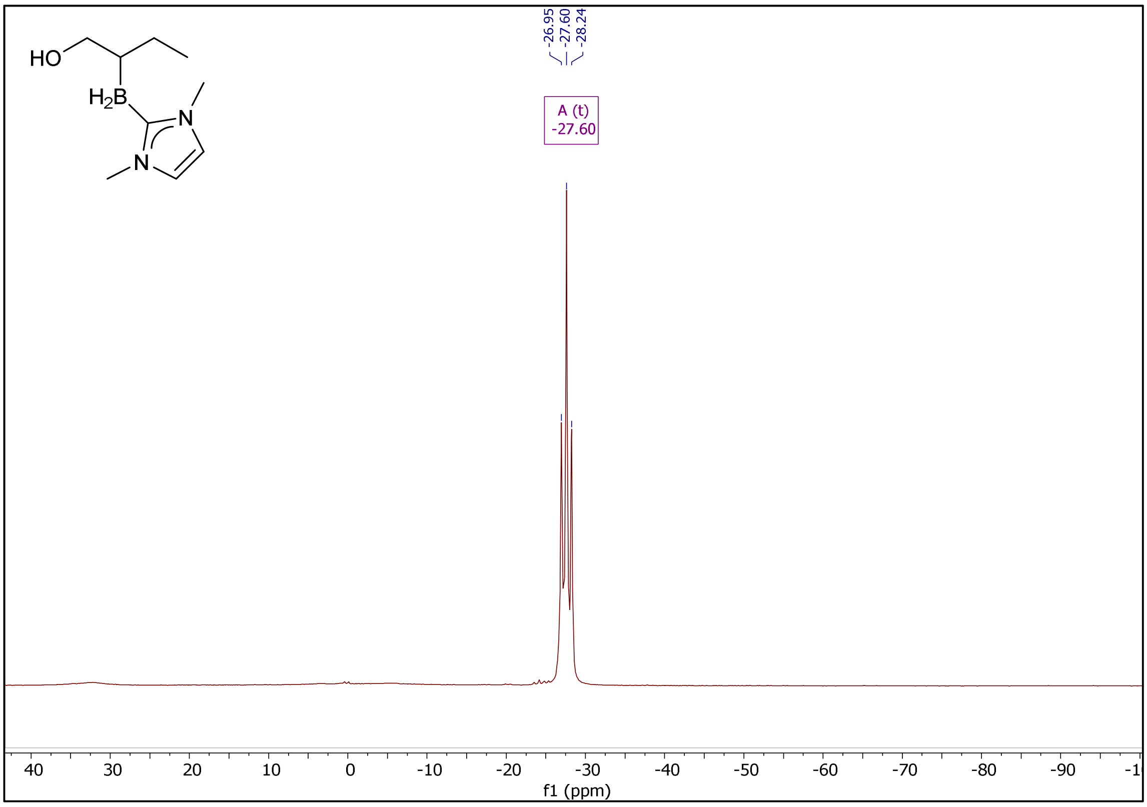

Figure S65: ^{11}B NMR spectrum of **8** ($CDCl_3$)

13.10 IMe-BH_2-Phalcohol 9

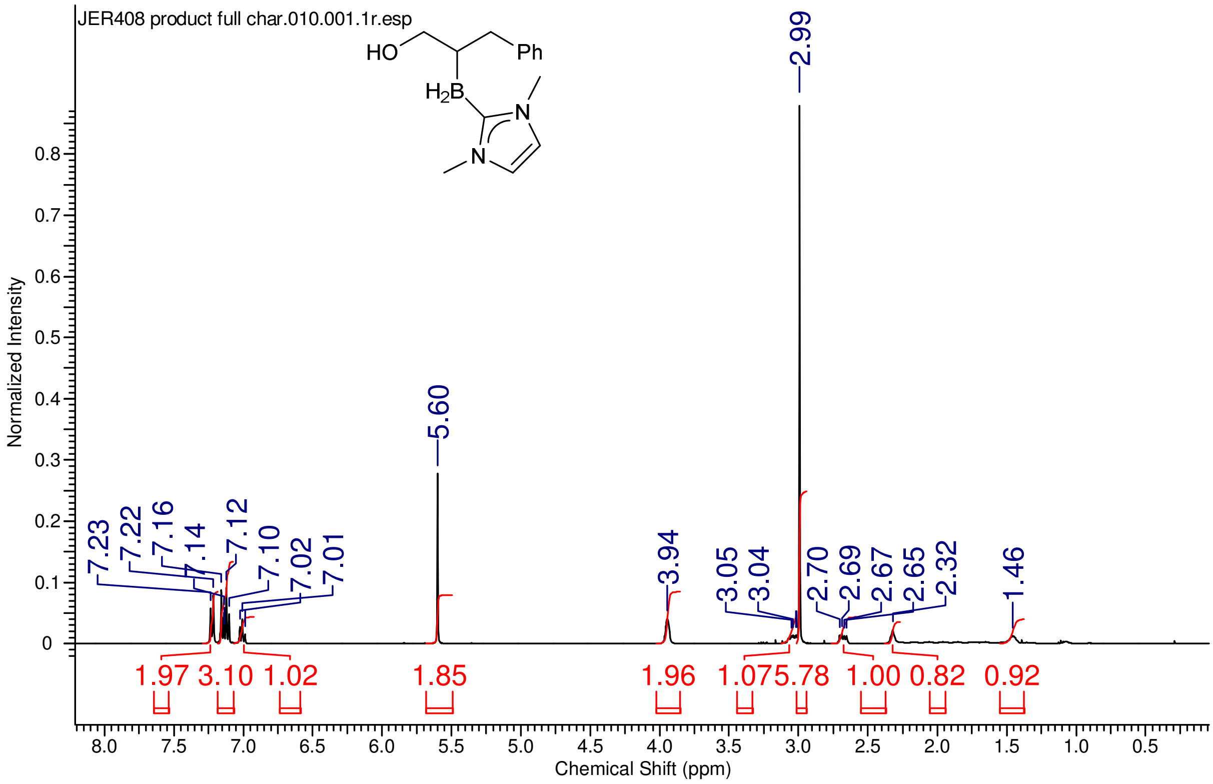

Figure S66: ^{1}H NMR spectrum of **9** ($CDCl_3$)

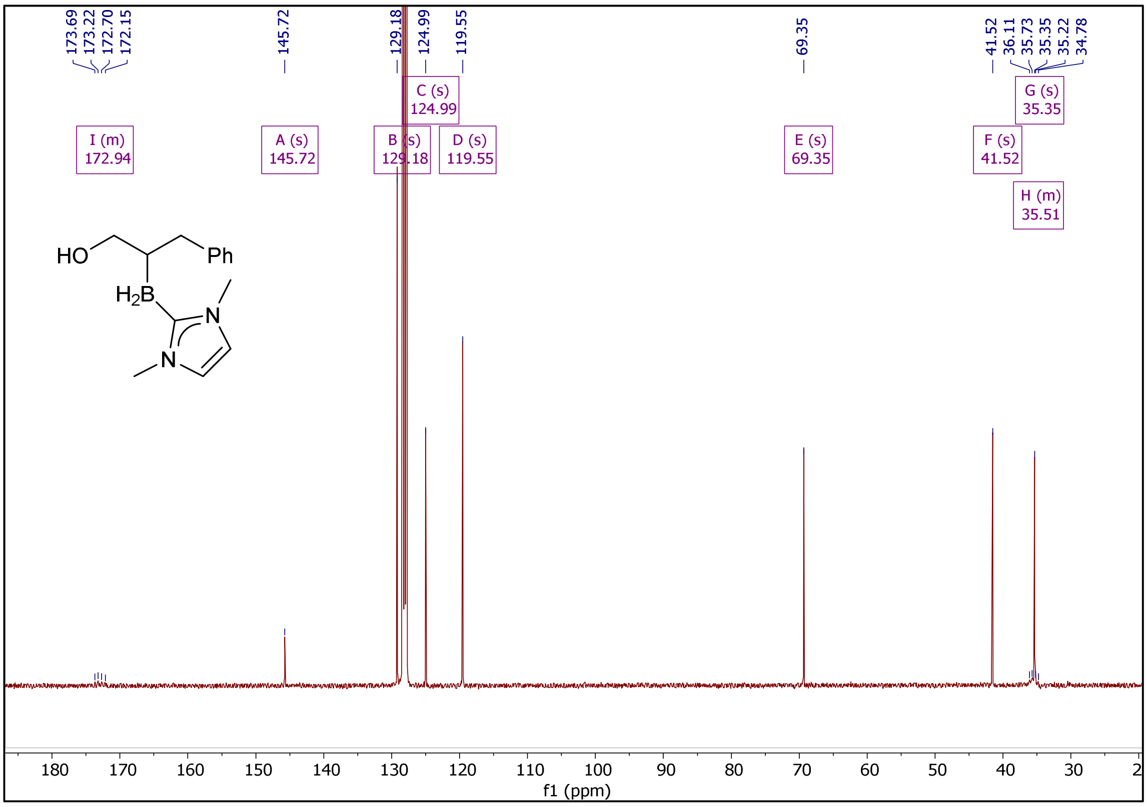

Figure S67: $^{13}C\{^{1}H\}$ NMR spectrum of **9** ($CDCl_3$)

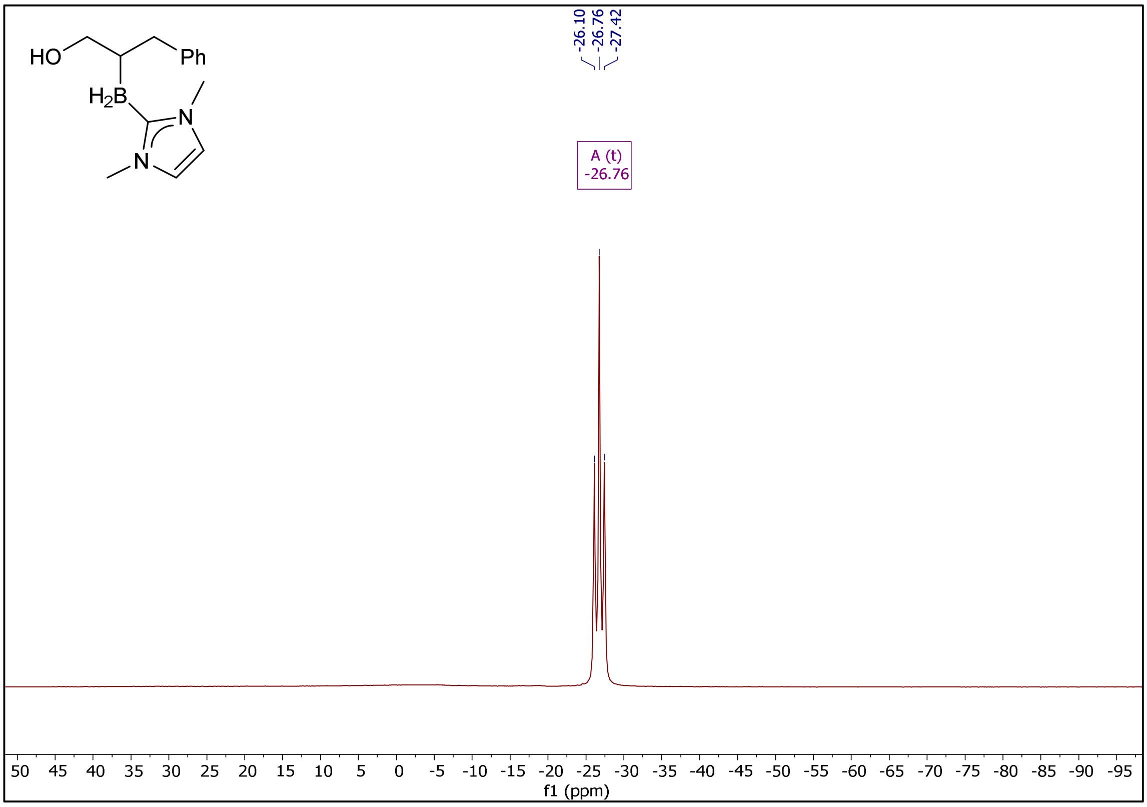

Figure S68: ^{11}B NMR spectrum of **9** ($CDCl_3$)

14 NMR Spectra of Catalysis runs

14.1 Low conc IMe-BH$_3$ + Methyl crotonate

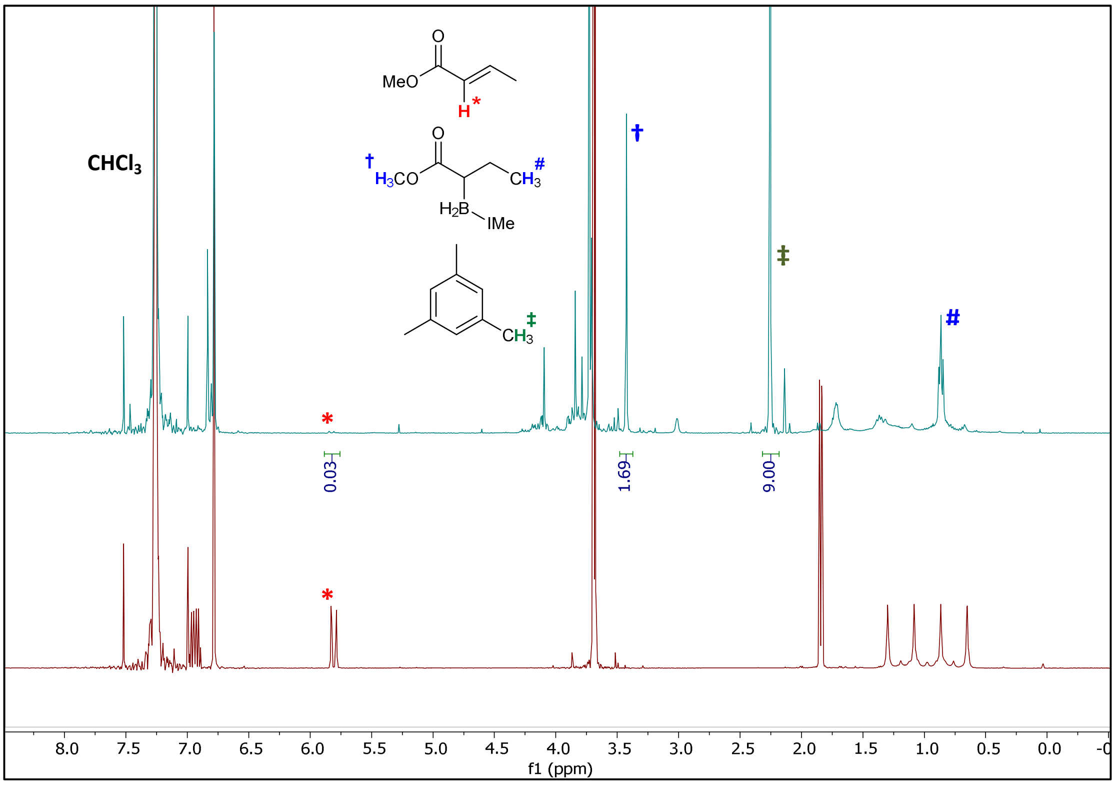

Figure S69: Collected ^{1}H NMR spectra ($CHCl_3$, 400 MHz, 298K with capillary insert containing C_6D_6) of: IMe-BH$_3$ and methyl crotonate (bottom); the reaction mixture 20 hours after the addition of I_2, with 1 eq. mesitylene as a standard (top).

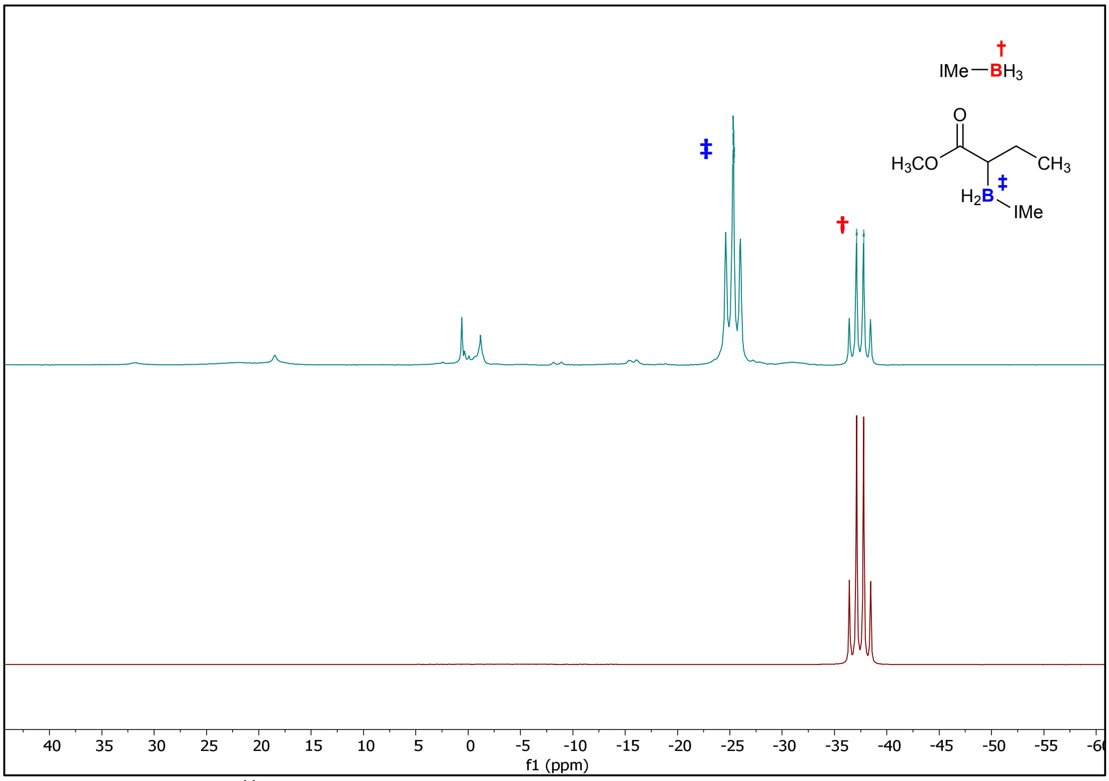

Figure S70: Collected ^{11}B NMR spectra ($CHCl_3$, 128.4 MHz, 298K) of: IMe-BH$_3$ and methyl crotonate (bottom); the reaction mixture 20 hours after the addition of I_2, with 1 eq. mesitylene as a standard (top).

14.2 High conc IMe-BH_3 + methyl crotonate

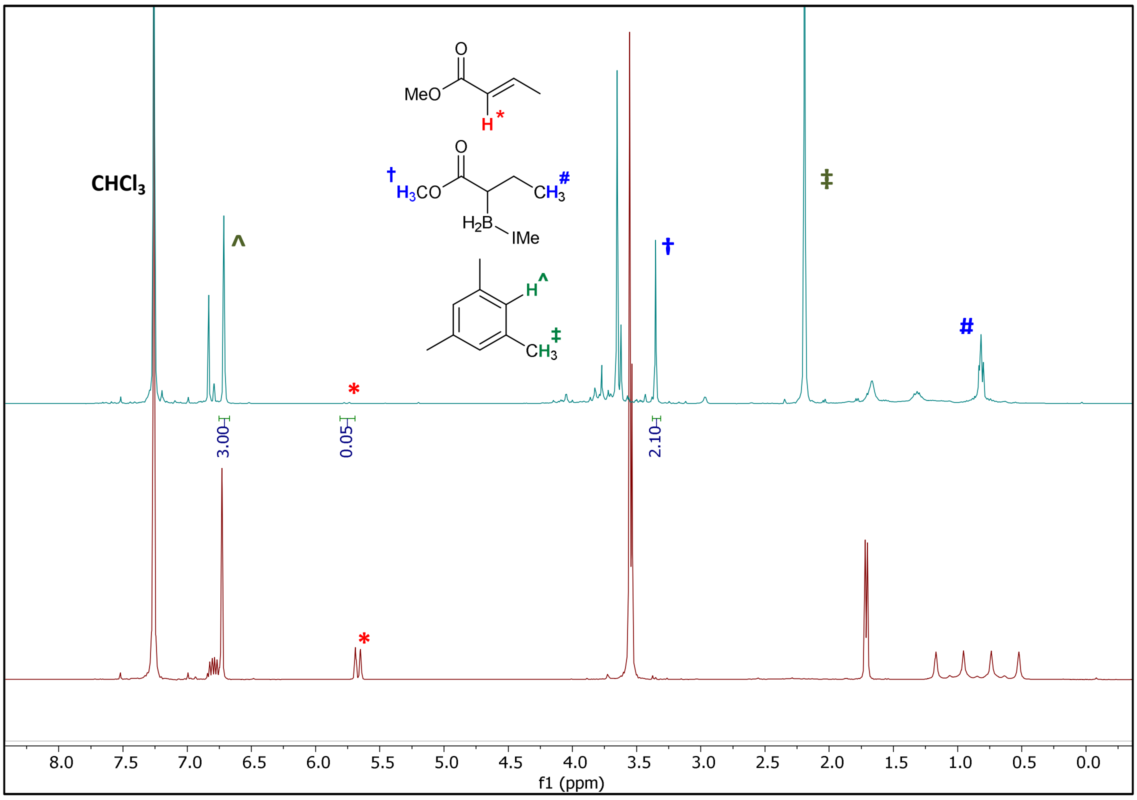

Figure S71: Collected ^{1}H NMR spectra ($CHCl_3$, 400 MHz, 298K with capillary insert containing C_6D_6) of: IMe-BH_3 and methyl crotonate (bottom); the reaction mixture 20 hours after the addition of I_2, with 1 eq. mesitylene as a standard (top).

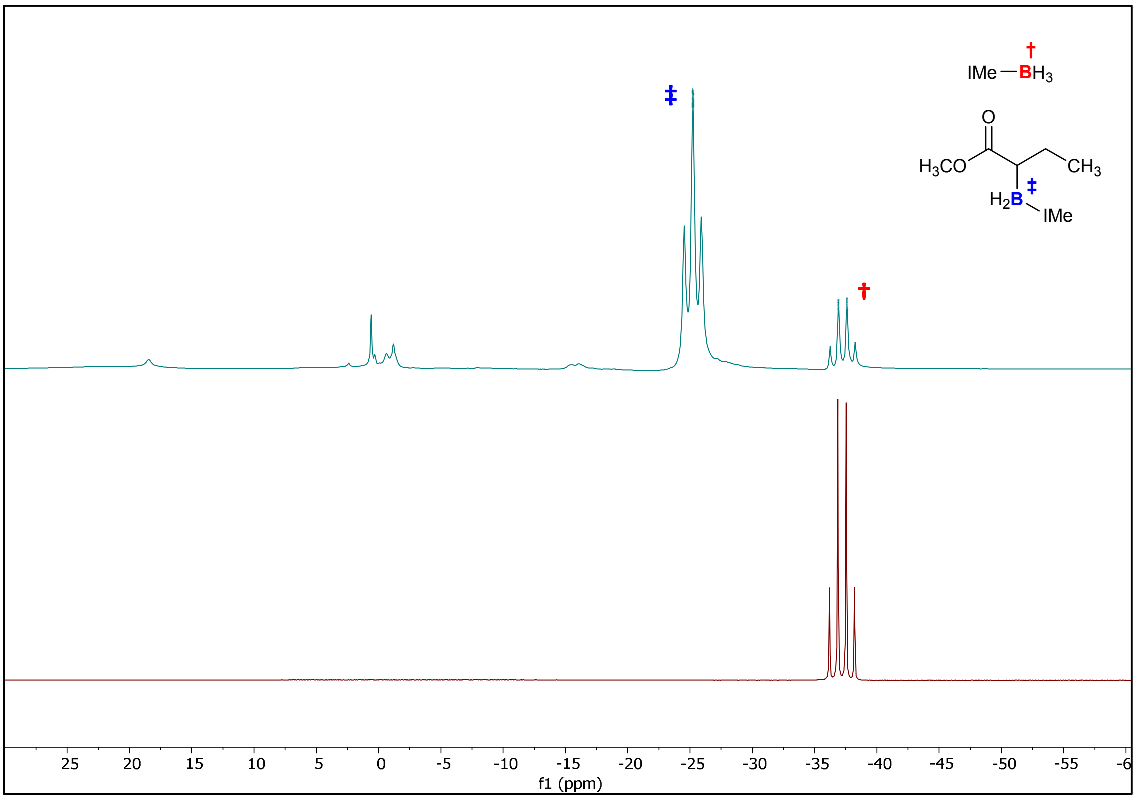

Figure S72: Collected ^{11}B NMR spectra ($CHCl_3$, 128.4 MHz, 298K) of: IMe-BH_3 and methyl crotonate (bottom); the reaction mixture 20 hours after the addition of I_2, with 1 eq. mesitylene as a standard (top).

14.3 $B(C_6F_5)_3$ as initiator

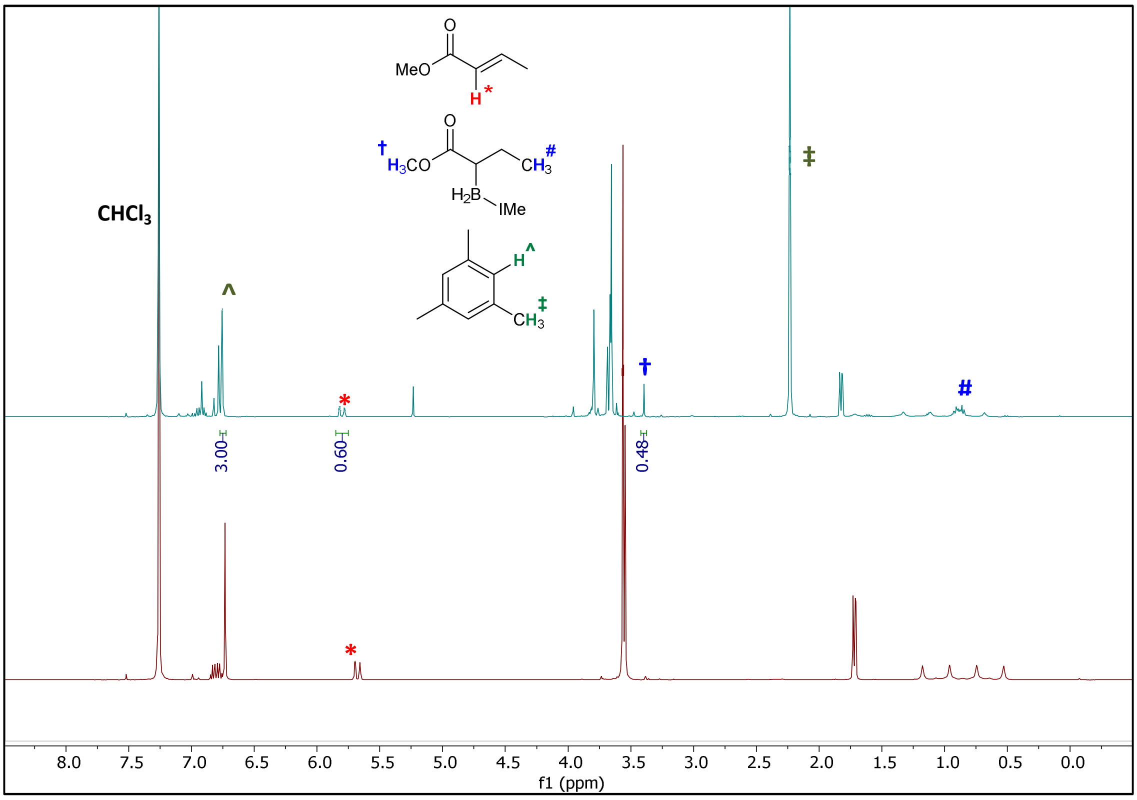

Figure S73: Collected ^{1}H NMR spectra ($CHCl_3$, 400 MHz, 298K with capillary insert containing C_6D_6) of: IMe-BH_3 and methyl crotonate (bottom); the reaction mixture 20 hours after the addition of BCF, with 1 eq. mesitylene as a standard (top).

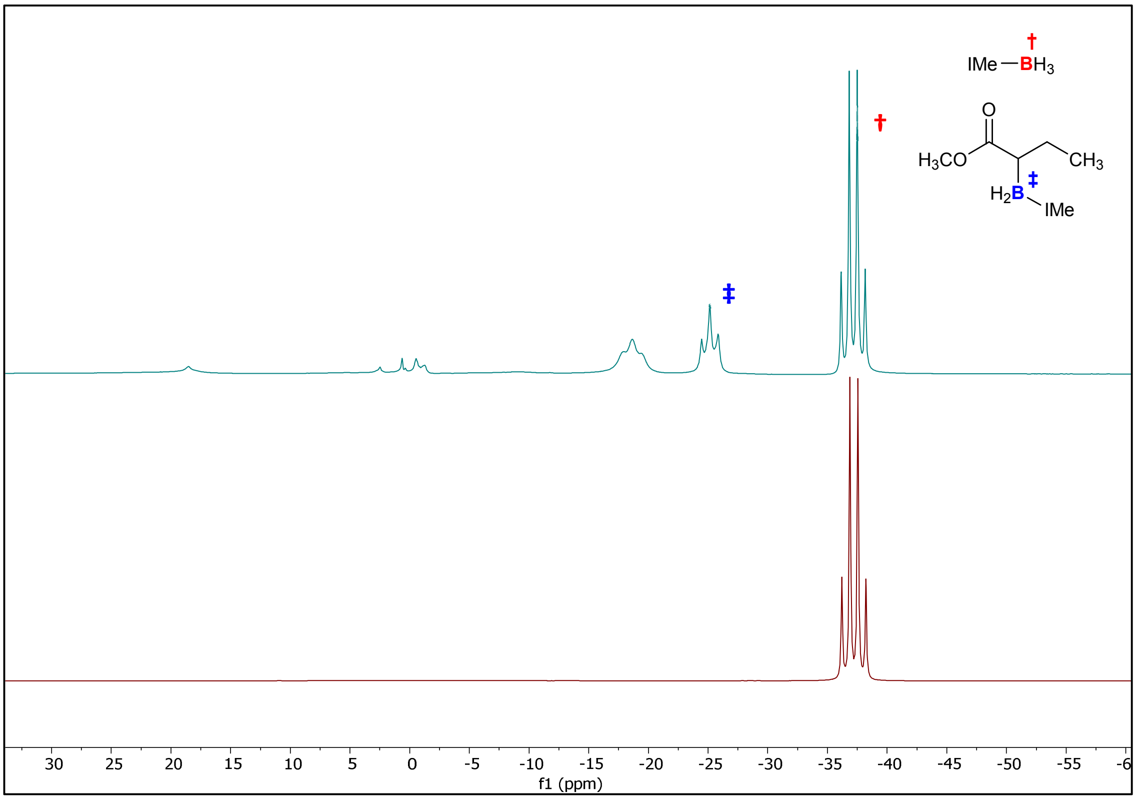

Figure S74: Collected ^{11}B NMR spectra ($CHCl_3$, 128.4 MHz, 298K) of: IMe-BH_3 and methyl crotonate (bottom); the reaction mixture 20 hours after the addition of BCF, with 1 eq. mesitylene as a standard (top).

14.4 TBHP initiator

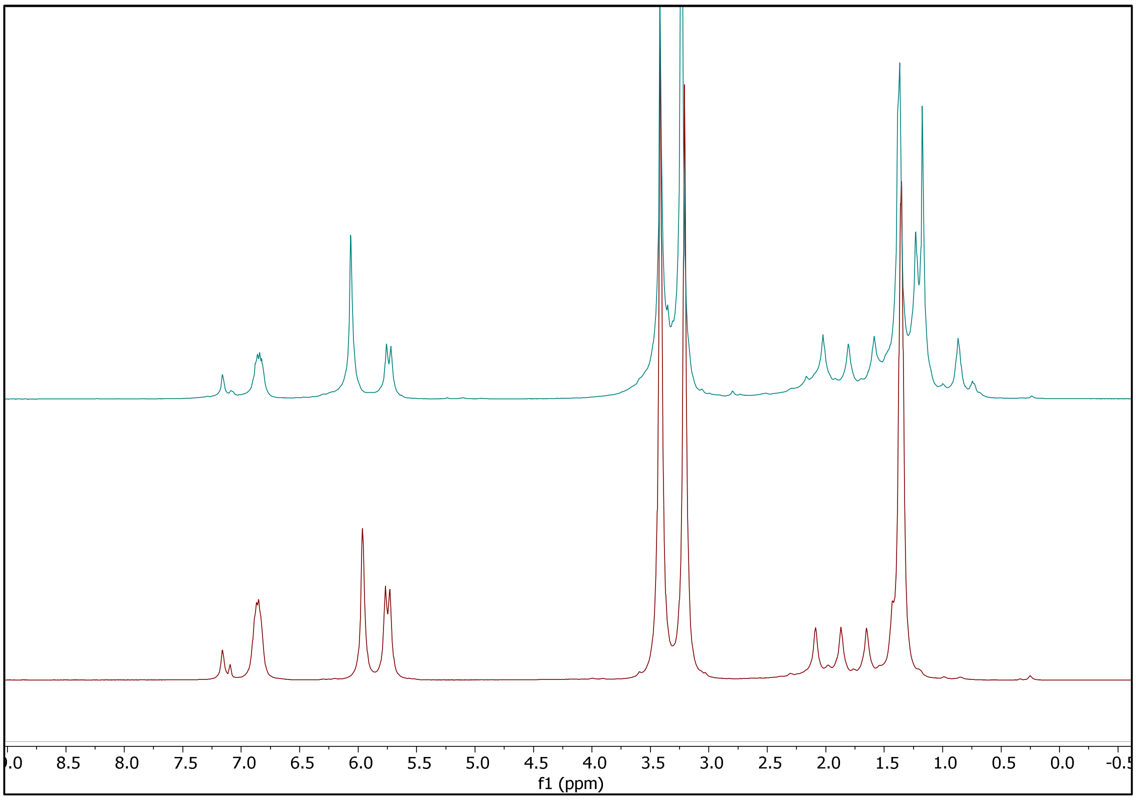

Figure S75: Collected ^{1}H NMR spectra (C_6D_6, 400 MHz, 298K) of: IMe-BH_3 and methyl crotonate (bottom); the reaction mixture after the addition of TBHP and subsequent heating at 60 °C for 18 hours (top).

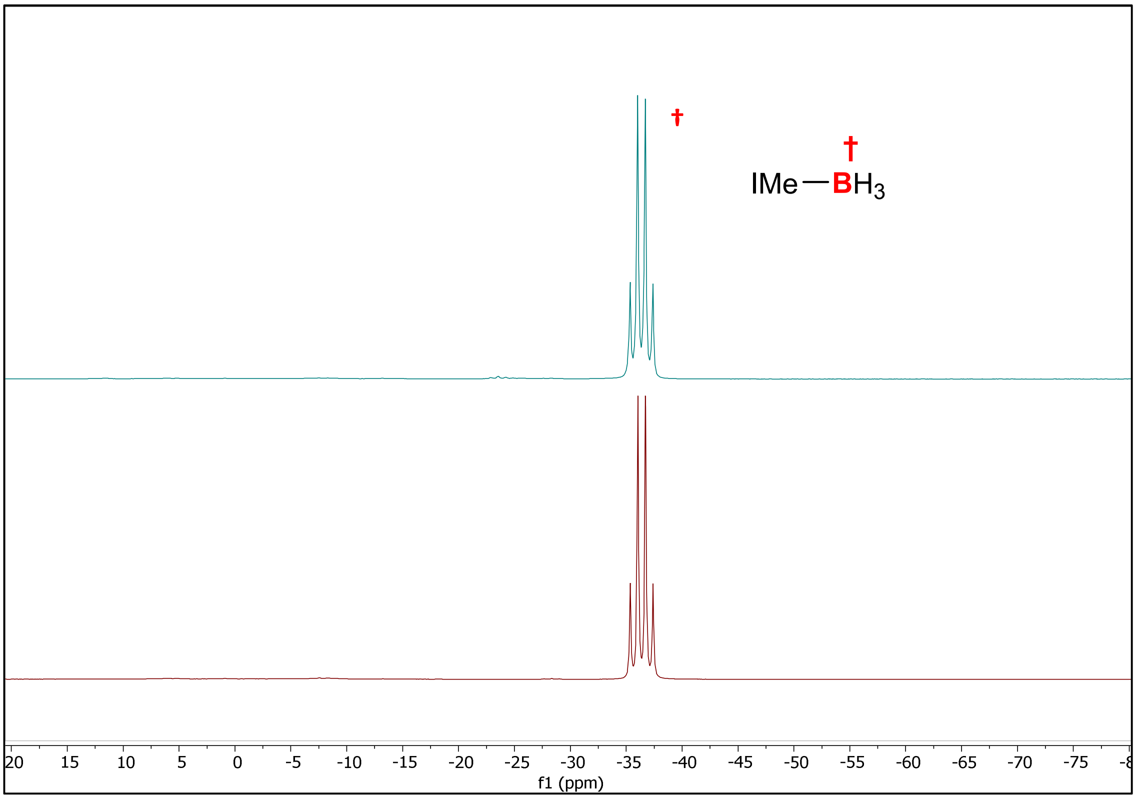

Figure S76: Collected ^{11}B NMR spectra (C_6D_6, 128.4 MHz, 298K) of: IMe-BH_3 and methyl crotonate (bottom); the reaction mixture after the addition of TBHP and subsequent heating at 60 °C for 18 hours (top).

14.5 BenzIMe-BH$_3$ + methyl crotonate

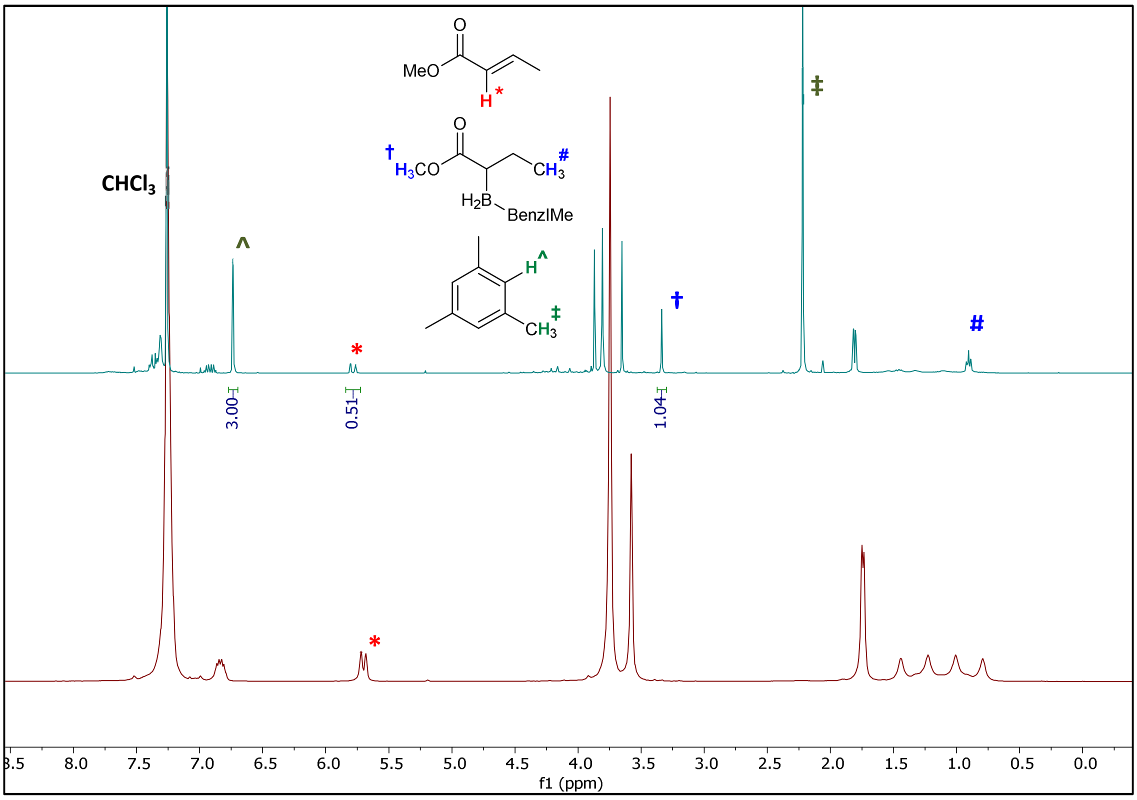

Figure S77: Collected ^{1}H NMR spectra ($CHCl_3$, 400 MHz, 298K with capillary insert containing C_6D_6) of: BenzIMe-BH$_3$ and methyl crotonate (bottom); the reaction mixture 20 hours after the addition of I_2, with 1 eq. mesitylene as a standard (top).

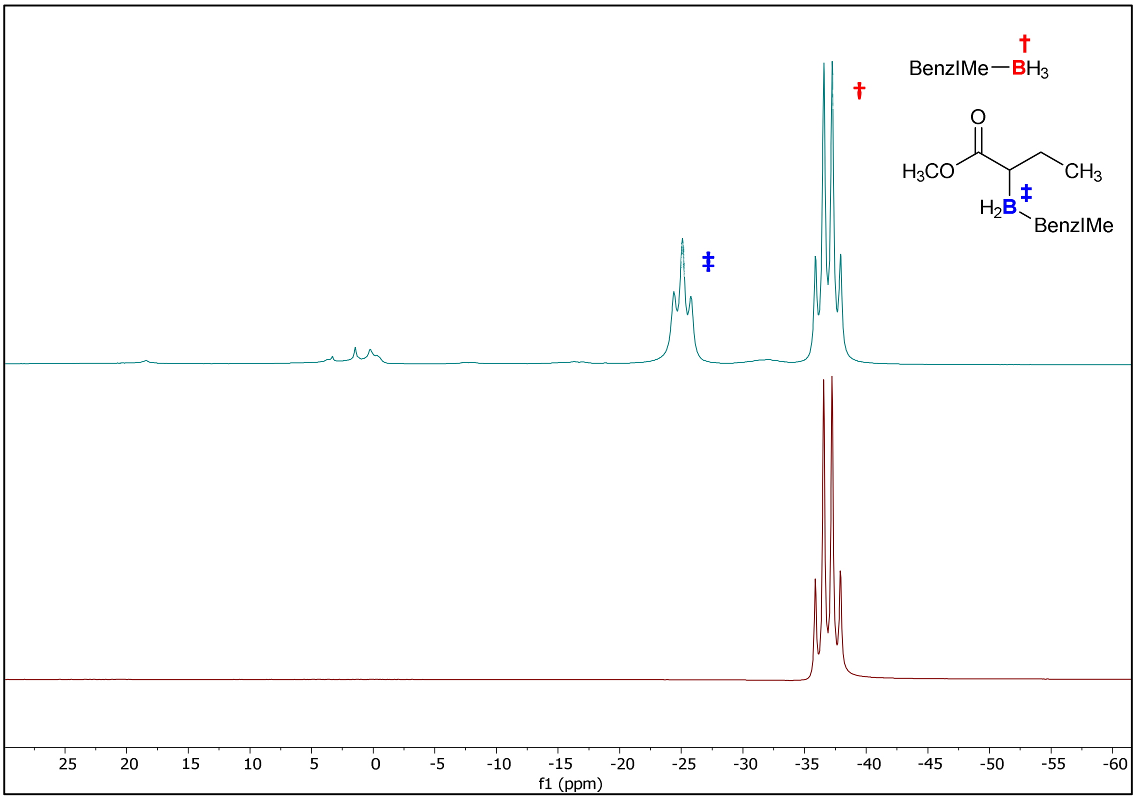

Figure S78: Collected ^{11}B NMR spectra ($CHCl_3$, 128.4 MHz, 298K) of: BenzIMe-BH$_3$ and methyl crotonate (bottom); the reaction mixture 20 hours after the addition of I_2, with 1 eq. mesitylene as a standard (top).

14.6 IPr-BH_3 + methyl crotonate

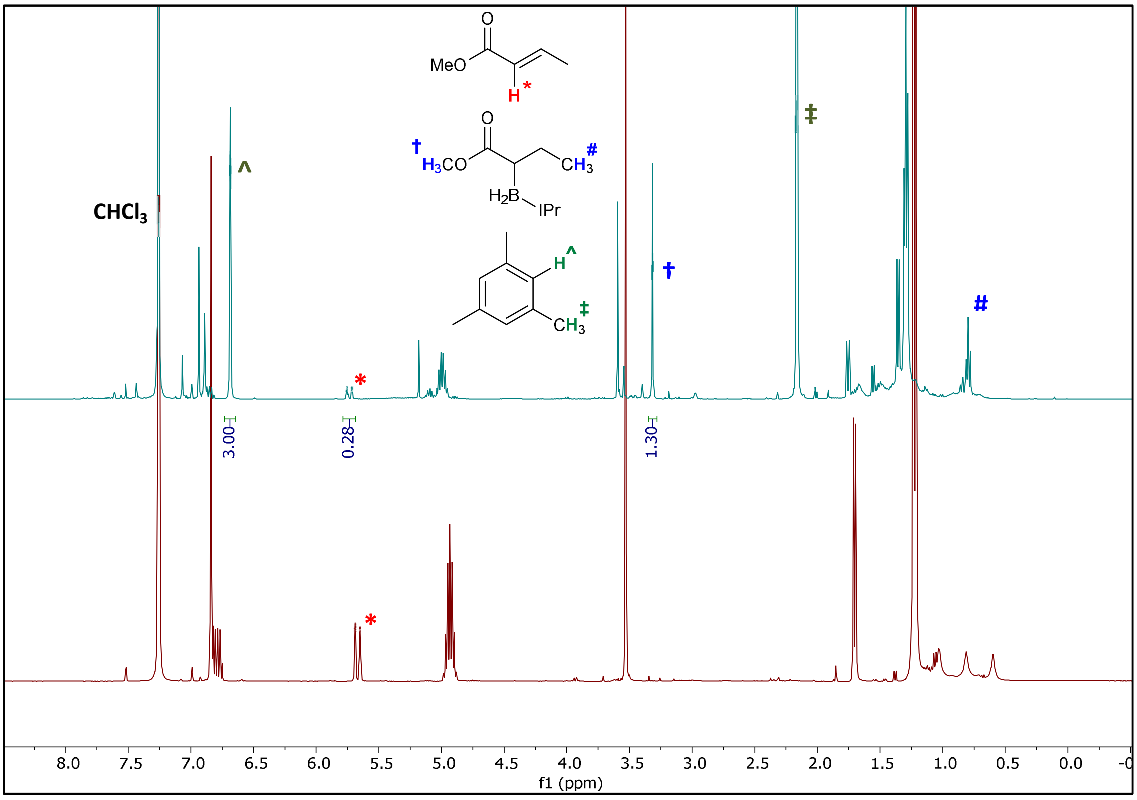

Figure S79: Collected ^{1}H NMR spectra ($CHCl_3$, 400 MHz, 298K with capillary insert containing C_6D_6) of: IPr-BH_3 and methyl crotonate (bottom); the reaction mixture 20 hours after the addition of I_2, with 1 eq. mesitylene as a standard (top).

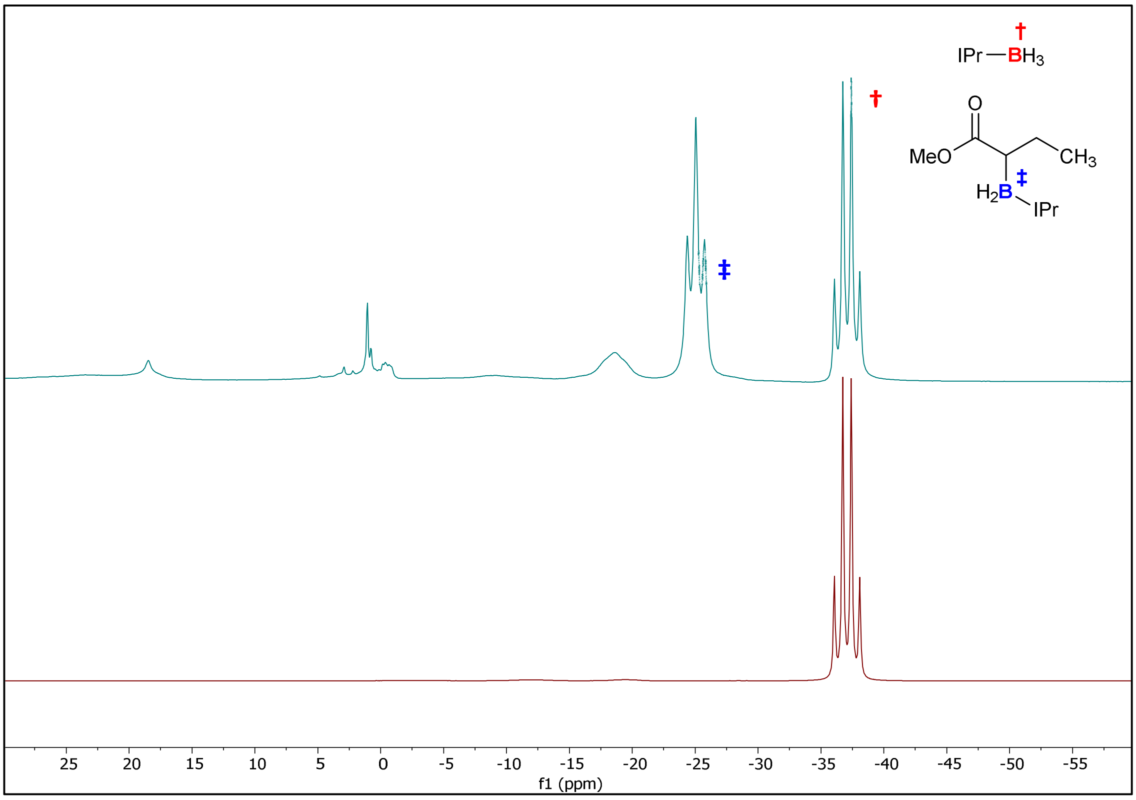

Figure S80: Collected ^{11}B NMR spectra ($CHCl_3$, 128.4 MHz, 298K) of: IPr-BH_3 and methyl crotonate (bottom); the reaction mixture 20 hours after the addition of I_2, with 1 eq. mesitylene as a standard (top).

14.7 **Ethyl crotonate**

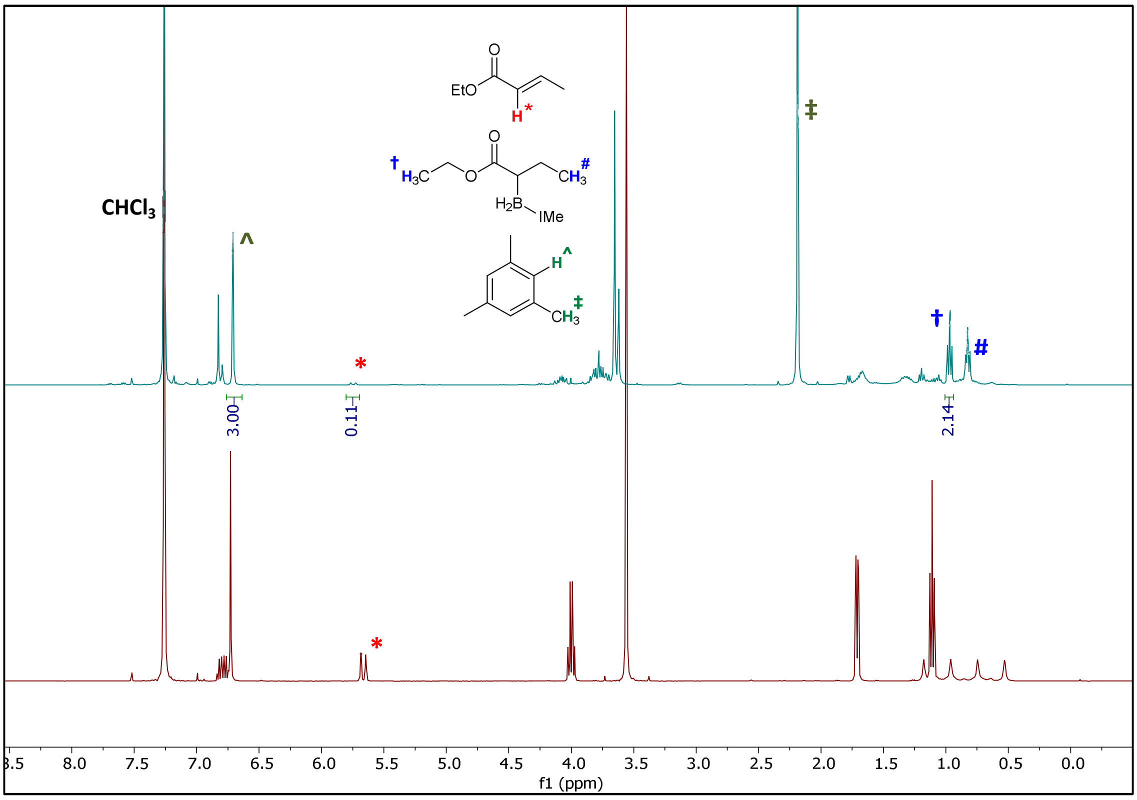

Figure S81: Collected ^{1}H NMR spectra ($CHCl_3$, 400 MHz, 298K with capillary insert containing C_6D_6) of: IMe-BH_3 and ethyl crotonate (bottom); the reaction mixture 20 hours after the addition of I_2, with 1 eq. mesitylene as a standard (top).

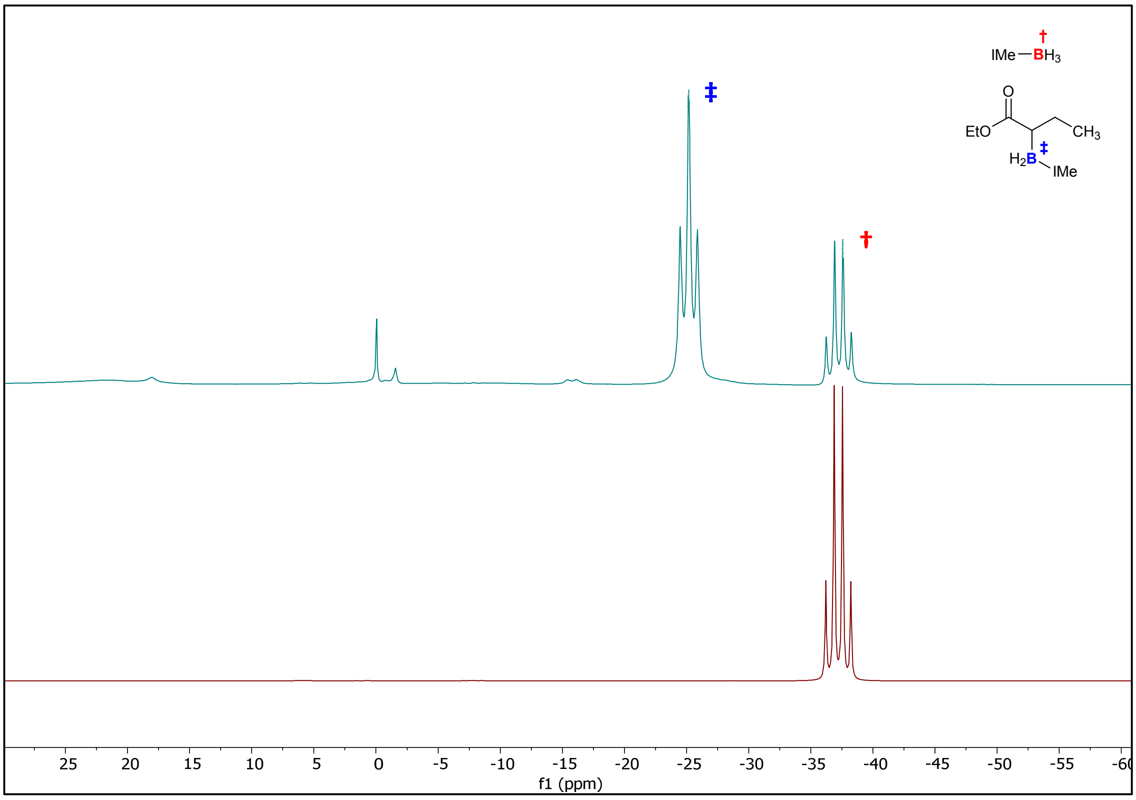

Figure S82: Collected ^{11}B NMR spectra ($CHCl_3$, 128.4 MHz, 298K) of: IMe-BH_3 and ethyl crotonate (bottom); the reaction mixture 20 hours after the addition of I_2, with 1 eq. mesitylene as a standard (top).

14.8 **tBu-crotonate**

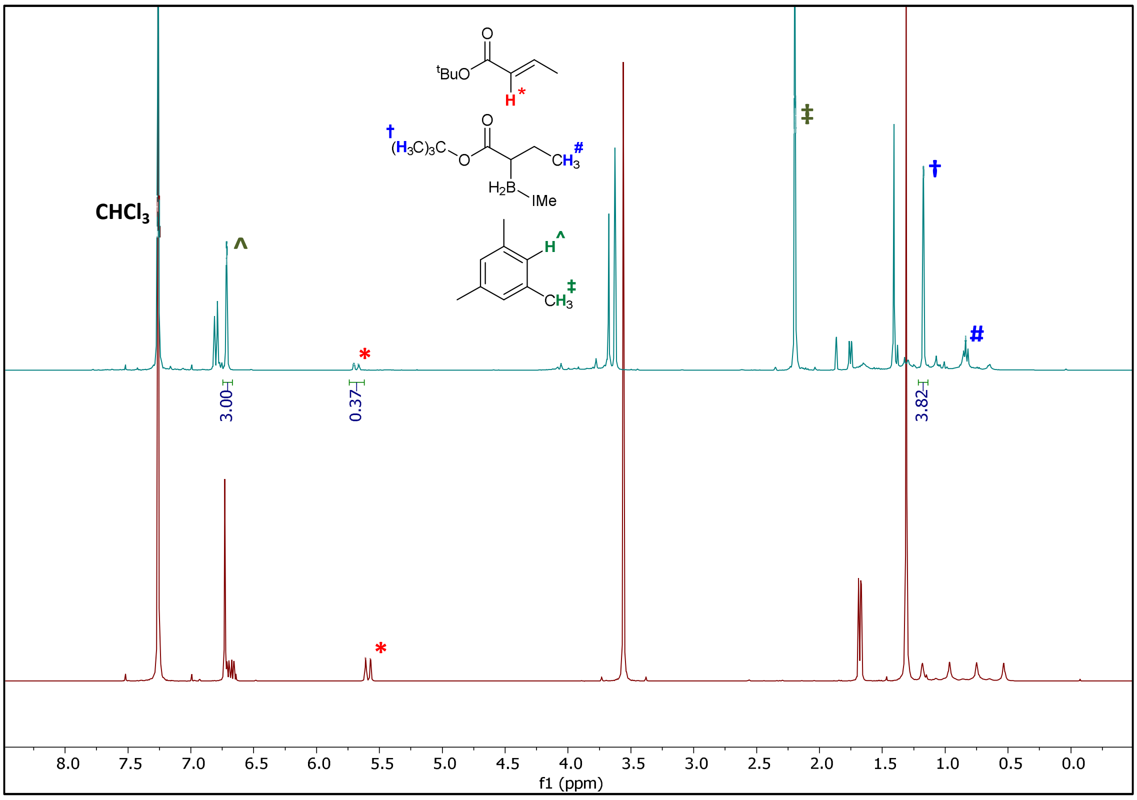

Figure S83: Collected ^{1}H NMR spectra ($CHCl_3$, 400 MHz, 298K with capillary insert containing C_6D_6) of: IMe-BH_3 and *tert*-butyl crotonate (bottom); the reaction mixture 20 hours after the addition of I_2, with 1 eq. mesitylene as a standard (top).

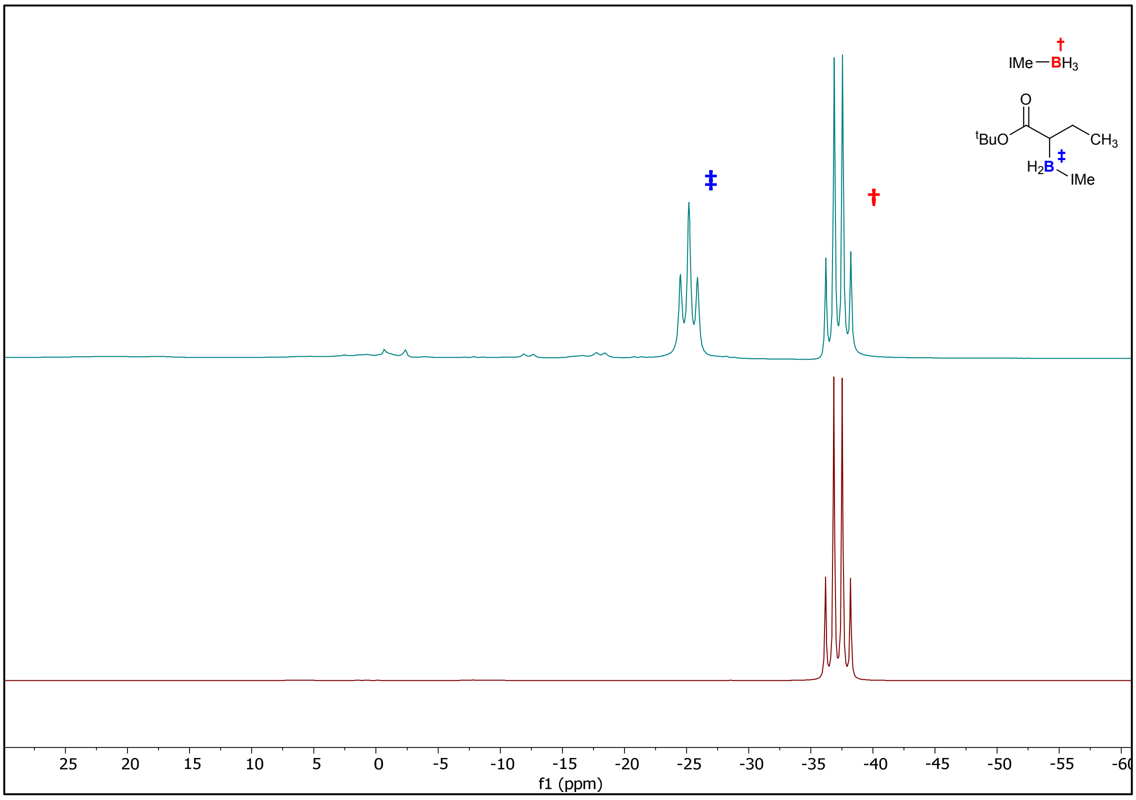

Figure S84: Collected ^{11}B NMR spectra ($CHCl_3$, 128.4 MHz, 298K) of: IMe-BH_3 and *tert*-butyl crotonate (bottom); the reaction mixture 20 hours after the addition of I_2, with 1 eq. mesitylene as a standard (top).

14.9 **Methyl cinnamate**

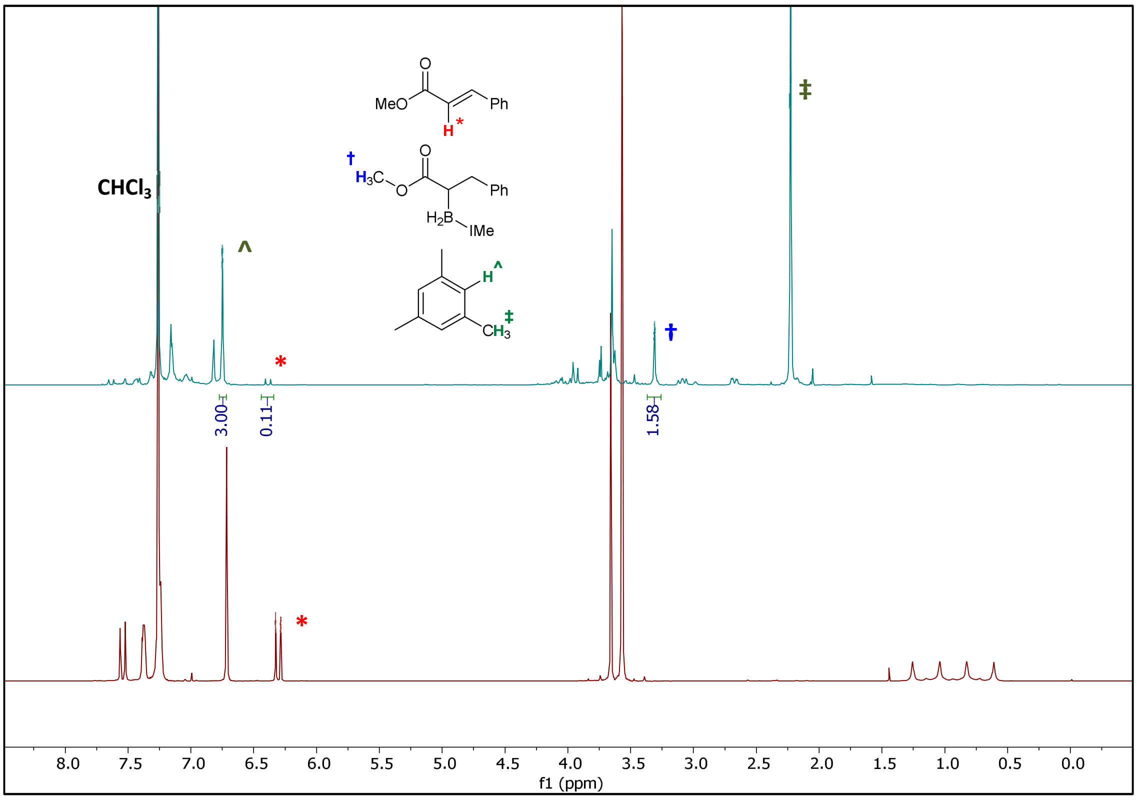

Figure S85: Collected ^{1}H NMR spectra ($CHCl_3$, 400 MHz, 298K with capillary insert containing C_6D_6) of: IMe-BH_3 and methyl cinnamate (bottom); the reaction mixture 20 hours after the addition of I_2, with 1 eq. mesitylene as a standard (top).

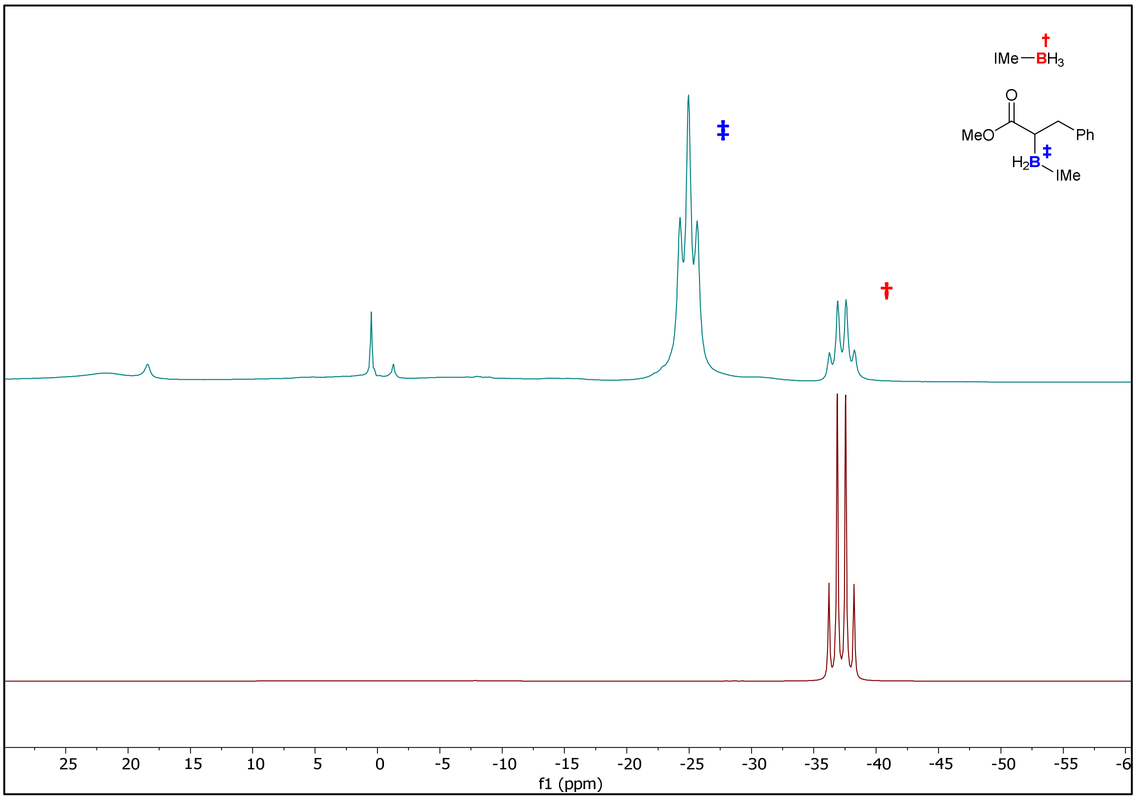

Figure S86: Collected ^{11}B NMR spectra ($CHCl_3$, 128.4 MHz, 298K) of: IMe-BH_3 and methyl cinnamate (bottom); the reaction mixture 20 hours after the addition of I_2, with 1 eq. mesitylene as a standard (top).

14.10 **Ethyl cinnamate**

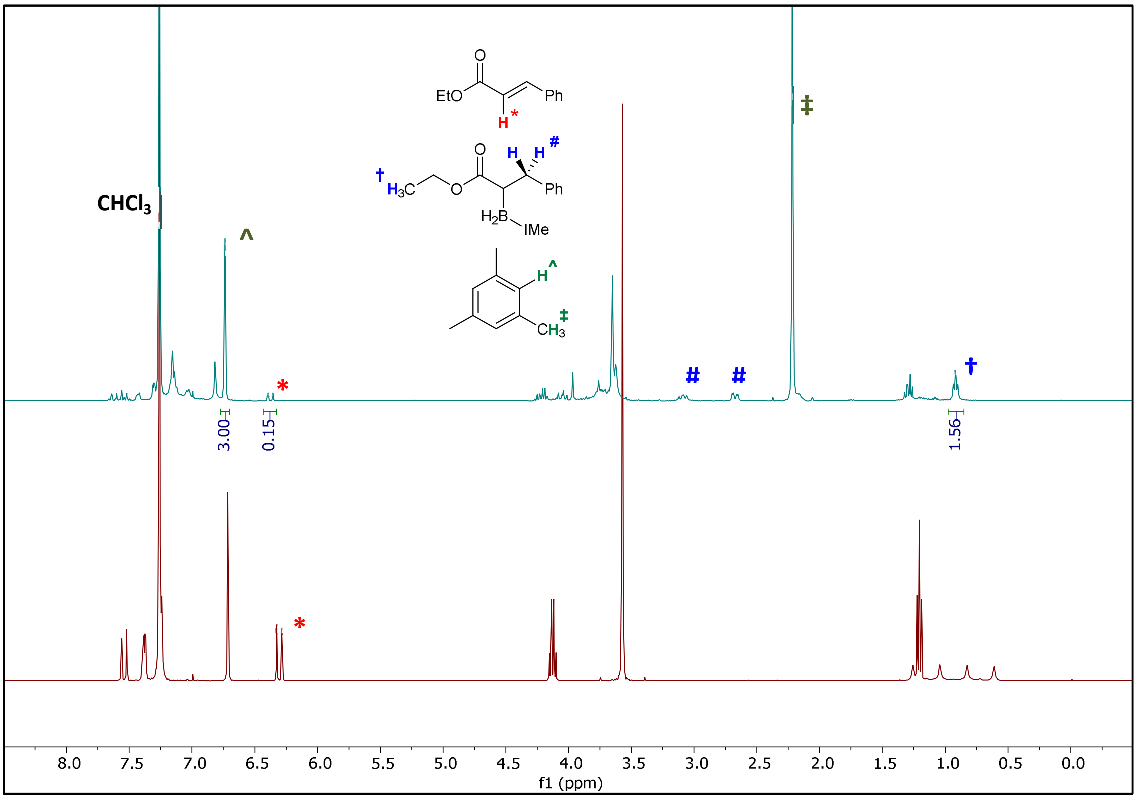

Figure S87: Collected ^{1}H NMR spectra ($CHCl_3$, 400 MHz, 298K with capillary insert containing C_6D_6) of: IMe-BH_3 and ethyl cinnamate (bottom); the reaction mixture 20 hours after the addition of I_2, with 1 eq. mesitylene as a standard (top).

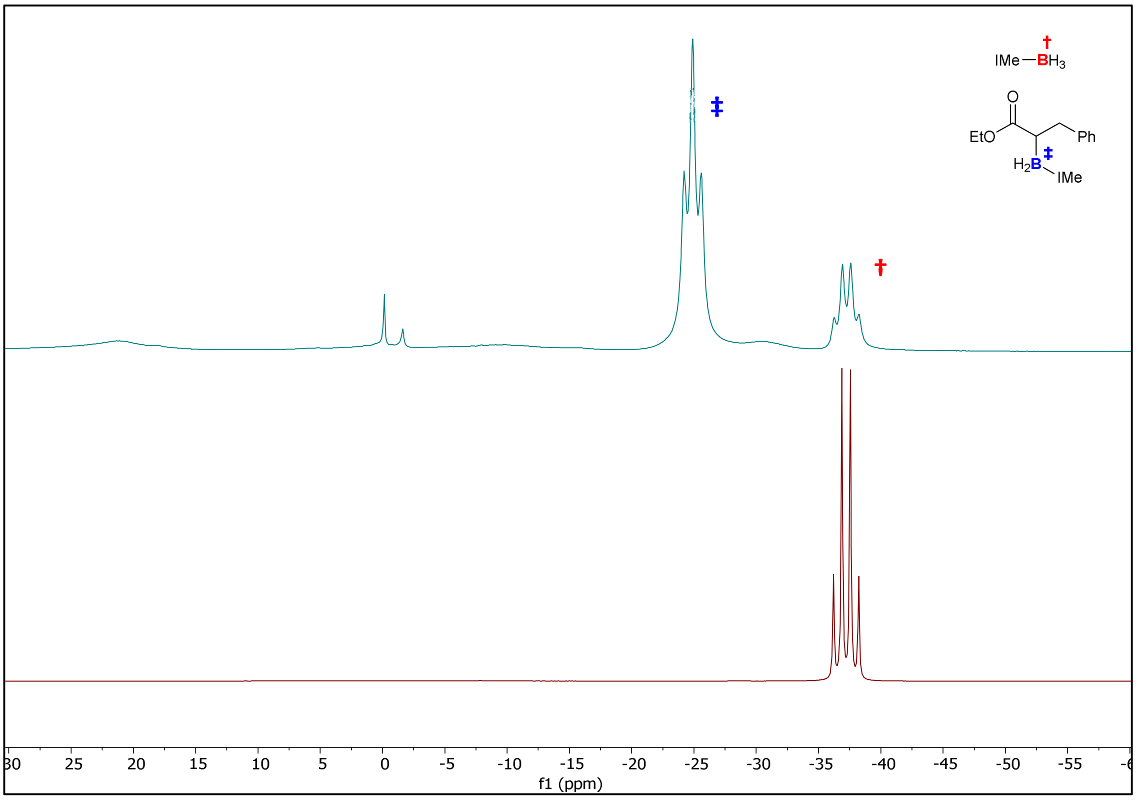

Figure S88: Collected ^{11}B NMR spectra ($CHCl_3$, 128.4 MHz, 298K) of: IMe-BH_3 and ethyl cinnamate (bottom); the reaction mixture 20 hours after the addition of I_2, with 1 eq. mesitylene as a standard (top).

14.11 **Iso-propyl cinnamate**

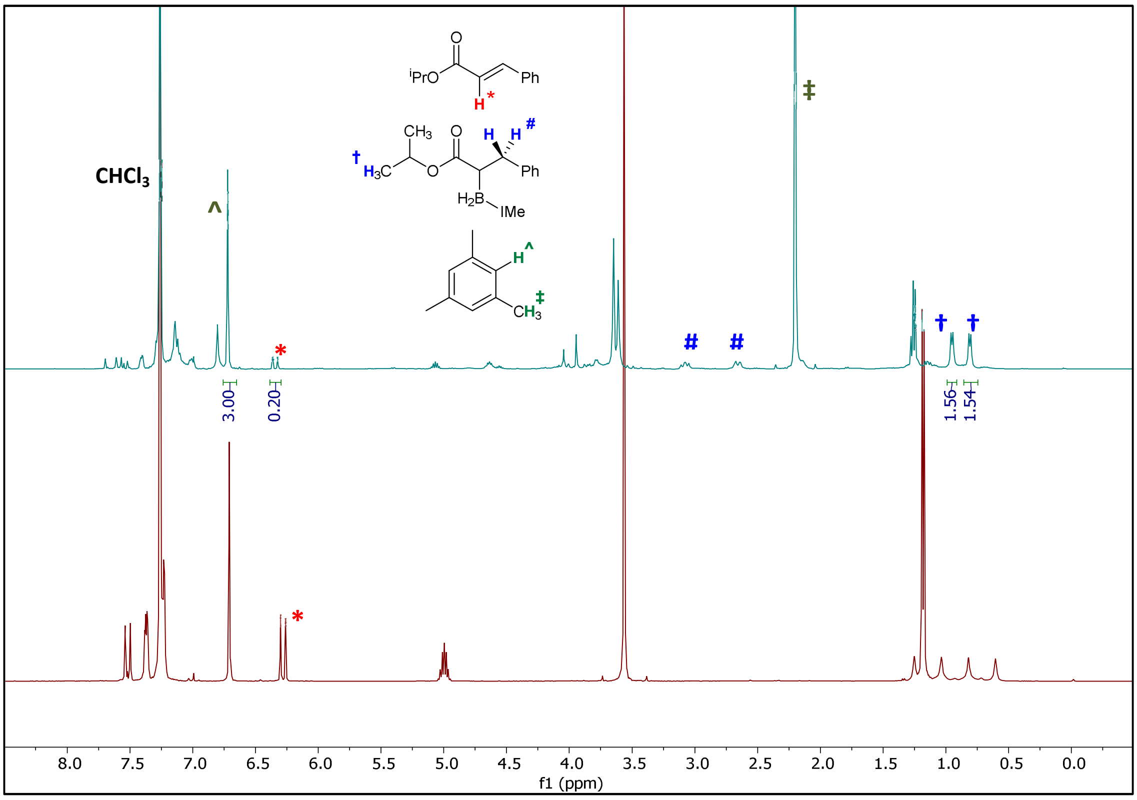

Figure S89: Collected ^{1}H NMR spectra ($CHCl_3$, 400 MHz, 298K with capillary insert containing C_6D_6) of: IMe-BH_3 and *iso*-propyl cinnamate (bottom); the reaction mixture 20 hours after the addition of I_2, with 1 eq. mesitylene as a standard (top).

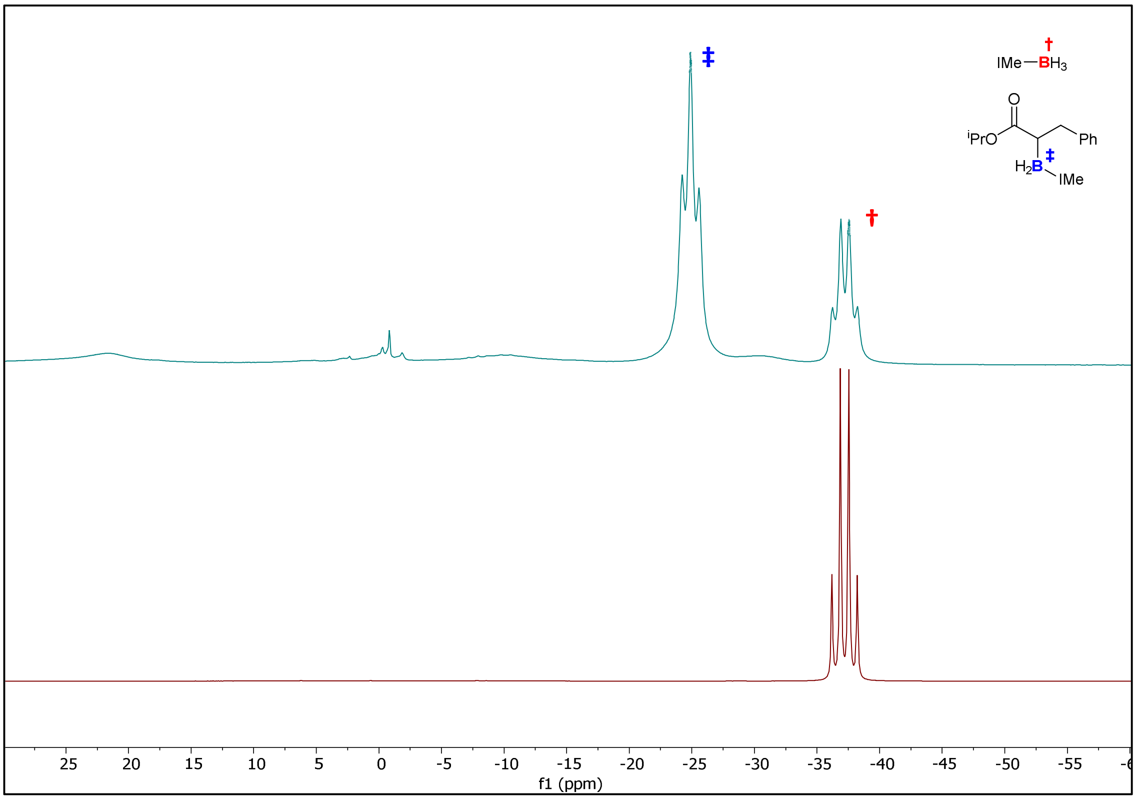

Figure S90: Collected ^{11}B NMR spectra ($CHCl_3$, 128.4 MHz, 298K) of: IMe-BH_3 and *iso*-propyl cinnamate (bottom); the reaction mixture 20 hours after the addition of I_2, with 1 eq. mesitylene as a standard (top).

14.12 **Benzyl cinnamate**

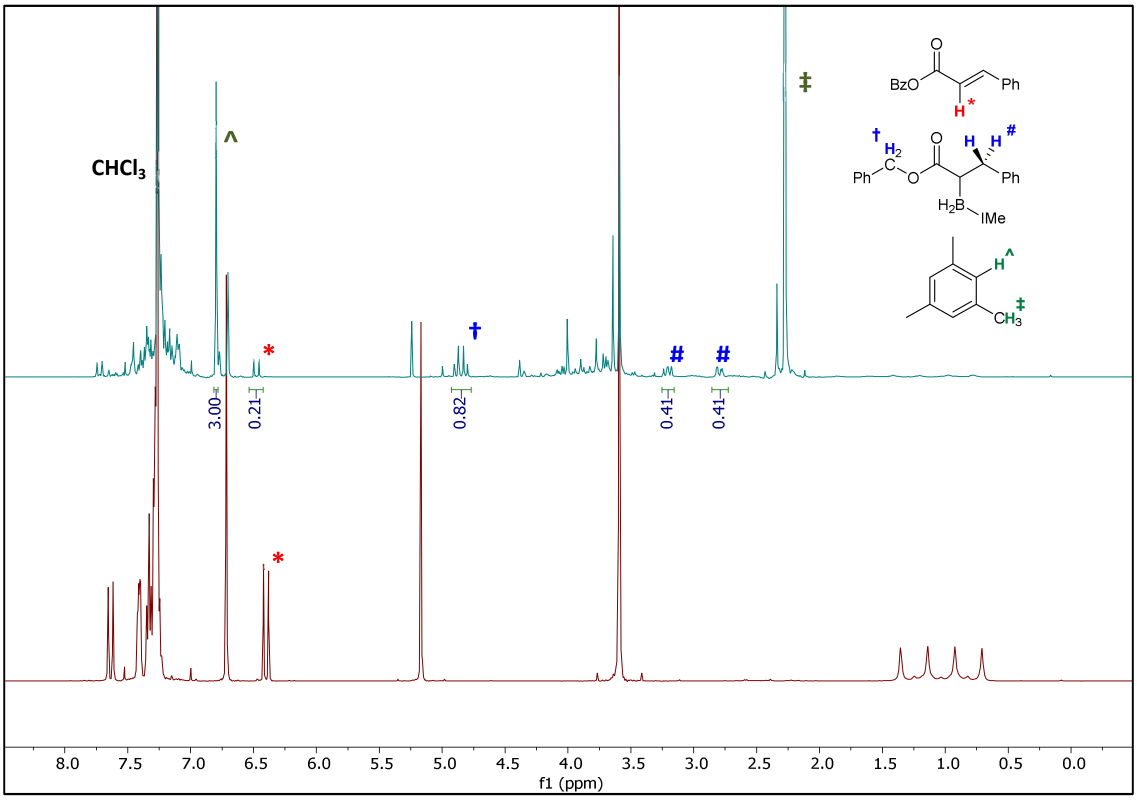

Figure S91: Collected ^{1}H NMR spectra ($CHCl_3$, 400 MHz, 298K with capillary insert containing C_6D_6) of: IMe-BH_3 and benzyl cinnamate (bottom); the reaction mixture 20 hours after the addition of I_2, with 1 eq. mesitylene as a standard (top).

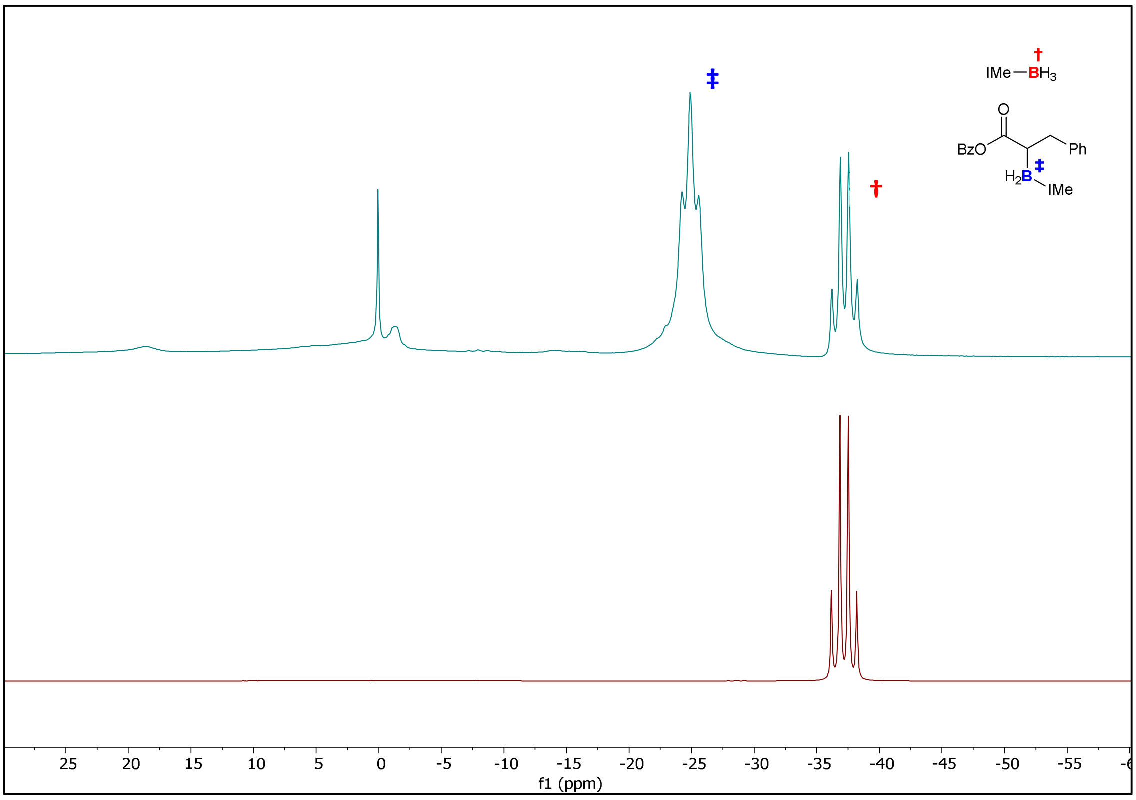

Figure S92: Collected ^{11}B NMR spectra ($CHCl_3$, 128.4 MHz, 298K) of: IMe-BH_3 and benzyl cinnamate (bottom); the reaction mixture 20 hours after the addition of I_2, with 1 eq. mesitylene as a standard (top).

14.13 **Methyl dimethylacrylate**

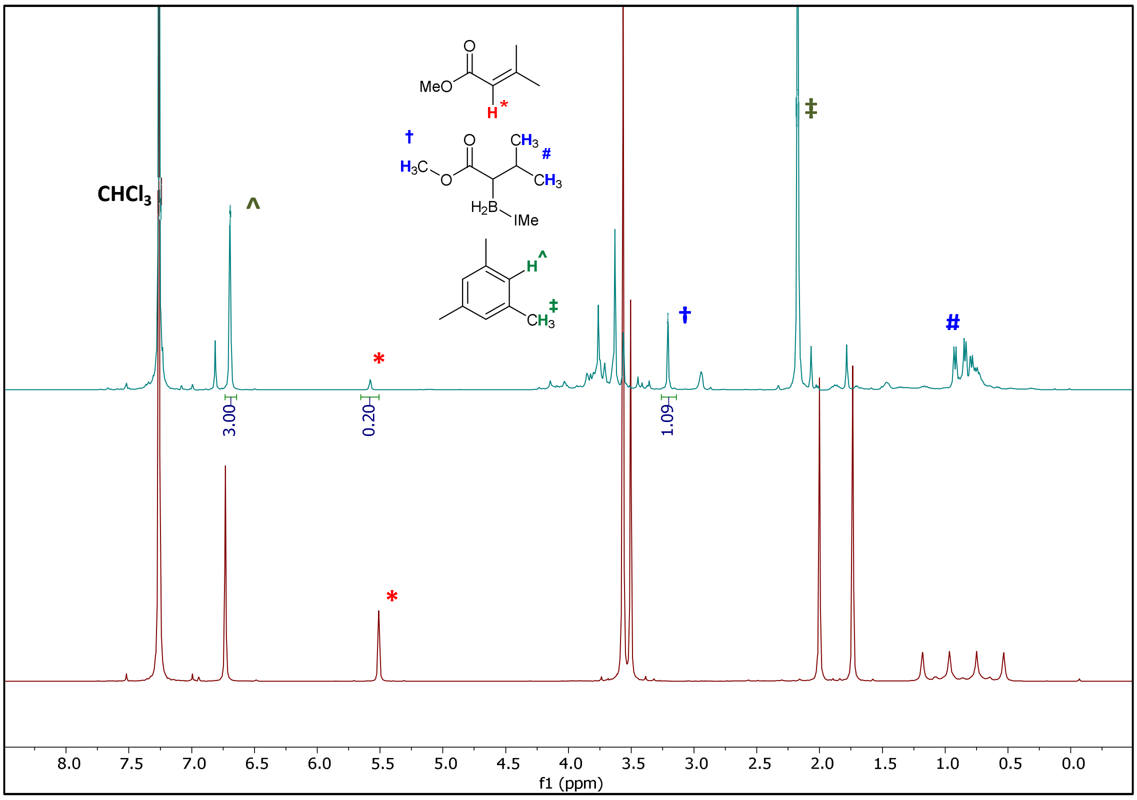

Figure S93: Collected ^{1}H NMR spectra ($CHCl_3$, 400 MHz, 298K with capillary insert containing C_6D_6) of: IMe-BH_3 and methyl 3-methylbut-2-enoate (bottom); the reaction mixture 20 hours after the addition of I_2, with 1 eq. mesitylene as a standard (top).

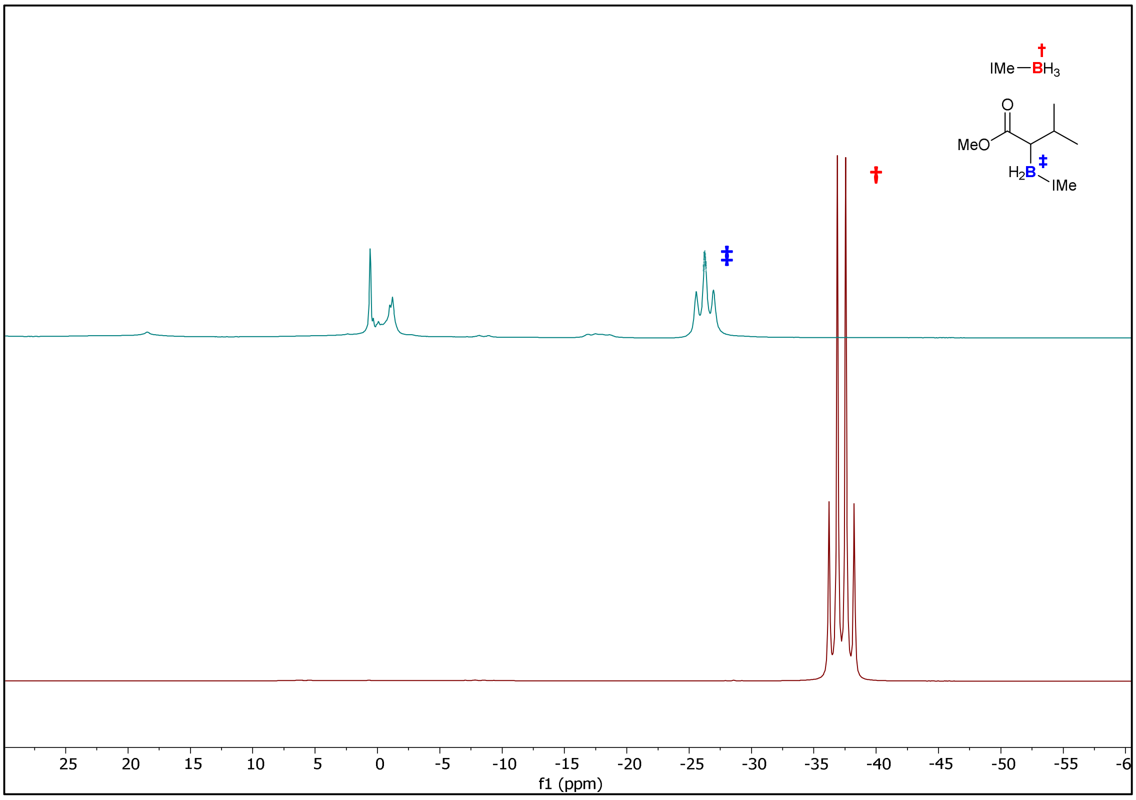

Figure S94: Collected ^{11}B NMR spectra ($CHCl_3$, 128.4 MHz, 298K) of: IMe-BH_3 and methyl 3-methylbut-2-enoate (bottom); the reaction mixture 20 hours after the addition of I_2, with 1 eq. mesitylene as a standard (top).

14.14 **Pyranone**

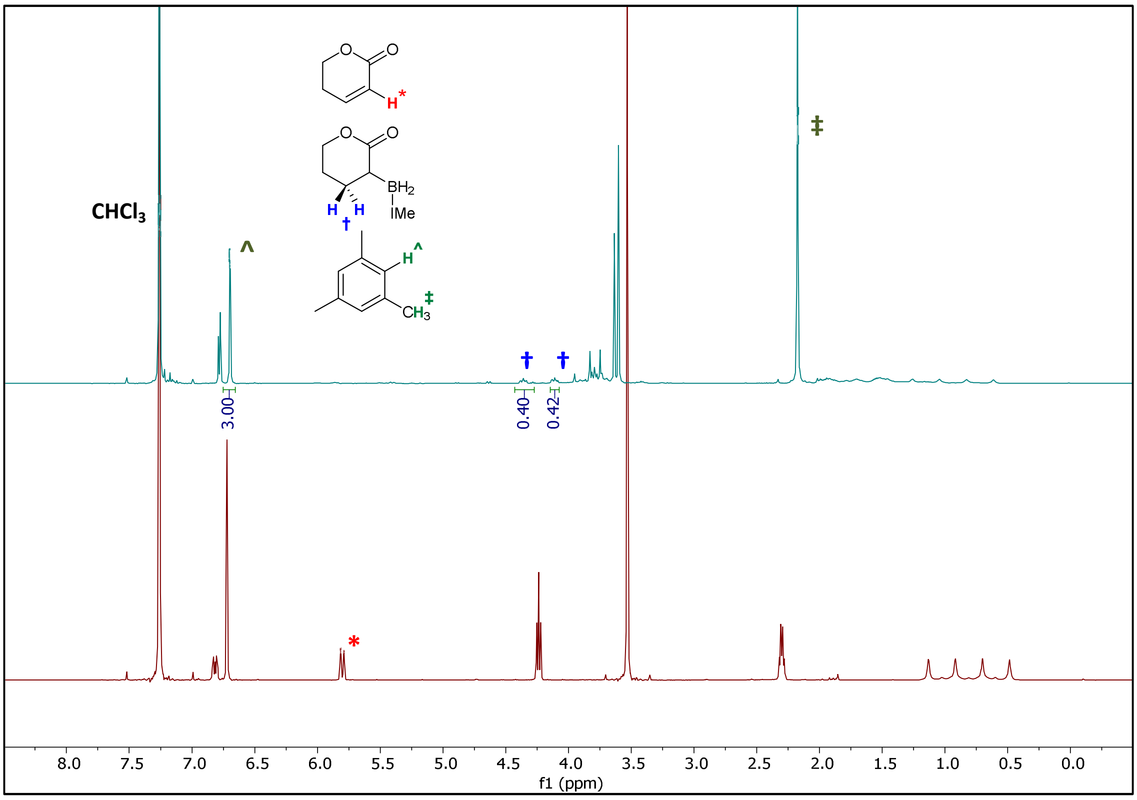

Figure S95: Collected ^{1}H NMR spectra ($CHCl_3$, 400 MHz, 298K with capillary insert containing C_6D_6) of: IMe-BH_3 and 5,6-dihydro-2H-pyran-2-one (bottom); the reaction mixture 20 hours after the addition of I_2, with 1 eq. mesitylene as a standard (top).

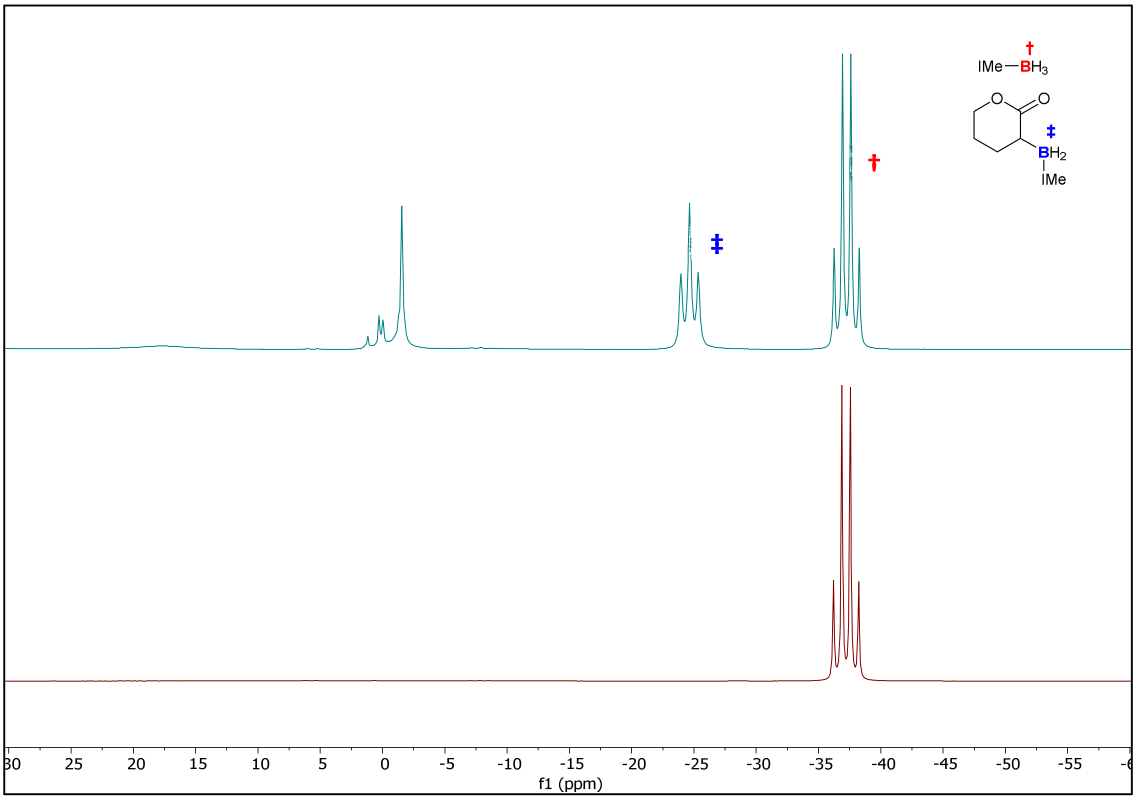

Figure S96: Collected ^{11}B NMR spectra ($CHCl_3$, 128.4 MHz, 298K) of: IMe-BH_3 and 5,6-dihydro-2H-pyran-2-one (bottom); the reaction mixture 20 hours after the addition of I_2, with 1 eq. mesitylene as a standard (top).

14.15 **Methyl tiglate**

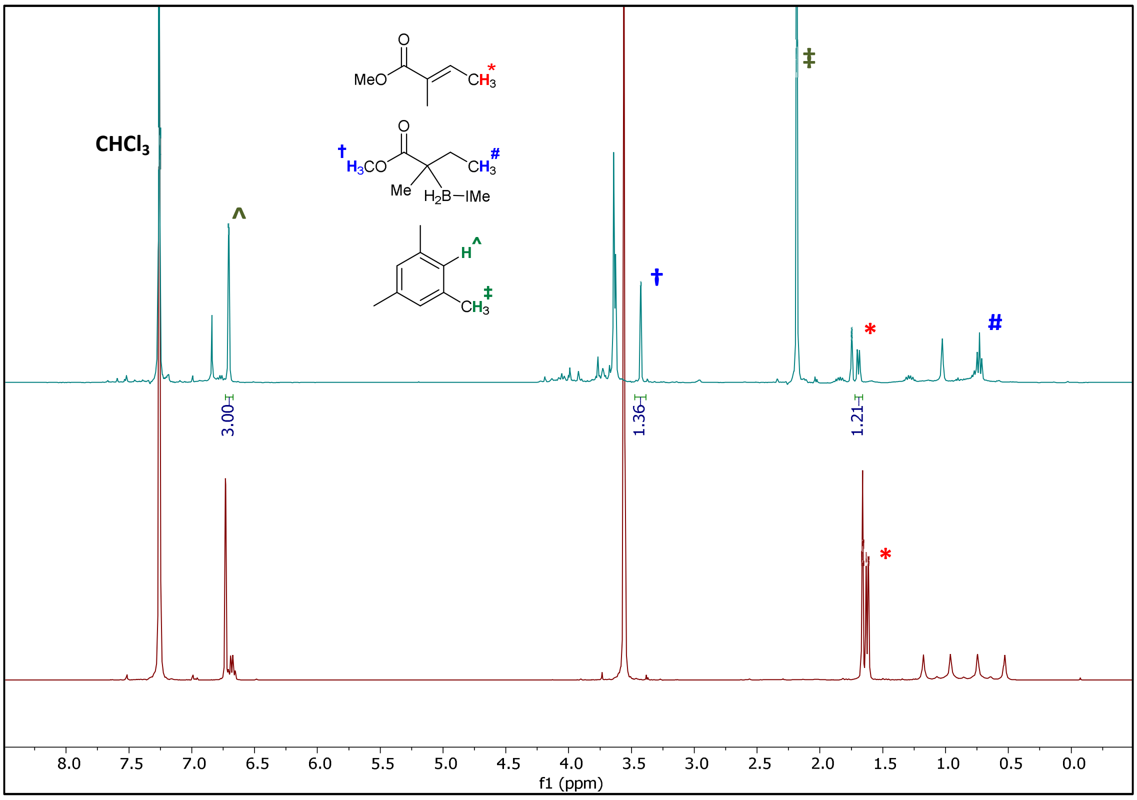

Figure S97: Collected ^{1}H NMR spectra ($CHCl_3$, 400 MHz, 298K with capillary insert containing C_6D_6) of: IMe-BH_3 and methyl tiglate (bottom); the reaction mixture 20 hours after the addition of I_2, with 1 eq. mesitylene as a standard (top).

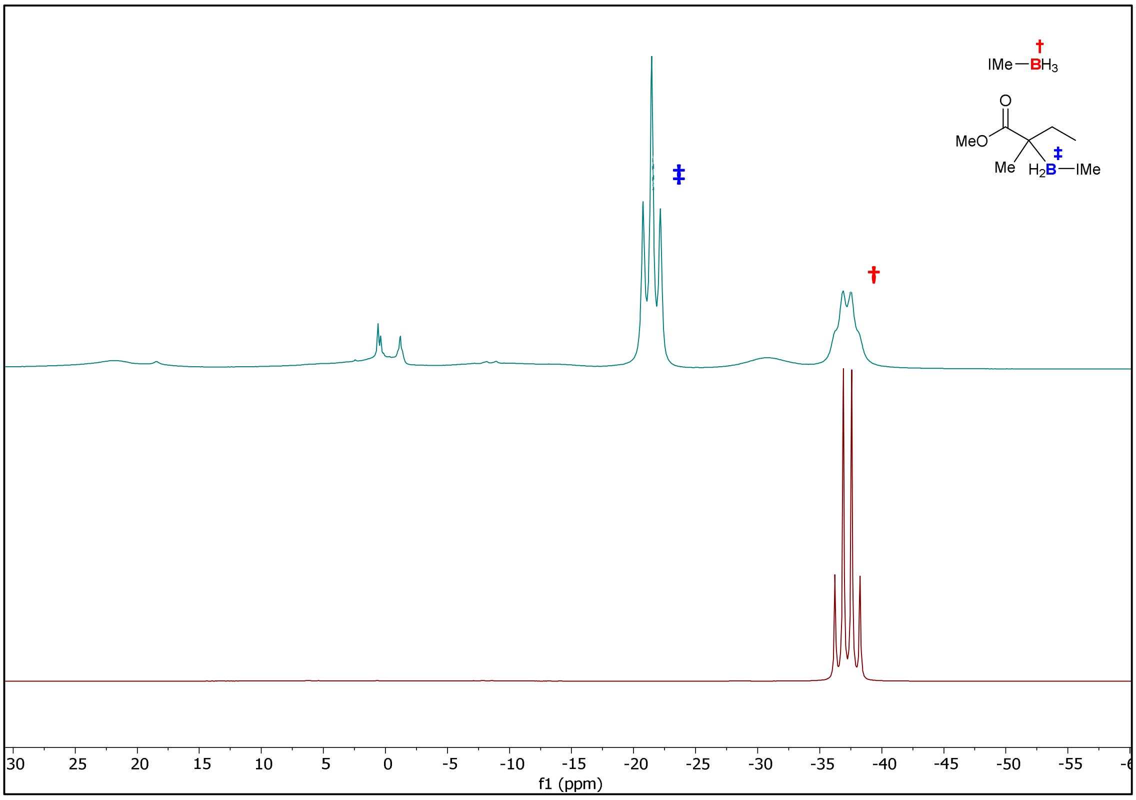

Figure S98: Collected ^{11}B NMR spectra ($CHCl_3$, 128.4 MHz, 298K) of: IMe-BH_3 and methyl tiglate (bottom); the reaction mixture 20 hours after the addition of I_2, with 1 eq. mesitylene as a standard (top).

14.16 **Methyl pentenoate**

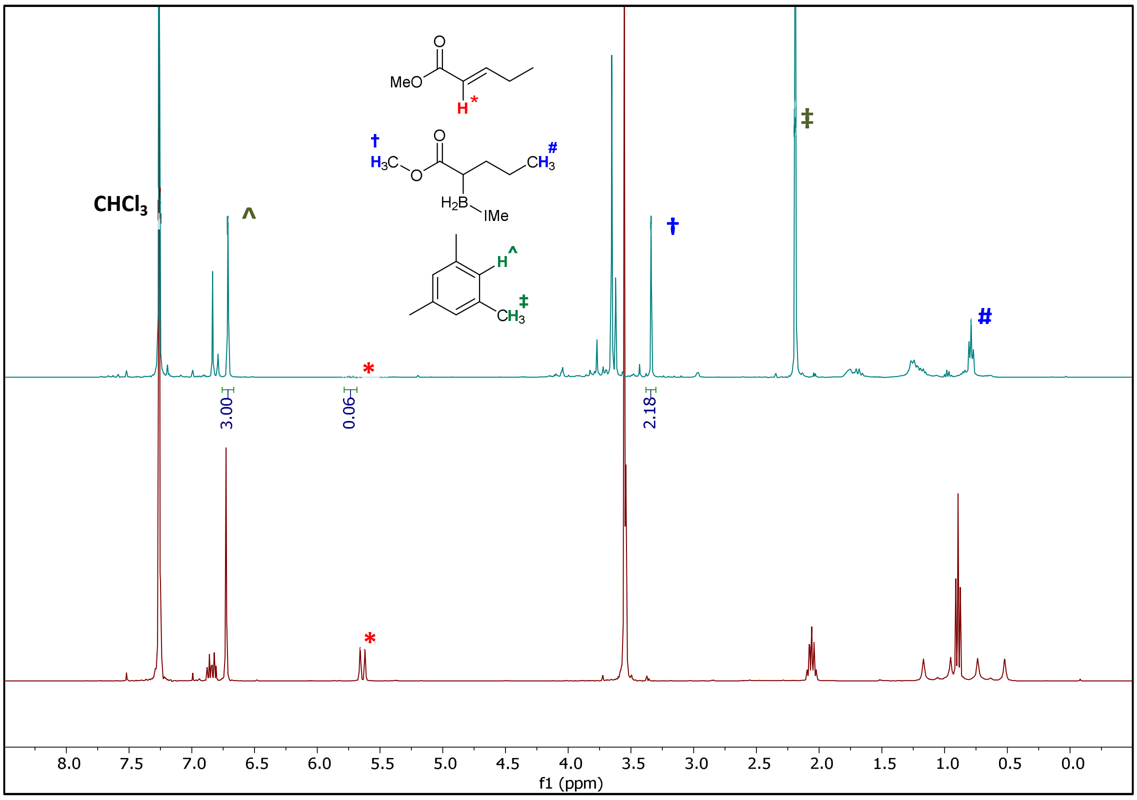

Figure S99: Collected ^{1}H NMR spectra ($CHCl_3$, 400 MHz, 298K with capillary insert containing C_6D_6) of: IMe-BH_3 and methyl pentenoate (bottom); the reaction mixture 20 hours after the addition of I_2, with 1 eq. mesitylene as a standard (top).

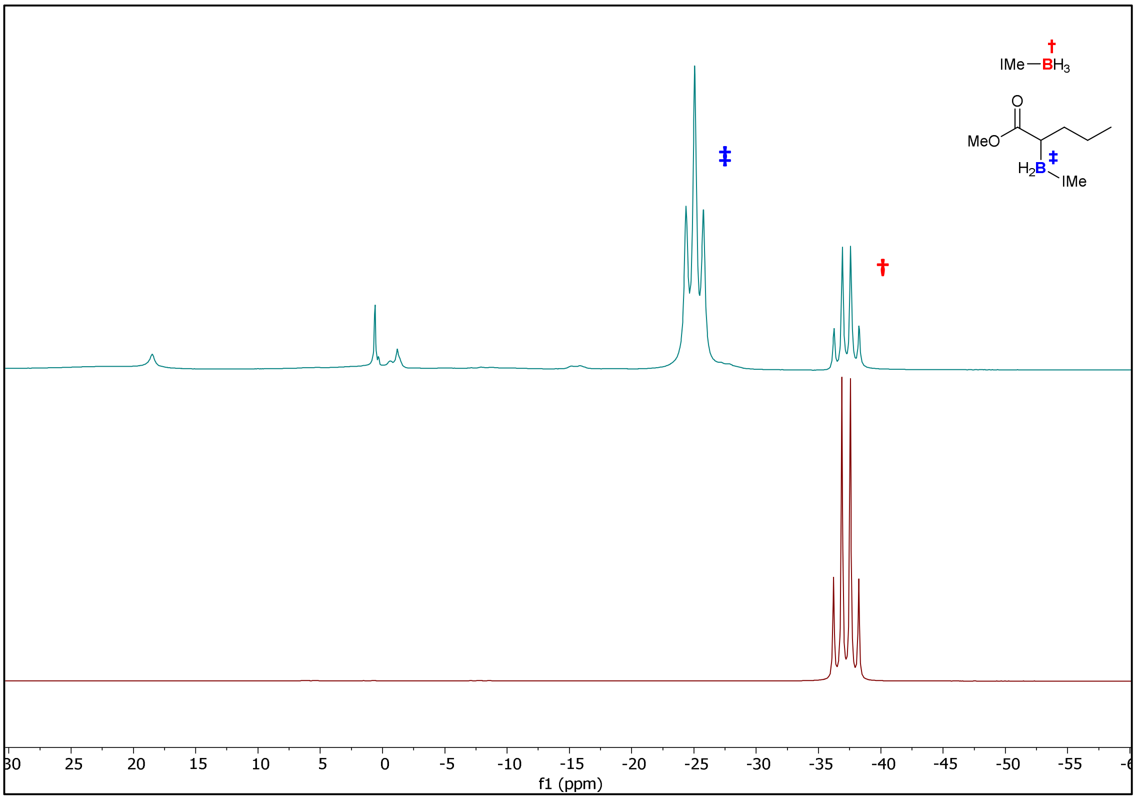

Figure S100: Collected ^{11}B NMR spectra ($CHCl_3$, 128.4 MHz, 298K) of: IMe-BH_3 and methyl pentenoate (bottom); the reaction mixture 20 hours after the addition of I_2, with 1 eq. mesitylene as a standard (top).

14.17 **Methyl 4-methyl pentenoate**

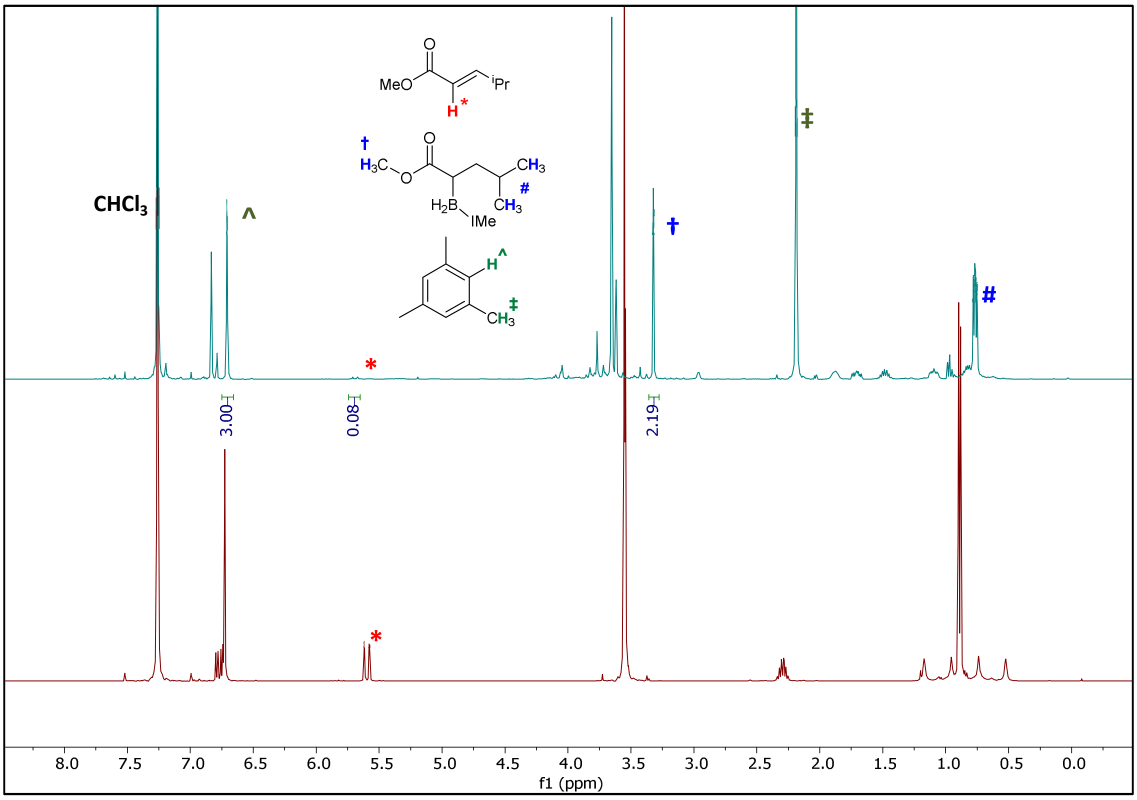

Figure S101: Collected ^{1}H NMR spectra ($CHCl_3$, 400 MHz, 298K with capillary insert containing C_6D_6) of: IMe-BH_3 and methyl 4-methylpentenoate (bottom); the reaction mixture 20 hours after the addition of I_2, with 1 eq. mesitylene as a standard (top).

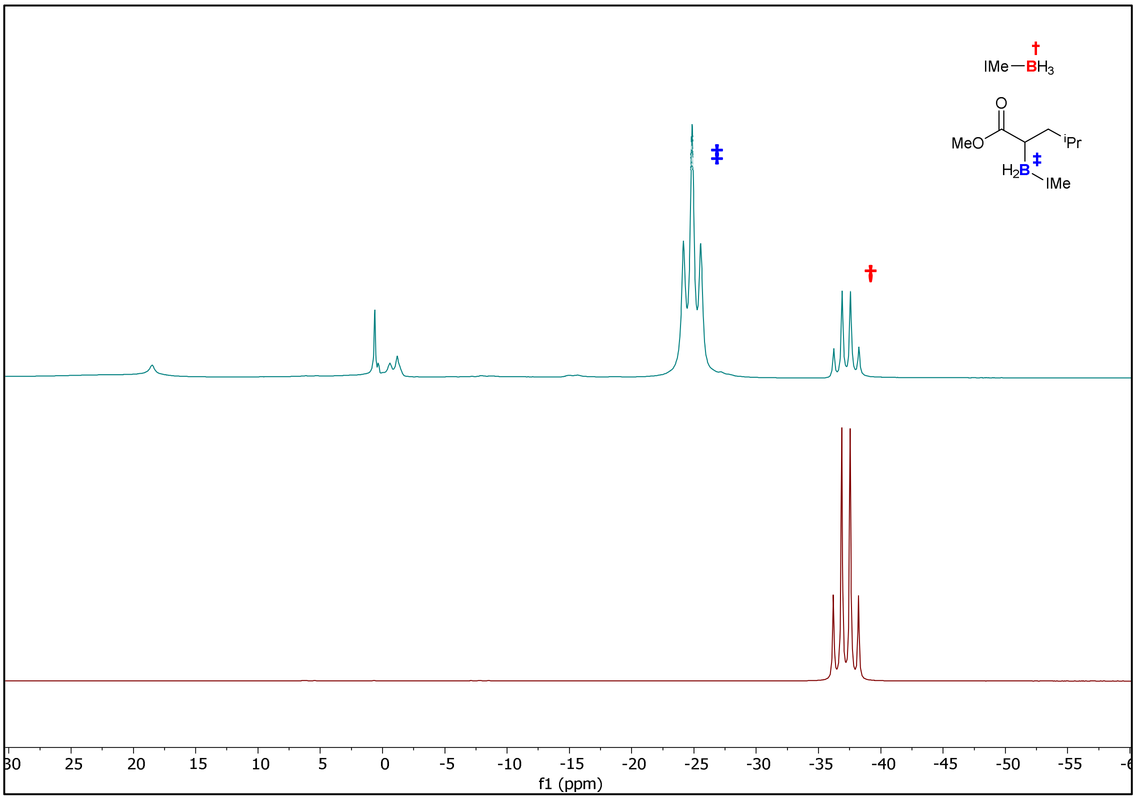

Figure S102: Collected ^{11}B NMR spectra ($CHCl_3$, 128.4 MHz, 298K) of: IMe-BH_3 and methyl 4-methylpentenoate (bottom); the reaction mixture 20 hours after the addition of I_2, with 1 eq. mesitylene as a standard (top).

14.18 **Methyl-Para-chloro cinnamate**

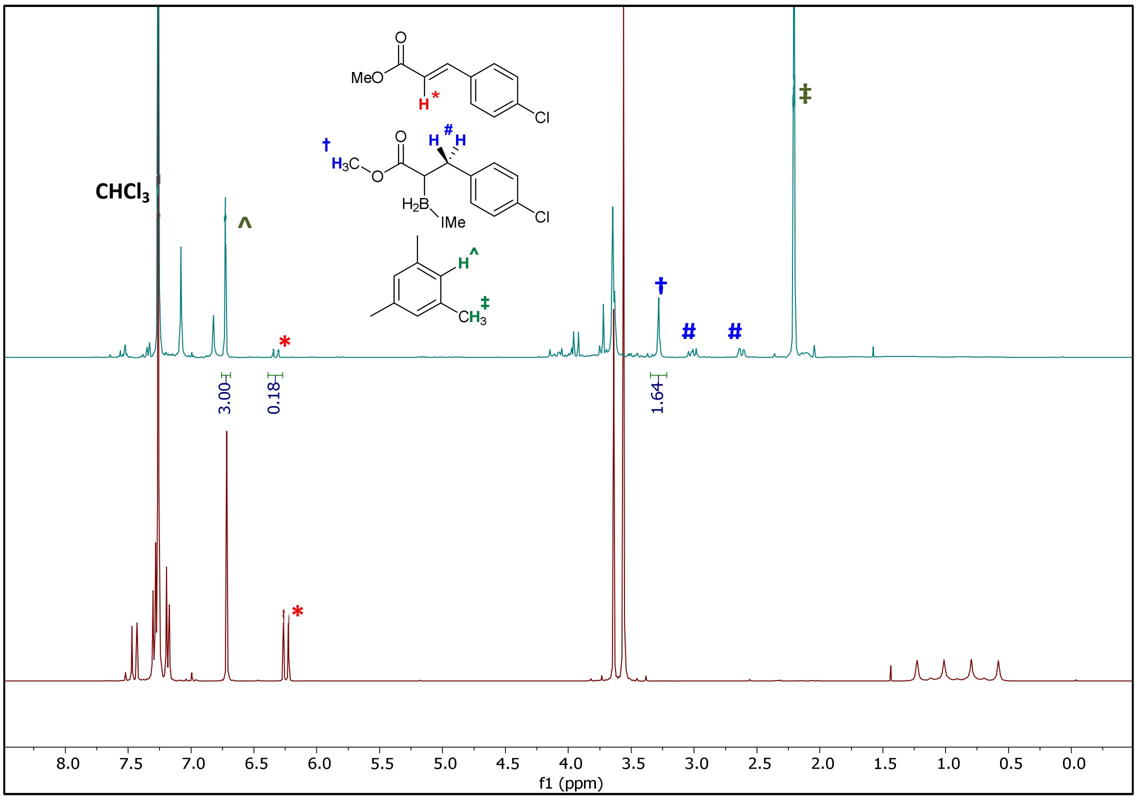

Figure S103: Collected ^{1}H NMR spectra ($CHCl_3$, 400 MHz, 298K with capillary insert containing C_6D_6) of: IMe-BH_3 and methyl 4-chlorocinnamate (bottom); the reaction mixture 20 hours after the addition of I_2, with 1 eq. mesitylene as a standard (top).

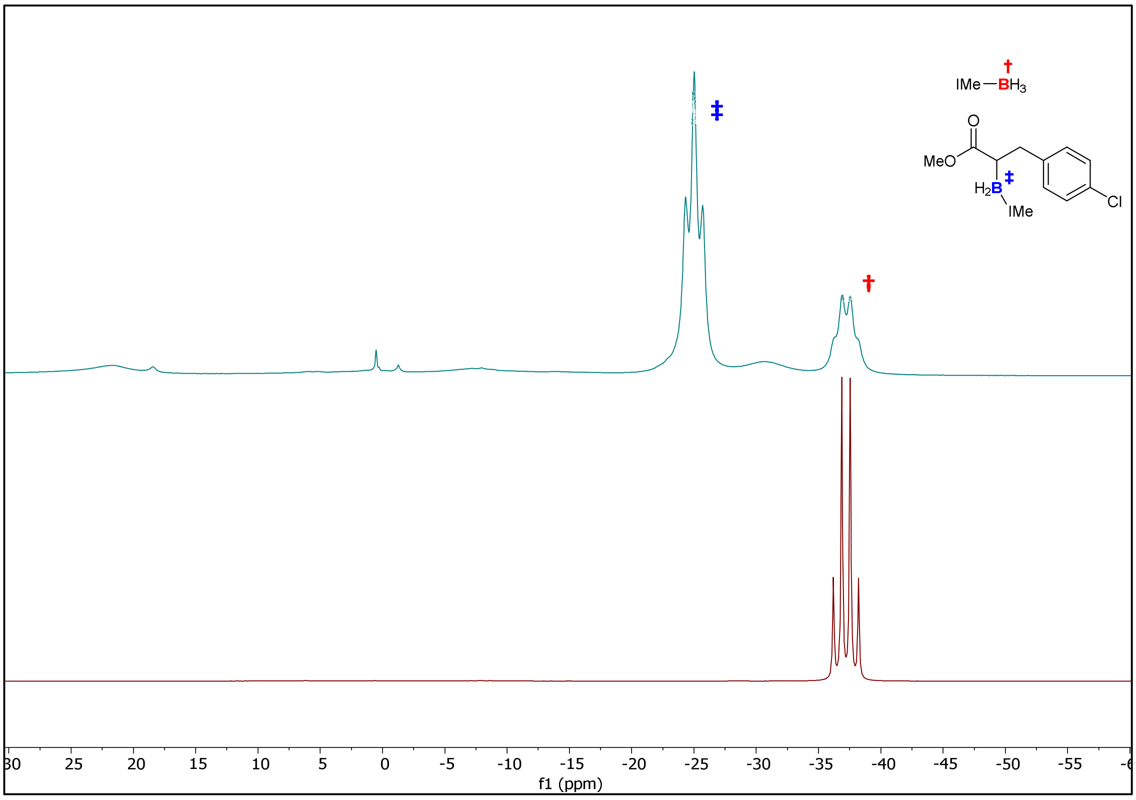

Figure S104: Collected ^{11}B NMR spectra ($CHCl_3$, 128.4 MHz, 298K) of: IMe-BH_3 and methyl 4-chlorocinnamate (bottom); the reaction mixture 20 hours after the addition of I_2, with 1 eq. mesitylene as a standard (top).

14.19 **Methyl para-bromo cinnamate**

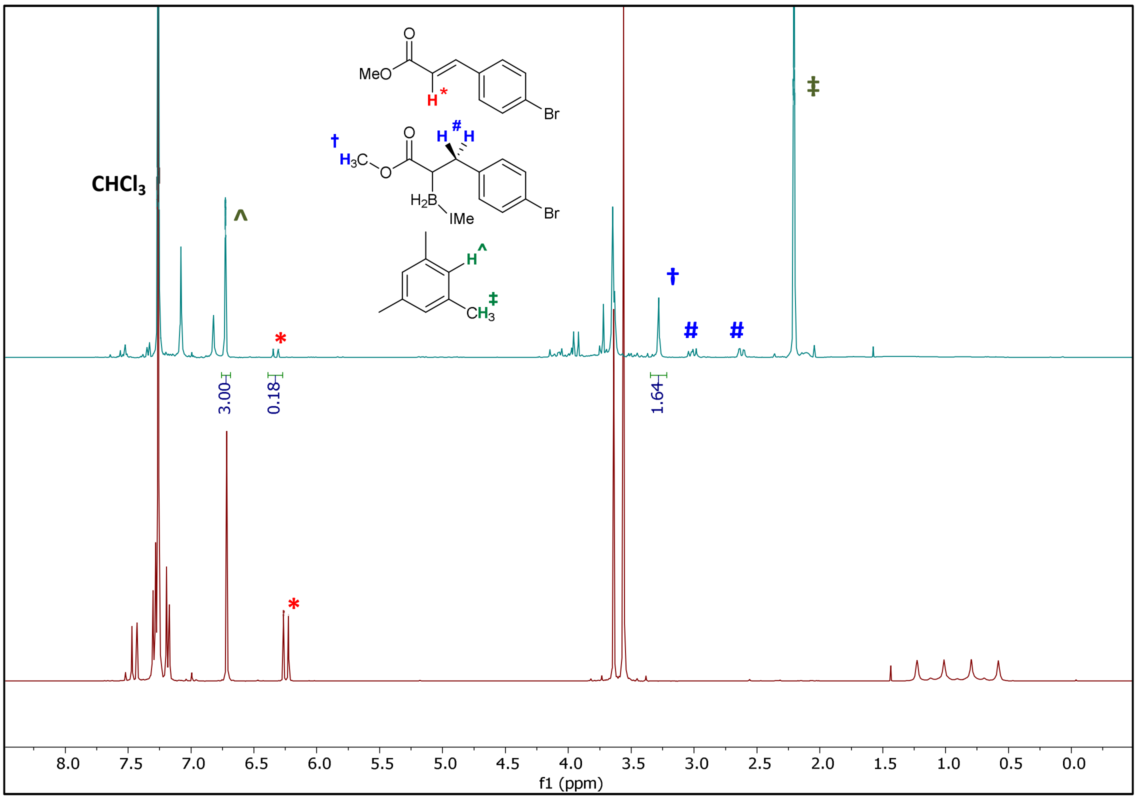

Figure S105: Collected ^{1}H NMR spectra ($CHCl_3$, 400 MHz, 298K with capillary insert containing C_6D_6) of: IMe-BH_3 and methyl 4-bromocinnamate (bottom); the reaction mixture 20 hours after the addition of I_2, with 1 eq. mesitylene as a standard (top).

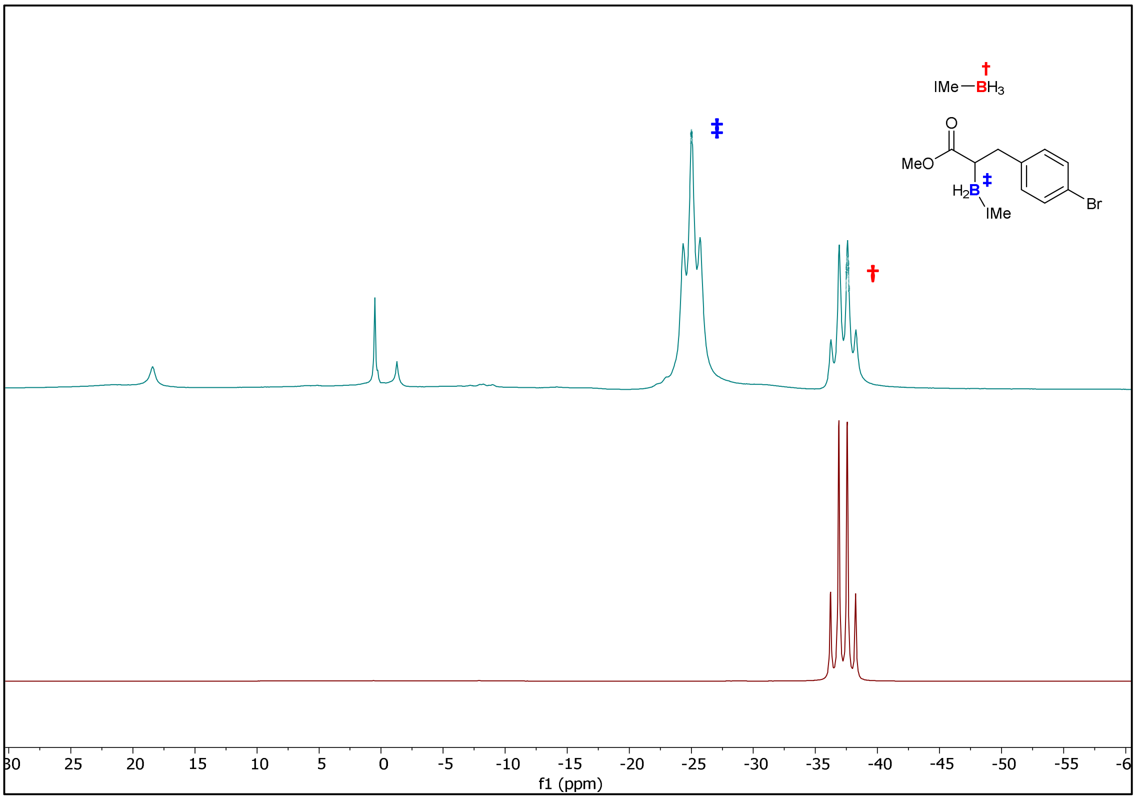

Figure S106: Collected ^{11}B NMR spectra ($CHCl_3$, 128.4 MHz, 298K) of: IMe-BH_3 and methyl 4-bromocinnamate (bottom); the reaction mixture 20 hours after the addition of I_2, with 1 eq. mesitylene as a standard (top).

14.20 **Ethyl para-nitro cinnamate**

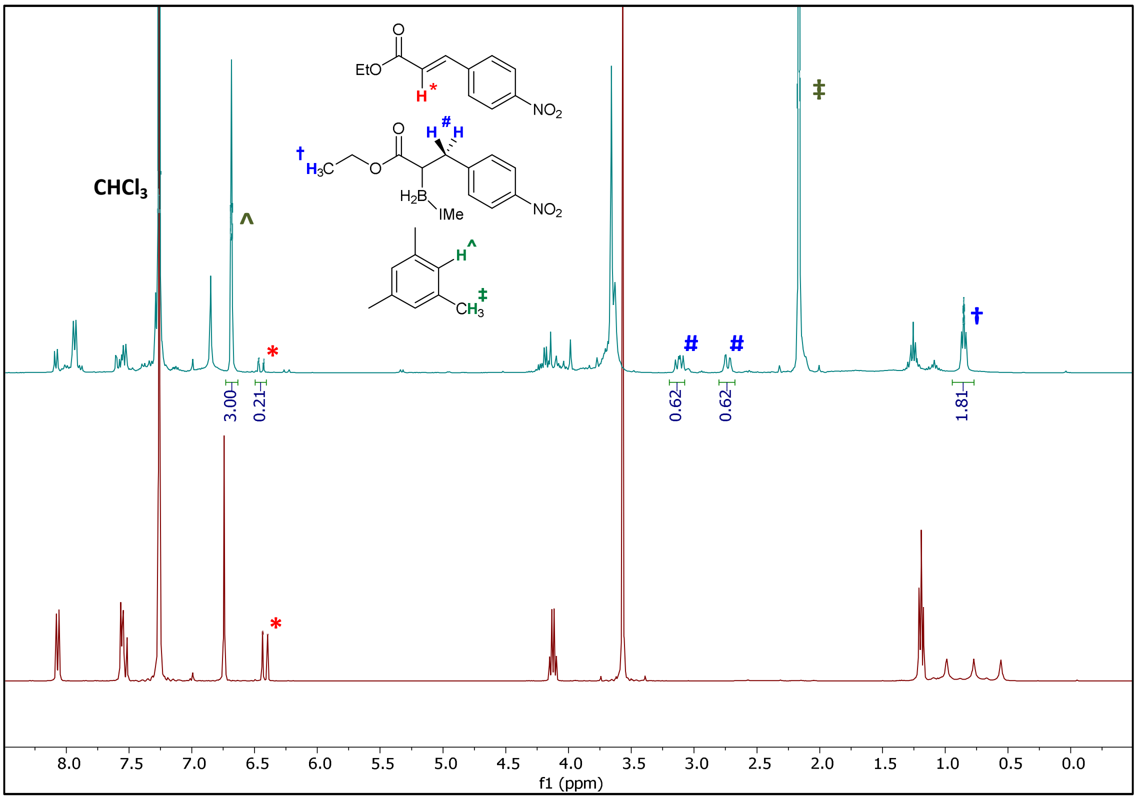

Figure S107: Collected ^{1}H NMR spectra ($CHCl_3$, 400 MHz, 298K with capillary insert containing C_6D_6) of: IMe-BH_3 and ethyl 4-nitrocinnamate (bottom); the reaction mixture 20 hours after the addition of I_2, with 1 eq. mesitylene as a standard (top).

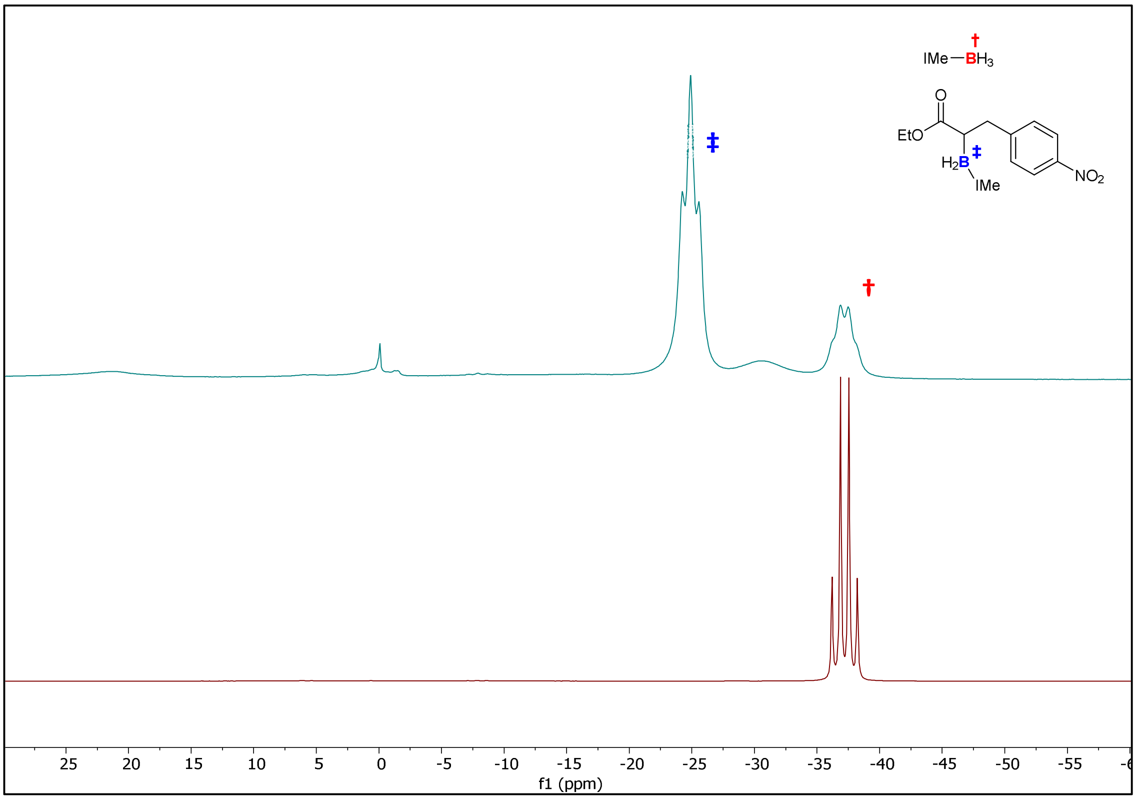

Figure S108: Collected ^{11}B NMR spectra ($CHCl_3$, 128.4 MHz, 298K) of: IMe-BH_3 and ethyl 4-nitrocinnamate (bottom); the reaction mixture 20 hours after the addition of I_2, with 1 eq. mesitylene as a standard (top).

14.21 **4-methyl methyl cinnamate**

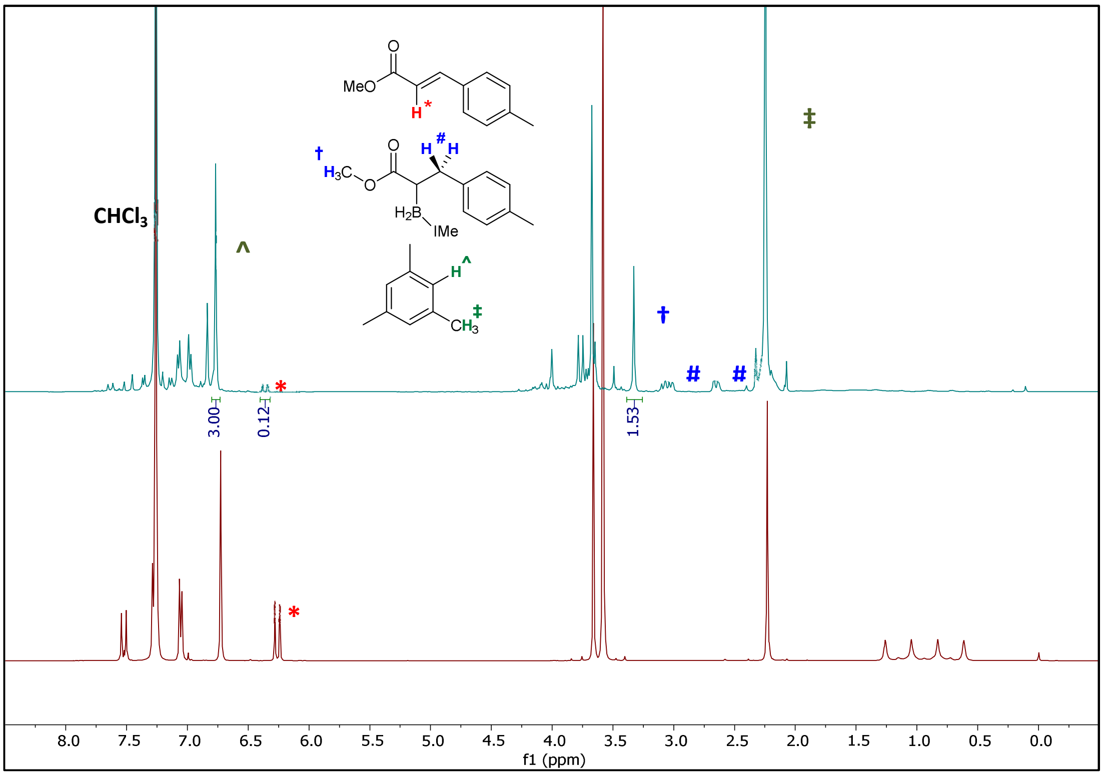

Figure S109: Collected ^{1}H NMR spectra ($CHCl_3$, 400 MHz, 298K with capillary insert containing C_6D_6) of: IMe-BH_3 and 4-methyl methyl cinnamate (bottom); the reaction mixture 20 hours after the addition of I_2, with 1 eq. mesitylene as a standard.

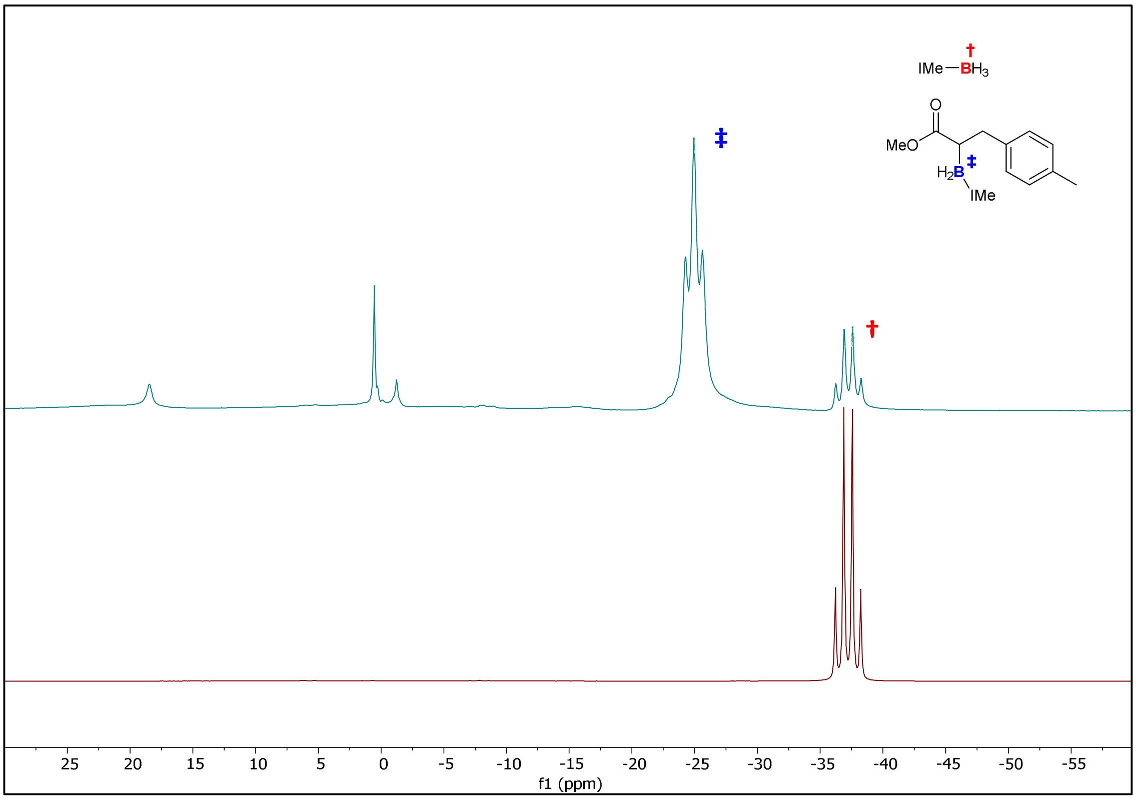

Figure S110: Collected ^{11}B NMR spectra ($CHCl_3$, 128.4 MHz, 298K) of: IMe-BH_3 and 4-methyl methyl cinnamate (bottom); the reaction mixture 20 hours after the addition of I_2, with 1 eq. mesitylene as a standard (top).

14.22 **Furanone**

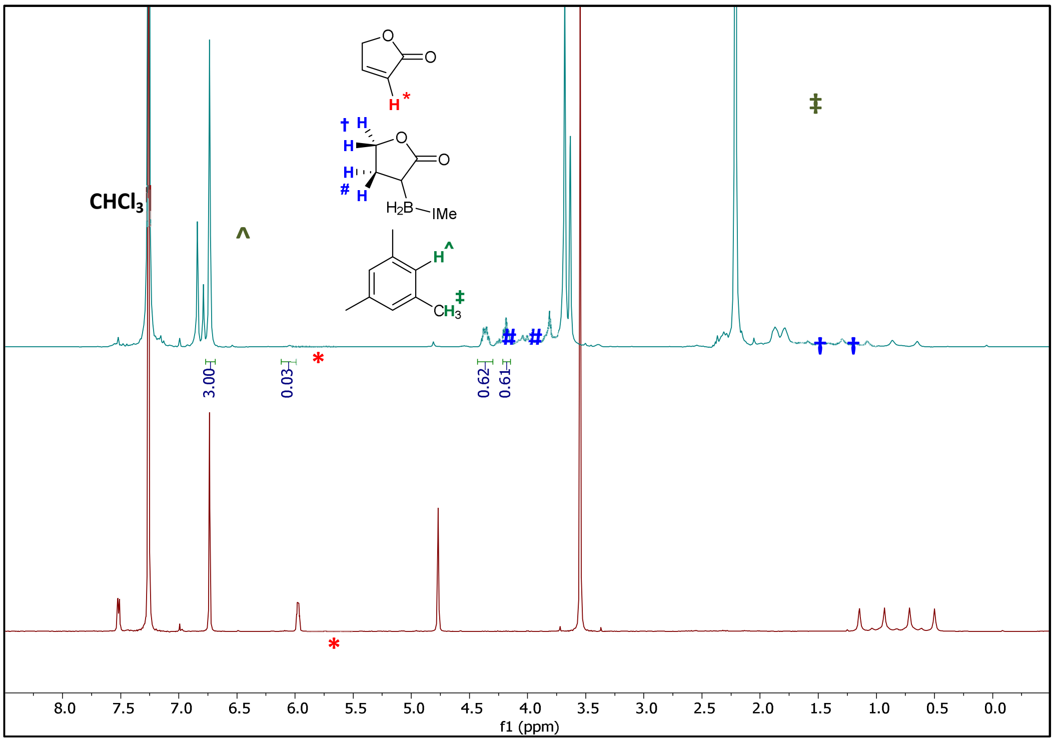

Figure S111: Collected ^{1}H NMR spectra ($CHCl_3$, 400 MHz, 298K with capillary insert containing C_6D_6) of: IMe-BH_3 and 2-furanone (bottom); the reaction mixture 20 hours after the addition of I_2, with 1 eq. mesitylene as a standard (top).

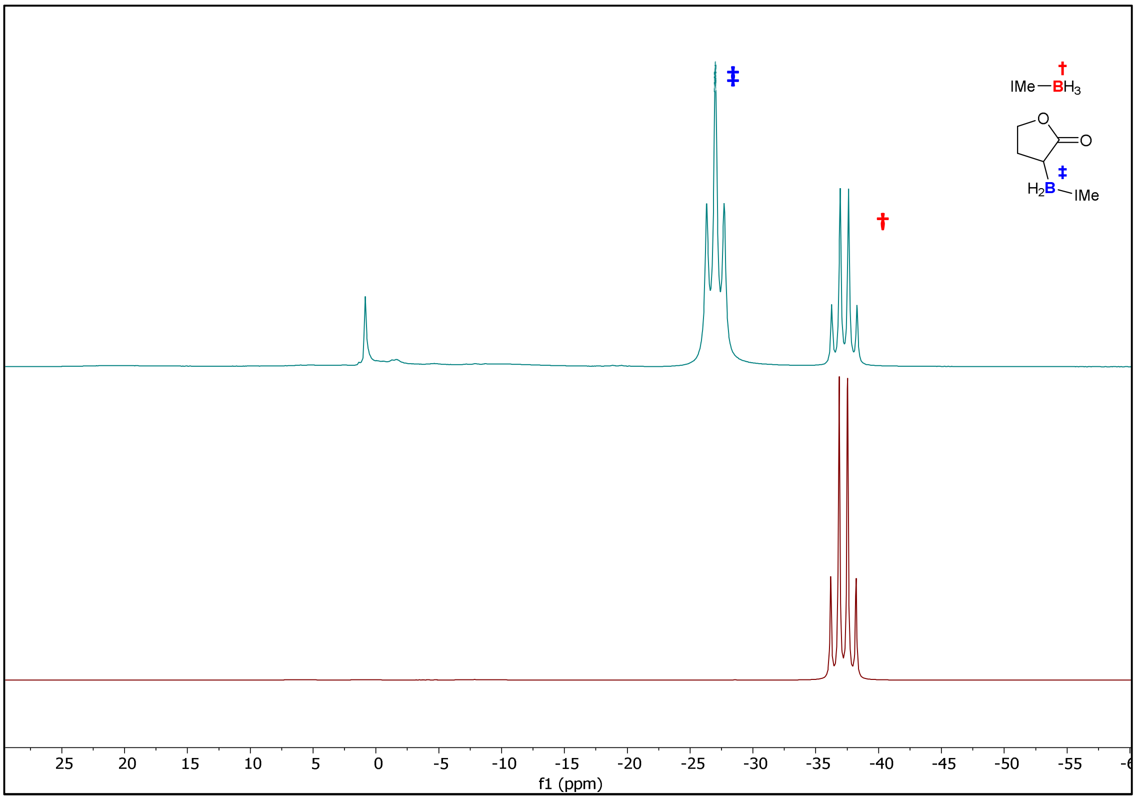

Figure S112: Collected ^{11}B NMR spectra ($CHCl_3$, 128.4 MHz, 298K) of: IMe-BH_3 and 2-furanone (bottom); the reaction mixture 20 hours after the addition of I_2, with 1 eq. mesitylene as a standard (top).

14.23 **4-phenyl furanone**

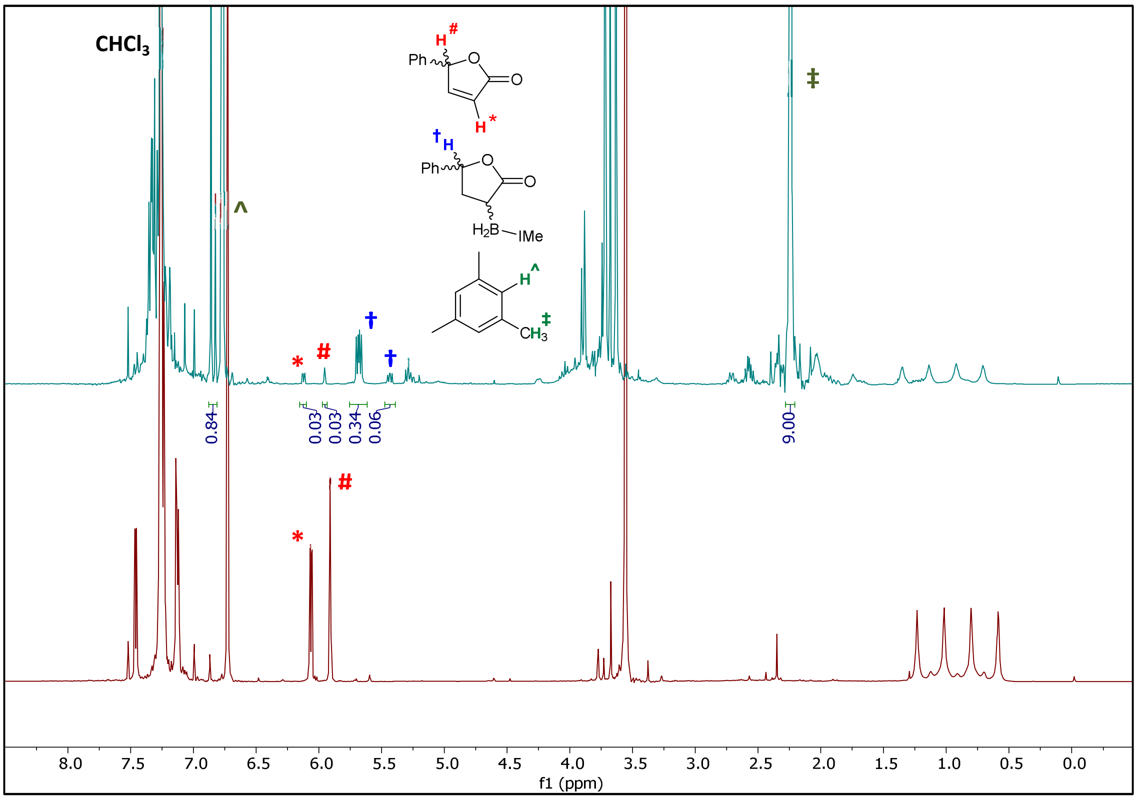

Figure S113: Collected ^{1}H NMR spectra ($CHCl_3$, 400 MHz, 298K with capillary insert containing C_6D_6) of: IMe-BH_3 and 5-phenyl-2(5H)-furanone (bottom); the reaction mixture 20 hours after the addition of I_2, with 1 eq. mesitylene as a standard.

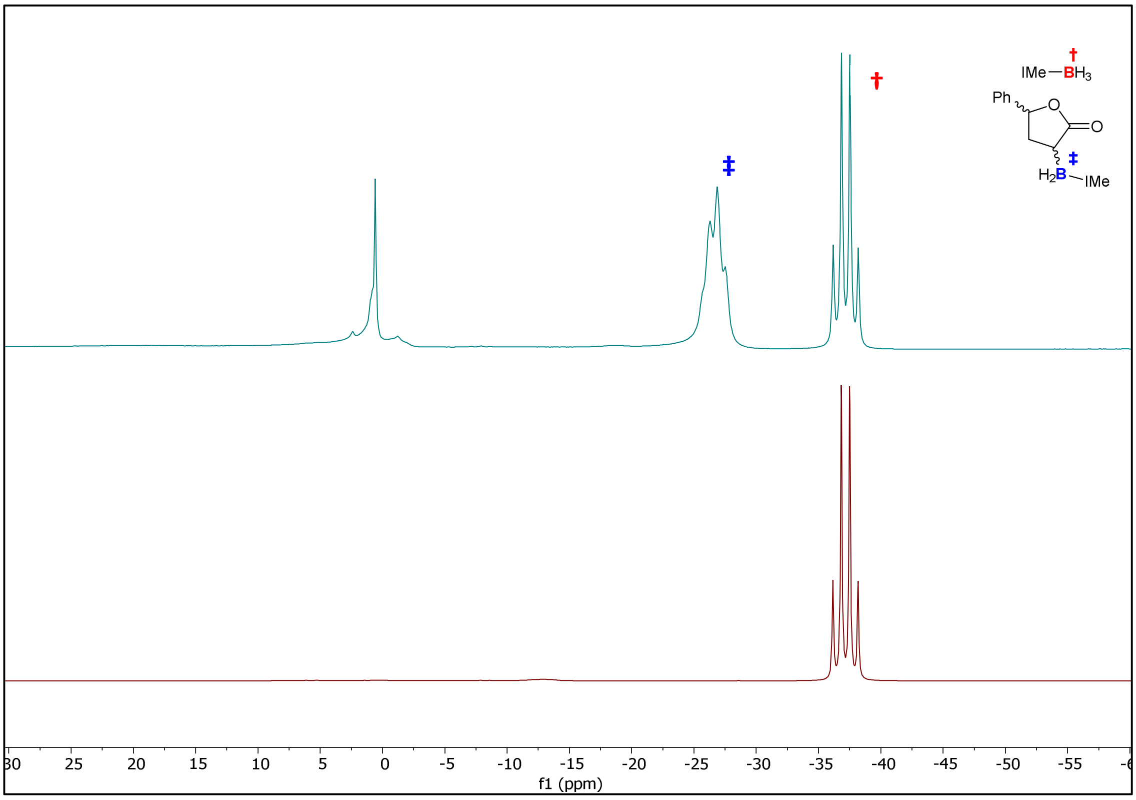

Figure S114: Collected ^{11}B NMR spectra ($CHCl_3$, 128.4 MHz, 298K) of: IMe-BH_3 and 5-phenyl-2(5H)-furanone (bottom); the reaction mixture 20 hours after the addition of I_2, with 1 eq. mesitylene as a standard (top).

15 References

[1] Gardener, S., Kawamoto, T., Curran, D. P., *J. Org. Chem*. **2015**, *80*, 9794–9797

[2] Walton, J. C., Brahmi, M. M., Fensterbank, L., Lacôte, E., Malacria, M., Chu, Q., Ueng, S.-H., Solovyev, A., Curran, D. P., *J. Am. Chem. Soc*., **2010**, *132*, 2350–2358

[3] Kim, Y., Chang, S., *Angew. Chem. Int. Ed*. **2016**, *55*, 218 –222

[4] Sakakura, A., Kawajiri, K., Ohkubo, T., Kosugi, Y., Ishihara, K., *J. Am. Chem. Soc*., **2007**, *129*, 14775-14779

[5] Muñiz, K., Iesato, A., Neiger, M., *Chem. Eur. J*., **2003**, *9*, 5581-5596

[6] Shimoi, M.; Watanabe, T.; Maeda, K.; Curran, D. P.; Taniguchi, T., *Angew. Chem. Int. Ed*. **2018**, *57*, 9485-9490

[7] Li, X.; Curran, D. P., *J. Am. Chem. Soc*. **2013**, *135*, 12076-12081

[8] Allen, T. H., Kawamoto, T., Gardner, S., Geib, S. J., Curran, D. P., *Org. Lett*., **2017**, 19, 3680-2683.

[9] Karplus M., *J. Am. Chem. Soc*., **1963**, *85*, 2870-2871

[10] Haasnoot C.A.G., De Leeuw F.A.A.M., Altona C., *Tetrahedron,* **1980**, *36*, 2783-2792

[11] Foroozandeh, M., Adams, R.W., Kiraly, P., Nilsson, M., Morris, G.A., *Chem. Commun*. **2015**, 15410-15413

[12] Nerkar, S., Curran, D. P., *Org. Lett*, **2015**, *17*, 3394-3397

[13] Bose, S.K., Fucke, K., Liu, L., Steel, P.G., Marder, T.B., *Angew. Chem. Int. Ed*. **2014**, *53*, 1799-1803

[14] Lee, Y.; Hoveyda, A. H., *J. Am. Chem. Soc.* **2009**, *131*, 3160-3161

[15] T. Kliman, S. N. Mlynarski, J. P. Morken, *J. Am. Chem. Soc.* **2009**, *131*, 13210-13211

[16] Gaussian 09, Revision D.01, Frisch, M. J.; Trucks, G. W.; Schlegel, H. B.; Scuseria, G. E.; Robb, M. A.; Cheeseman, J. R.; Scalmani, G.; Barone, V.; Mennucci, B.; Petersson, G. A.; Nakatsuji, H.; Caricato, M.; Li, X.; Hratchian, H. P.; Izmaylov, A. F.; Bloino, J.; Zheng, G.; Sonnenberg, J. L.; Hada, M.; Ehara, M.; Toyota, K.; Fukuda, R.; Hasegawa, J.; Ishida, M.; Nakajima, T.; Honda, Y.; Kitao, O.; Nakai, H.; Vreven, T.; Montgomery, Jr., J. A.; Peralta, J. E.; Ogliaro, F.; Bearpark, M.; Heyd, J. J.; Brothers, E.; Kudin, K. N.; Staroverov, V. N.; Kobayashi, R.; Normand, J.; Raghavachari, K.; Rendell, A.; Burant, J. C.; Iyengar, S. S.; Tomasi, J.; Cossi, M.; Rega, N.; Millam, J. M.; Klene, M.; Knox, J. E.; Cross, J. B.; Bakken, V.; Adamo, C.; Jaramillo, J.; Gomperts, R.; Stratmann, R. E.; Yazyev, O.; Austin, A. J.; Cammi, R.; Pomelli, C.; Ochterski, J. W.; Martin, R. L.; Morokuma, K.; Zakrzewski, V. G.; Voth, G. A.; Salvador, P.; Dannenberg, J. J.; Dapprich, S.; Daniels, A. D.; Farkas, Ö.; Foresman, J. B.; Ortiz, J. V.; Cioslowski, J.; Fox, D. J. Gaussian, Inc., Wallingford CT, **2009**

[17] CrysAlisPro, Agil. Technol. Version 1.1 71.35.19 (release 27-10-2011 CrysAlis171.NET) (compiled Oct 27 2011,150211)

[18] Dolomanov, O. V., Bourhis, L. J., Gildea, R. J., Howard, J. A. K., Puschmann, H., *J. Appl. Crystallogr.* **2009**, *42*, 339-341

[19] Palatinus, L., Chapuis, G., *J. Appl. Crystallogr*. **2007**, *40*, 786-790

[20] Palatinus, L., Van der Lee, A., J. *Appl. Crystallogr*. **2008**, *41*, 975-984

[21] Palatinus, A., Prathapa, S. J., Van Smaalen, S., *J. Appl. Crystallogr*. **2012**, *45*, 575-580

[22] Sheldrick, G. M. *Acta Crystallogr. Sect. A Found. Crystallogr*. **2008**, *64*, 112-122
